# Global research hotspots and trends on robotic surgery in obstetrics and gynecology: a bibliometric analysis based on VOSviewer

**DOI:** 10.3389/fsurg.2024.1308489

**Published:** 2024-02-09

**Authors:** Peichen Xiao, Lu Li, Jinfeng Qu, Guangxin Wang

**Affiliations:** ^1^ Department of Obstetrics and Gynecology, Jinan Central Hospital, Shandong University, Jinan, China; ^2^ Innovation Center of Intelligent Diagnosis, Jinan Central Hospital, Shandong University, Jinan, China; ^3^ Shandong Innovation Center of Intelligent Diagnosis, Central Hospital Affiliated to Shandong First Medical University, Jinan, China

**Keywords:** robotic surgery, obstetrics and gynecology, bibliometric analysis, Web of Science, VOSviewer

## Abstract

**Objective:**

Over the last two decades, the quantity of papers published in relation to robotic surgery in obstetrics and gynecology has continued to grow globally. However, no bibliometric analysis based on VOSviewer has been performed to evaluate the past and present of global research in the field. In this study, we aimed to analyze the bibliometric characteristics of papers on robotic surgery in obstetrics and gynecology to reveal research hotspots and trends in this field.

**Methods:**

The Web of Science Core Collection was searched for scientific papers on robotic surgery in obstetrics and gynecology published between January 1, 1998 and December 31, 2023. Bibliometric metadata of each selected paper was extracted for analysis. The results were visualized by VOSviewer (version 1.6.18).

**Results:**

A total of 1,430 papers met the inclusion criteria. The United States had the highest total link strengths and contributed the most papers (*n* = 793). The Mayo Clinic produced the largest number of papers (*n* = 85), and Professor Pedro T Ramirez contributed the most papers (*n* = 36). The number of citations ranged from 0 to 295 with a total sum of 29,103. The *Journal of Minimally Invasive Gynecology* published the most relevant papers (*n* = 252). Keywords were classified into six clusters based on co-occurrence data, of which cluster 1, cluster 4 and cluster 6 had more main keywords with the largest average publication year.

**Conclusions:**

This is the first VOSviewer-based bibliometric analysis of robotic surgery research in obstetrics and gynecology. The United States was the leading country, and the *Journal of Minimally Invasive Gynecology* was the most productive journal in the field. Scientists and institutions from around the world should push their boundaries to bring about deep collaboration. The main research topic has always been the use of robotic surgery in the treatment of gynecologic malignancies. More randomized controlled trials need to be conducted to compare surgical outcomes of robotic surgery with other surgical approaches. Robotic sacrocolpopexy for pelvic organ prolapse has become a new research hotspot, and robotic surgery for sentinel lymph node detection in gynecologic malignancies are more potential directions for future research.

## 1 Introduction

Minimally invasive surgery has gradually become a common surgical procedure with the continuous development of surgical techniques. The advent of robotic surgical systems is an exciting development in the field of minimally invasive surgery. Robotic surgical systems provide several benefits, including enhanced precision during the operation, as well as a clearer three-dimensional surgical field of view, thereby ensuring the safety of the operation (1). Additionally, surgeons can mitigate fatigue by sitting comfortably at the front of the operating table. Nevertheless, the employment of robotic surgical systems is not immune to limitations, owing to its propensity for exorbitant costs and vulnerability to signal disruption between the console and the apparatus during surgical procedures (2). In general, the trajectory of robotic surgical systems will progress toward greater cost-effectiveness, reduced weight, and enhanced stability.

The advancement of robotic surgery is closely linked to the evolution of information technology and the development of precision machine manufacturing technology. In 1994, an American company, Computer Motion, developed the Aesop system, which was the first endoscopic surgical system designed to assist in minimally invasive surgery. Although this system is not able to operate independently of instructions, it represents a pivotal advancement in the field of robotic surgery (3). In 1998, Computer Motion developed the Zeus system, which enabled voice-activated interaction for endoscope manipulation and surgical instrument operation under the guidance of a physician. The da Vinci system, a third-generation robotic system developed by Intuitive Surgical, was approved for clinical use in 2000 (3).

Research on the utilization of robotic or computer-assisted techniques in minimally invasive obstetric and gynecologic surgery has increased since the late 1990s (4). Much of that research involved the da Vinci robot, which was approved by the U.S. Food and Drug Administration as a modified laparoscopic approach for gynecologic surgery in April 2005 (5). Currently, robotic surgical systems are rapidly gaining popularity in obstetrics and gynecology. Their applications include but are not limited to sacrocolpopexy (6, 7), hysterectomy (8, 9), myomectomy (10, 11), tubal anastomosis (12, 13), and lymphadenectomy (14, 15).

In bibliometric analysis, statistical and mathematical approaches are used to measure the quality and quantity of documents, books and other communication media (16, 17). As an increasing number of scientific discoveries emerge and published studies are read and cited by other scholars, bibliometric indicators including impact factor, CiteScore, Eigenfactor score, SCImago Journal Rank, H-index, etc., have become increasingly significant (18). In addition to its widespread use in the fields of physics, chemistry, and computer science, bibliometric analysis has opened up new perspectives in the field of medicine (19–24). By analyzing bibliometric indicators, scholars can understand the influence of publications, countries, organizations, authors, and journals in a particular field (25). Moreover, the greater strength of bibliometric analysis is that it can summarize large amounts of data to report developments and emerging trends in the field (26).

Over the last two decades, the number of papers on robotic surgery in obstetrics and gynecology has grown exponentially. However, to our knowledge, no bibliometric analysis based on VOSviewer has been performed to evaluate the past and present of global research in the field. Thus, this study aimed to identify papers on robotic surgery in obstetrics and gynecology and then to analyze their bibliometric characteristics to help reveal research hotspots and trends in this field.

## 2 Materials and methods

### 2.1 Data source and search strategy

**Figure 1:**
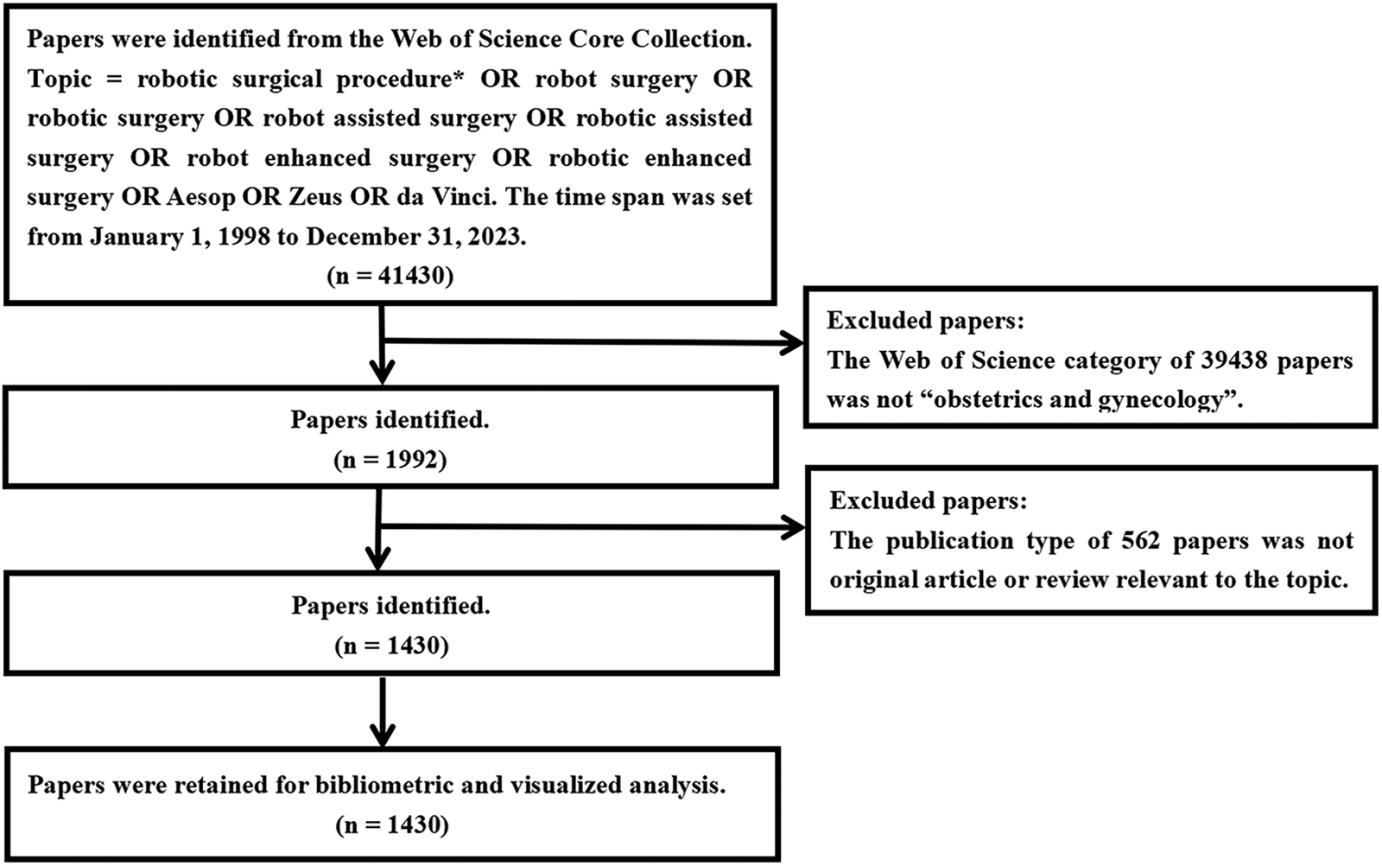
A flow diagram on inclusion and exclusion of papers related to robotic surgery in obstetrics and gynecology.

We searched for scientific papers related to robotic surgery in obstetrics and gynecology via the Web of Science Core Collection (WoSCC), which includes Science Citation Index Expanded, Social Sciences Citation Index, Arts & Humanities Citation Index, Conference Proceedings Citation Index—Science, Conference Proceedings Citation Index—Social Science & Humanities, Emerging Sources Citation Index, Current Chemical Reactions, and Index Chemicus. We accessed the Web of Science (WoS) by logging into the institutional account of Shandong University. The retrieval strategy was as follows: (Topic = robotic surgical procedure* OR robot surgery OR robotic surgery OR robot assisted surgery OR robotic assisted surgery OR robot enhanced surgery OR robotic enhanced surgery OR Aesop OR Zeus OR da Vinci). The WoS category was restricted to “obstetrics and gynecology”. The first robotic surgery on a human began in 1998 (27). Therefore, the time span of our study was set from January 1, 1998 to December 31, 2023. The publication type was limited to original articles and reviews. Two researchers first worked together to search the publications that met the requirements by reading the abstracts on October 31, 2022, and updated on January 18, 2024. Disagreements were settled by a third investigator. The detailed processes of inclusion and exclusion are displayed in Figure 1. This study did not require ethics committee approval. It was a retrospective bibliometric analysis of previously published articles.

### 2.2 Data export

Data from all selected papers, including authors, organizations, countries/regions, keywords, times cited, titles, publication years, source journals and corresponding impact factors, were exported and saved in Microsoft Excel 2019 and EndNote Desktop, respectively. The impact factor was defined in accordance with the Journal Citation Report (2022). These data were subsequently analyzed both qualitatively and quantitatively.

### 2.3 Visualization maps of data

VOSviewer is software that can be used for bibliometric visualization and analysis of literature (28). Visualization maps of authors, countries/regions and organizations based on co-authorship data, as well as keyword visualization maps based on co-occurrences can be constructed using this software (29, 30). Different nodes in the visualization map indicate different specific terms, such as keywords, authors, organizations, and countries/regions. The parameters and settings for using VOSviewer were as follows: Method = Association strength, Attraction = 2, Repulsion = 0, Resolution = 1, and Minimum cluster size = 1.

In network visualization maps of countries/regions, authors, and organizations based on co-authorship data, the size of the node indicates co-authorship frequency. A line between two countries/regions/authors/organizations indicates their collaboration. The line thickness between two nodes corresponds to the line strength (LS), which varied depending on the number of papers co-authored. Stronger collaboration is indicated by thicker lines. Countries/regions/authors/organizations with high levels of collaboration are depicted by nodes of the same color. The sum of all LS for a given term is the total link strength (TLS), which shows the collaboration strength between the term and other terms.

For visualization maps of keywords based on co-occurrence data, three different types of maps, network visualization map, density visualization map, and overlay visualization map, have their own meanings. In the network visualization map, the size of the node represents the corresponding frequency of occurrence. A larger node means that it appears more times, whereas a smaller node indicates that it appears fewer times. The keywords with the same color form a cluster, and each cluster represents a research hotspot (31). Through the network visualization map of keywords, we can identify the research hotspots represented by each cluster (31). In the density visualization map, the color of a keyword depends on its occurrence frequency. The red keywords appear most frequently, followed by the yellow, green and cyan keywords. With the density visualization map of keywords, we can determine the research focus in this field. In the overlay visualization map, different colors represent different years. The average publication year (APY) based on the average occurrence time of a keyword was used to evaluate the novelty of this keyword. Combined with the above information, we can understand the global research status and predict future research trends in this field.

## 3 Results

**Figure 2:**
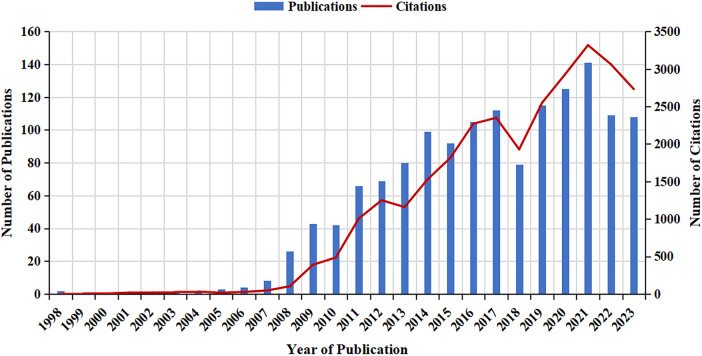
Number of papers published per year from 1998 to 2023.

A total of 1,430 papers were retrieved from the WoSCC using our specific search terms and restrictive conditions (Supplementary Data S1). Figure 2 shows the number of publications published per year from 1998 to 2023. In terms of publication type, the majority of the papers were original research articles (*n* = 1,249; 87.3%), while the rest were review articles (*n* = 181; 12.7%). Among the original research articles, there were 371 retrospective studies and 142 prospective studies. In addition, only 38 of the original research articles were randomized controlled trials (RCTs). For languages, there were 1,393 papers in English (97.4%).

### 3.1 Countries/regions

**Figure 3:**
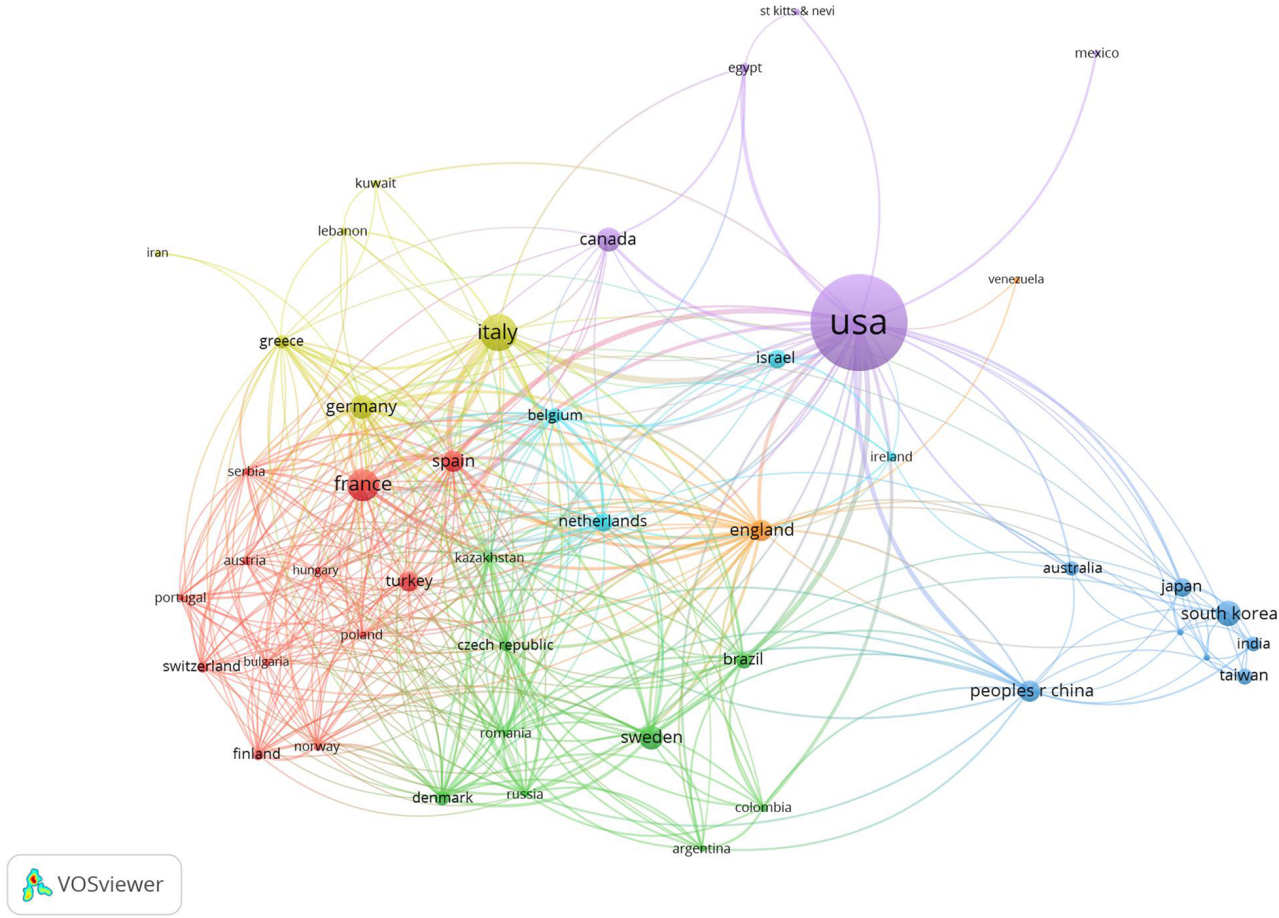
Network visualization map of countries/regions’ co-authorship analysis. The size of the node indicates co-authorship frequency. A line between two nodes indicates collaboration between two countries/regions. The line thickness between two nodes corresponds to the line strength, which varied depending on the number of papers co-authored. Stronger collaboration is indicated by thicker lines. Countries/regions with high levels of collaboration are depicted by nodes of the same color.

The papers originated from 69 countries/regions, and the top 10 countries/regions are listed in Table 1. The United States contributed the largest number of papers (*n* = 793), followed by Italy (*n* = 118) and France (*n* = 85). Moreover, the countries that published the most papers in the decade from 2014 to 2023 were the United States (*n* = 564), Italy (*n* = 96), and France (*n* = 65). Over the decade from 2004 to 2013, the same three countries produced the most papers, with 225 from the United States, 22 from Italy, and 20 from France. We constructed a network visualization map of countries/regions based on co-authorship data (Figure 3). After restricting the minimum number of publications for a country to two, 48 countries/regions were included. The United States had the highest level of cooperation (TLS = 149), followed by Italy (TLS = 102) and England (TLS = 90). The closest cooperation was between Italy and the United States (LS = 18), Brazil and the United States (LS = 13), and Italy and Spain (LS = 13).

**Table 1:** The top 10 authors/organizations/countries ranked by the number of papers.

Authors^a^			Organizations^a^			Countries/Regions^a^		
Name	Number of papers	Number of citations	Name	Number of papers	Number of citations	Name	Number of papers	Number of citations
Ramirez, PT	36	1,393	Mayo Clinic	85	1,953	USA	793	19,673
Scambia, G	35	716	University of Texas System	65	1,947	Italy	118	2,234
Magrina, JF	27	1,036	University of North Carolina	61	2,007	France	85	845
Persson, J	22	785	Cleveland Clinic Foundation	58	2,280	South Korea	55	694
Holloway, RW	21	902	Harvard University	52	1,254	Canada	50	1,336
Frumovitz, M	20	1,025	University of California System	47	1,048	Sweden	49	1,188
Falcone, T	20	924	Catholic University of The Sacred Heart	40	818	Germany	48	786
Ahmad, S	20	851	Columbia University	37	734	Spain	42	733
Zanagnolo, V	20	519	University System of Ohio	32	1,175	England	41	608
Fader, AN	19	1,251	Johns Hopkins University	30	1,068	Peoples R China	39	468

^a^ These three sub-tables in this table are not co-related.

### 3.2 Authors

**Figure 4:**
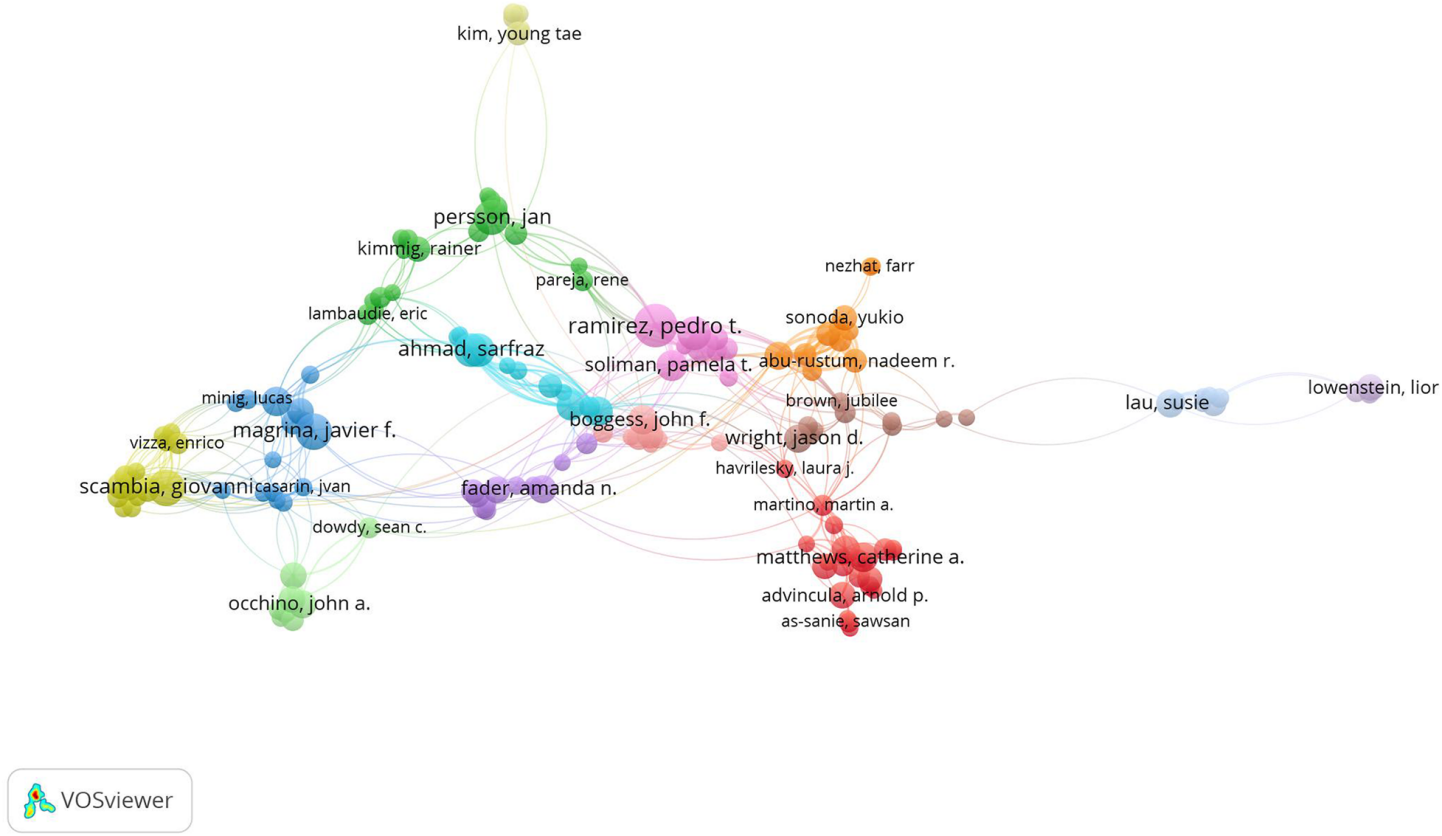
Network visualization map of authors’ co-authorship analysis. The size of the node indicates co-authorship frequency. A line between two nodes indicates collaboration between two authors. The line thickness between two nodes corresponds to the line strength, which varied depending on the number of papers co-authored. Stronger collaboration is indicated by thicker lines. Authors with high levels of collaboration are depicted by nodes of the same color.

The top 10 authors and organizations are also listed in Table 1. Professor Pedro T Ramirez contributed the most papers (*n* = 36), followed by Professor Giovanni Scambia (*n* = 35) and Professor Javier F Magrina (*n* = 27). A total of 185 papers published by the top 10 authors accounted for 12.9% of all studies in this field. Eight of the top 10 authors were from the United States, with one author from Italy and one author from Sweden. Based on the co-authorship data, a network visualization map of the authors' co-authorship was constructed as shown in Figure 4. For the purpose of author co-authorship analysis, the minimum number of papers for an author was set at five. A total of 189 authors met this threshold and were selected to be included in the co-authorship analysis. Professor Pedro T Ramirez (TLS = 113) was also the author with the highest total link strength, followed by Professor Giovanni Scambia (TLS = 89) and Professor Pamela T Soliman (TLS = 79). The closest collaboration was between Professor Sarfraz Ahmad and Professor Robert W Holloway (LS = 20).

### 3.3 Organizations

**Figure 5:**
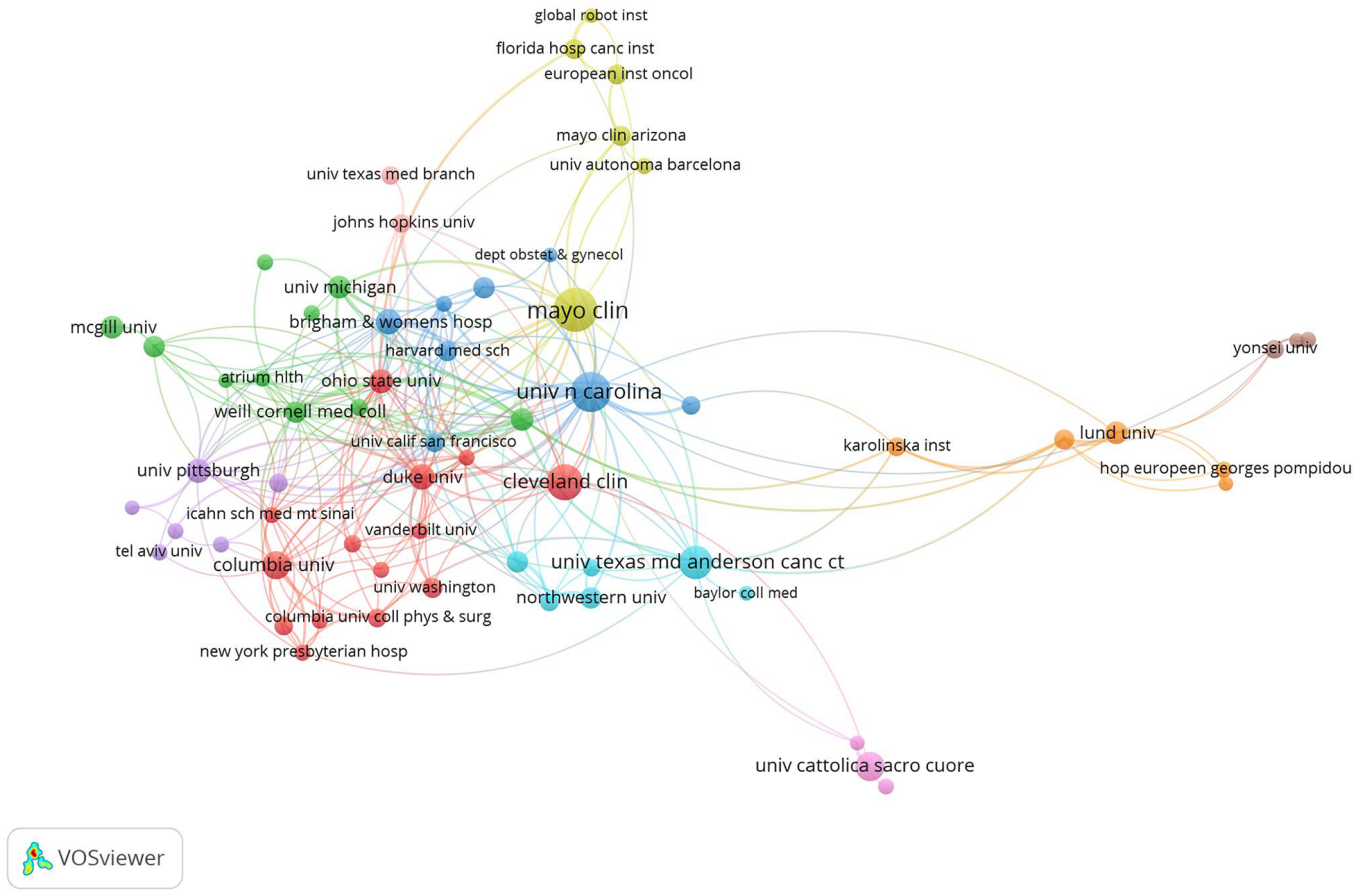
Network visualization map of organizations’ co-authorship analysis. The size of the node indicates co-authorship frequency. A line between two nodes indicates collaboration between two organizations. The line thickness between two nodes corresponds to the line strength, which varied depending on the number of papers co-authored. Stronger collaboration is indicated by thicker lines. Organizations with high levels of collaboration are depicted by nodes of the same color.

For organizations, the Mayo Clinic produced the most papers (*n* = 85), followed by the University of Texas System (*n* = 65) and the University of North Carolina (*n* = 61) (Table 1). Within the top 10 organizations in this field, eight were organizations in the United States, and two were Italian organizations. Similarly, we constructed a network visualization map of organizations based on the co-authorship data, as shown in Figure 5. For the purpose of organizations' co-authorship analysis, the minimum number of papers for an organization was set at eight. A total of 65 organizations met this threshold and were selected for inclusion in the co-authorship analysis. The organization with the highest total link strength was the University of North Carolina (TLS = 49), followed by Duke University (TLS = 43) and the Mayo Clinic (TLS = 37). The closest collaboration was between Skane University Hospital (Sweden) and Lund University (Sweden) (LS = 10).

### 3.4 Citations and journals

The number of citations for each of the 1,430 papers related to robotic surgery in obstetrics and gynecology ranged from 0 to 295, with a sum of 29,103. The average number of citations per paper was 20.35. Table 2 listed the top 10 most cited papers in this field (32–41), with the most cited paper published in 2011 by Professor Paraiso MFR. The average citation count for the top 10 papers was 210.20.

**Table 2:** The top 10 most cited papers on robotic surgery in obstetrics and gynecology.

Rank	Title of the paper	Number of citations	Authors	Publication year	OA^a^ status
1	Laparoscopic compared with robotic sacrocolpopexy for vaginal prolapse: a randomized controlled trial.	295	Paraiso et al. (32)	2011	non-OA
2	A case-control study of robot-assisted type III radical hysterectomy with pelvic lymph node dissection compared with open radical hysterectomy.	252	Boggess et al. (33)	2008	non-OA
3	Laparoendoscopic single-site surgery (LESS) in gynecologic oncology: Technique and initial report.	222	Fader et al. (34)	2009	non-OA
4	Detection of sentinel lymph nodes in minimally invasive surgery using indocyanine green and near-infrared fluorescence imaging for uterine and cervical malignancies.	212	Jewell et al. (35)	2014	OA
5	Short-term outcomes of robotic sacrocolpopexy compared with abdominal sacrocolpopexy.	206	Geller et al. (36)	2008	non-OA
6	What is the optimal minimally invasive surgical procedure for endometrial cancer staging in the obese and morbidly obese woman?	202	Gehrig et al. (37)	2008	non-OA
7	Minimally invasive comprehensive surgical staging for endometrial cancer: Robotics or laparoscopy?	192	Seamon et al. (38)	2009	non-OA
8	What is the learning curve for robotic assisted gynecologic surgery?	191	Lenihan et al. (39)	2008	non-OA
9	Robotic compared with laparoscopic sacrocolpopexy: a randomized controlled trial.	184	Anger et al. (40)	2014	OA
10	Robotic compared with conventional laparoscopic hysterectomy: a randomized controlled trial.	146	Sarlos et al. (41)	2012	non-OA

^a^ OA, open access.

A total of 1,430 papers were published in 74 journals. Among these journals, the *Journal of Minimally Invasive Gynecology* published the most papers (*n* = 252), followed by *Gynecologic Oncology* (*n* = 151) and the *International Urogynecology Journal* (*n* = 100). The top 10 journals ranked by the number of papers and their impact factors are listed in Table 3. Among these 10 journals, *Obstetrics and Gynecology* had the highest average number of citations per paper (*n* = 59.48), followed by *Gynecologic Oncology* (*n* = 44.86) and the *American Journal of Obstetrics and Gynecology* (*n* = 37.83)*.* Regarding the impact factors of the top 10 journals, the above three journals still had the highest impact factors, which were the *American Journal of Obstetrics and Gynecology*, *Obstetrics and Gynecology* and *Gynecologic Oncology* in descending order.

**Table 3:** The top 10 journals ranked by the number of papers.

Journals	Number of papers	Number of citations	Average number of citations per paper	IF^a^ (2022)	IF^a^ (last five years)	Citescore (2022)
Journal of minimally invasive gynecology	252	5,015	19.90	4.1	3.8	4.4
Gynecologic oncology	151	6,774	44.86	4.7	5.0	8.4
International urogynecology journal	100	1,058	10.58	1.8	2.2	3.4
International journal of gynecological cancer	84	1,810	21.55	4.8	4.0	6.7
Female pelvic medicine and reconstructive surgery	83	1,116	13.45	1.6	1.7	N/A
American journal of obstetrics and gynecology	65	2,459	37.83	9.8	9.1	14.3
Obstetrics and gynecology	52	3,093	59.48	7.2	7.6	9.8
Archives of gynecology and obstetrics	45	691	15.36	2.6	2.7	4.5
European journal of obstetrics & gynecology and reproductive biology	44	678	15.41	2.6	2.6	4.7
Best practice & research clinical obstetrics & gynaecology	35	242	6.91	5.5	6.5	7.6

^a^ Impact factor (IF) was defined according to the Journal Citation Report (2022).

### 3.5 Co-occurrence analysis of keywords

**Figure 6:**
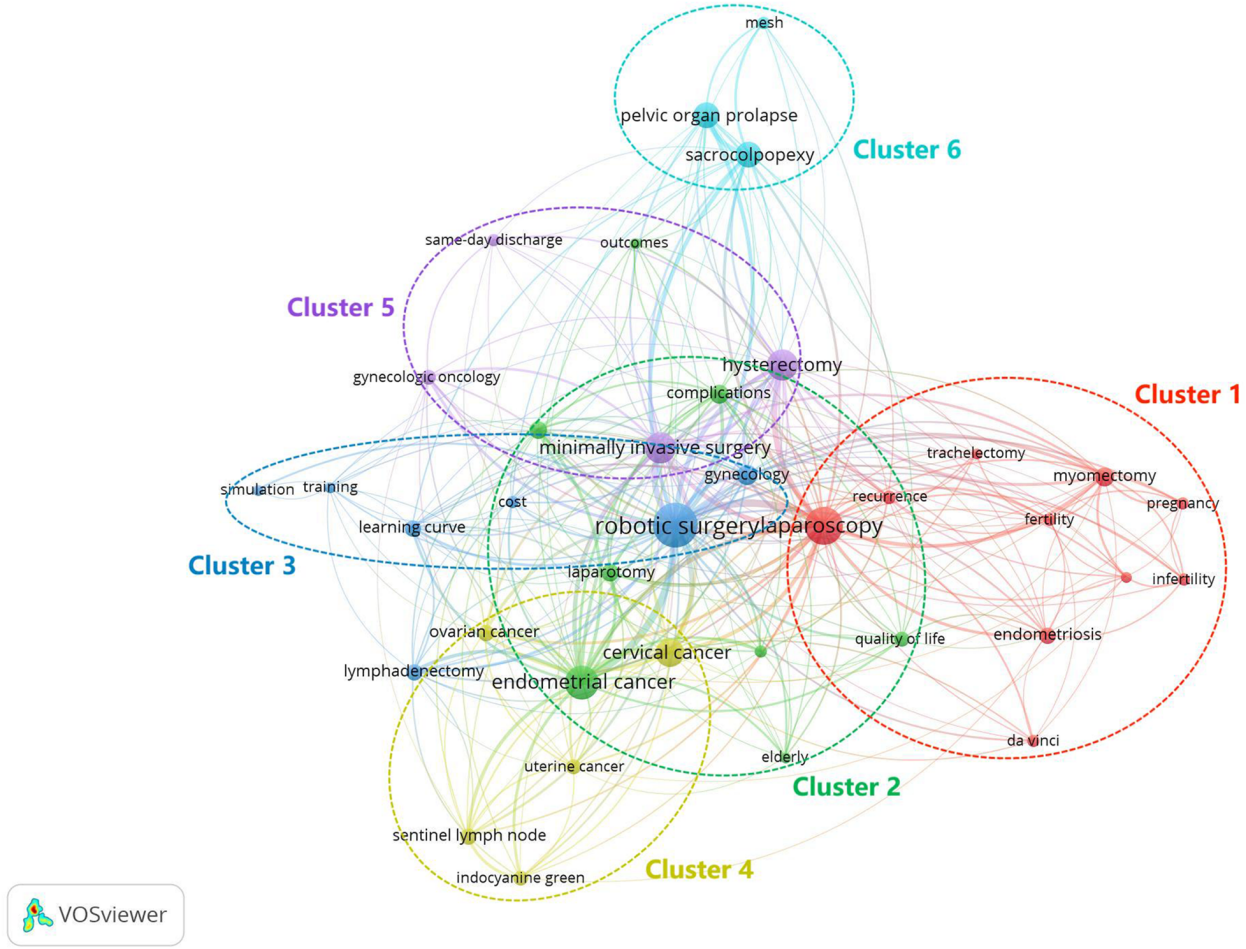
Network visualization map of keyword co-occurrence analysis conducted by VOSviewer. The size of a node indicates the frequency of keyword occurrence, and keywords are classified into six clusters: application of robotic surgery in gynecologic benign diseases (cluster 1), surgical outcomes of robotic surgery for endometrial cancer (cluster 2), cost and learning curve of robotic surgery for gynecologic diseases (cluster 3), robotic surgery for sentinel lymph node detection in gynecologic malignancies (cluster 4), robotic surgery for gynecologic oncology (cluster 5), and robotic sacrocolpopexy for pelvic organ prolapse (cluster 6).

VOSviewer identified a total of 2,050 keywords from 1,430 papers based on co-occurrence data. After restricting the minimum number of keyword occurrences to 12, a total of 66 items were included. We manually unified and standardized the keywords and finally identified 37 keywords. We then constructed a network visualization map using these keywords, which were classified into six clusters (Figure 6). The research hotspots were identified according to the keywords contained in each cluster, as shown in Table 4. Cluster 1 was the largest cluster in this study, and prominent keywords in this cluster were endometriosis, fertility, fibroids, infertility, myomectomy, pregnancy, recurrence and trachelectomy. For cluster 2, the main keywords were endometrial cancer, complications, outcomes, quality of life and survival. The primary keywords in cluster 3 were cost, learning curve, simulation and training. In cluster 4, the dominant keywords were cervical cancer, indocyanine green, ovarian cancer, sentinel lymph node and uterine cancer. Cluster 5 consisted of keywords such as gynecologic oncology, hysterectomy and same-day discharge. The keywords mesh, pelvic organ prolapse and sacrocolpopexy were frequently used in cluster 6.

**Table 4:** The clusters formed by keyword co-occurrence analysis.

Clusters	Research hotspots	Number of keywords	Main keywords
Cluster 1	Application of robotic surgery in gynecologic benign diseases	10	Endometriosis; fertility; fibroids; infertility; myomectomy; pregnancy; recurrence; trachelectomy
Cluster 2	Surgical outcomes of robotic surgery for endometrial cancer	8	Endometrial cancer; complications; outcomes; quality of life; survival
Cluster 3	Cost and learning curve of robotic surgery for gynecologic diseases	7	Cost; learning curve; simulation; training
Cluster 4	Robotic surgery for sentinel lymph node detection in gynecologic malignancies	5	Cervical cancer; indocyanine green; ovarian cancer; sentinel lymph node; uterine cancer
Cluster 5	Robotic surgery for gynecologic oncology	4	Gynecologic oncology; hysterectomy; same-day discharge
Cluster 6	Robotic sacrocolpopexy for pelvic organ prolapse	3	Mesh; pelvic organ prolapse; sacrocolpopexy

**Figure 7:**
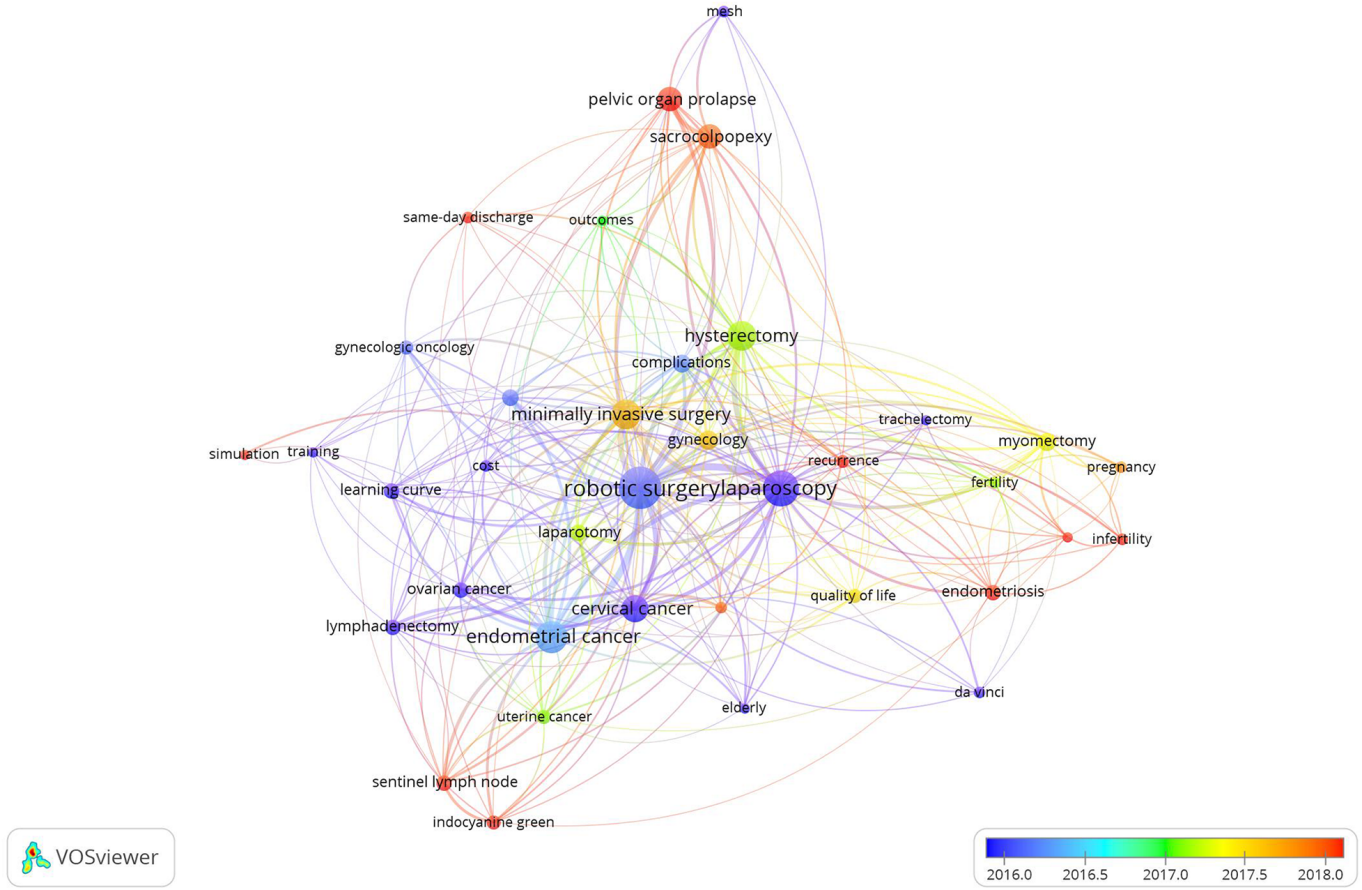
Overlay visualization map of keyword co-occurrence analysis conducted by VOSviewer. Keywords are imparted by using different colors based on the APY.

Along with the network visualization map of co-occurrence terms, an overlay visualization map was constructed in which keywords were imparted by using different colors based on the APY (Figure 7). The purple color indicates keywords appearing relatively early in the time course, while the red color reflects recent occurrences. This overlay visualization map showed that cluster 1, cluster 4 and cluster 6 had more main keywords with the largest APY, including sentinel lymph node, indocyanine green, endometriosis, recurrence, fibroids, infertility, sacrocolpopexy and pelvic organ prolapse. Furthermore, some main keywords in other clusters, such as same-day discharge, simulation and survival, also had a relatively large APY. This indicated that the topics related to these keywords had recently received increasing attention.

**Figure 8:**
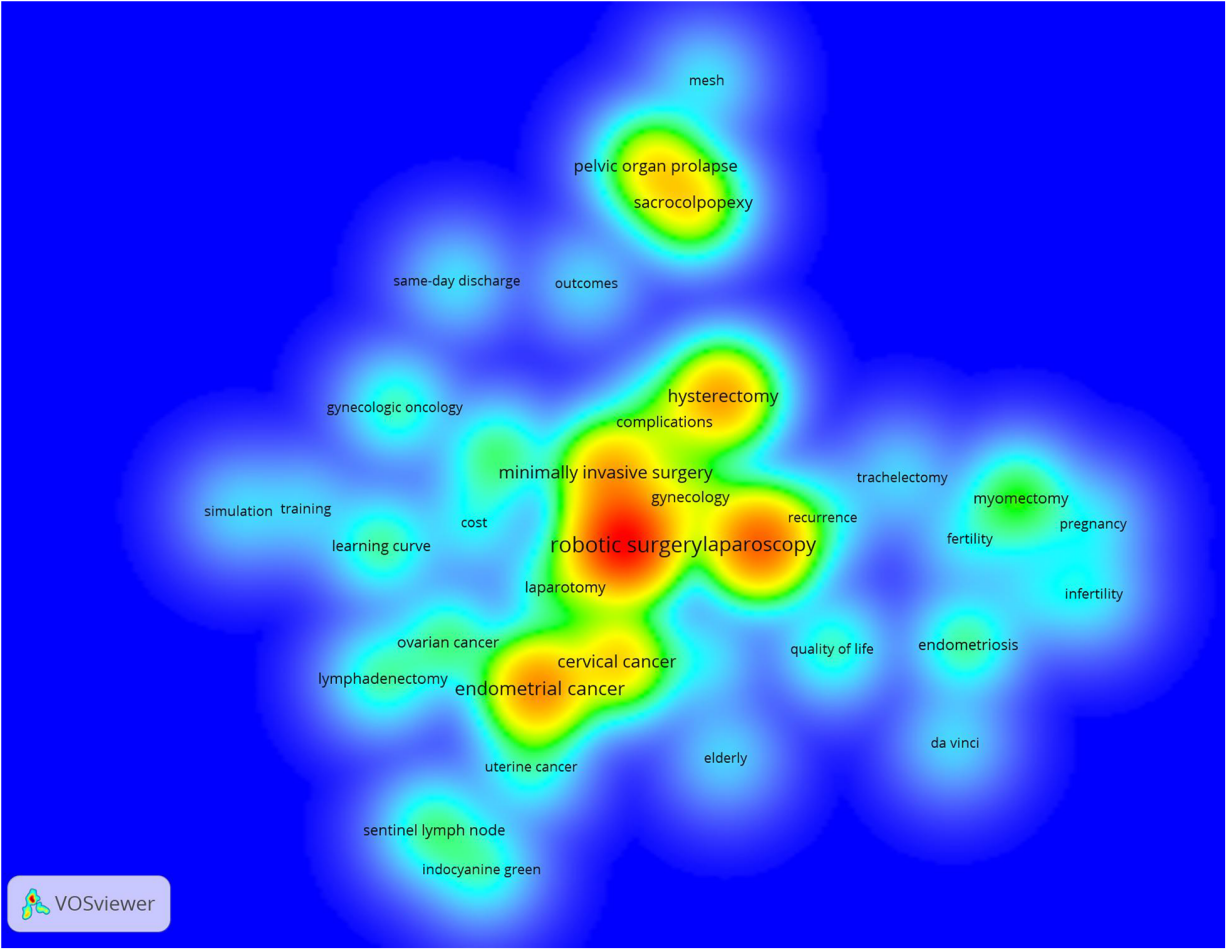
Density visualization map of keyword co-occurrence analysis conducted by VOSviewer. The color of a keyword depends on its occurrence frequency. The red keywords appear most frequently, followed by the yellow, green and cyan keywords.

A density visualization map of the keywords according to their occurrence frequency was also constructed, as shown in Figure 8. The main keywords included endometrial cancer (occurrences: 185), hysterectomy (occurrences: 159), cervical cancer (occurrences: 116), sacrocolpopexy (occurrences: 96) and pelvic organ prolapse (occurrences: 94), which appeared the most frequently.

## 4 Discussion

### 4.1 Principal results

This is a VOSviewer-based bibliometric analysis to identify papers on robotic surgery in obstetrics and gynecology as well as to analyze their bibliometric characteristics. In this study, we used the WOSCC to find 1,430 relevant papers from 1998 to 2023. The number of papers in this research field increased from year to year on the whole, peaking in 2021 with 141 published papers, except for a slight decrease in 2018, 2022 and 2023, which may be attributed to incomplete inclusion in the WoSCC in 2023. Especially since the last decade, robotic surgery in obstetrics and gynecology has stepped into a period of rapid development, with the number of publications increasing at a much faster rate than that in the previous decade. The results indicate that research in this field is attracting increasing attention from researchers.

According to the network visualization map of countries/regions based on co-authorship data, we can conclude that the distribution of related research on robotic surgery in obstetrics and gynecology is imbalanced, although research in this field has attracted the attention of many countries around the world. The economic environment plays an important role in the level of research and development (42). Correspondingly, the majority of countries in this research field are European countries with high economic levels. Our results showed that approximately half of the papers were published in the United States, reflecting the dominance of the United States in this field. This situation has also been observed in bibliometric analysis in other fields, such as endometrial carcinoma (43) and robotic surgery research in urology (44). This may be due to the high level of funding for academic activities and the long history of research on robotic surgery in the United States. In addition, the United States has the closest cooperation with other countries in this research field, whereas many other countries have research partnerships with only a handful of countries. Therefore, cooperation between countries should be further strengthened.

With respect to the authors, Professor Ramirez PT, from the University of Texas MD Anderson Cancer Center, ranked first in the number of papers published, with 36 papers related to robotic surgery in obstetrics and gynecology. Almost all of the top 10 authors with the most contributions stemmed from the top 10 organizations. Of all the organizations, the Mayo Clinic had the highest number of papers of any organization worldwide, with 85 relevant papers identified, accounting for 5.9% of all papers in this field. The majority of the top 10 organizations with the most contributions were from the United States. In addition, cooperation among authors and organizations was noted. Professor Ramirez PT was the author with the broadest connections to other scientists, and the University of North Carolina was the institution with the most partnerships with other institutions in this field. The network visualization maps of co-authorship analysis reveal that collaboration among authors was limited to small groups, and collaborative links between research organizations were also lacking. This phenomenon suggests that scientists and institutions from around the world should push their boundaries to bring about deep collaboration. Only then can we promote rapid development in this field for the benefit of patients.

Our results showed that the average number of citations for the top 10 papers on robotic surgery in obstetrics and gynecology was approximately ten times that of the average for all papers. The number of citations can be viewed as a direct measure of the recognition a paper has received in its field of study (45). For papers, the number of citations can be related to a number of factors, such as the year of publication and accessibility. In terms of the year of publication, even the most cited papers were not cited when they were originally published, and older papers may have more citations due to cumulative effects (46). We find that the majority of the top 10 most cited papers were published approximately in 2010. Therefore, the time factor should be taken into account when evaluating the impact of a paper through citation analysis (47). In terms of accessibility, open access (OA) means that anyone can have free and unrestricted online access to scientific journal literature (48). It has been shown that OA journals have higher citation metrics than non-OA journals (49).

By collecting journal information, we identified 74 journals that published papers in the field of robotic surgery in obstetrics and gynecology. The *Journal of Minimally Invasive Gynecology* topped the list with 252 papers, which accounted for approximately one-fifth of the total number of papers published in all journals. In the top 10 journals ranked by the number of papers, the *American Journal of Obstetrics and Gynecology* had the highest impact factor, and *Obstetrics and Gynecology* had the highest average number of citations per paper. In general, *Gynecologic Oncology*, the *American Journal of Obstetrics and Gynecology*, and *Obstetrics and Gynecology* were journals with the highest overall comprehensive levels in this research field, both in terms of impact factor and average number of citations per paper. It is worth noting that the second- and fourth-ranked journals were related to gynecologic cancer research. This result reflects the fact that scholars have focused on the application of robotic surgery in gynecologic malignancies.

Keywords in the network visualization map were divided into six clusters. Cluster 1 was related to the applications of robotic surgery in gynecologic benign diseases, mainly including endometriosis, uterine fibroids and infertility. A study based on one of the largest published samples assessed the perioperative outcomes of robotic-assisted laparoscopic surgery for the treatment of deep infiltrating endometriosis (DIE) (50). The researchers in this study did not observe an increase in bleeding or intra-operative or post-operative complications. They concluded that laparoscopic surgery for DIE may require multidisciplinary surgical teams to perform complex surgical procedures, and DIE may be one of the most promising indications for robot-assisted laparoscopic surgery. Cluster 2 reflected the surgical outcomes of robotic surgery for endometrial cancer with the keywords: quality of life, outcomes, survival, and complications. The quality of life is a very important aspect of reporting outcomes. Among 1,430 papers on robotic surgery research in obstetrics and gynecology in this research, we identified 64 papers on the quality of life. Kurt G et al.' paper named “Comparison of health-related quality of life of women undergoing robotic surgery, laparoscopic surgery or laparotomy for gynecologic conditions: A cross-sectional study” demonstrated that women in the robotic group had better quality of life than that in laparoscopic or laparotomy group after gynecologic surgery (51).

Cluster 3 was associated with cost and learning curve of robotic surgery for gynecologic diseases. Professor Lenihan JP's paper titled “What is the learning curve for robotic assisted gynecologic surgery?” was published in *Journal of Minimally Invasive Gynecology* in 2008 (39). This study showed that a surgeon with advanced laparoscopic skills needs 50 cases to stabilize operating times for the various procedures in women requiring benign gynecologic interventions. The authors predicted that the constant development of instruments suitable for gynecology and computer-based surgical simulators by the da Vinci System development team, as well as the standardization of general surgical protocols by inter-institutional robotic surgeons, will have significant benefits in shortening the learning curve process. Cluster 4 focused on robotic surgery for sentinel lymph node detection in gynecologic malignancies. The paper titled “Detection of sentinel lymph nodes in minimally invasive surgery using indocyanine green and near-infrared fluorescence imaging for uterine and cervical malignancies” published in *Gynecologic Oncology* in 2014 by Professor Jewell EL et al. was cited 212 times (35). The results of this study suggested that near-infrared fluorescence imaging with indocyanine green intracervical injection using a robotic platform had a high detection rate of bilateral sentinel lymph nodes and appeared to favor the use of blue dye alone or other modalities. The use of blue dye in combination with indocyanine green appears unnecessary.

Cluster 5 focused on robotic surgery for gynecologic oncology. A paper titled “Robotic radical hysterectomy in early stage cervical cancer: A systematic review and meta-analysis” was published in *Gynecologic Oncology* in 2015 (52). This study found that robotic radical hysterectomy may be superior to abdominal radical hysterectomy with lower estimated blood loss, fewer wound-related complications, and shorter hospital stays. Robotic radical hysterectomy and laparoscopic radical hysterectomy appeared to be equivalent in terms of intraoperative and postoperative short-term outcomes, so that the choice of procedure can be based on the choice of surgeon and patient. Cluster 6 was mainly related to robotic sacrocolpopexy for pelvic organ prolapse. The most frequently cited paper on robotic surgery in obstetrics and gynecology was published in *Obstetrics and Gynecology* by Professor Paraiso MFR et al. in 2011 (32). The objective of this research was to compare the efficacy of laparoscopic sacrocolpopexy vs. robotic sacrocolpopexy in the treatment of patients with post-hysterectomy vaginal prolapse. It was concluded that compared to the conventional laparoscopic approach, robotic sacrocolpopexy was associated with additional costs, increased post-operative pain, and longer procedures without improvement in any of the clinical outcome measures at perioperative, 6-month, or 1-year follow-up. A similar conclusion was reached in a subsequently published randomized controlled trial with a high number of citations in the research field (40).

Through the analysis of the network visualization and density visualization maps generated by the keywords, we concluded that although robotic surgery has been applied to many diseases in the field of gynecology, the main research topics of scholars were gynecologic malignant tumors. In the study of malignant tumors, endometrial cancer and cervical cancer have always been the focus. In addition, researchers typically compared the outcomes of robotic surgery for a given disease with other surgical approaches to assess the feasibility and safety of robotic surgery. While many studies have demonstrated the advantages of robotic surgery in the treatment of diseases in obstetrics and gynecology, the control groups in these studies have mostly been retrospective cohorts in which the surgery was performed over different time periods. In contrast, in one RCT, researchers compare one or more treatment groups to a control group, and randomly assign patients to either the treatment or control group. RCTs are considered the highest evidence for establishing causality in clinical studies, and this process of randomization minimizes differences in group characteristics that could affect outcomes (53). Moreover, there are still many questions regarding the cost and training of robotic surgery in obstetrics and gynecology, and the data from existing studies are still very limited with only 46 papers on cost-effectiveness analysis and 56 papers on training or learning analysis among 1,430 papers in this field. Overall, rigorous scientific research and long-term data are necessary to determine the appropriate use of robotics in obstetrics and gynecology (54).

Combined with the analysis of the overlay visualization map, we found that the keywords sacrocolpopexy and pelvic organ prolapse have attracted attention in recent years, and we also found that these two keywords have a high frequency of occurrence. This means that the study of robotic sacrocolpopexy for pelvic organ prolapse has become a new research hotspot. Other main keywords that have appeared in recent years include sentinel lymph node, indocyanine green, fibroids, infertility, endometriosis, recurrence, same-day discharge, simulation, and survival, and their frequency of occurrence was not high. Some of the topics related to the above keywords may become new research hotspots in the future. Robotic surgery for sentinel lymph node detection in gynecologic malignancies are more promising topics because there was a cluster of keywords related to this research direction in the network visualization map.

### 4.2 Strengths, limitations and recommendations for future research

In contrast to traditional literature review, this bibliometric analysis analyzed the papers on robotic surgery in obstetrics and gynecology with the help of VOSviewer to understand the global research status and predict future research trends in the field. This study can also help researchers identify influential authors, organizations, and journals in this field. Scholars interested in the field of robotic surgery in obstetrics and gynecology can conduct academic activities or seek collaboration with relevant scholars or institutions. At the same time, our results can guide researchers in this field to submit their manuscripts to appropriate journals. However, there were still several limitations to this study.

First, the bibliometric analysis was based on the WoSCC, so relevant papers from other databases were omitted. Second, while we had broadened the search terms as much as possible, there may still have been omissions. Third, the citation analysis did not exclude the effects of self-citation, citations of lectures or conferences, and the potential preference of authors to cite specific journal papers (55, 56). Some bibliometric indicators, such as the Eigenfactor score, can help to avoid the bias caused by journal self-citation by removing citations from one paper in a journal to another paper in the same journal (25). Fourth, we may have missed some valuable papers that had recently been published. Fifth, there may be classification errors by WOSCC when indexing the articles. Sixth, there may be other publications that were not categorized as “obstetrics and gynecology”.

Future works should involve more detailed methodology for a bibliometric review of the field to bridge some of the limitations of this study. Researchers should increase research on the application of robotic surgery for gynecologic benign diseases. Moreover, researchers need to conduct more RCTs and design more prospective studies. The study of the cost and training of robotic surgery should also be of concern to researchers.

## 5 Conclusions

To our knowledge, this is the first VOSviewer-based bibliometric analysis of robotic surgery research in obstetrics and gynecology. The United States was the leading country, and the *Journal of Minimally Invasive Gynecology* was the most productive journal in the field. Scientists and institutions from around the world should push their boundaries to bring about deep collaboration. The main research topic has always been the use of robotic surgery in the treatment of gynecologic malignancies. More randomized controlled trials need to be conducted to compare surgical outcomes of robotic surgery with other surgical approaches. Robotic sacrocolpopexy for pelvic organ prolapse has become a new research hotspot, and robotic surgery for sentinel lymph node detection in gynecologic malignancies are more potential directions for future research.

In summary, this study illustrates research hotspots and trends on robotic surgery in obstetrics and gynecology using the VOSviewer-based bibliometric analysis method. At the same time, this study identifies the most prolific researchers and institutions in this field, which helps scholars to find suitable scientific research collaborators and lay the foundation for international cooperative research in this field.

## Data Availability

The original contributions presented in the study are included in the article/**Supplementary Material**, further inquiries can be directed to the corresponding author.
