# Supplementary material for: Global research hotspots and trends on robotic surgery in obstetrics and gynecology: a bibliometric analysis based on VOSviewer
Source: Front Surg. 2024 Feb 9;11:1308489. doi: 10.3389/fsurg.2024.1308489 (PMC10884115; doi:10.3389/fsurg.2024.1308489)
Supplement: Supplementary file 1 [file Datasheet1.docx]

Supplementary Material

Global research hotspots and trends on robotic surgery in obstetrics and gynecology: A bibliometric analysis based on VOSviewer

Peichen Xiao, Lu Li, Jinfeng Qu, Guangxin Wang*

*** Correspondence:** Guangxin Wang: wanggxchina@sdu.edu.cn

**Supplementary Data S1.** Data of 1 430 papers related to robotic surgery in obstetrics and gynecology.

FN Clarivate Analytics Web of Science

VR 1.0

PT J

AU Marty, L

Myrick, O

AF Marty, Lindsay

Myrick, Olivia

TI Discrepancy in Gynecologic Case Volumes and Surgical Participation of

Obstetrics/Gynecology Residents

SO JOURNAL OF GYNECOLOGIC SURGERY

LA English

DT Article; Early Access

DE surgical education; surgical training; surgical residency; resident

education; obstetrics and gynecology education; obstetrics and

gynecology residency

AB Objective: Gynecology residents have self-reported deficits in preparation for surgical practice, particularly in robotic training. The primary source of surgical training is active participation in an operating room, which can be documented in a resident's case log. Educators and trainees may assume an institution's case volume reflects residents' participation, but there is no standard way to hold attending physicians accountable for surgical education, case-by-case. This study examined the percent of major gynecologic cases that allowed active trainee participation over 3 months at a major academic medical center.Materials and Methods: A baseline assessment of obstetrics/gynecology residents' participation in gynecologic surgical cases was conducted over 3 months at a large, urban academic medical center. All open and robotic gynecologic cases were recorded by the residency education team. Trainees were asked to document if a resident was present in a surgical case and that resident's level of participation in the procedure.Results: Of 324 open and robotic gynecologic surgeries, 74% were covered by residents. Of the 240 cases in which residents participated, 71% could be entered into clinical case logs, and 29% permitted minimal to no active participation. Thus, residents were only able to log active participation in 53% of all open and robotic gynecologic cases in the 3-month timeframe.Conclusions: Operative case volume at an academic medical center does not necessarily reflect resident participation and surgical training experience; there is a need to incorporate surgical-teaching skills into faculty-development programs and to emphasize the importance of active participation, case by case, in residency training further. (J GYNECOL SURG 20XX:000)

C1 [Marty, Lindsay] NYU, Grossman Sch Med, New York, NY USA.

[Myrick, Olivia] NYU Langone Hlth, Dept Obstet Gynecol, New York, NY USA.

[Marty, Lindsay] NYU, Grossman Sch Med, 550 1st Ave, New York, NY 10016 USA.

C3 New York University; NYU Langone Medical Center; New York University

RP Marty, L (corresponding author), NYU, Grossman Sch Med, 550 1st Ave, New York, NY 10016 USA.

EM Lindsay.Marty@nyulangone.org

NR 11

TC 0

Z9 0

U1 0

U2 0

PU MARY ANN LIEBERT, INC

PI NEW ROCHELLE

PA 140 HUGUENOT STREET, 3RD FL, NEW ROCHELLE, NY 10801 USA

SN 1042-4067

EI 1557-7724

J9 J GYNECOL SURG

JI J. Gynecol. Surg.

PD 2023 DEC 12

PY 2023

DI 10.1089/gyn.2023.0107

EA DEC 2023

PG 4

WC Obstetrics & Gynecology; Surgery

WE Emerging Sources Citation Index (ESCI)

SC Obstetrics & Gynecology; Surgery

GA AQ9G9

UT WOS:001120038500001

DA 2024-01-18

ER

PT J

AU Johannesson, U

Amato, M

Forsgren, C

AF Johannesson, Ulrika

Amato, Martina

Forsgren, Catharina

TI Pelvic floor and sexual function 3 years after hysterectomy - A

prospective cohort study

SO ACTA OBSTETRICIA ET GYNECOLOGICA SCANDINAVICA

LA English

DT Article; Early Access

DE abdominal hysterectomy; female sexual function; laparoscopic

hysterectomy; minimally invasive surgery; pelvic floor function; robotic

assisted laparoscopic hysterectomy; urinary incontinence

ID FUNCTION INDEX FSFI; LAPAROSCOPIC HYSTERECTOMIES; ORGAN PROLAPSE; WOMEN;

DISORDERS; PREVALENCE; VALIDATION; TRENDS

AB Introduction: Long term effects after hysterectomy, such as a worsening of pelvic floor and sexual function, have been studied with diverse results. Therefore, we investigated the long-term effects of hysterectomy for benign indication on pelvic floor and sexual function as well as differences in outcome depending on mode of hysterectomy.Material and methods: In a prospective clinical cohort study, we included 260 women scheduled for hysterectomy who answered validated questionnaires; pelvic floor impact questionnaire (PFIQ-7), pelvic floor distress inventory (PFDI-20) and female sexual function index (FSFI). Participants were followed up to 3 years after surgery. Nonparametric statistics and mixed effect models were used in analyses of the data.Results: After exclusions, 242 women remained in the study, with a response rate at the 3-year follow-up of 154/242 (63.6%) for all questionnaires. There was an improvement of pelvic floor function with a mean score of PFIQ-7 at baseline of 42.5 (SD 51.7) and at 3 years 22.7 (SD 49.4), (p < 0.001) and mean score of PFDI-20 at baseline was 69.6 (SD 51.1) and at 3 years 56.2 (SD 54.6), (p = 0.001). A deterioration of sexual function was seen among the sexually active women after 3 years with a mean score of FSFI at baseline 25.2 (SD 6.6) and after 3 years 21.6 (SD 10.1), (p < 0.001). However, this was not consistent with the unaltered sexual function for the whole cohort. No difference in pelvic floor or sexual function was detected when comparing robotic assisted laparoscopic hysterectomy, laparoscopic hysterectomy and abdominal hysterectomy.Conclusions: Three years after surgery robotic assisted laparoscopic hysterectomy, total laparoscopic hysterectomy and abdominal hysterectomy improve pelvic floor function to the same extent. Among the sexually active women, a decline of sexual function was seen after 3 years, not consistent with the entire cohort and independent of surgical methods. Whether this is a trend associated with aging or menopausal transition remains to be studied.

C1 [Johannesson, Ulrika; Amato, Martina; Forsgren, Catharina] Karolinska Inst, Danderyd Hosp, Dept Clin Sci, Stockholm, Sweden.

[Johannesson, Ulrika; Amato, Martina; Forsgren, Catharina] Danderyd Hosp, Dept Obstet & Gynecol, Stockholm, Sweden.

[Johannesson, Ulrika] Danderyd Hosp, Dept Obstet & Gynecol, SE-18288 Stockholm, Sweden.

C3 Danderyds Hospital; Karolinska Institutet; Danderyds Hospital; Danderyds

Hospital

RP Johannesson, U (corresponding author), Danderyd Hosp, Dept Obstet & Gynecol, SE-18288 Stockholm, Sweden.

EM ulrika.johannesson@regionstockholm.se

FU Intuitive Surgical

FX Intuitive Surgical

NR 33

TC 0

Z9 0

U1 0

U2 0

PU WILEY

PI HOBOKEN

PA 111 RIVER ST, HOBOKEN 07030-5774, NJ USA

SN 0001-6349

EI 1600-0412

J9 ACTA OBSTET GYN SCAN

JI Acta Obstet. Gynecol. Scand.

PD 2023 DEC 10

PY 2023

DI 10.1111/aogs.14751

EA DEC 2023

PG 10

WC Obstetrics & Gynecology

WE Science Citation Index Expanded (SCI-EXPANDED)

SC Obstetrics & Gynecology

GA AM1V6

UT WOS:001118800000001

PM 38071460

OA hybrid

DA 2024-01-18

ER

PT J

AU Monterossi, G

Anchora, LP

Oliva, R

Fagotti, A

Fanfani, F

Costantini, B

Naldini, A

Giannarelli, D

Scambia, G

AF Monterossi, G.

Anchora, L. Pedone

Oliva, R.

Fagotti, A.

Fanfani, F.

Costantini, B.

Naldini, A.

Giannarelli, D.

Scambia, G.

TI The new surgical robot HugoTM RAS for total hysterectomy: a pilot study

SO FACTS VIEWS AND VISION IN OBGYN

LA English

DT Article

DE Robotic surgery; hysterectomy; surgical outcomes; malfunction; safety;

Hugo

ID TELELAP ALF-X; LAPAROSCOPIC HYSTERECTOMY; SYSTEM; FEASIBILITY;

MALFUNCTION; SURGERY

AB Background: With the rising popularity of robotic surgery, HugoTM RAS is one of the newest surgical robotic platforms. Investigating the reliability of this tool is the first step toward validating its use in clinical practice; and presently there arelimited data available regarding this. The literature is constantly enriched with initial experiences, however no study has demonstrated the safety of this platform yet. Objectives: This study aimed to investigate its reliability during total hysterectomy. Materials and Methods: A series of 20 consecutive patients scheduled for minimally invasive total hysterectomy with or without salpingo-oophorectomy for benign disease or prophylactic surgery were selected to undergo surgery with HugoTM RAS. Data regarding any malfunction or breakdown of the robotic system as well as intraand post-operative complications were prospectively recorded. Results: Fifteen of the twenty patients (75.0%) underwent surgery for benign uterine diseases, and five (25.0%) underwent prophylactic surgery. Among the entire series, an instrument fault occurred in one case (5.0%). The problem was solved in 4.8 minutes and without complications for the patient. The median total operative time was 127 min (range, 98-255 min). The median estimated blood loss was 50 mL (range:30-125 mL). No intraoperative complications were observed. One patient (5.0%) developed Clavien-Dindo grade 2 postoperative complication. Conclusions: In this pilot study, HugoTM RAS showed high reliability, similar to other robotic devices. What is new? Present findings suggest that HugoTM RAS is a viable option for major surgical procedures and deserves further investigation in clinical practice.

C1 [Monterossi, G.; Anchora, L. Pedone; Oliva, R.; Fagotti, A.; Fanfani, F.; Costantini, B.; Naldini, A.; Scambia, G.] Fdn Policlin Univ A Gemelli IRCCS, Dipartimento Salute Donna & Bambino & Salute Publ, Rome, Italy.

[Oliva, R.; Fagotti, A.; Fanfani, F.; Scambia, G.] Univ Cattolica Sacro Cuore, Rome, Italy.

[Giannarelli, D.] Fdn Policlin Univ A Gemelli IRCCS, Facil Epidemiol & Biostat, Rome, Italy.

[Anchora, L. Pedone] Largo Agostino Gemelli 8, I-00168 Rome, Italy.

C3 Catholic University of the Sacred Heart; IRCCS Policlinico Gemelli;

Catholic University of the Sacred Heart; IRCCS Policlinico Gemelli;

Catholic University of the Sacred Heart; IRCCS Policlinico Gemelli

RP Anchora, LP (corresponding author), Largo Agostino Gemelli 8, I-00168 Rome, Italy.

EM luigi.us@hotmail.it

NR 31

TC 0

Z9 0

U1 0

U2 0

PU UNIVERSA PRESS

PI WETTEREN

PA RUE HOENDER 24, WETTEREN, 9230, BELGIUM

SN 2032-0418

J9 FACTS VIEWS VIS OBGY

JI Facts Views Vis. ObGyn

PD DEC

PY 2023

VL 15

IS 4

BP 331

EP 337

DI 10.52054/FVVO.15.4.11

PG 7

WC Obstetrics & Gynecology

WE Emerging Sources Citation Index (ESCI)

SC Obstetrics & Gynecology

GA CQ5N1

UT WOS:001126723300002

PM 38128091

OA Bronze

DA 2024-01-18

ER

PT J

AU Pavone, M

Goglia, M

Campolo, F

Scambia, G

Ianieri, MM

AF Pavone, M.

Goglia, M.

Campolo, F.

Scambia, G.

Ianieri, M. M.

TI En-block butterfly excision of posterior compartment deep endometriosis:

The first experience with the new surgical robot

SO FACTS VIEWS AND VISION IN OBGYN

LA English

DT Article

DE Endometriosis; robotic surgery; docking; minimally invasive surgery.

ID INFILTRATING ENDOMETRIOSIS

AB Background: Minimally invasive surgery is the gold standard treatment for deep endometriosis when medical management fails. In selected cases, such as when bowel or urinary tract are involved, robotic assisted surgery can be useful due to its characteristics of high dexterity and manoeuvrability. This is the first case of robotic en-bloc excision of posterior compartment deep endometriosis performed with the new HugoTM RAS system. Objective: The purpose of this video article is to show for the first time the feasibility of bowel surgery for deep endometriosis with this new robotic device. Materials and methods: A 24-years-old woman affected by severe dysmenorrhea, chronic pelvic pain, dyschezia and dyspareunia underwent to deep endometriosis excision using the new robotic platform HugoTM RAS system at the Unit of Gynaecological Oncology, Fondazione Policlinico Universitario A. Gemelli IRCCS, Rome, Italy. Main outcome measures: Intraoperative data, docking set up, post-operative outcomes up to three months follow up were evaluated. Results: The surgical procedure was carried out without in tra-operative or post-operative complications, operative time (OT) was 200 minutes, while docking time was 8 minutes. No system errors or faults in the robotic arms were registered. Post-operative complete disease-related symptoms relief was reported. Conclusion: According to our results, the introduction of this new robotic platform in the surgical management of deep endometriosis seems to be feasible, especially in advanced cases. However, further studies are needed to demonstrate the benefits of this surgical system and the advantages of robotic surgery compared to laparoscopy in this subset of patients.

C1 [Pavone, M.; Campolo, F.; Scambia, G.; Ianieri, M. M.] Fdn Policlin Univ A Gemelli, Dipartimento Sci Salute Donna & Bambino & Sanita P, IRCCS, UOC Ginecol Oncol, Rome, Italy.

[Pavone, M.; Goglia, M.] Univ Strasbourg, Univ Hosp Inst IHU, Inst Chirurg Guidee image, Strasbourg, France.

[Pavone, M.; Goglia, M.] Res Inst Digest Canc IRCAD France, IRCAD, Strasbourg, France.

[Goglia, M.] Sapienza Univ Rome, St Andrea Univ Hosp, Dept Med Sci & Translat Med, Rome, Italy.

[Ianieri, M. M.] Mater Olbia Hosp, Gynaecol & Breast Care Ctr, Olbia, Italy.

[Pavone, M.] Fdn Policlin Univ A Gemelli IRCCS, Dipartimento Salute Donna & Bambino & Salute Pubbl, Largo Agostino Gemelli 8, I-00168 Rome, Italy.

C3 Catholic University of the Sacred Heart; IRCCS Policlinico Gemelli;

Universites de Strasbourg Etablissements Associes; Universite de

Strasbourg; Sapienza University Rome; Azienda Ospedaliera Sant'Andrea;

Catholic University of the Sacred Heart; IRCCS Policlinico Gemelli

RP Pavone, M (corresponding author), Fdn Policlin Univ A Gemelli IRCCS, Dipartimento Salute Donna & Bambino & Salute Pubbl, Largo Agostino Gemelli 8, I-00168 Rome, Italy.

EM matteopavone.21@gmail.com

RI ianieri, manuel maria/HCI-5794-2022

OI ianieri, manuel maria/0000-0001-8383-1982

FU ANR [ANR-10-IAHU-02]; French state funds

FX This work was supported by French state funds managed within the "Plan

Investissements d'Avenir" and by the ANR (reference ANR-10-IAHU-02)

NR 16

TC 0

Z9 0

U1 0

U2 0

PU UNIVERSA PRESS

PI WETTEREN

PA RUE HOENDER 24, WETTEREN, 9230, BELGIUM

SN 2032-0418

J9 FACTS VIEWS VIS OBGY

JI Facts Views Vis. ObGyn

PD DEC

PY 2023

VL 15

IS 4

BP 359

EP 362

DI 10.52054/FVVO.14.5.104

PG 4

WC Obstetrics & Gynecology

WE Emerging Sources Citation Index (ESCI)

SC Obstetrics & Gynecology

GA CQ5N1

UT WOS:001126723300008

PM 38128095

OA Bronze

DA 2024-01-18

ER

PT J

AU Kurup, M

Bidarahalli, S

Jayaram, S

AF Kurup, Mayadevi

Bidarahalli, Suguna

Jayaram, Surya

TI Robotic Surgery in Gynaecology: A Retrospective Evaluation of an

Experience at a Single Centre

SO JOURNAL OF OBSTETRICS AND GYNECOLOGY OF INDIA

LA English

DT Article; Early Access

DE Hysterectomy; Laparoscopy; Robotic; Myomectomy; Cystectomy

ID ENDOMETRIOSIS; HYSTERECTOMY; MYOMECTOMY; DIAGNOSIS

AB ObjectiveThe aim of this research was to assess the role of robotics and its outcome in gynaecology both in benign and malignant cases in a single centre and provide a critical evaluation of possible advantages of robot assisted surgeries from surgeons' point of view.DesignA single centre, retrospective observational study.PopulationAll women who underwent robotic gynaecological surgeries between 2015 and 2022.MethodsThe Da Vinci Si (TM) robotic system was used for these surgeries performed by all surgeons at our quaternary care centre, and data were acquired retrospectively through electronic medical records. Descriptive statistical analysis of data was done. Main outcome measures included operative time, estimated blood loss, hospital stay, complications and conversion rates in all cases. Age was analysed as a demographic data.OutcomeA total of 211 robotic cases were performed including 172 hysterectomies, 20 myomectomies and 19 cases for other gynaecological indications. The mean operating time or hysterectomy and myomectomy was 113 and 129 min, respectively, and haemoglobin drop was 1.34 and 1.2 g/dl, respectively. No conversions to laparotomy were observed in either of the groups. The surgeries for 19 benign gynaecological conditions included ovarian cystectomy, cesarean scar repair and chronic cornual ectopic.ConclusionRobotic surgical system helps accomplish several procedures with exceptional laparoscopic skills. Robotic surgery is safe in all types of gynaecological procedures and is a promising alternative for comprehensive gynaecologic surgical care.

C1 [Kurup, Mayadevi; Bidarahalli, Suguna] Aster Medcity, Dept Obstet & Gynaecol, Kochi, Kerala, India.

[Jayaram, Surya] Med Trust Hosp, Dept Obstet & Gynaecol, Kochi, Kerala, India.

RP Bidarahalli, S (corresponding author), Aster Medcity, Dept Obstet & Gynaecol, Kochi, Kerala, India.

EM drmayadevi.kurup@asterhospital.com; suguna_gmcite@yahoo.co.in;

suryaj1@yahoo.co.in

NR 14

TC 0

Z9 0

U1 1

U2 1

PU SPRINGER INDIA

PI NEW DELHI

PA 7TH FLOOR, VIJAYA BUILDING, 17, BARAKHAMBA ROAD, NEW DELHI, 110 001,

INDIA

SN 0971-9202

EI 0975-6434

J9 J OBSTET GYN INDIA

JI J. Obstet. Gynecol. India

PD 2023 NOV 30

PY 2023

DI 10.1007/s13224-023-01852-x

EA NOV 2023

PG 7

WC Obstetrics & Gynecology

WE Emerging Sources Citation Index (ESCI)

SC Obstetrics & Gynecology

GA Z3JH7

UT WOS:001111063900001

DA 2024-01-18

ER

PT J

AU Olsen, RG

Hartwell, D

Dalsgaard, T

Madsen, ME

Bjerrum, F

Konge, L

Roder, A

AF Olsen, Rikke Groth

Hartwell, Dorthe

Dalsgaard, Torur

Madsen, Mette Elkjaer

Bjerrum, Flemming

Konge, Lars

Roder, Andreas

TI First experience with the Hugo™ robot-assisted surgery system for

endometriosis: A descriptive study

SO ACTA OBSTETRICIA ET GYNECOLOGICA SCANDINAVICA

LA English

DT Article; Early Access

DE endometriosis; gynecological surgery; Hugo (TM) RAS; IDEAL framework;

robotic surgical procedures

ID PERIOPERATIVE OUTCOMES; CLASSIFICATION; COMPLICATIONS; COHORT

AB Introduction: The Medtronic Hugo (TM) Robot-assisted Surgery (RAS) system was recently approved for clinical use. We explored the safety and feasibility of this system for endometriosis surgery. The primary outcome was safe case completion without major surgical complications (Clavien-Dindo grade <= 2) and no conversion to open surgery or laparoscopy.Material and methods: Surgeries for endometriosis performed at the Department of Gynecology, Rigshospitalet, on the Medtronic Hugo (TM) RAS system were included. Two experienced robotic surgeons performed all surgeries with their usual robotic team. The variables included were patient demographics, peri- and postoperative data, complications and 30-day readmission rate. We used the IDEAL framework 1/2a for surgical innovation in this descriptive study.Results: The first 12 patients were included. All cases were completed without intraoperative complications or conversion. Four patients experienced Clavien-Dindo grade 1 postoperative complications. No patients were re-admitted within 30 days. Median docking time (17 minutes), console time (87.5 minutes), blood loss (40 mL) and length of hospital stay (1 day) were acceptable compared with previous literature.Conclusions: In this pilot study, we found the Medtronic Hugo (TM) RAS system safe and feasible for robot-assisted surgery for endometriosis. The advent of new robotic systems is welcomed to accelerate the development of technology that will advance surgical care for patients across the globe.

C1 [Olsen, Rikke Groth; Bjerrum, Flemming; Konge, Lars] Copenhagen Acad Med Educ & Simulat CAMES, Copenhagen, Denmark.

[Olsen, Rikke Groth; Roder, Andreas] Copenhagen Univ Hosp, Rigshosp, Copenhagen Prostate Canc Ctr, Dept Urol, Copenhagen, Denmark.

[Olsen, Rikke Groth; Hartwell, Dorthe; Dalsgaard, Torur; Madsen, Mette Elkjaer] Copenhagen Univ Hosp, Rigshosp, Dept Gynecol, Copenhagen, Denmark.

[Madsen, Mette Elkjaer; Konge, Lars; Roder, Andreas] Univ Copenhagen, Fac Hlth & Med Sci, Copenhagen, Denmark.

[Bjerrum, Flemming] Herlev Gentofte Hosp, Dept Surg, Herlev, Denmark.

[Olsen, Rikke Groth] Copenhagen Acad Med Educ & Simulat CAMES, Ryesgade 53B, DK-2100 Copenhagen, Denmark.

C3 Rigshospitalet; University of Copenhagen; Rigshospitalet; University of

Copenhagen; University of Copenhagen

RP Olsen, RG (corresponding author), Copenhagen Acad Med Educ & Simulat CAMES, Ryesgade 53B, DK-2100 Copenhagen, Denmark.

EM qtn295@sund.ku.dk

OI Roder, Andreas/0000-0002-0019-5333

NR 37

TC 0

Z9 0

U1 0

U2 0

PU WILEY

PI HOBOKEN

PA 111 RIVER ST, HOBOKEN 07030-5774, NJ USA

SN 0001-6349

EI 1600-0412

J9 ACTA OBSTET GYN SCAN

JI Acta Obstet. Gynecol. Scand.

PD 2023 NOV 29

PY 2023

DI 10.1111/aogs.14727

EA NOV 2023

PG 10

WC Obstetrics & Gynecology

WE Science Citation Index Expanded (SCI-EXPANDED)

SC Obstetrics & Gynecology

GA Z3GI8

UT WOS:001110987000001

PM 38031442

OA hybrid

DA 2024-01-18

ER

PT J

AU Hebert, T

AF Hebert, T.

TI Robotic assisted laparoscopy for deep infiltrating endometriosis

SO BEST PRACTICE & RESEARCH CLINICAL OBSTETRICS & GYNAECOLOGY

LA English

DT Article

DE Robotic surgery; Endometriosis; Laparoscopy; Fertility; Colorectal

surgery; Urinary tract; Diaphragmatic endometriosis; Pelvic nerves

ID NERVE-SPARING SURGERY; INDOCYANINE GREEN; DIAPHRAGMATIC ENDOMETRIOSIS;

COLORECTAL RESECTION; SURGICAL-MANAGEMENT; CLINICAL-USE; BLADDER;

VISUALIZATION; FEASIBILITY; PREVALENCE

AB Deep infiltrative endometriosis is a condition affecting up to 15 % of women of childbearing age, defined by extra uterine location of endometrial like tissues. The symptoms of endometriosis range from severe dysmenorrhea to infertility, chronic pelvic pain, bowel dysfunction and urinary tract involvement to name the most common. Endometriosis has an impact on the quality of life of patients, with personal and social consequences. Although medical treatment is indicated in the first instance, surgery may be necessary. Standard laparoscopy has become the gold standard for this surgery. However, surgery for deep infiltrative endometriosis is known to be highly complex, and the significant development of robotic assistance in recent years has had an impact on the evolution of surgical practice. This comprehensive review of the literature provides an overview of the contributions of robotic surgery in the field of endometriosis and gives an insight into the next steps in its development.

C1 [Hebert, T.] Univ Hosp, Ctr Olympe Gouges, Gynecol Surg Dept, Tours, France.

C3 CHU Tours

RP Hebert, T (corresponding author), Ctr Olympe Gouges, Gynaecol Dept, 2 Bd Tonnelle, F-37044 Tours, France.

EM thebert2@yahoo.com

NR 78

TC 0

Z9 0

U1 1

U2 1

PU ELSEVIER SCI LTD

PI London

PA 125 London Wall, London, ENGLAND

SN 1521-6934

EI 1532-1932

J9 BEST PRACT RES CL OB

JI Best Pract. Res. Clin. Obstet. Gynaecol.

PD FEB

PY 2024

VL 92

AR 102422

DI 10.1016/j.bpobgyn.2023.102422

EA NOV 2023

PG 9

WC Obstetrics & Gynecology

WE Science Citation Index Expanded (SCI-EXPANDED)

SC Obstetrics & Gynecology

GA CO5T7

UT WOS:001126209200001

PM 38007964

DA 2024-01-18

ER

PT J

AU Russo, ML

Gallant, T

King, CR

AF Russo, Miguel Luna

Gallant, Thomas

King, Cara R.

TI Surgical techniques for mini-laparotomy myomectomy

SO FERTILITY AND STERILITY

LA English

DT Article

DE Myomectomy; fertility sparing; leiomyoma; fibroid; minimally invasive

surgery

AB Context and background: The prevalence of uterine fibroids is estimated to be approximately 80%. Fibroids can be associated with abnormal uterine bleeding, pressure symptoms, and infertility. Given this high prevalence, approximately 30,000 myomectomies are performed in the United States per year. Minimally invasive approaches are preferred, if feasible. The minimally invasive techniques include laparoscopic, robot-assisted, hysteroscopic, and mini-laparotomy.Objective: To discuss the multiple techniques for optimizing the use of mini-laparotomy in minimally invasive myomectomy.Design: We use intraoperative surgical video to demonstrate techniques that optimize the use of the mini-laparotomy for myomectomy.Setting: Cleveland Clinic.Patient(s): Patient's undergoing fertility preserving, minimally invasive myomectomy at the Cleveland Clinic. The patient(s) included in this video gave consent for publication of the video and posting of the video online, including social media, the journal website, sci-entific literature websites (such as PubMed, ScienceDirect, and Scopus), and other applicable sites.Intervention(s): After the surgeon has selected to proceed with mini-laparotomy myomectomy, different techniques can be employed to optimize management. We demonstrate and discuss these techniques to ensure that surgeons have a set of tools to tackle a fibroid uterus. These techniques include direct palpation of the fibroids, use of a uterine manipulator to visualize the endometrial cavity, use of the uterine manipulator to aid in repair of the cavity if entered, suturing technique that avoids the endometrial cavity and therefore limits foreign body exposure and decreases intrauterine adhesion formation, utilization of barbed suture in a layered fashion, in-situ debulking to avoid injury to fallopian tubes and other critical uterine structures, easy identification of the optimal enucleation plane, use of single hysterotomy for multiple fibroids, visualization of the "Tortuga"sign, and evaluation of the abdominal cavity using the mini-laparotomy site as a port site. To limit postoperative adhesion formation, the investigators place cellulose-based adhesion barriers with peritoneum closure. Although the need for prolonged postoperative observation can be made on a case-by -case basis, we consider this as an outpatient surgery and anticipate same-day discharge for our patients.Main outcome measure(s): In this video, we perform a mini-laparotomy myomectomy optimally and describe the techniques employed.Result(s): Specific techniques employed in mini-laparotomy myomectomy make the case safe, effective, and can lead to same-day discharge.Conclusion(s): Mini-laparotomy myomectomy is a technique used to perform minimally invasive myomectomy. Following the discussed steps, surgeons can be more confident in performing this method of myomectomy. (Fertil Steril (R) 2023;120:1262-3. (c) 2023 by American Society for Reproductive Medicine.) Use your smartphone to scan this code

C1 [Russo, Miguel Luna; Gallant, Thomas; King, Cara R.] Cleveland Clin, Obstet Gynecol & Womens Hlth Inst, Cleveland Hts, OH USA.

[Gallant, Thomas] Cleveland Clin, Anesthesiol Inst, Mail Code A81,9500 Euclid Ave, Cleveland Hts, OH 44195 USA.

C3 Cleveland Clinic Foundation; Cleveland Clinic Foundation

RP Gallant, T (corresponding author), Cleveland Clin, Anesthesiol Inst, Mail Code A81,9500 Euclid Ave, Cleveland Hts, OH 44195 USA.

EM Gallant@ccf.org

OI Luna Russo, Miguel/0000-0002-0230-3510

NR 4

TC 1

Z9 1

U1 0

U2 0

PU ELSEVIER SCIENCE INC

PI NEW YORK

PA STE 800, 230 PARK AVE, NEW YORK, NY 10169 USA

SN 0015-0282

EI 1556-5653

J9 FERTIL STERIL

JI Fertil. Steril.

PD DEC

PY 2023

VL 120

IS 6

BP 1262

EP 1263

DI 10.1016/j.fertnstert.2023.08.973

EA NOV 2023

PG 2

WC Obstetrics & Gynecology; Reproductive Biology

WE Science Citation Index Expanded (SCI-EXPANDED)

SC Obstetrics & Gynecology; Reproductive Biology

GA DA1J4

UT WOS:001129218700001

PM 37690734

OA Bronze

DA 2024-01-18

ER

PT J

AU Lim, YH

Dagher, C

Abu-Rustum, NR

Mueller, JJ

Sonoda, Y

Zivanovic, O

Broach, V

Leitao, MM Jr

AF Lim, Yu Hui

Dagher, Christian

Abu-Rustum, Nadeem R.

Mueller, Jennifer J.

Sonoda, Yukio

Zivanovic, Oliver

Broach, Vance

Leitao Jr, Mario M.

TI Oncologic outcomes of robot-assisted laparoscopy versus conventional

laparoscopy for the treatment of apparent early-stage endometrioid

adenocarcinoma of the uterus

SO GYNECOLOGIC ONCOLOGY

LA English

DT Article

DE Endometrial cancer; Outcomes; Laparoscopy; Minimally invasive surgery;

Robotic surgery

ID HYSTERECTOMY; CANCER; SURGERY; SURVIVAL

AB <bold>Objective: </bold>To compare long-term oncologic outcomes in patients with clinically uterine-confined endometrioid endometrial cancer who underwent surgical staging with robot-assisted (RA) versus conventional laparoscopy.<bold>Methods: </bold>We performed a retrospective chart review of patients with newly diagnosed, uterine-confined endometrioid endometrial cancer who were treated and had primary surgery at our institution between 1/1/2009-1/1/2018. Clinicopathologic, surgical, and survival data were collected. Appropriate statistical methods were applied.<bold>Results: </bold>Of 1728 patients identified, 1389 (80.4%) underwent RA and 339 (19.6%) conventional laparoscopy. At diagnosis, median age was 60 years (range, 24-92) and median BMI was 30.2 kg/m(2) (range, 15.1-71.5). In the RA group, patients had longer operative time (170 vs 152 min, P < .001), lower conversion rate to laparotomy (0.6% vs 4.7%, P < .001), and a higher proportion had a BMI > 40 kg/m(2) (17.2% vs 11.5%, P = .01) and same-day discharge (19.2% vs 5.3%, P < .001). Overall, 93% (RA) and 90% (conventional) of patients underwent lymph node assessment (P = .1). Comparing the RA versus conventional groups, final surgical stage on pathology (P = .6), median follow-up (55.7 vs 52.9 months, P = .4), and rates of perioperative complications (9.9% vs 7.7%, P = .6), recurrence (9.5% vs 7.4%, P = .3), 5-year PFS (88.5% vs 91.0%, P = .3), and 5-year OS (92.5% vs 92.4%, P = .7) were not significantly different. No significant increase in risk of recurrence (HR = 1.2, 95% CI: 0.8-1.9, P = .3) or poorer OS outcomes (HR = 0.9, 95% CI: 0.6-1.4, P = .7) were observed in the RA group.<bold>Conclusion: </bold>In uterine-confined endometrioid endometrial cancers, surgical staging using RA laparoscopy was not associated with adverse survival outcomes compared to conventional laparoscopy.

C1 [Lim, Yu Hui; Dagher, Christian; Abu-Rustum, Nadeem R.; Mueller, Jennifer J.; Sonoda, Yukio; Zivanovic, Oliver; Broach, Vance; Leitao Jr, Mario M.] Mem Sloan Kettering Canc Ctr, Dept Surg, Gynecol Serv, New York, NY USA.

[Abu-Rustum, Nadeem R.; Mueller, Jennifer J.; Sonoda, Yukio; Zivanovic, Oliver; Broach, Vance; Leitao Jr, Mario M.] Weill Cornell Med Coll, Dept OB GYN, New York, NY 10021 USA.

[Leitao Jr, Mario M.] Mem Sloan Kettering Canc Ctr, Dept Surg, Gynecol Serv, 1275 York Ave, New York, NY 10065 USA.

C3 Memorial Sloan Kettering Cancer Center; Cornell University; Weill

Cornell Medicine; Memorial Sloan Kettering Cancer Center

RP Leitao, MM Jr (corresponding author), Mem Sloan Kettering Canc Ctr, Dept Surg, Gynecol Serv, 1275 York Ave, New York, NY 10065 USA.

FU NIH/NCI Cancer Center Support Grant [P30 CA008748]

FX This research was funded in part by the NIH/NCI Cancer Center Support

Grant P30 CA008748.

NR 19

TC 0

Z9 0

U1 0

U2 0

PU ACADEMIC PRESS INC ELSEVIER SCIENCE

PI SAN DIEGO

PA 525 B ST, STE 1900, SAN DIEGO, CA 92101-4495 USA

SN 0090-8258

EI 1095-6859

J9 GYNECOL ONCOL

JI Gynecol. Oncol.

PD DEC

PY 2023

VL 179

BP 152

EP 157

DI 10.1016/j.ygyno.2023.11.009

EA NOV 2023

PG 6

WC Oncology; Obstetrics & Gynecology

WE Science Citation Index Expanded (SCI-EXPANDED)

SC Oncology; Obstetrics & Gynecology

GA CE6Y6

UT WOS:001123623700001

PM 37980770

DA 2024-01-18

ER

PT J

AU Collins, A

Jacob, A

Moss, E

AF Collins, Anna

Jacob, Annie

Moss, Esther

TI Robotic-assisted surgery in high-risk surgical patients with endometrial

cancer

SO BEST PRACTICE & RESEARCH CLINICAL OBSTETRICS & GYNAECOLOGY

LA English

DT Article

DE Robotic-assisted hysterectomy; Laparoscopic hysterectomy; Frailty;

Obesity; Prehabilitation

ID OBESE-PATIENTS; FRAILTY; MANAGEMENT; SURVIVAL; IMPACT; PREHABILITATION;

HYSTERECTOMY; LAPAROSCOPY; LAPAROTOMY; ANESTHESIA

AB Many patients diagnosed with an endometrial cancer are at high-risk for surgery due to factors such as advanced age, raised body mass index or frailty. Minimally-invasive surgery, in particular robotic-assisted, is increasingly used in the surgical management of endometrial cancer however, there are a lack of clinical trials investigating outcomes in high-risk patient populations. This article will review the current evidence and identify areas of uncertainty where future research is needed.

C1 [Collins, Anna; Moss, Esther] Univ Leicester, Coll Life Sci, Univ Rd, Leicester LE1 7RH, England.

[Collins, Anna; Moss, Esther] Univ Hosp Leicester NHS Trust, Dept Gynaecol Oncol, Leicester LE1 5WW, England.

[Jacob, Annie] Univ Hosp Leicester NHS Trust, Dept Anaesthesia, Leicester LE1 5WW, England.

C3 University of Leicester; University Hospitals of Leicester NHS Trust;

University of Leicester; University of Leicester; University Hospitals

of Leicester NHS Trust

RP Moss, E (corresponding author), Univ Leicester, Coll Life Sci, Univ Rd, Leicester LE1 7RH, England.

EM em321@le.ac.uk

OI Moss, Esther/0000-0002-2650-0172

FU National Institute for Health and Care Research (NIHR) Leicester

Biomedical Research Centre (BRC)

FX No funding was obtained for this study. This research was carried out at

the National Institute for Health and Care Research (NIHR) Leicester

Biomedical Research Centre (BRC) .

NR 76

TC 0

Z9 0

U1 0

U2 0

PU ELSEVIER SCI LTD

PI London

PA 125 London Wall, London, ENGLAND

SN 1521-6934

EI 1532-1932

J9 BEST PRACT RES CL OB

JI Best Pract. Res. Clin. Obstet. Gynaecol.

PD FEB

PY 2024

VL 92

AR 102421

DI 10.1016/j.bpobgyn.2023.102421

EA NOV 2023

PG 7

WC Obstetrics & Gynecology

WE Science Citation Index Expanded (SCI-EXPANDED)

SC Obstetrics & Gynecology

GA AF5M8

UT WOS:001117064000001

PM 37980868

OA hybrid

DA 2024-01-18

ER

PT J

AU Dueñas-Garcia, OF

Shah, TS

Fritts, L

Leung, K

Alrayyes, N

Garcia, K

Flynn, M

Shapiro, R

Vallejo, M

AF Duenas-Garcia, Omar F.

Shah, Twisha

Fritts, Lexi

Leung, Katherine

Alrayyes, Nasser

Garcia, Katerina

Flynn, Michael

Shapiro, Robert

Vallejo, Manuel

TI The effect of sugammadex on postoperative urinary retention

post-laparoscopic and robotic hysterectomy with and without concomitant

procedures

SO INTERNATIONAL UROGYNECOLOGY JOURNAL

LA English

DT Article; Early Access

DE Postoperative urinary retention; Sugammadex; Glycopyrrolate;

Neostigmine; Hysterectomy

AB Introduction and hypothesis This study was aimed at determining the effect of sugammadex versus a combination of glycopyrrolate and neostigmine (GN) for neuromuscular reversal blockage on transient postoperative urinary retention (TPOUR) in patients undergoing a laparoscopic and robot-assisted laparoscopic hysterectomy.

Methods We conducted a retrospective cohort study in patients undergoing a laparoscopic or robotic hysterectomy between February 2017 and December 2021. Patients with and without concomitant procedures were included. Demographics and perioperative data were extracted from the patient's medical record. Before discharge, all patients were required to spontaneously void and have a post-void residual of less than 150 ml.

Results We identified 500 patients and 485 were included in the final analysis. We had 319 subjects who received sugammadex and 166 GN combination. Both groups had overall similar demographics and perioperative characteristics. Most patients had a conventional laparoscopy procedure (391 [82.5%]) compared with robotic (83 [17.5%]). Patients who received GN were significantly more likely to be discharged home with an indwelling catheter (odds ratio [OR], 1.82; 95% confidence interval [CI], 1.09-3.05). After adjusting for perioperative medications and sling implantation during the surgery a logistic regression model continued to demonstrate that patients who received GN had significantly higher odds of being discharged with a catheter (OR, 1.79; 95% CI, 1.03-3.12).

Conclusions Our findings suggest that sugammadex decreases the odds of TPOUR after laparoscopic hysterectomies with and without slings compared with the combination of GN. Additional prospective trials are required to confirm this finding.

C1 [Duenas-Garcia, Omar F.; Shapiro, Robert] West Virginia Univ, Obstet & Gynecol Dept, 1 Med Ctr Dr, Morgantown, WV 26508 USA.

[Shah, Twisha; Fritts, Lexi; Alrayyes, Nasser; Garcia, Katerina] West Virginia Univ, Med Sch, Morgantown, WV USA.

[Leung, Katherine; Flynn, Michael] Univ Massachusetts, Obstet & Gynecol Dept, Worcester, MA USA.

[Vallejo, Manuel] West Virginia Univ, Dept Anesthesia & Med Educ, Morgantown, WV USA.

C3 West Virginia University; West Virginia University; University of

Massachusetts System; University of Massachusetts Worcester; West

Virginia University

RP Dueñas-Garcia, OF (corresponding author), West Virginia Univ, Obstet & Gynecol Dept, 1 Med Ctr Dr, Morgantown, WV 26508 USA.

EM omar.duenasgarcia@hsc.wvu.edu

NR 21

TC 0

Z9 0

U1 0

U2 0

PU SPRINGER LONDON LTD

PI LONDON

PA 236 GRAYS INN RD, 6TH FLOOR, LONDON WC1X 8HL, ENGLAND

SN 0937-3462

EI 1433-3023

J9 INT UROGYNECOL J

JI Int. Urogynecol. J.

PD 2023 NOV 6

PY 2023

DI 10.1007/s00192-023-05668-1

EA NOV 2023

PG 7

WC Obstetrics & Gynecology; Urology & Nephrology

WE Science Citation Index Expanded (SCI-EXPANDED)

SC Obstetrics & Gynecology; Urology & Nephrology

GA X2RB3

UT WOS:001096968100001

PM 37930427

DA 2024-01-18

ER

PT J

AU Stewart, C

Moreno, A

Vo, E

Bhattarai, B

Farley, J

Willmott, L

Monk, BJ

Chase, DM

AF Stewart, Chelsea

Moreno, Andrea

Vo, Elise

Bhattarai, Bikash

Farley, John

Willmott, Lyndsay

Monk, Bradley J.

Chase, Dana M.

TI Factors Associated with Inpatient Narcotic Medication Usage after

Robotic-assisted Laparoscopy

SO JOURNAL OF MINIMALLY INVASIVE GYNECOLOGY

LA English

DT Article

DE Robotic surgery; Narcotic medication; Gynecologic oncology

ID PAIN; HYSTERECTOMY

AB Study Objective: Describe factors that contribute to an increased narcotic medication use after robotic-assisted laparoscopic (RAL) surgery.Design: A retrospective cohort.Setting: A teaching hospital.Patients: All patients undergoing RAL surgery by gynecologist oncologists at St. Joseph's Hospital and Medical Center over a 3-year period.Interventions: RAL by gynecologist oncologists.Measurements and Main Results: Using retrospective chart review, patients who underwent RAL surgery from 2012 to 2015 in the division of gynecologic oncology were identified; 757 patients were eligible for inclusion in the study. Total narcotic use during the postoperative hospital stay was converted to oral morphine milligram equivalents (OME). Bivariate correlations of total OME narcotics to multiple variables were evaluated using Spearman's rho. The average age, body mass index, and length of stay were 53.76 years (17-92), 31.75 kg/m(2) (17-56), and 1.56 days (range, 0-19), respectively. Increased OME correlated positively with body mass index (Spearman's rho = .077, p = .036), any intraoperative complication (Spearman's rho = .05, p = .886), any postoperative complication (Spearman's rho = .16, p <.0001), length of stay in days (Spearman's rho = .282, p <.0001), procedure time (Spearman's rho .023, p = .52), and total anesthesia time (Spearman's rho, .032). Total OME narcotics were correlated negatively with age of 65 years or older (Spearman's rho, -.144, p <.0001) and use of patient-controlled analgesia (Spearman's rho, -.185, p <.0001).Conclusion: Age younger than 65 years seems to be a predictor for increased requirement of total morphine equivalent medication after RAL surgery, whereas patient-controlled analgesia use had a negative association. Journal of Minimally Invasive Gynecology (2023) 30, 926-930. (c) 2023 AAGL. This is an open access article under the CC BY license (http://creativecommons.org/licenses/by/4.0/)

C1 [Stewart, Chelsea; Moreno, Andrea; Vo, Elise; Chase, Dana M.] Univ Sch Med, Dept Obstet & Gynecol, Phoenix, AZ USA.

[Bhattarai, Bikash] Creighton Univ, Dept Clin Res, Sch Med, Phoenix, AZ USA.

[Bhattarai, Bikash; Willmott, Lyndsay; Monk, Bradley J.] Univ Arizona, Dept Obstet & Gynecol, Coll Med, Phoenix, AZ USA.

[Bhattarai, Bikash] ValleyWise Hlth, Phoenix, AZ USA.

[Farley, John] Dign Hlth St Josephs Hosp & Med Ctr, Div Gynecol Oncol, Phoenix, AZ USA.

[Chase, Dana M.] David Geffen UCLA Sch Med, Los Angeles, CA 90095 USA.

C3 Creighton University; University of Arizona; St. Joseph's Hospital and

Medical Center; University of California System; University of

California Los Angeles; University of California Los Angeles Medical

Center; David Geffen School of Medicine at UCLA

RP Chase, DM (corresponding author), David Geffen UCLA Sch Med, Los Angeles, CA 90095 USA.

EM dmchase@mednet.ucla.edu

OI Chase, Dana/0000-0002-1073-8688

NR 9

TC 0

Z9 0

U1 0

U2 0

PU ELSEVIER SCIENCE INC

PI NEW YORK

PA STE 800, 230 PARK AVE, NEW YORK, NY 10169 USA

SN 1553-4650

EI 1553-4669

J9 J MINIM INVAS GYN

JI J. Minim. Invasive Gynecol.

PD NOV

PY 2023

VL 30

IS 11

BP 926

EP 930

DI 10.1016/j.jmig.2023.07.011

EA NOV 2023

PG 5

WC Obstetrics & Gynecology

WE Science Citation Index Expanded (SCI-EXPANDED)

SC Obstetrics & Gynecology

GA Z0LT2

UT WOS:001109091700001

PM 37506878

DA 2024-01-18

ER

PT J

AU Snyder, A

Baumfeld, Y

Shobeiri, SA

AF Snyder, Alexandra

Baumfeld, Yael

Shobeiri, S. Abbas

TI Robotic Surgery Trends and Efficiency in a High-Volume Gynecologic

Surgery Center

SO JOURNAL OF GYNECOLOGIC SURGERY

LA English

DT Article; Early Access

DE Robotic-assisted laparoscopic surgery; high volume medical center;

efficiency

ID HYSTERECTOMY; LAPAROSCOPY

AB Objective: This study evaluated trends and efficiency of robotic surgeons per surgical volume, procedure type, and subspecialty. As robotic surgery is more popular in gynecologic subspecialties, studies comparing surgical volume suggest that robotic gynecologic surgery is advantageous when performed by high-volume surgeons and used for complex surgeries.Materials and Methods: During 2017-2021, evaluations were made of 3914 robotic gynecologic surgery cases performed at the Inova Fairfax Women's Hospital, Fairfax, VA, USA. All patients having robotic gynecologic surgery during the study were included. Surgeons were divided into high-, medium-, and low-volume groups. Statistical analysis evaluated changes in procedure types, surgeon subspecialties, operating times, and surgeon volumes.Results: Over the 5 years, there was a significant increase in adnexal surgery, surgery for pelvic organ prolapse, and myomectomy. High-volume surgeons performed more-complex surgeries overall. Operating room (OR) and surgery times were significantly lower for high-volume surgeons than for other groups.Conclusions: There was an increased variety of procedure types performed on the Da Vinci (R) robot over the 5 years. The data showed that high-volume surgeons had shorter OR and surgery times while performing more-complicated surgeries. This supports that high-volume gynecologic surgeons perform robotic cases more efficiently. With the increased interest in robotic surgery among surgeons and patients, an ongoing analysis of robotic surgery trends is essential to optimize availability, usage, and outcomes of these surgeries. (J GYNECOL SURG 20XX:000)

C1 [Snyder, Alexandra; Baumfeld, Yael; Shobeiri, S. Abbas] INOVA Womens Hosp, Inova Hlth Syst, Dept Obstet & Gynecol, Falls Church, VA USA.

[Shobeiri, S. Abbas] George Mason Univ, Dept Bioengn, Fairfax, VA USA.

[Shobeiri, S. Abbas] Inova Womens Hosp, INOVA Hlth Syst, Dept Obstet & Gynecol, 3300 Gallows Rd,Second Floor,South Tower, Falls Church, VA 22042 USA.

C3 Inova Fairfax Hospital; Inova Health System; George Mason University;

Inova Fairfax Hospital; Inova Health System

RP Shobeiri, SA (corresponding author), Inova Womens Hosp, INOVA Hlth Syst, Dept Obstet & Gynecol, 3300 Gallows Rd,Second Floor,South Tower, Falls Church, VA 22042 USA.

EM Abbas.Shobeiri@inova.org

FU Department of Obstetrics and Gynecology at the Inova Fairfax Hospital

FX This study was supported by the Department of Obstetrics and Gynecology

at the Inova Fairfax Hospital. The authors thank Raven Eberth, BS, and

Suha Suliman, BS, (University of Virginia medical students) for

contributions to data collection and chart review.

NR 17

TC 0

Z9 0

U1 0

U2 0

PU MARY ANN LIEBERT, INC

PI NEW ROCHELLE

PA 140 HUGUENOT STREET, 3RD FL, NEW ROCHELLE, NY 10801 USA

SN 1042-4067

EI 1557-7724

J9 J GYNECOL SURG

JI J. Gynecol. Surg.

PD 2023 NOV 2

PY 2023

DI 10.1089/gyn.2023.0020

EA NOV 2023

PG 6

WC Obstetrics & Gynecology; Surgery

WE Emerging Sources Citation Index (ESCI)

SC Obstetrics & Gynecology; Surgery

GA EF1W3

UT WOS:001137425100001

DA 2024-01-18

ER

PT J

AU Baeten, IGT

Hoogendam, JP

Schreuder, HWR

Jürgenliemk-Schulz, IM

Gerestein, CG

Zweemer, RP

AF Baeten, Ilse G. T.

Hoogendam, Jacob P.

Schreuder, Henk W. R.

Jurgenliemk-Schulz, Ina M.

Gerestein, Cornelis G.

Zweemer, Ronald P.

TI Introducing a novice surgeon to an experienced robotic gynaecological

oncology team: An observational cohort study on the impact of a

structured curriculum on outcomes of cervical cancer surgery

SO GYNECOLOGIC ONCOLOGY

LA English

DT Article

DE Cervical cancer; Robot-assisted surgery; Learning curve; Cumulative sum

analysis

ID OPEN RADICAL HYSTERECTOMY; LEARNING-CURVE; PELVIC LYMPHADENECTOMY;

LAPAROSCOPIC SURGERY; STAGE; SINGLE; SIMULATION; CARCINOMA; SOCIETY; NO

AB Objective. To evaluate the effect on patient outcomes when introducing a novice robotic surgeon, trained in accordance with a structured learning curriculum, to an experienced robotic surgery team treating cervical cancer patients.Methods. Patients with early-stage cervical cancer who were treated with primary robot-assisted surgery be-tween 2007 and 2019 were retrospectively included. In addition to the 165 patients included in a former analysis, we included a further 61 consecutively treated patients and divided all 226 patients over three groups: early learning phase of 61 procedures without structured training (group 1), experienced phase of 104 procedures (group 2), and the 61 procedures during introduction of a novice with structured training (group 3). Risk-adjusted cumulative sum (RA-CUSUM) analysis was performed to assess the learning curve effect. Patient out-comes between the groups were compared.Results. Based on RA-CUSUM analysis, no learning curve effect was observed for group 3. Regarding surgical outcomes, mean operation time in group 3 was significantly shorter than group 1 (p < 0.001) and similar to group 2 (p = 0.96). Proportions of intraoperative and postoperative adverse events in group 3 were not significantly different from the experienced group (group 2). Regarding oncological outcomes, the 5-year disease-free survival, disease-specific survival, and overall survival in group 3 were not significantly different from the experienced group.Conclusions. Introducing a novice robotic surgeon, who was trained in accordance with a structured learning curriculum, resulted in similar patient outcomes as by experienced surgeons suggesting novices can progress through a learning phase without compromising outcomes of cervical cancer patients.(c) 2023 The Author(s). Published by Elsevier Inc. This is an open access article under the CC BY license (http:// creativecommons.org/licenses/by/4.0/).

C1 [Baeten, Ilse G. T.; Hoogendam, Jacob P.; Schreuder, Henk W. R.; Gerestein, Cornelis G.; Zweemer, Ronald P.] Univ Utrecht, Univ Med Ctr Utrecht, Dept Gynaecol Oncol, Div Imaging & Oncol, Utrecht, Netherlands.

[Jurgenliemk-Schulz, Ina M.] Univ Utrecht, Univ Med Ctr Utrecht, Dept Radiol & Nucl Med, Div Imaging & Oncol, Utrecht, Netherlands.

[Baeten, Ilse G. T.] Univ Med Ctr Utrecht, Dept Gynaecol Oncol, Div Imaging & Oncol, POB 85500, NL-3508 GA Utrecht, Netherlands.

C3 Utrecht University; Utrecht University Medical Center; Utrecht

University; Utrecht University Medical Center; Utrecht University;

Utrecht University Medical Center

RP Baeten, IGT (corresponding author), Univ Med Ctr Utrecht, Dept Gynaecol Oncol, Div Imaging & Oncol, POB 85500, NL-3508 GA Utrecht, Netherlands.

EM i.g.t.baeten@umcutrecht.nl

NR 51

TC 0

Z9 0

U1 0

U2 0

PU ACADEMIC PRESS INC ELSEVIER SCIENCE

PI SAN DIEGO

PA 525 B ST, STE 1900, SAN DIEGO, CA 92101-4495 USA

SN 0090-8258

EI 1095-6859

J9 GYNECOL ONCOL

JI Gynecol. Oncol.

PD NOV

PY 2023

VL 178

BP 153

EP 160

DI 10.1016/j.ygyno.2023.10.008

PG 8

WC Oncology; Obstetrics & Gynecology

WE Science Citation Index Expanded (SCI-EXPANDED)

SC Oncology; Obstetrics & Gynecology

GA Y4AQ1

UT WOS:001104710900001

PM 37865051

OA Green Submitted

DA 2024-01-18

ER

PT J

AU Rodriguez, MA

Hooker, RS

AF Rodriguez, Melissa A.

Hooker, Roderick S.

TI Procedures by Physician Associates in Obstetrics and Gynecology

SO WOMENS HEALTH REPORTS

LA English

DT Article; Proceedings Paper

CT Annual Conference of the American-Academy-Physician-Associates (AAPA)

CY MAY 20-24, 2023

CL Nashville, TN

SP Amer Acad Physician Assoc

DE physician associate; obstetrics; gynecology; workforce; procedures

ID PROVIDERS

AB Background and Objectives: The number of obstetricians and gynecologists in the United States is decreasing and providers backfilling this service have not been well described. The intent of the study was to identify the skills that physician associates (PAs) in obstetrics and gynecology (OBGyn) contribute to this aspect of medicine and surgery.Methods: A survey of PAs specializing in OBGyn was conducted in 2022. The intent was to list office-based procedures that were part of their skill set. A vetted questionnaire was sent to the 1,630 American Academy of Physician Associates members who identified themselves in OBGyn at some point in their career, and 729 responded (44.7% relative risk).Results: Most PAs (88.7%) in OBGyn first assist in surgery. This first-assist role ranged across the open, laparoscopic, and robotic-type operations. Categories of surgery included Cesarean section, hysterectomy, salpingo-oophorectomy, and subspecialty surgeries such as oncology and urogynecology. In the outpatient setting, PAs listed over 40 procedures ranging from biopsies of the endometrium, cervix, vagina, and vulva, as well as fetal assessment, ultrasonography, and long-acting contraceptive insertion and removals.Conclusions: The proceduralist role of PAs in OBGyn is broad. Furthermore, this role may need to be utilized more at a time of growing scarcity of clinicians. The OBGyn role for PAs adds to their specialization and increasing presence in American medicine.

C1 [Rodriguez, Melissa A.] Orlando Hlth Winnie Palmer Hosp Women & Babies, Orlando, FL USA.

[Rodriguez, Melissa A.] Orlando Hlth Winnie Palmer Hosp Women & Babies, 83 W Miller St, Orlando, FL 32806 USA.

RP Rodriguez, MA (corresponding author), Orlando Hlth Winnie Palmer Hosp Women & Babies, 83 W Miller St, Orlando, FL 32806 USA.

EM merodriguez417@gmail.com

FU American Academy of Physician Associates

FX The study was supported by a Small Research Grant 2021-2022 from the

American Academy of Physician Associates. This work was carried out

under the Institutional Review Board of Orlando Health.

NR 21

TC 0

Z9 0

U1 0

U2 0

PU MARY ANN LIEBERT, INC

PI NEW ROCHELLE

PA 140 HUGUENOT STREET, 3RD FL, NEW ROCHELLE, NY 10801 USA

EI 2688-4844

J9 WOMENS HEALTH REP

JI Womens Health Reports

PD NOV 1

PY 2023

VL 4

IS 1

BP 536

EP 543

DI 10.1089/whr.2023.0044

PG 8

WC Obstetrics & Gynecology

WE Emerging Sources Citation Index (ESCI)

SC Obstetrics & Gynecology

GA Y3ZM6

UT WOS:001104681400002

PM 38023373

OA Green Published, gold

DA 2024-01-18

ER

PT J

AU Togami, S

Fukuda, M

Mizuno, M

Yanazume, S

Kobayashi, H

AF Togami, Shinichi

Fukuda, Mika

Mizuno, Mika

Yanazume, Shintaro

Kobayashi, Hiroaki

TI Efficacy and prognosis of robotic surgery with sentinel node navigation

surgery in endometrial cancer

SO JOURNAL OF GYNECOLOGIC ONCOLOGY

LA English

DT Article

DE Endometrial Cancer; Prognosis; Surgery; Sentinel Lymph Node; Lymph Nodes

ID PELVIC LYMPHADENECTOMY; MAPPING ALGORITHM; RISK-FACTORS; LYMPH-NODES;

MULTICENTER; BIOPSY; GUIDELINES; SURVIVAL; IMPACT; SAFETY

AB Objective: This study aimed to validate the surgical and oncologic outcomes of robotic surgery with sentinel node navigation surgery (SNNS) in endometrial cancer.Methods: This study included 130 patients with endometrial cancer, who underwent robotic surgery, including hysterectomy, bilateral salpingo-oophorectomy, and pelvic SNNS at the Department of Obstetrics and Gynecology of Kagoshima University Hospital. Pelvic sentinel lymph nodes (SLNs) were identified using the uterine cervix 99m Technetium-labeled phytate and indocyanine green injections. Surgery-related and survival outcomes were also evaluated. Results: The median operative and console times and volume of blood loss were 204 (range: 101-555) minutes, 152 (range: 70-453) minutes, and 20 (range: 2-620) mL, respectively. The bilateral and unilateral pelvic SLN detection rates were 90.0% (117/130) and 5.4% (7/130), respectively, and the identification rate (the rate at which at least one SLN could be identified on either side) was 95% (124/130). Lower extremity lymphedema occurred in only 1 patient (0.8%), and no pelvic lymphocele occurred. Recurrence occurred in 3 patients (2.3%), and the recurrence site was the abdominal cavity, with dissemination in 2 patients and vaginal stump in one. The 3-year recurrence-free survival and 3-year overall survival rates were 97.1% and 98.9%, respectively.Conclusion: Robotic surgery with SNNS for endometrial cancer showed a high SLN identification rate, low occurrence rates of lower extremity lymphedema and pelvic lymphocele, and excellent oncologic outcomes.

C1 [Togami, Shinichi; Fukuda, Mika; Mizuno, Mika; Yanazume, Shintaro; Kobayashi, Hiroaki] Kagoshima Univ, Fac Med, Dept Obstet & Gynecol, Kagoshima, Japan.

[Kobayashi, Hiroaki] Kagoshima Univ, Fac Med, Dept Obstet & Gynecol, 8-35-1 Sakuragaoka, Kagoshima 8908520, Japan.

C3 Kagoshima University; Kagoshima University

RP Kobayashi, H (corresponding author), Kagoshima Univ, Fac Med, Dept Obstet & Gynecol, 8-35-1 Sakuragaoka, Kagoshima 8908520, Japan.

EM hirokoba@m2.kufm.kagoshima-u.ac.jp

OI KOBAYASHI, HIROAKI/0000-0003-2491-2189

NR 27

TC 2

Z9 2

U1 0

U2 0

PU KOREAN SOC GYNECOLOGY ONCOLOGY & COLPOSCOPY

PI SEOUL

PA 102-HO, 55-5, NONHYEON-DONG, GANGNAM-GU, SEOUL, 135-010, SOUTH KOREA

SN 2005-0380

EI 2005-0399

J9 J GYNECOL ONCOL

JI J. Gynecol. Oncol.

PD NOV

PY 2023

VL 34

IS 6

AR e68

DI 10.3802/jgo.2023.34.e68

PG 9

WC Oncology; Obstetrics & Gynecology

WE Science Citation Index Expanded (SCI-EXPANDED)

SC Oncology; Obstetrics & Gynecology

GA Z1PA2

UT WOS:001109859500008

PM 37293801

OA Green Published, gold

DA 2024-01-18

ER

PT J

AU Walker, Z

Gargiulo, A

AF Walker, Zachary

Gargiulo, Antonio

TI Near-infrared and hysteroscopy-guided robotic excision of uterine

isthmocele with laser fiber: a novel high-precision technique

SO FERTILITY AND STERILITY

LA English

DT Article

DE Carbon dioxide laser; cesarean scar defect; near-infrared; robotic

surgery; uterine isthmocele

ID SCAR DEFECT; CLOSURE

AB Objective: To describe a novel high-precision technique for robotic excision of uterine isthmocele, employing a carbon dioxide laser fiber, under hysteroscopic guidance, and near-infrared guidance.Design: Video article.Patient(s): A 36-year-old multipara with 3 prior cesarean sections presented to our infertility clinic with secondary infertility. The patient had been trying to conceive for 6 months without success. The patient underwent a hystero-salpingo contrast sonography that identified a large cesarean scar defect with a 1.4-mm residual myometrial thickness (RMT). The patient was counseled on surgical management with robotic approach because of RMT <3 mm precluding her from hysteroscopic resection and the potential risk for a cesarean scar ectopic or abnormal placentation if she were to become pregnant in the future. She elected to undergo excision and repair and informed consent was obtained from the patient.Intervention(s): The robot was docked for traditional gynecologic robotic surgery. The uterus was injected with 5 units of vasopressin. We used a carbon dioxide laser fiber (Lumenis FIberLase) at a power of 5 watts as the sole energy source for dissection. The bladder was dissected off the uterus to identify the general area of the isthmocele. At that point, diagnostic hysteroscopy was performed using a 30 -degree 5-mm hysteroscope (Karl Storz) to identify and enter the isthmocele. Near-infrared vision (da Vinci Firefly, Intuitive USA) was activated to precisely outline the extent of the isthmocele, which was not visible with simple transillumination from the hysteroscope. We proceeded with laser excision in infrared/gray scale using the laser at a power of 20 watts removing the entire area that was highlighted by the Firefly. After full excision of the isthmocele, the hysteroscope was removed and was eventually replaced by a uterine manipulator (ConMed VCare DX). The hysterotomy was closed with a 2-layer closure: 4 mattress sutures of 2-0 Vicryl (Ethicon) followed by a running 2-0 PDS Stratafix (Ethicon). The peritoneal layer was closed over these 2 layers with 2-0 PDS Stratafix (Ethicon) in a running fashion. The uterine manipulator was removed and a 14 French Malecot catheter (Bard) was placed in the uterine cavity to allow the healing to proceed with minimal risk of cervical stenosis. The bladder was backfilled to ensure integrity of the bladder wall. Interceed adhesion barrier (Gynecare) was then placed over the area of the repair and the procedure was concluded. The patient included in this video gave consent for publication of the video and posting of the video online including social media, the journal website, scientific literature websites (such as PubMed, ScienceDirect, Scopus, etc.), and other applicable sites.Main outcome measure(s): Completion of excision and repair of cesarean scar defect without surgical complications.Result(s): Robotic excision and repair of a sizable uterine isthmocele with carbon dioxide laser fiber and da Vinci Firefly was completed successfully without any surgical complications. Diagnostic hysteroscopy was used to positively identify the isthmocele and provide transillumination. However, the thickness of the cervical myometrium only allows the hysteroscopic light to shine through the thinnest portion of myometrium at the apex of the isthmocele, whereas the near-infrared vision allowed by the da Vinci Firefly technology was used to precisely identify the borders of the defect.

The carbon dioxide laser was used to completely remove the defect while avoiding damage to delicate reproductive tissue and over-excision. No complications were identified during the postoperative visit. Magnetic resonance imaging 3 months after the surgery revealed an RMT of 10 mm at the location of excision compared with the initial RMT of 1.4 mm.Conclusion(s): Currently, there is no gold-standard technique for surgical management of isthmocele. This is the first description of the combined use of hysteroscopy, near-infrared vision, and laser fiber for the robotic excision of isthmocele. This specific setup proves to be a useful technical improvement. The use of near-infrared vision combined with precise hysteroscopic targeting allows much clearer definition of he isthmocele borders, and the flexible laser fiber allows millimetric xcision in the absence of appreciable lateral thermal spread. Further investigation is warranted to identify a gold-standard surgical technique for patients with cesarean scar defect. (c) 2023 by American Society for Reproductive Medicine.) El resumen esta disponible en Espanol al final del articulo.

C1 [Walker, Zachary; Gargiulo, Antonio] Harvard Med Sch, Brigham & Womens Hosp, Dept Obstet Gynecol & Reprod Biol, Div Reprod Endocrinol & Infertil, Boston, MA USA.

[Walker, Zachary] Brigham & Womens Hosp, Dept Obstet Gynecol & Reprod Biol, Div Reprod Endocrinol & Infertil, 75 Francis St, Boston, MA 02115 USA.

C3 Harvard University; Brigham & Women's Hospital; Harvard Medical School;

Harvard University; Brigham & Women's Hospital

RP Walker, Z (corresponding author), Brigham & Womens Hosp, Dept Obstet Gynecol & Reprod Biol, Div Reprod Endocrinol & Infertil, 75 Francis St, Boston, MA 02115 USA.

EM zwalker1@bwh.harvard.edu

FU Boston Scientific, Inc.

FX Declaration of interests: Z.W. has nothing to disclose. A.G. is a

consultant for Medicaroid, Inc. and Boston Scientific, Inc., and an

advisory board member at Pregmune, Inc.

NR 11

TC 0

Z9 0

U1 1

U2 1

PU ELSEVIER SCIENCE INC

PI NEW YORK

PA STE 800, 230 PARK AVE, NEW YORK, NY 10169 USA

SN 0015-0282

EI 1556-5653

J9 FERTIL STERIL

JI Fertil. Steril.

PD NOV

PY 2023

VL 120

IS 5

BP 1081

EP 1083

DI 10.1016/j.fertnstert.2023.08.006

PG 3

WC Obstetrics & Gynecology; Reproductive Biology

WE Science Citation Index Expanded (SCI-EXPANDED)

SC Obstetrics & Gynecology; Reproductive Biology

GA X9IQ8

UT WOS:001101509800001

PM 37567494

DA 2024-01-18

ER

PT J

AU Frost, AS

Kohn, JR

Le Neveu, M

Brah, T

Okonkwo, O

Borahay, MA

Wu, HR

Simpson, K

Patzkowsky, KE

Wang, KC

AF Frost, Anja S.

Kohn, Jaden R.

Le Neveu, Margot

Brah, Tara

Okonkwo, Obianuju

Borahay, Mostafa A.

Wu, Harold

Simpson, Khara

Patzkowsky, Kristin E.

Wang, Karen C.

TI Laparoscopic administration of bupivacaine at the uterosacral ligaments

during benign laparoscopic and robotic hysterectomy: a randomized

controlled trial

SO AMERICAN JOURNAL OF OBSTETRICS AND GYNECOLOGY

LA English

DT Article

DE bupivacaine; minimally invasive hysterectomy; opioids; postoperative

pain

ID MINIMALLY INVASIVE HYSTERECTOMY; POSTOPERATIVE PAIN; MANAGEMENT;

ANALGESIA; BLOCK

AB BACKGROUND: Postoperative pain continues to be an undermanaged part of the surgical experience. Multimodal analgesia has been adopted in response to the opioid epidemic, but opioid prescribing practices remain high after minimally invasive hysterectomy. Novel adjuvant opioid-sparing analgesia to optimize acute postoperative pain control is crucial in pre-venting chronic pain and minimizing opioid usage.OBJECTIVE: This study aimed to determine the effect of direct laparo-scopic uterosacral bupivacaine administration on opioid usage and post-operative pain in patients undergoing benign minimally invasive (laparoscopic and robotic) hysterectomy.STUDY DESIGN: This was a single-blinded, triple-arm, randomized controlled trial at an academic medical center between March 15, 2021, and April 8, 2022. The inclusion criteria were patients aged >18 years undergoing benign laparoscopic or robotic hysterectomy. The exclusion criteria were noneEnglish-speaking patients, patients with an allergy to bupivacaine or actively using opioid medications, patients undergoing transversus abdominis plane block, and patients under-going supracervical hysterectomy or combination cases with other surgical services. Patients were randomized in a 1:1:1 fashion to the following uterosacral administration before colpotomy: no adminis-tration, 20 mL of normal saline, or 20 mL of 0.25% bupivacaine. All patients received incisional infiltration with 10 mL of 0.25% bupiva-caine. The primary outcome was 24-hour oral morphine equivalent usage (postoperative day 0 and postoperative day 1). The secondary outcomes were total oral morphine equivalent usage in 7 days, last day of oral morphine equivalent usage, numeric pain scores from the universal pain assessment tool, and return of bowel function. Patients reported postoperative pain scores, total opioid consumption, and re-turn of bowel function via Qualtrics surveys. Patient and surgical characteristics and primary and secondary outcomes were compared using chi-square analysis and 1-way analysis of variance. Multiple linear regression was used to identify predictors of opioid use in the first 24 hours after surgery and total opioid use in the 7 days after surgery.RESULTS: Of 518 hysterectomies screened, 410 (79%) were eligible, 215 (52%) agreed to participate, and 180 were ultimately included in the final analysis after accounting for dropout. Most hysterectomies (70%) were performed laparoscopically, and the remainder were performed robotically. Most hysterectomies (94%) were outpatient. Patients ran-domized to bupivacaine had higher rates of former and current tobacco use, and patients randomized to the no-administration group had higher rates of previous surgery. There was no difference in first 24-hour oral morphine equivalent use among the groups (P1/4.10). Moreover, there was no difference in numeric pain scores (although a trend toward significance in discharge pain scores in the bupivacaine group), total 7-day oral morphine equivalent use, day of last opioid use, or return of bowel function among the groups (P>.05 for all). The predictors of increased 24-hour opioid usage among all patients included only increased postanesthesia care unit oral morphine equivalent usage. The predictors of 7-day opioid usage among all patients included concurrent tobacco use and mood disorder, history of previous laparoscopy, estimated blood loss of >200 mL, and increased oral morphine equivalent usage in the postanesthesia care unit.

CONCLUSION: Laparoscopic uterosacral administration of bupiva-caine at the time of minimally invasive hysterectomy did not result in decreased opioid usage or change in numeric pain scores.

C1 [Frost, Anja S.; Borahay, Mostafa A.; Simpson, Khara; Patzkowsky, Kristin E.; Wang, Karen C.] Johns Hopkins Univ, Dept Gynecol & Obstet, Div Minimally Invas Gynecol Surg, Sch Med, Baltimore, MD 21218 USA.

[Kohn, Jaden R.; Le Neveu, Margot; Okonkwo, Obianuju] Johns Hopkins Univ, Sch Med, Dept Gynecol & Obstet, Baltimore, MD USA.

[Okonkwo, Obianuju] Johns Hopkins Bloomberg Sch Publ Hlth, Baltimore, MD USA.

C3 Johns Hopkins University; Johns Hopkins University; Johns Hopkins

University; Johns Hopkins Bloomberg School of Public Health

RP Frost, AS (corresponding author), Johns Hopkins Univ, Dept Gynecol & Obstet, Div Minimally Invas Gynecol Surg, Sch Med, Baltimore, MD 21218 USA.

EM afrost6@jh.edu

OI Frost, Anja/0000-0002-1754-1028

NR 16

TC 0

Z9 0

U1 0

U2 0

PU MOSBY-ELSEVIER

PI NEW YORK

PA 360 PARK AVENUE SOUTH, NEW YORK, NY 10010-1710 USA

SN 0002-9378

EI 1097-6868

J9 AM J OBSTET GYNECOL

JI Am. J. Obstet. Gynecol.

PD NOV

PY 2023

VL 229

IS 5

DI 10.1016/j.ajog.2023.07.047

EA OCT 2023

PG 14

WC Obstetrics & Gynecology

WE Science Citation Index Expanded (SCI-EXPANDED)

SC Obstetrics & Gynecology

GA Y4ZF2

UT WOS:001105350400001

PM 37531986

DA 2024-01-18

ER

PT J

AU Sastre, J

Mínguez, JA

Alcázar, JL

Chiva, L

AF Sastre, Juan

Minguez, Jose Angel

Alcazar, Juan Luis

Chiva, Luis

TI Microsurgical anastomosis of the fallopian tubes after tubal ligation: a

systematic review and meta-analysis

SO EUROPEAN JOURNAL OF OBSTETRICS & GYNECOLOGY AND REPRODUCTIVE BIOLOGY

LA English

DT Review

DE Tubal reversal; Tubal anastomosis; Tubal reanastomosis; Reproductive

surgery; IVF

ID STERILIZATION REVERSAL; FEMALE STERILIZATION; REANASTOMOSIS; FERTILITY

AB Objective: Between 20% and 30% of women who have undergone tubal ligation regret their decision. The alternative to regain fertility for these women is either in vitro fertilization or tubal re-anastomosis. This article presents a systematic review with meta-analysis to assess the current evidence on the efficacy of tubal recanalization surgery in patients who have previously undergone tubal ligation. Study design: The search was conducted in the World of Science (WOS) database, The Cochrane Library and ClinicalTrials.gov record using the keywords "tubal reversal", "tubal reanastomosis" and "tubal anastomosis". The review was carried out by two of the authors. Data from 22 studies were evaluated, comprising over 14,113 patients who underwent the studied surgery, following strict inclusion criteria: articles published between January 2012 and June 2022, in English and with a sample size bigger than 10 patients were included. A random-effects meta-analysis was performed. Results: The overall pregnancy rate after anastomosis was found to be 65.3 % (95 % CI: 61.0-69.6). The percentage of women who had at least one live birth, known as the birth rate, was 42.6 % (95 % CI: 34.9-51.4). Adverse outcomes after surgery were also examined: the observed abortion rate among women who underwent surgery was 9.4 % (95 % CI: 7.0-11.7), and the overall ectopic pregnancy rate was 6.8 % (95 % CI: 4.6-9.0). No differences were found between the outcomes when differentiating surgical approaches: laparotomy, laparoscopy, or robotic-assisted surgery. The patient's age was identified as the most significant determining factor for fertility restoration. Finally, when comparing the results of tubal reversal with in vitro fertilization, reversal procedures appear more favorable for patients over 35 years old, while the results are similar for patients under 35 years old, but more data is needed to evaluate this finding. Conclusion: Therefore, the available literature review demonstrates that surgical anastomosis following tubal ligation is a reproducible technique with relevant success rates, performed by multiple expert groups worldwide.

C1 [Sastre, Juan; Minguez, Jose Angel; Alcazar, Juan Luis; Chiva, Luis] Clin Univ Navarra, Dept Obstet & Gynecol, Navarra, Spain.

[Chiva, Luis] Clin Univ Navarra, C Marquesado de Sta Marta 1, Madrid 28027, Spain.

C3 University of Navarra; University of Navarra

RP Chiva, L (corresponding author), Clin Univ Navarra, C Marquesado de Sta Marta 1, Madrid 28027, Spain.

EM lchiva@unav.es

NR 33

TC 0

Z9 0

U1 0

U2 0

PU ELSEVIER

PI AMSTERDAM

PA RADARWEG 29, 1043 NX AMSTERDAM, NETHERLANDS

SN 0301-2115

EI 1872-7654

J9 EUR J OBSTET GYN R B

JI Eur. J. Obstet. Gynecol. Reprod. Biol.

PD DEC

PY 2023

VL 291

BP 168

EP 177

DI 10.1016/j.ejogrb.2023.10.017

EA OCT 2023

PG 10

WC Obstetrics & Gynecology; Reproductive Biology

WE Science Citation Index Expanded (SCI-EXPANDED)

SC Obstetrics & Gynecology; Reproductive Biology

GA Y0VH4

UT WOS:001102526900001

OA hybrid

DA 2024-01-18

ER

PT J

AU Hayashi, S

Yamanaka, Z

Kojima, J

Ono, M

Sasaki, T

Yamamoto, A

Ono, M

Futagami, M

Nishi, H

AF Hayashi, Shigehiro

Yamanaka, Zenta

Kojima, Junya

Ono, Masataka

Sasaki, Toru

Yamamoto, Akiko

Ono, Masanori

Futagami, Masayuki

Nishi, Hirotaka

TI Retrospective comparative study of robot-assisted surgery, laparoscopic

surgery, and laparotomy for endometrial cancer in patients with a low

risk of recurrence

SO JOURNAL OF OBSTETRICS AND GYNAECOLOGY RESEARCH

LA English

DT Article; Early Access

DE endometrial cancer; laparoscopy; laparotomy; retrospective studies;

robotics

ID MINIMALLY INVASIVE HYSTERECTOMY; ABDOMINAL HYSTERECTOMY;

LYMPHADENECTOMY; COMPLICATIONS; METASTASIS; DISEASE; WOMEN

AB Aim Minimally invasive surgeries for endometrial cancer are increasing worldwide. In Japan, some articles have examined surgical outcomes, but only a few have addressed oncological outcomes. This study aims to compare robot surgery, laparoscopic surgery, and laparotomy in terms of surgical and oncological outcomes within a low-risk group for endometrial cancer recurrence.Methods This study included patients with endometrial cancer deemed to be at low risk of recurrence and who underwent surgery between January 2011 and December 2020. We studied 99 patients who underwent robot surgery, 85 patients who underwent laparotomy, and 77 patients who underwent laparoscopic surgery. Surgical and oncological outcomes were compared retrospectively for these groups of patients.Results The median follow-up period was 47, 61, and 60 months in the laparotomy, laparoscopy, and robotic groups, respectively. The three groups had similar perioperative and pathological data. No significant differences in overall survival and disease-free survival were observed among the groups. Univariate and multivariate analyses conducted on the overall study population for disease-free survival and overall survival showed that the surgical approach did not have any influence. Minimally invasive surgery groups had longer operating times compared to the laparotomy group, but they had significantly less blood loss. The number of resected pelvic lymph nodes was similar, and the complication rate was not significant.Conclusions Robot-assisted surgery and laparoscopic surgery were found to be less invasive and showed similar oncologic outcomes compared to laparotomy surgery for endometrial cancer in patients with a low risk of recurrence.

C1 [Hayashi, Shigehiro; Yamanaka, Zenta; Kojima, Junya; Ono, Masataka; Sasaki, Toru; Yamamoto, Akiko; Ono, Masanori; Futagami, Masayuki; Nishi, Hirotaka] Tokyo Med Univ, Dept Obstet & Gynecol, Tokyo, Japan.

[Nishi, Hirotaka] Tokyo Med Univ, Dept Obstet & Gynecol, 6-7-1 Nishishinjuku, Tokyo 1608402, Japan.

C3 Tokyo Medical University; Tokyo Medical University

RP Nishi, H (corresponding author), Tokyo Med Univ, Dept Obstet & Gynecol, 6-7-1 Nishishinjuku, Tokyo 1608402, Japan.

EM nishih@tokyo-med.ac.jp

OI Nishi, Hirotaka/0000-0003-4110-7704; Yamamoto, Akiko/0000-0002-1952-7166

FU We would like to thank the members of the Department of Obstetrics and

Gynecology, Tokyo Medical University for useful discussions, Editage ()

for English language editing, and Satista () for statistical analysis.

FX We would like to thank the members of the Department of Obstetrics and

Gynecology, Tokyo Medical University for useful discussions, Editage ()

for English language editing, and Satista () for statistical analysis.

NR 24

TC 0

Z9 0

U1 0

U2 0

PU WILEY

PI HOBOKEN

PA 111 RIVER ST, HOBOKEN 07030-5774, NJ USA

SN 1341-8076

EI 1447-0756

J9 J OBSTET GYNAECOL RE

JI J. Obstet. Gynaecol. Res.

PD 2023 OCT 17

PY 2023

DI 10.1111/jog.15816

EA OCT 2023

PG 10

WC Obstetrics & Gynecology

WE Science Citation Index Expanded (SCI-EXPANDED)

SC Obstetrics & Gynecology

GA U5SI0

UT WOS:001085396200001

PM 37848280

OA Bronze

DA 2024-01-18

ER

PT J

AU Ballet, E

Rousseau, C

Barjat, TR

Chauleur, C

AF Ballet, Elodie

Rousseau, Clement

Barjat, Tiphaine Raia

Chauleur, Celine

TI Robotic retroperitoneal para-aortic lymphadenectomy via single-site port

SO JOURNAL OF GYNECOLOGY OBSTETRICS AND HUMAN REPRODUCTION

LA English

DT Article

DE Extraperitoneal lymphadenectomy; Robotic; Single -site port

AB Para-aortic staging is sometimes a standard feature in the management of pelvic cancers. Minimal invasive approach is recommended. Several routes are possible: extra-peritoneal or intraperitoneal depending on the expertise of the surgeon. We performed several extraperitoneal para-aortic lymphadenectomy using the Da Vinci Xi robotic system through single-site incision. We have developed a step-by-step guide from patient installation, installation of the Gelpoint V monotrocar, docking of the robot arms, to surgery, with the aim of performing the most efficient and safest procedure.The surgery does not differ from standard laparoscopic extraperitoneal lymphadenectomy. The advantages of minimally invasive robotic surgery in this indication are comparable to those of the standard laparoscopy approach. But through single-site incision, the Da Vinci Xi robot improves video quality, plus its wristed tools facilitates movements compared to conventional laparoscopy.

C1 [Ballet, Elodie; Rousseau, Clement; Barjat, Tiphaine Raia; Chauleur, Celine] St Etienne Univ Hosp Ctr, North Hosp, Gynecol & Obstet Dept, Ave Albert Raimond, F-42270 St Priest En Jarez, France.

[Barjat, Tiphaine Raia; Chauleur, Celine] Univ Jean Monnet St Etienne, Mines St Etienne, INSERM, SAINBIOSE U1059, F-42023 St Etienne, France.

[Chauleur, Celine] CHU St Etienne, NORTH Hosp, Dept Gynecol Obstet, Ave Albert Raimond, F-42270 St Priest En Jarez, France.

C3 CHU de St Etienne; Institut National de la Sante et de la Recherche

Medicale (Inserm); IMT - Institut Mines-Telecom; Mines Saint-Etienne;

CHU de St Etienne

RP Chauleur, C (corresponding author), CHU St Etienne, NORTH Hosp, Dept Gynecol Obstet, Ave Albert Raimond, F-42270 St Priest En Jarez, France.

EM celine.chauleur@chu-st-etienne.fr

NR 9

TC 0

Z9 0

U1 0

U2 0

PU ELSEVIER MASSON, CORP OFF

PI PARIS

PA 65 CAMILLE DESMOULINS CS50083 ISSY-LES-MOULINEAUX, 92442 PARIS, FRANCE

SN 2468-7847

EI 1773-0430

J9 J GYNECOL OBSTET HUM

JI J. Gynecol. Obstet. Hum. Reprod.

PD DEC

PY 2023

VL 52

IS 10

AR 102675

DI 10.1016/j.jogoh.2023.102675

EA OCT 2023

PG 4

WC Obstetrics & Gynecology

WE Science Citation Index Expanded (SCI-EXPANDED)

SC Obstetrics & Gynecology

GA W6CR1

UT WOS:001092489400001

OA Bronze

DA 2024-01-18

ER

PT J

AU Ballet, E

Rousseau, C

Barjat, TR

Chauleur, C

AF Ballet, Elodie

Rousseau, Clement

Barjat, Tiphaine Raia

Chauleur, Celine

TI Robotic retroperitoneal para-aortic lymphadenectomy via single-site port

SO JOURNAL OF GYNECOLOGY OBSTETRICS AND HUMAN REPRODUCTION

LA English

DT Article

DE Extraperitoneal lymphadenectomy; Robotic; Single-site port

AB Para-aortic staging is sometimes a standard feature in the management of pelvic cancers. Minimal invasive approach is recommended. Several routes are possible: extra-peritoneal or intraperitoneal depending on the expertise of the surgeon. We performed several extraperitoneal para-aortic lymphadenectomy using the Da Vinci Xi robotic system through single-site incision. We have developed a step-by-step guide from patient installation, installation of the Gelpoint V monotrocar, docking of the robot arms, to surgery, with the aim of performing the most efficient and safest procedure.

The surgery does not differ from standard laparoscopic extraperitoneal lymphadenectomy. The advantages of minimally invasive robotic surgery in this indication are comparable to those of the standard laparoscopy approach. But through single-site incision, the Da Vinci Xi robot improves video quality, plus its wristed tools facilitates movements compared to conventional laparoscopy.

C1 [Ballet, Elodie; Rousseau, Clement; Barjat, Tiphaine Raia; Chauleur, Celine] St Etienne Univ Hosp Ctr, North Hosp, Gynecol & Obstet Dept, Ave Albert Raimond, F-42270 St Priest En Jarez, France.

[Barjat, Tiphaine Raia; Chauleur, Celine] Univ Jean Monnet St Etienne, Mines St Etienne, INSERM, SAINBIOSE U1059, F-42023 St Etienne, France.

C3 CHU de St Etienne; Institut National de la Sante et de la Recherche

Medicale (Inserm); IMT - Institut Mines-Telecom; Mines Saint-Etienne

RP Chauleur, C (corresponding author), Univ Jean Monnet St Etienne, Mines St Etienne, INSERM, SAINBIOSE U1059, F-42023 St Etienne, France.; Chauleur, C (corresponding author), CHU St Etienne North Hosp, Dept Obstet Gynecol, Ave Albert Raimond, F-42270 St Priest En Jarez, France.

EM celine.chauleur@chu-st-etienne.fr

NR 9

TC 0

Z9 0

U1 0

U2 0

PU ELSEVIER MASSON, CORP OFF

PI PARIS

PA 65 CAMILLE DESMOULINS CS50083 ISSY-LES-MOULINEAUX, 92442 PARIS, FRANCE

SN 2468-7847

EI 1773-0430

J9 J GYNECOL OBSTET HUM

JI J. Gynecol. Obstet. Hum. Reprod.

PD DEC

PY 2023

VL 52

IS 10

AR 102675

DI 10.1016/j.jogoh.2023.102675

EA OCT 2023

PG 4

WC Obstetrics & Gynecology

WE Science Citation Index Expanded (SCI-EXPANDED)

SC Obstetrics & Gynecology

GA W1NA2

UT WOS:001089354100003

OA Bronze

DA 2024-01-18

ER

PT J

AU Della Corte, L

Guarino, MC

Vitale, SG

Angioni, S

Mercorio, A

Bifulco, G

Giampaolino, P

AF Della Corte, Luigi

Guarino, Maria Chiara

Vitale, Salvatore Giovanni

Angioni, Stefano

Mercorio, Antonio

Bifulco, Giuseppe

Giampaolino, Pierluigi

TI C-section technique vs minilaparotomy after minimally invasive uterine

surgery: a retrospective cohort study

SO ARCHIVES OF GYNECOLOGY AND OBSTETRICS

LA English

DT Article; Early Access

DE C-section technique; Minilaparotomy; Myomectomy; Laparoscopy; Pain

management

ID LAPAROSCOPIC MYOMECTOMY; HYSTERECTOMY

AB Purpose Uterine leiomyomas are benign uterine tumors. The choice of surgical treatment is guided by patient's age, desire to preserve fertility or avoid "radical" surgical interventions such as hysterectomy. In laparotomy, the issue of extracting the fibroid from the cavity does not arise. However, in laparoscopy and robotic surgery, this becomes a challenge. The aim of the present study was to determine the optimal surgical approach for fibroid extraction following laparoscopic or robotic myomectomy in terms of postoperative pain, extraction time, overall surgical time, scar size, and patient satisfaction.Methods A total of 51 patients met the inclusion criteria and were considered in our analysis: 33 patients who had undergone the "ExCITE technique" (Group A), and 18 patients a minilaparotomy procedure (Group B), after either simple myomectomy, multiple myomectomy, supracervical hysterectomy, or total hysterectomy. The diagnosis of myoma was histologically confirmed in all cases.Results Regarding the postoperative pain evaluation, at 6 h, patients reported 4 [3-4] vs 6 [5.3-7] on the VAS in Group A and B, as well as at 12 h, 2 [0-2] vs 3.5 [2.3-4] in Group A and B, respectively: both differences were statistically significant (p < 0.001). No statistically significant difference at 24 h from surgery was found. All patients in Group A were satisfied with the ExCITE technique, while in Group B only 67% of them. The length of the hospital stay was significantly shorter in Group A as compared to Group B (p = 0.007). In terms of the operative time for the extraction of the surgical specimen, overall operative time, and the scar size after the surgery, there was a statistically significant difference for those in Group A.Conclusion The ExCITE technique does not require specific training and allows the surgeon to offer a minimally invasive surgical option for patients, with also an aesthetic result. It is a safe and standardized approach that ensures tissue extraction without the need for mechanical morcellation.

C1 [Della Corte, Luigi] Univ Naples Federico II, Sch Med, Dept Neurosci Reprod Sci & Dent, I-80131 Naples, Italy.

[Guarino, Maria Chiara; Mercorio, Antonio; Bifulco, Giuseppe; Giampaolino, Pierluigi] Univ Naples Federico II, Dept Publ Hlth, I-80131 Naples, Italy.

[Vitale, Salvatore Giovanni; Angioni, Stefano] Univ Cagliari, Dept Surg Sci, Div Gynecol & Obstet, I-09124 Cagliari, Italy.

C3 University of Naples Federico II; University of Naples Federico II;

University of Cagliari

RP Della Corte, L (corresponding author), Univ Naples Federico II, Sch Med, Dept Neurosci Reprod Sci & Dent, I-80131 Naples, Italy.

EM dellacorte.luigi25@gmail.com

OI Della Corte, Luigi/0000-0002-0584-2181

FU There are no acknowledgments to declare.

FX There are no acknowledgments to declare.

NR 26

TC 0

Z9 0

U1 1

U2 1

PU SPRINGER HEIDELBERG

PI HEIDELBERG

PA TIERGARTENSTRASSE 17, D-69121 HEIDELBERG, GERMANY

SN 0932-0067

EI 1432-0711

J9 ARCH GYNECOL OBSTET

JI Arch. Gynecol. Obstet.

PD 2023 OCT 5

PY 2023

DI 10.1007/s00404-023-07239-7

EA OCT 2023

PG 8

WC Obstetrics & Gynecology

WE Science Citation Index Expanded (SCI-EXPANDED)

SC Obstetrics & Gynecology

GA T6DD1

UT WOS:001078863900001

PM 37796281

OA hybrid

DA 2024-01-18

ER

PT J

AU Andjic, M

Sleiman, Z

Sparic, R

Tomasevic, D

Morciano, A

Tinelli, A

AF Andjic, Mladen

Sleiman, Zaki

Sparic, Radmila

Tomasevic, Dina

Morciano, Andrea

Tinelli, Andrea

TI The Transvaginal Natural Orifice Transluminal Endoscopic Surgery

(vNOTES) Procedures in Contemporary Gynecology: An Appraisal of the

Published Evidence and a Review

SO CLINICAL AND EXPERIMENTAL OBSTETRICS & GYNECOLOGY

LA English

DT Review

DE vNOTES; gynecology; endoscopic surgery; laparoscopy; minimally invasive

surgery

ID VAGINAL SURGERY; HYSTERECTOMY; OUTCOMES; SERIES

AB Objective: The transvaginal natural orifice transluminal endoscopic surgery (vNOTES) is a kind of natural orifice transluminal endo-scopic surgery in which the abdominal cavity is reached by using the natural orifices, such as the stomach, rectum, esophagus, and bladder. In comparison to traditional laparoscopic and robotic surgery, there are potential advantages of the vNOTES. This narrative review shows the use of vNOTES in contemporary gynecologic endoscopic surgery. Mechanism: MEDLINE, Scopus, and PubMed searches on these themes were conducted from 1990 to 2023 using a mix of keywords. Papers and articles were identified and included in this narrative review after the authors' revision and evaluation. Findings in Brief: The vNOTES procedures allow a short surgery time, as well as estimated blood loss and postoperative pain. These procedures are safe and feasible in contemporary endoscopic gynecology surgery. Conclusions: The vNOTES procedures are beneficial for the patients, and to be added to other minimally invasive procedures, such as conventional laparoscopy and robotic surgery. However, further studies about the long-term outcomes of vNOTES procedures are still needed.

C1 [Andjic, Mladen; Sparic, Radmila] Univ Clin Ctr Serbia, Clin Gynecol & Obstet, Belgrade 11000, Serbia.

[Sleiman, Zaki] Lebanese Amer Univ, Med Ctr, Rizk Hosp, Dept Obstet & Gynecol, Beirut 4505, Lebanon.

[Sparic, Radmila] Univ Belgrade, Fac Med, Belgrade 11000, Serbia.

[Tomasevic, Dina] Gen Hosp Cacak, Clin Gen & Pediat Surg, Cacak 32000, Serbia.

[Morciano, Andrea] Pia Fdn Card G Panico, Dept Gynaecol & Obstet, I-73039 Tricase, Lecce, Italy.

[Tinelli, Andrea] Veris Ponti Hosp, Dept Obstet & Gynecol, I-73020 Scorrano, Lecce, Italy.

[Tinelli, Andrea] Veris Ponti Hosp, CERICSAL Ctr Ric Clin SALentino, I-73020 Scorrano, Lecce, Italy.

C3 Clinical Centre of Serbia; Lebanese American University; American

University of Beirut; University of Belgrade

RP Tinelli, A (corresponding author), Veris Ponti Hosp, Dept Obstet & Gynecol, I-73020 Scorrano, Lecce, Italy.; Tinelli, A (corresponding author), Veris Ponti Hosp, CERICSAL Ctr Ric Clin SALentino, I-73020 Scorrano, Lecce, Italy.

EM andreatinelli@gmail.com

RI Morciano, Andrea/GPS-9104-2022

OI Morciano, Andrea/0000-0001-5420-4277; Sparic,

Radmila/0000-0003-0515-1951

NR 52

TC 0

Z9 0

U1 0

U2 0

PU IMR PRESS

PI ROBINSON

PA 112 ROBINSON RD, ROBINSON, SINGAPORE

SN 0390-6663

EI 2709-0094

J9 CLIN EXP OBSTET GYN

JI Clin. Exp. Obstet. Gynecol.

PD OCT

PY 2023

VL 50

IS 10

AR 223

DI 10.31083/j.ceog5010223

PG 10

WC Obstetrics & Gynecology

WE Science Citation Index Expanded (SCI-EXPANDED)

SC Obstetrics & Gynecology

GA X8ST5

UT WOS:001101090700017

OA gold

DA 2024-01-18

ER

PT J

AU Minciuna, CE

Ivanov, M

Aioanei, S

Tudor, S

Lacatus, M

Vasilescu, C

AF Minciuna, Corina-Elena

Ivanov, Mihail

Aioanei, Sanziana

Tudor, Stefan

Lacatus, Monica

Vasilescu, Catalin

TI Short-term outcome of robotic and laparoscopic surgery for gynecological

malignancies: A single-center experience

SO GYNECOLOGY AND MINIMALLY INVASIVE THERAPY-GMIT

LA English

DT Article

DE Gynecologic neoplasms; gynecologic surgical procedures; laparoscopy;

minimally invasive surgical procedures; neoplasms; postoperative

complications; robotic surgical procedures

ID MINIMALLY-INVASIVE SURGERY; RADICAL HYSTERECTOMY; CERVICAL-CANCER;

POSTOPERATIVE COMPLICATIONS; PREVENTION; SURVIVAL

AB Objectives: Minimally invasive surgery (MIS) has become the preferred option for many gynecologic pathologies since complication rate and postoperative recovery time have decreased considerably. Postoperative complications remain an important aspect when using the MIS approach, if they are not timely or accurately diagnosed and treated. The main aim of the study is to first assess their incidence, followed by identifying possible risk factors. Furthermore, the secondary aim is to identify if the type of MIS approach used, robotic or laparoscopic, may render some additional benefits.Materials and Methods: The database of the General Surgery Department was queried between 2008 and 2019 for patients with gynecologic pathology: 2907 cases were identified. An additional selection was performed using the following filters: MIS and neoplasia. All emergency surgeries were excluded. One hundred and ninety-eight cases were obtained.Results: The majority of complications were urological (11.6%) with only 7.07% requiring a specific urological procedure. The second most common was lymphorrhea 4.5%. Dindo-Clavien classification correlates positively with the postoperative hospital stay (PHS) (P = 0.000), the type of surgery (P = 0.046), the primary tumor location (P = 0.011), conversion rate (P = 0.049), the expertise of the lead surgeon (P = 0.012), and the operative time (P = 0.002). The urological complications correlate positively with the type of surgery (P = 0.002), the tumor location (P = 0.001), early reintervention (P = 0.000), operative time (P = 0.006), postoperative hemorrhage (P = 0.000), pelvic abscess (P = 0.000), venous thrombosis (P = 0.011), and postoperative cardiac complications (P = 0.002). Laparoscopic and robotic approaches were comparatively assessed. The PHS (P = 0.025), the type of surgery performed (P = 0.000), and primary tumor location (P = 0.011) were statistically significantly different.Conclusion: Postoperative complications reported after MIS for gynecological malignancies show similar incidence as in the current literature, also taking into consideration those for the open approach. The robotic approach seems to be able to perform more complex surgeries with no difference in the postoperative complication rates. The expertise of the lead surgeon in gynecology correlates with lower postoperative complications. Further prospective studies are needed to confirm these results.

C1 [Minciuna, Corina-Elena; Ivanov, Mihail; Aioanei, Sanziana; Tudor, Stefan; Lacatus, Monica; Vasilescu, Catalin] Fundeni Clin Inst, Dept Gen Surg, Bucharest, Romania.

[Minciuna, Corina-Elena; Tudor, Stefan; Lacatus, Monica; Vasilescu, Catalin] Carol Davila Univ Med & Pharm, Dept Gen Surg, Bucharest, Romania.

C3 Institutul Clinic Fundeni; Carol Davila University of Medicine &

Pharmacy

RP Vasilescu, C (corresponding author), Fundeni Clin Inst, Dept Gen Surg, 258 Fundeni St, Bucharest, Romania.

EM catvasilescu@gmail.com

NR 20

TC 0

Z9 0

U1 0

U2 0

PU WOLTERS KLUWER MEDKNOW PUBLICATIONS

PI MUMBAI

PA WOLTERS KLUWER INDIA PVT LTD , A-202, 2ND FLR, QUBE, C T S NO 1498A-2

VILLAGE MAROL, ANDHERI EAST, MUMBAI, Maharashtra, INDIA

SN 2213-3070

EI 2213-3089

J9 GYNECOL MINIM INVASI

JI Gynecol. Minim. Invasive Ther.-GMIT

PD OCT-DEC

PY 2023

VL 12

IS 4

BP 236

EP 242

DI 10.4103/gmit.gmit_137_22

PG 7

WC Obstetrics & Gynecology

WE Emerging Sources Citation Index (ESCI)

SC Obstetrics & Gynecology

GA U0FW6

UT WOS:001081661200008

PM 38034104

OA gold, Green Published

DA 2024-01-18

ER

PT J

AU Nakai, C

Yamanoi, K

Horie, A

Yamaguchi, K

Hamanishi, J

Mandai, M

AF Nakai, Chihiro

Yamanoi, Koji

Horie, Akihito

Yamaguchi, Ken

Hamanishi, Junzo

Mandai, Masaki

TI Investigation of the effect of puncture order and position on the

difficulty of lower and middle abdominal port placement

SO GYNECOLOGY AND MINIMALLY INVASIVE THERAPY-GMIT

LA English

DT Article

DE Laparoscopy; robotic-assisted surgery; trocar order; trocar place

ID SURGERY

AB Objectives: Port placements at the mid-abdomen (mainstay of robotic surgery [Rob]) appear to be difficult compared to that at lower abdomen (mainstay of conventional laparoscopy [Con-Lap]). We hypothesized that the reason for this may be the difference in port puncture places.Materials and Methods: We examined how the differences between the place and puncture order of ports affected Con-Lap cases with ports mainly placed in the lower abdomen and Rob cases with ports mainly placed in the middle abdomen. The trocar time was measured from the time when the puncture position and skin incision were determined and initiated, respectively, to the time when the port was punctured and fixed and used as the indicator of difficulty.Results: In the Con-Lap group analysis, the trocar time of the left lower port was longer (right lower: 77 s, middle lower: 117.5 s, and left lower: 138 s, P < 0.0001). In the Rob group analysis, the trocar time of the left most port was significantly longer (right-most: 89.0 s, right-middle: 92.5 s, left-middle: 121.0 s, and left-most: 197.0 s; P < 0.0001). In addition, the total trocar time was significantly longer in the first puncture at the right-middle port in the Rob group (right-most first: 8.4 min, right-middle first: 12.4 min, and left-middle first: 8.5 min, P = 0.0063).Conclusion: In the mid-abdomen port placement, mainstay of Rob cases, the puncture order, and port site have a significant impact on the difficulty of the procedure. It is preferable to avoid initially puncturing the right-middle port in case of the Rob.

C1 [Nakai, Chihiro; Yamanoi, Koji; Horie, Akihito; Yamaguchi, Ken; Hamanishi, Junzo; Mandai, Masaki] Kyoto Univ, Grad Sch Med, Dept Gynecol & Obstet, Kyoto, Japan.

[Yamanoi, Koji] Kyoto Univ, Grad Sch Med, Dept Gynecol & Obstet, 54 Shogoin Kawahara Cho,Sakyo Ku, Kyoto 6068507, Japan.

C3 Kyoto University; Kyoto University

RP Yamanoi, K (corresponding author), Kyoto Univ, Grad Sch Med, Dept Gynecol & Obstet, 54 Shogoin Kawahara Cho,Sakyo Ku, Kyoto 6068507, Japan.

EM kojiymni@kuhp.kyoto-u.ac.jp

NR 14

TC 0

Z9 0

U1 0

U2 0

PU WOLTERS KLUWER MEDKNOW PUBLICATIONS

PI MUMBAI

PA WOLTERS KLUWER INDIA PVT LTD , A-202, 2ND FLR, QUBE, C T S NO 1498A-2

VILLAGE MAROL, ANDHERI EAST, MUMBAI, Maharashtra, INDIA

SN 2213-3070

EI 2213-3089

J9 GYNECOL MINIM INVASI

JI Gynecol. Minim. Invasive Ther.-GMIT

PD OCT-DEC

PY 2023

VL 12

IS 4

BP 218

EP +

DI 10.4103/gmit.gmit_124_22

PG 10

WC Obstetrics & Gynecology

WE Emerging Sources Citation Index (ESCI)

SC Obstetrics & Gynecology

GA U0FW6

UT WOS:001081661200005

PM 38034114

OA Green Published, gold

DA 2024-01-18

ER

PT J

AU Yang, YY

Li, Z

Si, KY

Dai, QQ

Qiao, YY

Li, DZ

Zhang, L

Wu, F

He, J

Wu, GZ

AF Yang, Yingying

Li, Zhen

Si, Keyi

Dai, Qingqiang

Qiao, Yingying

Li, Dazhuang

Zhang, Li

Wu, Fan

He, Jia

Wu, Guizhu

TI Effectiveness of Laparoscopic Pectopexy for Pelvic Organ Prolapse

Compared with Laparoscopic Sacrocolpopexy

SO JOURNAL OF MINIMALLY INVASIVE GYNECOLOGY

LA English

DT Article

DE Effectiveness; Laparoscopic pectopexy; Laparoscopic sacrocolpopexy;

Pelvic organ prolapse; Quality of life

ID QUALITY-OF-LIFE; COMPARATIVE CLINICAL-TRIAL; QUESTIONNAIRE;

INCONTINENCE; VALIDATION; PFDI-20; WOMEN

AB Study Objective: To evaluate the clinical benefits of laparoscopic pectopexy vs laparoscopic sacrocolpopexy in women with pelvic organ prolapse (POP).Design: Prospective cohort study.Setting: A tertiary hospital.Patients: We included 203 patients with POP.Interventions: Laparoscopic pectopexy or laparoscopic sacrocolpopexy.Measurements and Main Results: Anatomic effectiveness was measured using the POP Quantification system, both before and after operation. Functional recovery effectiveness was evaluated using complications and recurrence rates within 1 year. Quality of life was assessed by the Pelvic Floor Distress Inventory-20 and Incontinence Quality of Life questionnaires at enrollment and postoperative months 3, 6, and 12. Comparisons between groups were performed using t test, chi-square test, and mixed-effects model with repeated measures. The analysis included 203 eligible patients (sacrocolpopexy, 101; pectopexy, 102). The proportion of robotic-assisted surgeries was lower in the pectopexy group than in the sacrocolpopexy group (15.7% vs 41.6%, p <.001). The average operation time of pectopexy was shorter than that of sacrocolpopexy (174.2 vs 187.7 minutes) with a mean difference of 13.5 minutes (95% confidence interval, 3.9-23.0; p = .006). Differences of intraoperative blood loss, length of hospital stay, and postoperative 7-day complications between groups were not significant. Anatomic successes were obtained in both groups with similar improvement in POP Quantification scores. The rate of urinary symptoms recurrence was higher in the pectopexy group (13.7%) than in the sacrocolpopexy group (5.0%) at the 1-year follow-up (odds ratio, 3.1; 95% confidence interval, 1.1-8.8, p = .032). The Pelvic Floor Distress Inventory-20 and Incontinence Quality of Life scores were better improved at postoperative months 3, 6, and 12 for laparoscopic pectopexy than for sacrocolpopexy.Conclusion: Laparoscopic pectopexy revealed comparable anatomic success, shorter operation time, and better improve-ment in quality of life scores of prolapse, colorectal-anal, and urinary symptoms at 1-year follow-up, possibly being an alter-native when sacrocolpopexy is not practicable. However, clinicians should pay more attention to the recurrence of urinary symptoms after pectopexy. Journal of Minimally Invasive Gynecology (2023) 30, 833-840.

C1 [Yang, Yingying; Li, Zhen] Tongji Univ, Shanghai Matern & Infant Hosp 1, Sch Med, Clin Res Unit,Shanghai Key Lab Maternal Fetal Med,, Shanghai, Peoples R China.

[Dai, Qingqiang; Qiao, Yingying; Zhang, Li; Wu, Fan; Wu, Guizhu] Tongji Univ, Shanghai Matern & Infant Hosp 1, Sch Med, Dept Gynecol,Shanghai Key Lab Maternal Fetal Med,S, Shanghai, Peoples R China.

[Si, Keyi] Naval Med Univ, Dept Mil Hlth Stat, Shanghai, Peoples R China.

[Li, Dazhuang] Fudan Univ, Sch Publ Hlth, Shanghai, Peoples R China.

[He, Jia] Tongji Univ, Sch Med, Shanghai, Peoples R China.

[Wu, Guizhu] Tongji Univ, Shanghai Matern & Infant Hosp 1, Sch Med, Dept Gynecol, 2699 West GaoKe Rd, Shanghai 200092, Peoples R China.

C3 Tongji University; Tongji University; Naval Medical University; Fudan

University; Tongji University; Tongji University

RP Wu, GZ (corresponding author), Tongji Univ, Shanghai Matern & Infant Hosp 1, Sch Med, Dept Gynecol, 2699 West GaoKe Rd, Shanghai 200092, Peoples R China.

EM 1905009@tongji.edu.cn

OI Yang, Yingying/0000-0001-5115-8375

FU Science and Technology Innovation Plan of Shanghai Science and

Technology Commission [22Y11906200]; National Natural Science Foundation

of China [82204047]; Shanghai Hospital Development Center [2023LYPYB03];

Pudong Medical Combination; [SHDC2022CRS050]; [PFYLT2022-17]

FX This study was supported by Science and Technology Innovation Plan of

Shanghai Science and Technology Commission (22Y11906200) , National

Natural Science Foundation of China (82204047) , Pudong Medical

Combination (PFYLT2022-17, 2023LYPYB03) , Shanghai Hospital Development

Center (SHDC2022CRS050) .

NR 26

TC 0

Z9 0

U1 1

U2 1

PU ELSEVIER SCIENCE INC

PI NEW YORK

PA STE 800, 230 PARK AVE, NEW YORK, NY 10169 USA

SN 1553-4650

EI 1553-4669

J9 J MINIM INVAS GYN

JI J. Minim. Invasive Gynecol.

PD OCT

PY 2023

VL 30

IS 10

DI 10.1016/j.jmig.2023.06.011

PG 10

WC Obstetrics & Gynecology

WE Science Citation Index Expanded (SCI-EXPANDED)

SC Obstetrics & Gynecology

GA W7MO6

UT WOS:001093431000001

PM 37369345

OA hybrid

DA 2024-01-18

ER

PT J

AU Daykan, Y

Rotem, R

O'Reilly, BA

AF Daykan, Yair

Rotem, Reut

O'Reilly, Barry A.

TI Robot-assisted laparoscopic pelvic floor surgery: Review

SO BEST PRACTICE & RESEARCH CLINICAL OBSTETRICS & GYNAECOLOGY

LA English

DT Review

DE Robotic-assisted surgery; Sacrocolpopexy; Hysteropexy; Pelvic floor

repair

ID ORGAN PROLAPSE; VESICOVAGINAL FISTULA; RADICAL PROSTATECTOMY;

SACROCOLPOPEXY; HYSTERECTOMY; COMPLICATIONS; REPAIR; SACROHYSTEROPEXY;

PRESERVATION; OUTCOMES

AB Minimally invasive surgical techniques have become more common in pelvic floor reconstructive urogynaecological surgery, specifically, robotic-assisted pelvic floor surgery. Female pelvic floor anatomy is complex, and some repairs require highly experienced surgical skills that can be gained more easily using robotic-assisted surgery. A common application of the robotic platform in urogynaecological surgeries includes sacrocolpopexy, which has become the gold standard approach in the last decade for the correction of apical prolapse. Additional procedures include sacrohysteropexy, sacrocervicopexy, fistula repair, and complex procedures involving the bladder and other pelvic organs.

Despite its increasing use and clear benefit in our field, data in the literature and, in particular, randomised controlled trials are sparse. This review provides an update, incorporating recently published literature and our personal experience in that field. (c) 2023 Published by Elsevier Ltd.

C1 [Daykan, Yair] Meir Med Ctr, Dept Obstet & Gynecol, Kefar Sava, Israel.

[Daykan, Yair] Tel Aviv Univ, Sackler Sch Med, Tel Aviv, Israel.

[Rotem, Reut; O'Reilly, Barry A.] Cork Univ, Matern Hosp, Dept Urogynaecol, Cork, Ireland.

[Rotem, Reut] Hebrew Univ Jerusalem, Shaare Zedek Med Ctr, Dept Obstet & Gynecol, Sch Med, Jerusalem, Israel.

C3 Tel Aviv University; Sackler Faculty of Medicine; Tel Aviv University;

Sackler Faculty of Medicine; University College Cork; Hebrew University

of Jerusalem; Shaare Zedek Medical Center

RP Daykan, Y (corresponding author), Meir Med Ctr, Dept Obstet & Gynecol, Kefar Sava, Israel.

EM yair.dykan@gmail.com

RI Daykan, Yair/AAF-2867-2021

OI Daykan, Yair/0000-0002-0447-2414

NR 59

TC 0

Z9 0

U1 5

U2 5

PU ELSEVIER SCI LTD

PI OXFORD

PA THE BOULEVARD, LANGFORD LANE, KIDLINGTON, OXFORD OX5 1GB, OXON, ENGLAND

SN 1521-6934

EI 1532-1932

J9 BEST PRACT RES CL OB

JI Best Pract. Res. Clin. Obstet. Gynaecol.

PD DEC

PY 2023

VL 91

AR 102418

DI 10.1016/j.bpobgyn.2023.102418

EA SEP 2023

PG 15

WC Obstetrics & Gynecology

WE Science Citation Index Expanded (SCI-EXPANDED)

SC Obstetrics & Gynecology

GA W0HR4

UT WOS:001088531400001

PM 37776580

DA 2024-01-18

ER

PT J

AU Marchand, G

Masoud, AT

Abdelsattar, A

King, A

Brazil, G

Ulibarri, H

Parise, J

Arroyo, A

Coriell, C

Goetz, S

Moir, C

Baruelo, G

Govindan, M

AF Marchand, Greg

Masoud, Ahmed Taher

Abdelsattar, Ahmed

King, Alexa

Brazil, Giovanna

Ulibarri, Hollie

Parise, Julia

Arroyo, Amanda

Coriell, Catherine

Goetz, Sydnee

Moir, Carmen

Baruelo, Geneva

Govindan, Malini

TI Systematic Review and Meta-analysis of laparoscopic radical hysterectomy

vs. Robotic assisted radical hysterectomy for early stage cervical

cancer

SO EUROPEAN JOURNAL OF OBSTETRICS & GYNECOLOGY AND REPRODUCTIVE BIOLOGY

LA English

DT Review

DE Hysterectomy; Laparoscopic radical hysterectomy; Robotic radical

hysterectomy

ID CONVENTIONAL LAPAROSCOPY; PELVIC LYMPHADENECTOMY; SURGERY; OUTCOMES;

RISK

AB Objective: Following compelling evidence that open techniques may be related to better survival and disease free survival rates, many gynecologic oncologists in the US have turned away from performing laparoscopic radical hysterectomy (LRH) and robotic radical hysterectomy (RRH) for the treatment of early-stage cervical cancer. While this may be warranted as a safety concern, there is little high-quality data on the head-to-head comparison of LRH and RRH and therefore little evidence to answer the question of where this decrease in patient survival is originating from. In our systematic review, we aimed to compare the complications and outcomes of LRH against those of RRH.Data Sources: We searched PubMed, Cochrane CENTRAL, Medline, ClinicalTrials.Gov, SCOPUS, and Web of Science from database inception until February 1st, 2022.Methods of Study Selection: A total of 676 studies were identified and screened through a manual three-step process. Ultimately 33 studies were included in our final analysis. We included all studies that compared LRH and RRH and included at least one of our selected outcomes. We included retrospective cohorts, prospective cohorts, case-control, and randomized clinical trials.Tabulation, Integration, and Results: Data was independently extracted manually by multiple observers and the analysis was performed using Review Manager Software. PRISMA guidelines were followed. We analyzed homogenous data using a fixed-effects model, while a random-effects model was used for heterogeneous outcomes. We found that following RRH, women had a decreased hospital stay (MD = 0.80[0.38,1.21],(P < 0.002). We found no differences in estimated blood loss (MD = 35.24[-0.40,70.89],(P = 0.05), blood transfusion rate ((OR = 1.32[0.86,2.02],(P = 0.20), rate of post-operative complications (OR = 0.84[0.60,1.17],(P = 0.30), the operative time (MD = 6.01[-4.64,16.66],(P = 0.27), number of resected lymph node (MD =-1.22[-3.28,0.84],(P = 0.25) intraoperative complications (OR = 0.78[0.51,1.19],(P = 0.25), five-year overall survival (OR = 1.37 [0.51,3.69],(P = 0.53), lifetime disease free survival (OR = 0.89[0.59,1.32],(P = 0.55), intraoperative and postoperative mortality (within 30 days) (OR = 1.30[0.66,2.54],(P = 0.44), and recurrence (OR = 1.14 [0.79,1.64],(P = 0.50).Conclusions: RRH seems to result in the patient leaving the hospital sooner after surgery. We were unable to find any differences in our ten other outcomes related to complications or efficacy. These findings suggest that the decreased survival seen in minimally invasive RH in previous studies could be due to factors inherent to both LRH and RRH.Prospero Prospective Registration Number: CRD42022273727.

C1 [Marchand, Greg; Masoud, Ahmed Taher; King, Alexa; Brazil, Giovanna; Ulibarri, Hollie; Parise, Julia; Arroyo, Amanda; Coriell, Catherine; Goetz, Sydnee; Moir, Carmen; Govindan, Malini] Marchand Inst Minimally Invas Surg, Mesa, AZ 85209 USA.

[Masoud, Ahmed Taher; Abdelsattar, Ahmed] Fayoum Univ, Fac Med, Al Fayyum, Egypt.

[Baruelo, Geneva] Midwestern Univ, Coll Osteopath Med, Glendale, AZ USA.

C3 Egyptian Knowledge Bank (EKB); Fayoum University; Midwestern University

RP Marchand, G (corresponding author), Marchand Inst Minimally Invas Surg, Mesa, AZ 85209 USA.

EM gm@marchandinstitute.org

RI Massoud, Ahmed Taher/T-6212-2018

OI Massoud, Ahmed Taher/0000-0003-4304-6781; Marchand,

Greg/0000-0003-4724-9148

NR 65

TC 0

Z9 0

U1 0

U2 0

PU ELSEVIER

PI AMSTERDAM

PA RADARWEG 29, 1043 NX AMSTERDAM, NETHERLANDS

SN 0301-2115

EI 1872-7654

J9 EUR J OBSTET GYN R B

JI Eur. J. Obstet. Gynecol. Reprod. Biol.

PD OCT

PY 2023

VL 289

BP 190

EP 202

DI 10.1016/j.ejogrb.2023.09.002

EA SEP 2023

PG 13

WC Obstetrics & Gynecology; Reproductive Biology

WE Science Citation Index Expanded (SCI-EXPANDED)

SC Obstetrics & Gynecology; Reproductive Biology

GA T6OY0

UT WOS:001079171200001

PM 37690282

DA 2024-01-18

ER

PT J

AU Ponce, J

Fernández, S

Barahona, M

Martínez, JM

Ortega, C

Martí, L

AF Ponce, Jordi

Fernandez, Sergi

Barahona, Marc

Martinez, Jose Manuel

Ortega, Carlos

Marti, Lola

TI Robotic-assisted para-aortic lymphadenectomy: Technique and indications

in gynecological oncology

SO BEST PRACTICE & RESEARCH CLINICAL OBSTETRICS & GYNAECOLOGY

LA English

DT Article

DE Robotic surgery; Lymphadenectomy; Minimally-invasive surgery; Oncology;

Gynecology

ID LYMPH-NODE DISSECTION; ENDOMETRIAL CANCER; CERVICAL-CANCER;

OVARIAN-CANCER; DOUBLE DOCKING; TRIAL; LAPAROSCOPY; CARCINOMA

AB The benefits of minimally-invasive surgeries have been documented, and they have been established as the preferred approach for gynecological surgeries. With the development of robotic surgery, many highly complex surgeries can benefit from these advantages. Due to the complexity of aortocaval lymphadenectomy, surgical technique protocols have been described to reduce risks by maximizing benefits.We describe the technique using five ports (4 robotic arms and an assistant) to work the upper abdominal field, and different instruments recommended in each of their positions to reduce errors and optimize surgical time. After the "step by step" description, we summarize indications of aortocaval lymphadenectomy for every gynecological cancer in different stages.& COPY; 2023 Elsevier Ltd. All rights reserved.

C1 [Ponce, Jordi; Fernandez, Sergi; Barahona, Marc; Ortega, Carlos; Marti, Lola] Univ Barcelona, Univ Hosp Bellvitge IDIBELL, Gynecol Dept, Barcelona, Spain.

[Ponce, Jordi] Hosp Univ Bellvitge, Serv Ginecol, Planta 17, Carrer Feixa Llarga, S-N, Lhospitalet De Llobregat 08097, Barcelona, Spain.

C3 Institut d'Investigacio Biomedica de Bellvitge (IDIBELL); Bellvitge

University Hospital; University of Barcelona; Institut d'Investigacio

Biomedica de Bellvitge (IDIBELL); Bellvitge University Hospital

RP Ponce, J (corresponding author), Hosp Univ Bellvitge, Serv Ginecol, Planta 17, Carrer Feixa Llarga, S-N, Lhospitalet De Llobregat 08097, Barcelona, Spain.

EM jponce@bellvitgehospital.cat; sfernandez@bellvitgehospital.cat;

mbarahona@bellvitgehospital.cat; jmartinezgar@bellvitgehospital.cat;

cortega@bellvitgehospital.cat; jmartinezgar@bellvitgehospital.cat

OI Martinez-Garcia, Jose Manuel/0000-0002-8322-7694

NR 34

TC 0

Z9 0

U1 1

U2 1

PU ELSEVIER SCI LTD

PI OXFORD

PA THE BOULEVARD, LANGFORD LANE, KIDLINGTON, OXFORD OX5 1GB, OXON, ENGLAND

SN 1521-6934

EI 1532-1932

J9 BEST PRACT RES CL OB

JI Best Pract. Res. Clin. Obstet. Gynaecol.

PD DEC

PY 2023

VL 91

AR 102401

DI 10.1016/j.bpobgyn.2023.102401

EA SEP 2023

PG 14

WC Obstetrics & Gynecology

WE Science Citation Index Expanded (SCI-EXPANDED)

SC Obstetrics & Gynecology

GA S4PW0

UT WOS:001071012700001

PM 37678062

DA 2024-01-18

ER

PT J

AU Fennimore, NJ

Fitch, K

Kiff, J

Nguyen, CG

Garg, B

Munro, EG

Bruegl, AS

AF Fennimore, Nicole J.

Fitch, Katherine

Kiff, Jaime

Nguyen, Christine G.

Garg, Bharti

Munro, Elizabeth G.

Bruegl, Amanda S.

TI Success Rates of Sentinel Lymph Node Mapping for Endometrial Cancer in

Patients with Body Mass Index &lt; 45 Compared with Body Mass Index ≥ 45

SO JOURNAL OF MINIMALLY INVASIVE GYNECOLOGY

LA English

DT Article

DE Lymphadenectomy; Minimally invasive; Obesity; Staging

ID MINIMALLY INVASIVE SURGERY; INDOCYANINE GREEN; OBESITY; MULTICENTER;

MORTALITY; BIOPSY; IMPACT

AB Study Objective: The objective is to evaluate the rate of sentinel lymph node (SLN) mapping in patients with body mass index (BMI [kg/m(2)]) BMI > 45 compared with < 45.Design: A retrospective chart review.Setting: Three urban referral-based settings-1 academic and 2 community based. Patients: Patients age > 18 years, with endometrial intraepithelial neoplasia or clinical stage 1 endometrial cancer who underwent robot-assisted total laparoscopic hysterectomy with attempted SLN mapping between January 2015 and December 2021.Interventions: Robot-assisted total laparoscopic hysterectomy with attempted SLN mapping.Measurements and Main Results: A total of 933 subjects were included: 795 (85.2%) with BMI < 45 and 138 (14.8%) with BMI > 45. Comparing the BMI < 45 with BMI > 45 group, bilateral mapping was successful in 541 (68.1%) vs 63 (45.7%), respectively. Unilateral mapping was successful in 162 (20.4%) vs 33 (23.9%), respectively. Failure to map occurred in 92 (11.6%) vs 42 (30.4%) (p <.001), respectively. Exploratory analysis also suggested an inverse relationship between success rate of bilateral SLN mapping and BMI, with patients with BMI < 20 having bilateral SLN mapping rates of 86.5% and patients with BMI > 61 having rates of 20.0%. The steepest decline in bilateral SLN mapping rates was from BMI group 46 to 50 compared to 51 to 55, at 55.4% to 37.5%, respectively. Adjusted odds ratio (compared with those with BMI < 30) for those in the BMI 30 to 44 group was 0.36 (95% confidence interval 0.21-0.60) and for those in the BMI > 45 group was 0.10 (95% confidence interval 0.06-0.19). Conclusion: There is a statistically significant lower rate of SLN mapping in patients with a BMI > 45 than BMI < 45. Understanding the success of SLN mapping in patients with morbid obesity is essential for preoperative counseling, surgical planning, and developing a risk-appropriate postoperative treatment plan. Journal of Minimally Invasive Gynecology (2023) 30, 735-741.(c) 2023 AAGL. All rights reserved.

C1 [Fennimore, Nicole J.; Fitch, Katherine; Nguyen, Christine G.; Garg, Bharti; Munro, Elizabeth G.; Bruegl, Amanda S.] Oregon Hlth & Sci Univ, Dept Obstet & Gynecol, Div Gynecol Oncol, 3181 SW Sam Jackson Pk Rd, Portland, OR 97239 USA.

[Kiff, Jaime] Univ Oklahoma, Dept Obstet & Gynecol, Oklahoma City, OK USA.

C3 Oregon Health & Science University; University of Oklahoma System;

University of Oklahoma Health Sciences Center

RP Fennimore, NJ (corresponding author), Oregon Hlth & Sci Univ, Dept Obstet & Gynecol, Div Gynecol Oncol, 3181 SW Sam Jackson Pk Rd, Portland, OR 97239 USA.

EM fennimon@ohsu.edu

OI Bruegl, Amanda/0000-0003-1989-983X

NR 20

TC 1

Z9 1

U1 1

U2 1

PU ELSEVIER SCIENCE INC

PI NEW YORK

PA STE 800, 230 PARK AVE, NEW YORK, NY 10169 USA

SN 1553-4650

EI 1553-4669

J9 J MINIM INVAS GYN

JI J. Minim. Invasive Gynecol.

PD SEP

PY 2023

VL 30

IS 9

DI 10.1016/j.jmig.2023.04.013

EA SEP 2023

PG 7

WC Obstetrics & Gynecology

WE Science Citation Index Expanded (SCI-EXPANDED)

SC Obstetrics & Gynecology

GA AU7T7

UT WOS:001121039600001

PM 37142090

DA 2024-01-18

ER

PT J

AU Isoyama, K

Matsuura, M

Hayasaka, M

Nagao, S

Nishimura, Y

Yoshioka, T

Imai, Y

Miyagi, E

Suzuki, Y

Saito, T

AF Isoyama, Kyoko

Matsuura, Motoki

Hayasaka, Misa

Nagao, Sachiko

Nishimura, Yoko

Yoshioka, Toshiki

Imai, Yuichi

Miyagi, Etsuko

Suzuki, Yukio

Saito, Tsuyoshi

TI Nationwide trends in and regional factors associated with minimally

invasive hysterectomy for benign indications in Japan

SO EUROPEAN JOURNAL OF OBSTETRICS & GYNECOLOGY AND REPRODUCTIVE BIOLOGY

LA English

DT Article

DE Laparoscopic hysterectomy; Minimally invasive surgery; Benign

gynecological disease; Regional disparities

ID DISPARITIES; SURGERY

AB Objective: To examine the prevalence trends of minimally invasive hysterectomy for benign indications in Japan and investigate regional disparities. Study Design: A retrospective cohort and ecological study using "The National Database of Health Insurance Claims and Specific Health Checkups of Japan (NDB) Open Data". Setting: Nationwide Japan. Patients: Individuals who underwent hysterectomy for benign indications from 2014 to 2020. Interventions: Trend analysis of minimally invasive surgery (MIS) rates through laparoscopic hysterectomies (LH) and robotic-assisted laparoscopic hysterectomies (RA-LH) at the national and prefecture levels. Examination of regional factors contributing to the disparity in MIS implementation rates by second medical service area (SMSA). Results: The number of LH has increased from 16,016 in 2014 to 27,755 in 2020. The nationwide MIS hysterectomy rate increased from 29% in 2014 to 55% in 2020 (p less than 0.001). More than 50% of hysterectomies have been performed as MIS since 2019. There was an increasing trend in MIS rates in all age groups. All prefectures except one showed a significant upward trend (p less than 0.05) in the MIS rates, but MIS rates varied widely (23-84%). In a multivariable model, the MIS was more likely to be performed in the SMSAs in western Japan (p = 0.011), in the SMSAs where the number of laparoscopy-qualified gynecologists is 5-10 (p = 0.013), and 11 or higher (p less than 0.001). Conclusions: This study reveals a shift towards minimally invasive surgery (MIS) in total hysterectomy procedures in Japan. However, significant disparities in the prevalence of MIS hysterectomy exist, potentially influenced by the number of laparoscopy-qualified gynecologists.

C1 [Isoyama, Kyoko; Matsuura, Motoki; Nagao, Sachiko; Nishimura, Yoko; Saito, Tsuyoshi] Sapporo Med Univ, Dept Gynecol, 291 West 16,South 1,Chuo Ku, Sapporo, Hokkaido 0608543, Japan.

[Hayasaka, Misa] Asahikawa Med Univ, Dept Obstet & Gynecol, Midorigaokahigashi 2-1-1-1, Asahikawa, Hokkaido 0788510, Japan.

[Yoshioka, Toshiki; Imai, Yuichi; Miyagi, Etsuko] Yokohama City Univ, Grad Sch Med, Dept Obstet & Gynecol, 3-9 Fukuura,Kanazawa Ku, Yokohama, Kanagawa 2360004, Japan.

[Suzuki, Yukio] Columbia Univ, Vagelos Coll Phys & Surg, Dept Obstet & Gynecol, Div Gynecol Oncol, New York, NY 10032 USA.

C3 Sapporo Medical University; Asahikawa Medical College; Yokohama City

University; Columbia University

RP Matsuura, M (corresponding author), Sapporo Med Univ, Dept Gynecol, 291 West 16,South 1,Chuo Ku, Sapporo, Hokkaido 0608543, Japan.; Suzuki, Y (corresponding author), Columbia Univ, Vagelos Coll Phys & Surg, Dept Obstet & Gynecol, Div Gynecol Oncol, New York, NY 10032 USA.

EM mmatsuura@sapmed.ac.jp; yetii@yokohama-cu.ac.jp

RI Matsuura, Motoki/AAC-9301-2022

OI Matsuura, Motoki/0000-0002-6589-6480

NR 29

TC 0

Z9 0

U1 1

U2 1

PU ELSEVIER

PI AMSTERDAM

PA RADARWEG 29, 1043 NX AMSTERDAM, NETHERLANDS

SN 0301-2115

EI 1872-7654

J9 EUR J OBSTET GYN R B

JI Eur. J. Obstet. Gynecol. Reprod. Biol.

PD OCT

PY 2023

VL 289

BP 129

EP 135

DI 10.1016/j.ejogrb.2023.08.388

EA SEP 2023

PG 7

WC Obstetrics & Gynecology; Reproductive Biology

WE Science Citation Index Expanded (SCI-EXPANDED)

SC Obstetrics & Gynecology; Reproductive Biology

GA T3SC1

UT WOS:001077208200001

PM 37660507

OA hybrid

DA 2024-01-18

ER

PT J

AU Tahapary, M

Timmerman, S

Ledger, A

Dewilde, K

Froyman, W

AF Tahapary, M.

Timmerman, S.

Ledger, A.

Dewilde, K.

Froyman, W.

TI Implementation of robot-assisted myomectomy in a large university

hospital: a retrospective descriptive study

SO FACTS VIEWS AND VISION IN OBGYN

LA English

DT Article

DE Myoma; leiomyoma; fibroid; myomectomy; robot assisted surgery; robot

-assisted myomectomy.

ID UTERINE LEIOMYOMAS; MANAGEMENT; FIBROIDS; HYSTERECTOMY; BURDEN

AB Background: Myomectomy is often the preferred treatment for symptomatic patients with myomas who wish to preserve their fertility, with a shift from open surgery towards minimally invasive techniques.Objectives: Retrospective study assessing patient and surgery characteristics, follow-up, and outcomes of robot -assisted myomectomy (RAM) and abdominal myomectomy (AM) in women treated between January 1, 2018, and February 28, 2022, in a Belgian tertiary care hospital.Materials and Methods: A descriptive analysis was conducted on consecutive patients who underwent myomectomies. 2018 was considered the learning curve for RAM. Main Outcome Measures: We assessed rate of open surgery, operation time, postoperative hospital stay, and operative complications. Results: In total, 94 RAMs and 15 AMs were performed. The rate of AMs was 56.5% in 2018 versus 2.3% after the learning curve. The median operation time for RAM was 136.5 minutes and 131 minutes for AM. Conversion rate for RAM was 0%. The median postoperative hospital stay after RAM was 1 night and 4 nights for AM. Postoperative complication rate was low, with only 14.9% and 33.3% of patients requiring pharmacological treatment of complications after RAM or AM, respectively. No surgical re-intervention was needed in any group.Conclusions: Implementation of RAM at our centre resulted in a significant reduction of open surgery rate. RAM demonstrated shorter hospital stays and a lower incidence of complications compared to AM. What is new? Our study highlights the successful adoption of RAM, showcasing its potential to replace AM even in complex cases. The findings affirm the safety and feasibility of RAM, supporting its use as a valuable technique for minimally invasive myomectomy.

C1 [Tahapary, M.; Timmerman, S.; Dewilde, K.; Froyman, W.] Univ Hosp Leuven, Dept Obstet & Gynecol, Leuven, Belgium.

[Tahapary, M.] Med Spectrum Twente, Afdeling Gynaecol, Enschede, Netherlands.

[Timmerman, S.; Ledger, A.; Dewilde, K.; Froyman, W.] Katholieke Univ Leuven, Dept Dev & Regenerat, Leuven, Belgium.

[Froyman, W.] UZ Leuven, Herestr 49, B-3000 Leuven, Belgium.

C3 KU Leuven; University Hospital Leuven; Medical Spectrum Twente; KU

Leuven; KU Leuven; University Hospital Leuven

RP Froyman, W (corresponding author), UZ Leuven, Herestr 49, B-3000 Leuven, Belgium.

EM wouter.froyman@uzleuven.be

OI Froyman, Wouter/0000-0002-1398-9124

NR 29

TC 0

Z9 0

U1 2

U2 2

PU UNIVERSA PRESS

PI WETTEREN

PA RUE HOENDER 24, WETTEREN, 9230, BELGIUM

SN 2032-0418

J9 FACTS VIEWS VIS OBGY

JI Facts Views Vis. ObGyn

PD SEP

PY 2023

VL 15

IS 3

BP 243

EP 250

PG 8

WC Obstetrics & Gynecology

WE Emerging Sources Citation Index (ESCI)

SC Obstetrics & Gynecology

GA U6HF4

UT WOS:001085786000007

PM 37742201

OA Green Published, Bronze

DA 2024-01-18

ER

PT J

AU D'Ancona, G

Merlot, B

Verrelli, L

Boulos, S

Dennis, T

Roman, H

AF D'Ancona, Gianmarco

Merlot, Benjamin

Verrelli, Ludovica

Boulos, Sari

Dennis, Thomas

Roman, Horace

TI Robotic management of isolated endometriosis of sciatic nerve: a

reproducible approach that can guide through the labyrinth of pelvic

neuroanatomy

SO FERTILITY AND STERILITY

LA English

DT Article

DE Endometriosis; sciatic nerve; robotic surgery; pelvic neuroanatomy;

nerve-sparing

ID DEEP ENDOMETRIOSIS

AB Objective: To present the robotic, standardized, and reproducible surgical technique we routinely use in our center to manage isolated endometriosis of the sciatic nerve.Design: Surgical video article.Setting: Tertiary referral center.Patient: A 36-year-old woman suffering from left-sided sciatica pain was diagnosed with an isolated endometriotic nodule of the left sciatic nerve at preoperative assessment. The patient included in this video gave consent for publication of the video and posting of the video online, including on social media, the journal website, scientific literature websites (such as PubMed, ScienceDirect, and Scopus), and other applicable sites.Intervention(s): Complete removal of the isolated endometriotic nodule of the sciatic nerve may be performed through a stepwise robotic approach. The surgery starts laterally with the opening of the iliolumbar space between the external iliac vessels and the psoas muscle, as well as the identification of the genitofemoral and obturator nerves. The lumbosacral trunk and emergence of the sciatic nerve were then identified medially and caudally to the obturator nerve. The surgery moves medially with the anterograde dissection of both the internal iliac artery and vein, which allows a safe approach to the posterior and medial limits of the nodule. Ligation of branches of internal iliac vessels directed toward the nodule may be necessary during this step. Isolation and ligation of obturator ves-sels are frequently required to obtain a bloodless dissection of the lateral limit of the nodule from the lateral pelvic wall. The complete removal of the nodule was then achieved using an alternating approach to all limits of the nodule previously identified, with subsequent release of the sciatic nerve.Main outcome measure(s): Description of the relevant pelvic neuroanatomy and the evaluation of robotic routes in the field of pelvic neurosurgery.Result(s): The use of standardized techniques together with the advantages of a robotic route can make the radical excision of isolated endometriosis of the sciatic nerve reproducible, feasible, and safe. Conclusion(s): Because of the complexity of neuroanatomy and the risk of severe complications, this surgery remains challenging, and patients affected by deep infiltrating endometriosis involving retroperitoneal neural structures should be referred to multidisciplinary management in expert centers.(Fertil Steril & REG; 2023;120:703-5. & COPY;2023 by American Society for Reproductive Medicine.)

C1 [D'Ancona, Gianmarco; Merlot, Benjamin; Verrelli, Ludovica; Boulos, Sari; Dennis, Thomas; Roman, Horace] IFEMEndo, Clin Tivoli Ducos, Bordeaux, France.

[D'Ancona, Gianmarco] Clin Tivoli Ducos, Ctr Endometriosis, 91 rue Riviere, F-33000 Bordeaux, France.

RP D'Ancona, G (corresponding author), Clin Tivoli Ducos, Ctr Endometriosis, 91 rue Riviere, F-33000 Bordeaux, France.

EM gianmarcodancona@libero.it

OI D'Ancona, Gianmarco/0000-0002-4594-7359

NR 5

TC 0

Z9 0

U1 0

U2 0

PU ELSEVIER SCIENCE INC

PI NEW YORK

PA STE 800, 230 PARK AVE, NEW YORK, NY 10169 USA

SN 0015-0282

EI 1556-5653

J9 FERTIL STERIL

JI Fertil. Steril.

PD SEP

PY 2023

VL 120

IS 3

BP 703

EP 705

DI 10.1016/j.fertnstert.2023.06.008

EA AUG 2023

PG 3

WC Obstetrics & Gynecology; Reproductive Biology

WE Science Citation Index Expanded (SCI-EXPANDED)

SC Obstetrics & Gynecology; Reproductive Biology

GA S2VN3

UT WOS:001069800200001

PM 37302779

DA 2024-01-18

ER

PT J

AU Ind, T

AF Ind, Thomas

TI Providing a standardised educational programme in robot-assisted

gynaecological surgery

SO BEST PRACTICE & RESEARCH CLINICAL OBSTETRICS & GYNAECOLOGY

LA English

DT Article

DE Education; Training; Assessment

ID CURRICULUM; VALIDATION; MORTALITY; SOCIETY; VOLUME

AB Standardisation of an educational programme in robotic gynae-cological surgery requires careful reflection to ensure that the correct surgeons are selected, that they are trained to the best of their ability, and that they have continued education into their careers. The generally agreed pathways included a proficiency-based progression model for procedures with validated assessment tools used for both formative and summative assessment. For new surgeons, a basic and advanced curriculum is required, involving tools on how to use the instruments as well as educational lectures and simulation. For advanced learning, there is a need for proctorship. To maintain their skills, a surgeon should demonstrate a reflective practice and continued good outcomes while adhering to a process of credentialing. Trainers should be validated on their ability to teach based on recognised training-the-trainers courses.Crown Copyright (c) 2023 Published by Elsevier Ltd. All rights reserved.

C1 [Ind, Thomas] Royal Marsden Hosp, Dept Gynaecol Oncol, London SW3 6JJ, England.

C3 Royal Marsden NHS Foundation Trust

RP Ind, T (corresponding author), Royal Marsden Hosp, Dept Gynaecol Oncol, London SW3 6JJ, England.

EM ThomasMedsec.Ind@rmh.nhs.uk

NR 22

TC 0

Z9 0

U1 0

U2 0

PU ELSEVIER SCI LTD

PI OXFORD

PA THE BOULEVARD, LANGFORD LANE, KIDLINGTON, OXFORD OX5 1GB, OXON, ENGLAND

SN 1521-6934

EI 1532-1932

J9 BEST PRACT RES CL OB

JI Best Pract. Res. Clin. Obstet. Gynaecol.

PD DEC

PY 2023

VL 91

AR 102399

DI 10.1016/j.bpobgyn.2023.102399

EA AUG 2023

PG 11

WC Obstetrics & Gynecology

WE Science Citation Index Expanded (SCI-EXPANDED)

SC Obstetrics & Gynecology

GA T0WG7

UT WOS:001075272600001

PM 37651956

DA 2024-01-18

ER

PT J

AU Park, SY

Cho, EH

Jeong, K

Yoo, HK

Lee, JH

Moon, HS

AF Park, So Yun

Cho, Eun Hye

Jeong, Kyungah

Yoo, Hae Kyung

Lee, Jung Hun

Moon, Hye-Sung

TI Robotic single-port hysterectomy versus robotic multisite hysterectomy

in benign gynecologic diseases: A retrospective comparison of clinical

and surgical outcomes

SO JOURNAL OF OBSTETRICS AND GYNAECOLOGY RESEARCH

LA English

DT Article

DE hysterectomy; multisite; robotic surgery; single-port; uterus

ID SITE; SURGERY; LAPAROSCOPY

AB Background: This study aimed to compare clinical and surgical outcomes of robotic single-port hysterectomy (RSPH) using the da Vinci((R)) SP surgical system and robotic multisite hysterectomy (RMSH) with the da Vinci Xi system in benign gynecologic disease.

Methods: The retrospective study included 134 patients who underwent RSPH or RMSH between November 2019 and December 2020. Total operation time, amount of blood loss, and the change in hemoglobin (Hb) after surgery and the weight of the removed uteri were also measured. Data on complications such as post-operative fever and length of hospitalization were also compared and analyzed.

Results: There was no significant difference in the total operation time between the two groups, although the operation time was slightly longer in the RSPH group. Results in the RSPH group were superior to the RMSH group in docking time and wound incision time (1.67 +/- 0.79 vs. 5.46 +/- 2.25 min, p-value <0.01; 6.48 +/- 4.29 vs. 9.10 +/- 4.64 min, p-value <0.01, respectively). On the other hand, wound suture time took longer in the RSPH group (18.12 +/- 5.66 vs. 10.69 +/- 3.18 min, p-value <0.01). The weights of the removed specimens were higher in the RMSH group (302.64 +/- 190.56 vs. 369.24 +/- 181.70 g, p-value <0.04). The amount of blood loss during surgery and the difference in hemoglobin (Hb) before and after surgery were less in the RSPH group (97.39 +/- 113.79 vs. 224.93 +/- 152.29 mL, p-value <0.01, 1.51 +/- 1.08 vs. 2.54 +/- 1.08 g/dL, p-value <0.01). When considering the weight difference as a correction between the two surgical groups (because there were many heavier samples in the RMSH group), the blood loss of the RSPH group was also less than that of the RMSH group by 115.95 +/- 23.78 mL (p-value <0.01).

Conclusions: On the basis of our data, the robotic hysterectomy using the da Vinci SP surgical system might be feasible and safe, even if the hysterectomy is complex, and comparable to robotic multisite surgery by the da Vinci Xi system.

C1 [Park, So Yun; Cho, Eun Hye; Yoo, Hae Kyung; Lee, Jung Hun; Moon, Hye-Sung] Ewha Womans Univ, Ewha Womans Univ Seoul Hosp, Coll Med, Dept Obstet & Gynecol, Seoul, South Korea.

[Jeong, Kyungah] Ewha Womans Univ, Mokdong Hosp, Coll Med, Dept Obstet & Gynecol, Seoul, South Korea.

[Jeong, Kyungah] Ewha Womans Univ, MokDong Hosp, Coll Med, Dept Obstet & Gynecol, 1071 AnYangCheon Ro, Seoul 07985, South Korea.

C3 Ewha Womans University; Ewha Womans University; Ewha Womans University

RP Jeong, K (corresponding author), Ewha Womans Univ, MokDong Hosp, Coll Med, Dept Obstet & Gynecol, 1071 AnYangCheon Ro, Seoul 07985, South Korea.

EM ogjeong@ewha.ac.kr

OI Lee, Jung Hun/0000-0002-7221-6455; CHO, EUNHYE/0000-0002-1370-0992

NR 15

TC 0

Z9 0

U1 0

U2 0

PU WILEY

PI HOBOKEN

PA 111 RIVER ST, HOBOKEN 07030-5774, NJ USA

SN 1341-8076

EI 1447-0756

J9 J OBSTET GYNAECOL RE

JI J. Obstet. Gynaecol. Res.

PD NOV

PY 2023

VL 49

IS 11

BP 2746

EP 2752

DI 10.1111/jog.15778

EA AUG 2023

PG 7

WC Obstetrics & Gynecology

WE Science Citation Index Expanded (SCI-EXPANDED)

SC Obstetrics & Gynecology

GA X3TF8

UT WOS:001063769900001

PM 37635443

DA 2024-01-18

ER

PT J

AU Anchan, RM

Spies, JB

Zhang, SQ

Wojdyla, D

Bortoletto, P

Terry, K

Disler, E

Milne, A

Gargiulo, A

Petrozza, J

Brook, O

Srouji, S

Morton, CC

Greenberg, J

Wegienka, G

Stewart, EA

Nicholson, WK

Thomas, L

Venable, S

Laughlin-Tommaso, S

Diamond, MP

Maxwell, GL

Marsh, EE

Myers, ER

Vines, AI

Wise, LA

Wallace, K

Jacoby, VL

AF Anchan, Raymond M.

Spies, James B.

Zhang, Shuaiqi

Wojdyla, Daniel

Bortoletto, Pietro

Terry, Kathryn

Disler, Emily

Milne, Ankrish

Gargiulo, Antonio

Petrozza, John

Brook, Olga

Srouji, Serene

Morton, Cynthia C.

Greenberg, James

Wegienka, Ganesa

Stewart, Elizabeth A.

Nicholson, Wanda K.

Thomas, Laine

Venable, Sateria

Laughlin-Tommaso, Shannon

Diamond, Michael P.

Maxwell, G. Larry

Marsh, Erica E.

Myers, Evan R.

Vines, Anissa I.

Wise, Lauren A.

Wallace, Kedra

Jacoby, Vanessa L.

TI Long-term health-related quality of life and symptom severity following

hysterectomy, myomectomy, or uterine artery embolization for the

treatment of symptomatic uterine fibroids

SO AMERICAN JOURNAL OF OBSTETRICS AND GYNECOLOGY

LA English

DT Article

DE hysterectomy; myomectomy; quality of life; uterine artery embolization;

uterine fibroids

ID MANAGEMENT; EPIDEMIOLOGY; LEIOMYOMA; IMPACT; WOMEN; QUESTIONNAIRE;

MORBIDITY; BURDEN

AB BACKGROUND: Few studies have directly compared different surgical procedures for uterine fibroids with respect to long-term health-related quality of life outcomes and symptom improvement.OBJECTIVE: We examined differences in change from baseline to 1-, 2-, and 3-year follow-up in health-related quality of life and symptom severity among patients who underwent abdominal myomectomy, laparoscopic or robotic myomectomy, abdominal hysterectomy, laparoscopic or robotic hysterectomy, or uterine artery embolization.STUDY DESIGN: The COMPARE-UF registry is a multiinstitutional prospective observational cohort study of women undergoing treatment for uterine fibroids. A subset of 1384 women aged 31 to 45 years who underwent either abdominal myomectomy (n=237), laparoscopic myomectomy (n=272), abdominal hysterectomy (n=177), laparoscopic hysterectomy (n=522), or uterine artery embolization (n=176) were included in this analysis. We obtained demographics, fibroid history, and symptoms by questionnaires at enrollment and at 1, 2, and 3 years posttreatment. We used the UFS-QoL (Uterine Fibroid Symptom and Quality of Life) questionnaire to ascertain symptom severity and health-related quality of life scores among participants. To account for potential baseline differences across treatment groups, a propensity score model was used to derive overlap weights and compare total health-related quality of life and symptom severity scores after enrollment with a repeated measures model. For this health-related quality of life tool, a specific minimal clinically important difference has not been determined, but on the basis of previous research, a difference of 10 points was considered as a reasonable estimate. Use of this difference was agreed upon by the Steering Committee at the time when the analysis was planned.RESULTS: At baseline, women undergoing hysterectomy and uterine artery embolization reported the lowest health-related quality of life scores and highest symptom severity scores compared with those undergoing abdominal myomectomy or laparoscopic myomectomy (P<.001). Those undergoing hysterectomy and uterine artery embolization reported the longest duration of fibroid symptoms with a mean of 6.3 years (standard deviation, 6.7; P<.001). The most common fibroid symptoms were menorrhagia (75.3%), bulk symptoms (74.2%), and bloating (73.2%). More than half (54.9%) of participants reported anemia, and 9.4% women reported a history of blood transfusion. Across all modalities, total health-related quality of life and symptom severity score markedly improved from baseline to 1-year with the largest improvement in the laparoscopic hysterectomy group (Uterine Fibroids Symptom and Quality of Life: delta= [+] 49.2; symptom severity: delta= [-] 51.3). Those undergoing abdominal myomectomy, laparoscopic myomectomy, and uterine artery embolization also demonstrated significant improvement in health-related quality of life (delta= [+]43.9, [+]32.9, [+] 40.7, respectively) and symptom severity (delta= [-]41.4, [-] 31.5, [-] 38.5, respectively) at 1 year, and the improvement persisted from baseline for uterine-sparing procedures during second (Uterine Fibroids Symptom and Quality of Life: delta= [+]40.7, [+]37.4, [+]39.3 SS: delta= [-] 38.5, [-] 32.0, [-] 37.7 and third year (Uterine Fibroids Symptom and Quality of Life: delta= [+] 40.9, [+]39.9, [+]41.1 and SS: delta= [-] 33.9, [-]36.5, [-] 33.0, respectively), posttreatment intervals, however with a trend toward decline in degree of improvement from years 1 and 2.

Differences from baseline were greatest for hysterectomy; however, this may reflect the relative importance of bleeding in the Uterine Fibroids Symptom and Quality of Life, rather than clinically meaningful symptom recurrence among women undergoing uterus-sparing treatments.CONCLUSION: All treatment modalities were associated with significant improvements in health-related quality of life and symptom severity reduction 1-year posttreatment. However, abdominal myomectomy, laparoscopic myomectomy and uterine artery embolization indicated a gradual decline in symptom improvement and health-related quality of life by third year after the procedure.

C1 [Anchan, Raymond M.; Terry, Kathryn; Disler, Emily; Milne, Ankrish; Gargiulo, Antonio; Srouji, Serene; Morton, Cynthia C.; Greenberg, James] Harvard Med Sch, Brigham & Womens Hosp, Dept Obstet & Gynecol, Boston, MA 02115 USA.

[Spies, James B.] Georgetown Univ, Sch Med, Dept Radiol, Washington, DC USA.

[Zhang, Shuaiqi; Wojdyla, Daniel; Thomas, Laine] Duke Univ, Sch Med, Duke Clin Res Inst, Durham, NC USA.

[Bortoletto, Pietro] Weill Cornell Med Coll, Ronald O Perelman & Claudia Cohen Ctr Reprod Med, New York, NY USA.

[Petrozza, John] Harvard Med Sch, Dept Obstet & Gynecol, Massachusetts Gen Hosp, Boston, MA USA.

[Brook, Olga] Beth Israel Deaconess Med Ctr, Dept Radiol, Boston, MA USA.

[Morton, Cynthia C.] Harvard Med Sch, Brigham & Womens Hosp, Dept Pathol, Boston, MA USA.

[Morton, Cynthia C.] Broad Inst MIT & Harvard, Cambridge, MA USA.

[Morton, Cynthia C.] Univ Manchester, Manchester Ctr Audiol & Deafness, Sch Hlth Sci, Manchester, England.

[Wegienka, Ganesa] Henry Ford Hlth Syst, Dept Publ Hlth Sci, Detroit, MI USA.

[Stewart, Elizabeth A.; Laughlin-Tommaso, Shannon] Mayo Clin, Dept Obstet & Gynecol, Rochester, MN USA.

[Nicholson, Wanda K.] Univ N Carolina, Ctr Womens Hlth Res, Dept Obstet & Gynecol, Chapel Hill, NC USA.

[Nicholson, Wanda K.] Univ N Carolina, Ctr Hlth Promot & Dis Prevent, Dept Obstet & Gynecol, Chapel Hill, NC USA.

[Thomas, Laine] Duke Univ, Sch Med, Dept Biostat & Bioinformat, Durham, NC USA.

[Venable, Sateria] Fibroid Fdn, Bethesda, MD USA.

[Stewart, Elizabeth A.; Laughlin-Tommaso, Shannon] Mayo Clin, Dept Surg, Rochester, MN USA.

[Diamond, Michael P.] Augusta Univ, Dept Obstet & Gynecol, Augusta, GA USA.

[Maxwell, G. Larry] Inova Fairfax Hosp, Dept Obstet & Gynecol, Falls Church, VA USA.

[Marsh, Erica E.] Univ Michigan, Dept Obstet & Gynecol, Ann Arbor, MI USA.

[Myers, Evan R.] Duke Univ, Sch Med, Dept Obstet & Gynecol, Durham, NC USA.

[Vines, Anissa I.] Univ N Carolina, Gillings Sch Global Publ Hlth, Dept Epidemiol, Chapel Hill, NC USA.

[Wise, Lauren A.] Boston Univ, Sch Publ Hlth, Dept Epidemiol, Boston, MA USA.

[Wallace, Kedra] Univ Mississippi, Med Ctr, Dept Obstet & Gynecol, Jackson, MS USA.

[Jacoby, Vanessa L.] Univ Calif San Francisco, Dept Obstet Gynecol & Reprod Sci, San Francisco, CA USA.

[Anchan, Raymond M.] Yale Sch Publ Hlth, Dept Biostat, New Haven, CT 06510 USA.

C3 Harvard University; Brigham & Women's Hospital; Harvard Medical School;

Georgetown University; Duke University; Cornell University; Weill

Cornell Medicine; Harvard University; Harvard Medical School;

Massachusetts General Hospital; Harvard University; Beth Israel

Deaconess Medical Center; Harvard University; Brigham & Women's

Hospital; Harvard Medical School; Harvard University; Massachusetts

Institute of Technology (MIT); Broad Institute; University of

Manchester; Henry Ford Health System; Henry Ford Hospital; Mayo Clinic;

University of North Carolina; University of North Carolina Chapel Hill;

University of North Carolina; University of North Carolina Chapel Hill;

Duke University; Mayo Clinic; University System of Georgia; Augusta

University; Inova Fairfax Hospital; University of Michigan System;

University of Michigan; Duke University; University of North Carolina;

University of North Carolina Chapel Hill; Boston University; University

of Mississippi; University of Mississippi Medical Center; University of

California System; University of California San Francisco; Yale

University

RP Anchan, RM (corresponding author), Harvard Med Sch, Brigham & Womens Hosp, Dept Obstet & Gynecol, Boston, MA 02115 USA.; Anchan, RM (corresponding author), Yale Sch Publ Hlth, Dept Biostat, New Haven, CT 06510 USA.

EM ranchan@bwh.harvard.edu

FU Agency for Healthcare Research and Quality (AHRQ) [P50HS023418];

Patient-Centered Outcomes Research Institute (PCORI) under mem-orandum

of understanding [2013-001]; US Depart-ment of Health and Human Services

FX This study was supported by grants from the Agency for Healthcare

Research and Quality (AHRQ) (number P50HS023418) with funding provided

by the Patient-Centered Outcomes Research Institute (PCORI) under

mem-orandum of understanding (number 2013-001) . The content of this

manuscript is solely the responsibility of the authors, and readers

should not interpret any statement in this product as an official

position or the views of AHRQ, the US Depart-ment of Health and Human

Services, or PCORI.

NR 48

TC 2

Z9 2

U1 3

U2 3

PU MOSBY-ELSEVIER

PI NEW YORK

PA 360 PARK AVENUE SOUTH, NEW YORK, NY 10010-1710 USA

SN 0002-9378

EI 1097-6868

J9 AM J OBSTET GYNECOL

JI Am. J. Obstet. Gynecol.

PD SEP

PY 2023

VL 229

IS 3

DI 10.1016/j.ajog.2023.05.020

EA AUG 2023

PG 17

WC Obstetrics & Gynecology

WE Science Citation Index Expanded (SCI-EXPANDED)

SC Obstetrics & Gynecology

GA S3GB6

UT WOS:001070076200001

PM 37244458

DA 2024-01-18

ER

PT J

AU Van Trappen, P

AF Van Trappen, Philippe

TI Robotic para-aortic sentinel lymph node mapping in endometrial,

cervical, and ovarian cancer

SO BEST PRACTICE & RESEARCH CLINICAL OBSTETRICS & GYNAECOLOGY

LA English

DT Article

DE Cervical cancer; Endometrial cancer; Ovarian cancer; Para-aortic;

Sentinel; Staging

ID INDOCYANINE GREEN; BIOPSY; STANDARDIZATION; GUIDELINES; MANAGEMENT;

ALGORITHM; STEP

AB The concept of pelvic sentinel lymph node mapping has been well investigated in endometrial and cervical cancer. A variety of tracers have been used including blue dye, technetium-99-m (Tc-99 m), and fluorescent tracer indocyanine green. Pelvic sentinel lymph node mapping has shown its safety, efficacy, and diagnostic accuracy, with high sensitivity and negative predictive value of more than 90%, in retrospective cohort studies as well as in prospective trials for robotic surgery. The concept of pelvic sentinel lymph node biopsy has been incorporated in several international guidelines in early-stage endometrial cancer and a subgroup of early-stage cervical cancer, although survival data are still needed to confirm its standard use. The application of para-aortic sentinel lymph node mapping is still in a development phase, but its detection rate and diagnostic accuracy seem to be promising in initial studies. Here, an overview is given of the recent developments in the different methodologies used for identifying para-aortic sentinel lymph nodes in endometrial, cervical, and ovarian cancer.& COPY; 2023 Elsevier Ltd. All rights reserved.

C1 [Van Trappen, Philippe] AZ Sint Jan Bruges Ostend AV, Dept Gynecol & Gynecol Oncol, Ruddershove 10, B-8000 Brugge, Belgium.

RP Van Trappen, P (corresponding author), AZ Sint Jan Bruges Ostend AV, Dept Gynecol & Gynecol Oncol, Ruddershove 10, B-8000 Brugge, Belgium.

EM philippe.vantrappen@azsintjan.be

OI Van Trappen, Philippe/0000-0002-9784-7974

NR 46

TC 0

Z9 0

U1 0

U2 0

PU ELSEVIER SCI LTD

PI OXFORD

PA THE BOULEVARD, LANGFORD LANE, KIDLINGTON, OXFORD OX5 1GB, OXON, ENGLAND

SN 1521-6934

EI 1532-1932

J9 BEST PRACT RES CL OB

JI Best Pract. Res. Clin. Obstet. Gynaecol.

PD AUG

PY 2023

VL 90

AR 102402

DI 10.1016/j.bpobgyn.2023.102402

EA AUG 2023

PG 10

WC Obstetrics & Gynecology

WE Science Citation Index Expanded (SCI-EXPANDED)

SC Obstetrics & Gynecology

GA R5PN9

UT WOS:001064872200001

PM 37619486

DA 2024-01-18

ER

PT J

AU Yu, HX

Zhang, SF

Zhang, WD

Tang, HM

Chen, Y

Dong, ZY

Qin, ZY

Liu, JL

Wang, HH

Bao, MY

Wei, WW

Shi, RX

Xia, BR

Chen, JM

AF Yu, Hongxia

Zhang, Shoufeng

Zhang, Wendi

Tang, Huimin

Chen, Yao

Dong, Zhiyong

Qin, Zhenyue

Liu, Junling

Wang, Huihui

Bao, Mingyue

Wei, Weiwei

Shi, Ruxia

Xia, Bairong

Chen, Jiming

TI Conservative surgical treatment of uterine fibroids in women of

childbearing age

SO EUROPEAN JOURNAL OF GYNAECOLOGICAL ONCOLOGY

LA English

DT Review

DE Uterine fibroids; Myomectomy; Uterine artery embolization;

High-Intensity focused ultrasound; Radiofrequency ablation; Pregnancy;

Fertility

ID OUTCOMES; SURGERY

AB Uterine fibroids are benign gynecologic tumors, and women aged between 30 to 50 years are known to have a high incidence of uterine fibroids. A growing number of pharmacotherapies and minimally invasive organ-preserving treatments have been designed and conducted over the past few years. However, there has not been any therapeutic drugs exhibiting an ideal therapeutic effect and low recurrence rate, such that the surgical treatment continues to be primarily employed in the actual clinical treatment. In general, surgical treatment has been performed as the organ-and fertility -preserving hysteroscopic or laparoscopic resections of the fibroids. Minimally invasive surgical equipment (e.g., hysteroscopy, traditional porous laparoscopy, trans-umbilical laparoscopy, transvaginal laparoscopy, as well as robot-assisted laparoscopy) has been extensively applied to clinical treatment. Compared with traditional laparotomy, minimally invasive surgical equipment is characterized by minimally invasive surgery, high efficiency and safety. As medical technology has been leaping forward, interventional therapy and radiofrequency ablation can also be employed for treating uterine fibroids. In accordance with the research progress worldwide, the current situation, limitations, and advantages of the treatment of uterine fibroids in patients with fertility requirements are reviewed in this study.

C1 [Yu, Hongxia; Zhang, Shoufeng; Zhang, Wendi; Qin, Zhenyue; Wang, Huihui; Bao, Mingyue] Dalian Med Univ, Dalian 116000, Liaoning, Peoples R China.

[Tang, Huimin; Dong, Zhiyong; Liu, Junling; Wei, Weiwei; Shi, Ruxia; Chen, Jiming] Nanjing Med Univ, Affiliated Changzhou Peoples Hosp 2, Dept Gynecol, Changzhou 213000, Jiangsu, Peoples R China.

[Chen, Yao; Xia, Bairong] Univ Sci & Technol China, Affiliated Hosp USTC 1, Dept Gynecol, Div Life Sci & Med, Hefei 230031, Anhui, Peoples R China.

C3 Dalian Medical University; Nanjing Medical University; Chinese Academy

of Sciences; University of Science & Technology of China, CAS

RP Chen, JM (corresponding author), Nanjing Med Univ, Affiliated Changzhou Peoples Hosp 2, Dept Gynecol, Changzhou 213000, Jiangsu, Peoples R China.; Xia, BR (corresponding author), Univ Sci & Technol China, Affiliated Hosp USTC 1, Dept Gynecol, Div Life Sci & Med, Hefei 230031, Anhui, Peoples R China.

EM xiabairong@ustc.edu.cn; cjming@126.com

RI Yang, Fan/JMA-9594-2023; LU, Li/JFJ-9011-2023; Yang, Fan/JVO-8611-2024;

lin, qing/JTU-4293-2023; Wei, Wei/JVM-8876-2024; Zhang,

Lanyue/JNS-8209-2023; zhang, yan/JGL-8022-2023; Yuan,

Fang/JQV-7426-2023; liu, lin/JFK-3401-2023; yang, li/JGM-1009-2023;

Yang, Min/JPY-3791-2023

OI Wei, Wei/0000-0002-4109-3878;

FU Changzhou High- Level Medical Talents Training Project [2022CZBJ074];

maternal and child health key talent project of Jiangsu Province

[RC202101]; maternal and child health research project of Jiangsu

Province [F202138]; Scientific Research Support Program for Postdoctoral

of Jiangsu Province [2019K064]; Scientific Research Support Program for

"333 Project" of Jiangsu Province [BRA2019161]

FX This work was supported by grants from Changzhou High- Level Medical

Talents Training Project (2022CZBJ074) , the maternal and child health

key talent project of Jiangsu Province (RC202101) , the maternal and

child health research project of Jiangsu Province (F202138) , the

Scientific Research Support Program for Postdoctoral of Jiangsu Province

(2019K064) , and the Scientific Research Support Program for "333

Project" of Jiangsu Province (BRA2019161) .

NR 30

TC 0

Z9 0

U1 4

U2 4

PU MRE PRESS

PI SINGAPORE

PA 14 ROBINSON RD #08-01A FAR EAST FINANCE, SINGAPORE, SINGAPORE

SN 0392-2936

EI 2709-0086

J9 EUR J GYNAECOL ONCOL

JI Eur. J. Gynaecol. Oncol.

PD AUG 15

PY 2023

VL 44

IS 4

BP 1

EP 5

DI 10.22514/ejgo.2023.053

PG 5

WC Oncology; Obstetrics & Gynecology

WE Science Citation Index Expanded (SCI-EXPANDED)

SC Oncology; Obstetrics & Gynecology

GA P5TV6

UT WOS:001051308800001

OA gold

DA 2024-01-18

ER

PT J

AU Gallotta, V

Certelli, C

Oliva, R

Rosati, A

Federico, A

Loverro, M

Lodoli, C

Foschi, N

Lathouras, K

Fagotti, A

Scambia, G

AF Gallotta, Valerio

Certelli, Camilla

Oliva, Riccardo

Rosati, Andrea

Federico, Alex

Loverro, Matteo

Lodoli, Claudio

Foschi, Nazario

Lathouras, Konstantinos

Fagotti, Anna

Scambia, Giovanni

TI Robotic surgery in ovarian cancer

SO BEST PRACTICE & RESEARCH CLINICAL OBSTETRICS & GYNAECOLOGY

LA English

DT Article

DE Ovarian cancer; Robotic surgery; Minimally invasive surgery

ID EARLY-STAGE OVARIAN; SECONDARY CYTOREDUCTIVE SURGERY; INTERVAL DEBULKING

SURGERY; MINIMALLY INVASIVE SURGERY; FERTILITY-SPARING SURGERY;

NEOADJUVANT CHEMOTHERAPY; LAPAROSCOPIC CYTOREDUCTION; CONSERVATIVE

TREATMENT; MANAGEMENT; CARCINOMA

AB Ovarian cancer (OC) represents one of the most lethal cancers in women. The aim of surgical treatment is complete cytoreduction in advanced stages and a surgical staging in early stages. Although the guidelines still suggest laparotomy as the standard approach, in recent years minimally invasive surgery (MIS) has become increasingly popular in the treatment of OC, especially in early stages, because the 5-year relative survival exceeds 90% and the patients' quality of life cannot be overshadowed. However, MIS has been demonstrated to have a role even in advanced stages, in the prediction of optimal cytoreduction, identification patients who may benefit from neoadjuvant chemotherapy, and, more recently, in the interval debulking surgery, as in selected cases of secondary cytoreduction for recurrent ovarian cancer. The aim of this review is to describe the MIS (especially robotic surgery), with its advantages and pitfalls, in the treatment of OC.& COPY; 2023 Published by Elsevier Ltd.

C1 [Gallotta, Valerio; Rosati, Andrea; Federico, Alex; Loverro, Matteo; Fagotti, Anna; Scambia, Giovanni] Fdn Policlin Univ A Gemelli IRCCS, Dept Woman Child & Publ Hlth, Rome, Italy.

[Certelli, Camilla; Oliva, Riccardo; Fagotti, Anna; Scambia, Giovanni] Univ Cattolica Sacro Cuore, Inst Obstet & Gynecol, Rome, Italy.

[Lodoli, Claudio] Fdn Policlin Univ A Gemelli IRCCS, Dept Gen Surg, Rome, Italy.

[Foschi, Nazario] Fdn Policlin Univ Agostino Gemelli IRCCS, Div Urol, Rome, Italy.

[Lathouras, Konstantinos] IASO Gen Hosp, Dept Gynaecol Oncol, Athens, Greece.

[Gallotta, Valerio] Catholic Univ, Dept Obstet & Gynecol, Lgo A Gemelli 8, I-00168 Rome, Italy.

C3 Catholic University of the Sacred Heart; IRCCS Policlinico Gemelli;

Catholic University of the Sacred Heart; IRCCS Policlinico Gemelli;

Catholic University of the Sacred Heart; IRCCS Policlinico Gemelli;

Catholic University of the Sacred Heart; IRCCS Policlinico Gemelli;

Catholic University of the Sacred Heart; IRCCS Policlinico Gemelli

RP Gallotta, V (corresponding author), Catholic Univ, Dept Obstet & Gynecol, Lgo A Gemelli 8, I-00168 Rome, Italy.

EM gallottav@gmail.com

OI foschi, nazario/0000-0003-0464-0788

NR 87

TC 3

Z9 3

U1 0

U2 0

PU ELSEVIER SCI LTD

PI OXFORD

PA THE BOULEVARD, LANGFORD LANE, KIDLINGTON, OXFORD OX5 1GB, OXON, ENGLAND

SN 1521-6934

EI 1532-1932

J9 BEST PRACT RES CL OB

JI Best Pract. Res. Clin. Obstet. Gynaecol.

PD AUG

PY 2023

VL 90

AR 102391

DI 10.1016/j.bpobgyn.2023.102391

EA AUG 2023

PG 15

WC Obstetrics & Gynecology

WE Science Citation Index Expanded (SCI-EXPANDED)

SC Obstetrics & Gynecology

GA Q2DQ8

UT WOS:001055679000001

PM 37573801

OA hybrid

DA 2024-01-18

ER

PT J

AU Imai, K

Suzuki, Y

Hiiragi, K

Hotta, Y

Shigeta, H

AF Imai, Kazuaki

Suzuki, Yukio

Hiiragi, Kazuya

Hotta, Yuichiro

Shigeta, Hiroyuki

TI Comparison of quality of life after robotic-transvaginal natural orifice

transluminal endoscopic surgery and robot-assisted laparoscopic

hysterectomy

SO EUROPEAN JOURNAL OF OBSTETRICS & GYNECOLOGY AND REPRODUCTIVE BIOLOGY

LA English

DT Article

DE complete QOL recovery; Hysterectomy; RALH; Robot-assisted; R-vNOTES

ID SURGICAL OUTCOMES; STATES

AB Objectives: We investigated quality of life (QOL) of patients who underwent total hysterectomy for benign uterine diseases using two surgical approaches: robotic-transvaginal natural orifice transluminal endoscopic surgery (RvNOTES) and robot-assisted laparoscopic hysterectomy (RALH). Study design: This single-center retrospective study was conducted in a tertiary academic setting and included 65 patients who underwent robotic-assisted hysterectomy for benign uterine diseases. Total hysterectomy was performed using R-vNOTES or RALH by the same gynecologist between December 2021 and June 2022. The primary outcome was a comparison of QOL over time and complete QOL recovery (postoperative QOL score/ preoperative QOL score & GE; 1) by postoperative day 28 (POD28) in the R-vNOTES and RALH groups. QOL was examined using EQ-5D-5L in this study. The secondary outcome was a comparison of the surgical outcomes in the R-vNOTES and RALH groups. Results: Complete QOL recovery was achieved by 62.7% in the R-vNOTES group and 7.3% in the RALH group at POD7 (p <.001) and by 100% in the R-vNOTES group and 56.1% in the RALH group at POD28 (p <.001). In a multivariable model, patients who underwent R-vNOTES achieved higher complete QOL recovery at POD 28 [adjusted hazard ratio: 4.03, 95% confidence interval: 2.03-8.04]. There was no significant difference between the R-vNOTES and RALH groups in terms of operating time (p =.07), intraoperative blood loss (p =.35), uterus weight (p =.76), or postoperative complications (p =.71). Conclusion: The R-vNOTES approach for total hysterectomy for benign uterine diseases provided better complete QOL recovery by POD28 compared to the RALH approach. The surgical outcomes for R-vNOTES were equivalent to those for RALH, suggesting that R-vNOTES may provide a safe approach for robot-assisted hysterectomy.

C1 [Imai, Kazuaki; Hiiragi, Kazuya; Hotta, Yuichiro; Shigeta, Hiroyuki] Yokohama Municipal Citizens Hosp, Dept Obstet & Gynecol, Yokohama, Japan.

[Suzuki, Yukio] Columbia Univ, Vagelos Coll Phys & Surg, Dept Obstet & Gynecol, Div Gynecol Oncol, New York, NY 10032 USA.

C3 Columbia University

RP Suzuki, Y (corresponding author), Columbia Univ, Vagelos Coll Phys & Surg, Dept Obstet & Gynecol, Div Gynecol Oncol, New York, NY 10032 USA.

EM yetii@yokohama-cu.ac.jp

NR 21

TC 0

Z9 0

U1 1

U2 1

PU ELSEVIER

PI AMSTERDAM

PA RADARWEG 29, 1043 NX AMSTERDAM, NETHERLANDS

SN 0301-2115

EI 1872-7654

J9 EUR J OBSTET GYN R B

JI Eur. J. Obstet. Gynecol. Reprod. Biol.

PD SEP

PY 2023

VL 288

BP 211

EP 215

DI 10.1016/j.ejogrb.2023.08.003

EA AUG 2023

PG 5

WC Obstetrics & Gynecology; Reproductive Biology

WE Science Citation Index Expanded (SCI-EXPANDED)

SC Obstetrics & Gynecology; Reproductive Biology

GA R1VY7

UT WOS:001062300000001

PM 37572450

DA 2024-01-18

ER

PT J

AU Comba, C

Aslan, E

Vatankulu, B

Tatar, Z

Demir, AA

Demir, O

AF Comba, Cihan

Aslan, Erkan

Vatankulu, Betul

Tatar, Zeynep

Demir, Ali Aslan

Demir, Omer

TI An Extraordinary Location of Sentinel Lymph Nodes in a Patient with

Endometrial Cancer

SO JOURNAL OF MINIMALLY INVASIVE GYNECOLOGY

LA English

DT Article

DE Endometrial cancer; Para-aortic sentinel lymph node; Indocyanine green

ID BIOPSY

AB Objective: To show dissection of sentinel lymph nodes.

Design: A step-by-step demonstration of the technique with narration.

Setting: Endometrial cancer (EC) is the most common gynecologic malignancy worldwide. Sentinel lymph node biopsy with indocyanine green (ICG) has become more widely used and has been featured in recently published guidelines for EC [1]. Minimally invasive approaches with the sentinel lymph node concept (conventional laparoscopy, laparoscopic-assisted vaginal surgeries or robotic) to EC staging have resulted in lower rates of peri- and postoperative complications than conventional staging procedures [2].

Interventions: No video article has been published in the literature about high pelvic, para-aortic sentinel lymph node dissection. An informed consent form was obtained from the patient. An institutional review board approval was not required. A 45 -year-old female with gravidity 0, parity 0, and body mass index of 23.4 kg/m(2) presented with complaints of abnormal uterine bleeding (spotting). Increased endometrial thickness was detected on transvaginal ultrasound (10 mm) in the post-menstrual period. Endometrioid-type endometrial adenocancer with focal squamous differentiation International Federation of Gynecology and Obstetrics grade I was detected on endometrial biopsy. The patient had hepatitis B virus positivity and no other chronic disease. A laparotomic myomectomy had been performed in 2016. Laparoscopic high pelvic, low para-aortic sentinel lymph node dissection with ICG and hysterectomy (without uterine manipulator) + bilateral salpingo-oophorectomy were performed (Supplemental Video 1). The operation time for the procedure was 110 minutes and the estimated blood loss was <20 mL. No major complications occurred during or after the surgery. The patient stayed in the hospital for 1 day. The final pathology result showed an International Federation of Gynecology and Obstetrics grade I, endometrioid-type endometrial adenocancer with focal squamous differentiation, as a 1.5 x 1 cm tumorous mass invading less than one-half of the myometrium. Neither lymphovascular invasion nor sentinel lymph node metastasis was detected. A multicenter, prospective study showed that sentinel lymph node dissection with ICG in clinical stage 1 EC is feasible and has a high degree of diagnostic accuracy in detecting EC metastases. In that study, isolated para-aortic sentinel lymph node was detected in 3 of 340 patients (<1%) [2]. Another study reported the detection rate of isolated para-aortic sentinel lymph node to be 1.1% in patients with intermediate- and high-risk EC [3].

Conclusion: There are in some cases 2 distinct channels emanating from one side, and it is important to follow each and to acknowledge there may be more than one sentinel, one of which is lower in a typical location and one higher as in this case. This video article is the first video demonstration of bilateral isolated high pelvic, para-aortic sentinel lymph node dissection in EC. Journal of Minimally Invasive Gynecology (2023) 30, 613-614. 2023 Published by Elsevier Inc. on behalf of AAGL.

C1 [Comba, Cihan] Istanbul Aydin Univ, VM Med Pk Florya Hosp, Dept Gynecol Oncol, Istanbul, Turkiye.

[Aslan, Erkan] Istanbul Aydin Univ, VM Med Pk Florya Hosp, Dept Obstet & Gynecol, Istanbul, Turkiye.

[Vatankulu, Betul] Istanbul Aydin Univ, VM Med Pk Florya Hosp, Dept Nucl Med, Istanbul, Turkiye.

[Demir, Ali Aslan] Istanbul Aydin Univ, VM Med Pk Florya Hosp, Dept Diagnost Radiol, Istanbul, Turkiye.

[Tatar, Zeynep] Patomer Pathol Lab, Dept Pathol, Istanbul, Turkiye.

[Demir, Omer] Karadeniz Tech Univ, Dept Obstet & Gynecol, Trabzon, Turkiye.

[Comba, Cihan] Istanbul Aydin Univ, VM Med Pk Florya Hosp, Dept Gynecol Oncol, Akasya Sk 4D1, TR-34295 Istanbul, Turkiye.

C3 Istanbul Aydin University; Istanbul Aydin University; Istanbul Aydin

University; Istanbul Aydin University; Karadeniz Technical University;

Istanbul Aydin University

RP Comba, C (corresponding author), Istanbul Aydin Univ, VM Med Pk Florya Hosp, Dept Gynecol Oncol, Akasya Sk 4D1, TR-34295 Istanbul, Turkiye.

EM comba.cihan@yahoo.com.tr

RI Demir, Omer/AAL-9190-2021

OI ASLAN, ERKAN/0000-0002-1300-1884

NR 3

TC 0

Z9 0

U1 2

U2 2

PU ELSEVIER SCIENCE INC

PI NEW YORK

PA STE 800, 230 PARK AVE, NEW YORK, NY 10169 USA

SN 1553-4650

EI 1553-4669

J9 J MINIM INVAS GYN

JI J. Minim. Invasive Gynecol.

PD AUG

PY 2023

VL 30

IS 8

BP 613

EP 614

DI 10.1016/j.jmig.2023.04.012

EA AUG 2023

PG 2

WC Obstetrics & Gynecology

WE Science Citation Index Expanded (SCI-EXPANDED)

SC Obstetrics & Gynecology

GA Q1NR5

UT WOS:001055260600001

PM 37137423

DA 2024-01-18

ER

PT J

AU Arcieri, M

Romeo, P

Vizzielli, G

Restaino, S

Driul, L

Stabile, G

Granese, R

Cianci, S

Ercoli, A

AF Arcieri, Martina

Romeo, Paola

Vizzielli, Giuseppe

Restaino, Stefano

Driul, Lorenza

Stabile, Guglielmo

Granese, Roberta

Cianci, Stefano

Ercoli, Alfredo

TI Robotic Single-Port da Vinci Surgical System (SP1098) in Gynecologic

Surgery: A Systematic Review of Literature

SO CLINICAL AND EXPERIMENTAL OBSTETRICS & GYNECOLOGY

LA English

DT Review

DE minimally invasive surgery; single -port surgery; robotic surgery

ID SITE; HYSTERECTOMY; COMPLICATIONS

AB Background: Recently, new surgical systems less invasive than standard laparoscopy have been developed. Among these, robotic single site surgery is playing a pivotal role. In this field, the da Vinci SP (Single-Port) Surgical System (SP1098) is one of the newest surgical technology that presents innovative characteristics that may lead to better surgical outcomes. Few groups have already published their experience and results with this system in gynecology. Methods: The aim of the present systematic review was to provide a comprehensive overview of the status and applications of da Vinci SP1098 in gynecologic surgery. A systematic review of the literature was performed. Studies were identified until September 2022. Results: Six studies were included, reporting a total of 211 patients. The indication for surgery was both benign and malignant disorders. In terms of operative outcomes, the mean/median docking time varied from 2.1 to 5 min while mean/median operating time from 86.5 to 245 min. There was no conversion to multi-port laparoscopy or laparotomy and no major complications related to SP surgery. Conclusions: In conclusion, the preliminary and limited data available regarding the da Vinci SP1098 Surgical System suggest the technical feasibility and safety for its use in gynecologic surgery, with minimal alteration of the surgical technique.

C1 [Arcieri, Martina; Granese, Roberta] Univ Messina, Dept Biomed Dent Morphol & Funct Imaging Sci, I-98122 Messina, Italy.

[Arcieri, Martina; Vizzielli, Giuseppe; Restaino, Stefano; Driul, Lorenza] Univ Hosp Udine, Dept Maternal & Child Hlth, Obstet & Gynecol Clin, I-33100 Udine, Italy.

[Romeo, Paola; Cianci, Stefano; Ercoli, Alfredo] Univ Messina, Dept Human Pathol Adult & Childhood G Barresi, Unit Gynecol & Obstet, I-98122 Messina, Italy.

[Vizzielli, Giuseppe; Driul, Lorenza] Univ Udine, Dept Med, Obstet & Gynecol Clin, I-33100 Udine, Italy.

[Stabile, Guglielmo] Inst Maternal & Child Hlth IRCCS Burlo Garofolo, Dept Obstet & Gynaecol, I-34137 Trieste, Italy.

C3 University of Messina; University of Udine; University Hospital of

Udine; University of Messina; University of Udine; IRCCS Burlo Garofolo

RP Cianci, S (corresponding author), Univ Messina, Dept Human Pathol Adult & Childhood G Barresi, Unit Gynecol & Obstet, I-98122 Messina, Italy.

EM stefanoc85@hotmail.it

RI Vizzielli, Giuseppe/L-1062-2016

OI Vizzielli, Giuseppe/0000-0002-2424-2691

NR 28

TC 0

Z9 0

U1 1

U2 1

PU IMR PRESS

PI ROBINSON

PA 112 ROBINSON RD, ROBINSON, SINGAPORE

SN 0390-6663

EI 2709-0094

J9 CLIN EXP OBSTET GYN

JI Clin. Exp. Obstet. Gynecol.

PD AUG

PY 2023

VL 50

IS 8

AR 158

DI 10.31083/j.ceog5008158

PG 7

WC Obstetrics & Gynecology

WE Science Citation Index Expanded (SCI-EXPANDED)

SC Obstetrics & Gynecology

GA S1AP3

UT WOS:001068567200014

OA gold

DA 2024-01-18

ER

PT J

AU Hamilton, KM

VanHise, K

Truong, MD

Wright, KN

Siedhoff, MT

AF Hamilton, Kacey M.

VanHise, Katherine

Truong, Mireille D.

Wright, Kelly N.

Siedhoff, Matthew T.

TI Surgical management of endometriosis to optimize fertility

SO CURRENT OPINION IN OBSTETRICS & GYNECOLOGY

LA English

DT Review

DE assisted reproductive technology; deep infiltrating endometriosis;

endometrioma; endometriosis; infertility; laparoscopy; surgery

ID IN-VITRO FERTILIZATION; DEEP INFILTRATIVE ENDOMETRIOSIS; UNEXPLAINED

INFERTILITY; LAPAROSCOPIC TREATMENT; MILD ENDOMETRIOSIS; SURGERY; WOMEN;

IMPACT; INSEMINATION; OUTCOMES

AB Purpose of reviewSurgery is an integral element of treatment for infertility caused by endometriosis. This review summarizes the purported mechanisms of infertility in endometriosis, as well as the impacts of surgery for endometriosis on fertility, including pregnancy achieved spontaneously and with assisted reproductive technology (ART).Recent findingsEndometriosis' effect on fertility is multifactorial. The sequela of increased inflammation resulting from endometriosis causes alterations in ovarian, tubal, and uterine function. Removing or destroying these lesions reduces inflammation. Surgical treatment of both early-stage endometriosis and deeply infiltrating endometriosis improves spontaneous pregnancy rates and ART pregnancy rates. Conventional or robotic laparoscopy is the preferred surgical approach.Endometriosis has detrimental effects on fertility, including negative impacts on oocyte, tubal, and endometrial function. Laparoscopic surgery for endometriosis elevates both spontaneous and ART pregnancy rates above those achieved with expectant management alone. The resection or destruction of endometriosis implants reduces inflammation, which likely improves the multifactorial infertility related to endometriosis. This topic is complex and controversial; more research in the form of high-quality randomized control trials is needed.

C1 [Hamilton, Kacey M.; Truong, Mireille D.; Wright, Kelly N.; Siedhoff, Matthew T.] Cedars Sinai Med Ctr, Div Minimally Invas Gynecol Surg, Los Angeles, CA USA.

[VanHise, Katherine] Cedars Sinai Med Ctr, Dept Obstet & Gynecol, Div Reprod Endocrinol & Infertil, Los Angeles, CA USA.

[Siedhoff, Matthew T.] Cedars Sinai Med Ctr, Dept Obstet & Gynecol, Div Minimally Invas Gynecol Surg, Los Angeles, CA 90048 USA.

C3 Cedars Sinai Medical Center; Cedars Sinai Medical Center; Cedars Sinai

Medical Center

RP Siedhoff, MT (corresponding author), Cedars Sinai Med Ctr, Dept Obstet & Gynecol, Div Minimally Invas Gynecol Surg, Los Angeles, CA 90048 USA.

EM matthew.siedhoff@cshs.org

OI Siedhoff, Matthew/0000-0001-7524-2467; Wright,

Kelly/0000-0003-0321-1535; Truong, Mireille/0000-0002-7848-4151

NR 52

TC 0

Z9 0

U1 6

U2 6

PU LIPPINCOTT WILLIAMS & WILKINS

PI PHILADELPHIA

PA TWO COMMERCE SQ, 2001 MARKET ST, PHILADELPHIA, PA 19103 USA

SN 1040-872X

EI 1473-656X

J9 CURR OPIN OBSTET GYN

JI Curr. Opin. Obstet. Gynecol.

PD AUG

PY 2023

VL 35

IS 4

BP 389

EP 394

DI 10.1097/GCO.0000000000000876

PG 6

WC Obstetrics & Gynecology

WE Science Citation Index Expanded (SCI-EXPANDED)

SC Obstetrics & Gynecology

GA L2OL3

UT WOS:001021702800018

PM 37144586

DA 2024-01-18

ER

PT J

AU Lin, E

Young, R

Shields, J

Smith, K

Chao, L

AF Lin, Emily

Young, Riley

Shields, Jessica

Smith, Katherine

Chao, Lisa

TI Growing pains: strategies for improving ergonomics in minimally invasive

gynecologic surgery

SO CURRENT OPINION IN OBSTETRICS & GYNECOLOGY

LA English

DT Review

DE minimally invasive gynecologic surgery; strain; surgical ergonomics;

work-related musculoskeletal disorders

ID SURGICAL ERGONOMICS; PHYSICAL DISCOMFORT; SYMPTOMS; WORK

AB Purpose of reviewTo evaluate factors contributing to the development of work-related musculoskeletal disorders (WMSDs) and review strategies for mitigating ergonomic strain in minimally invasive gynecologic surgery.Recent findingsFactors associated with increased ergonomic strain and the development of WMSDs include increasing patient body mass index (BMI), smaller surgeon hand size, noninclusive design of instruments and energy devices and improper positioning of surgical equipment. Each type of minimally invasive surgery (laparoscopic, robotic, vaginal) confers its own ergonomic risk to the surgeon. Recommendations have been published regarding optimal ergonomic surgeon and equipment positioning. Intraoperative breaks and stretching are effective in reducing surgeon discomfort. Formal training in ergonomics has not yet been widely implemented, but educational interventions have been effective in reducing surgeon discomfort and can improve surgeon recognition of suboptimal ergonomics.Considering the serious downstream effects of WMSDs on surgeons, it is imperative to implement strategies for WMSD prevention. Optimal positioning of the surgeons and operative equipment should be routine. Intraoperative breaks and stretching should be incorporated during procedures and between every case. Formal education in ergonomics should be provided to surgeons and trainees. Additionally, more inclusive instrument design by industry partners should be prioritized.

C1 [Lin, Emily; Young, Riley; Shields, Jessica; Smith, Katherine; Chao, Lisa] Univ Texas Southwestern Med Ctr, Dept Obstet & Gynecol, Div Gynecol, Dallas, TX USA.

[Lin, Emily] 5323 Harry Hines Blvd, G6 234B, Dallas, TX 75390 USA.

C3 University of Texas System; University of Texas Southwestern Medical

Center Dallas

RP Lin, E (corresponding author), 5323 Harry Hines Blvd, G6 234B, Dallas, TX 75390 USA.

EM emily.lin@utsouthwestern.edu

NR 46

TC 0

Z9 0

U1 1

U2 1

PU LIPPINCOTT WILLIAMS & WILKINS

PI PHILADELPHIA

PA TWO COMMERCE SQ, 2001 MARKET ST, PHILADELPHIA, PA 19103 USA

SN 1040-872X

EI 1473-656X

J9 CURR OPIN OBSTET GYN

JI Curr. Opin. Obstet. Gynecol.

PD AUG

PY 2023

VL 35

IS 4

BP 361

EP 367

DI 10.1097/GCO.0000000000000875

PG 7

WC Obstetrics & Gynecology

WE Science Citation Index Expanded (SCI-EXPANDED)

SC Obstetrics & Gynecology

GA L2OL3

UT WOS:001021702800014

PM 37144567

DA 2024-01-18

ER

PT J

AU Iida, Y

Komatsu, H

Kudoh, A

Azuma, Y

Sato, S

Harada, T

Taniguchi, F

AF Iida, Yuki

Komatsu, Hiroaki

Kudoh, Akiko

Azuma, Yukihiro

Sato, Shinya

Harada, Tasuku

Taniguchi, Fuminori

TI The learning curve of introduced robotic-assisted hysterectomy versus

skilled laparoscopic hysterectomy for benign gynecologic diseases

SO JOURNAL OF OBSTETRICS AND GYNAECOLOGY RESEARCH

LA English

DT Article

DE hysterectomy; laparoscopy; robotic surgical procedures

AB Aim This study aimed to compare introduced robotic-assisted hysterectomy (RAH) and skilled total laparoscopic hysterectomy (TLH) for the treatment of benign gynecological diseases.Methods Patients who underwent RAH or TLH by two surgeons at the Tottori University Hospital between January 2018 and May 2022 were included in this retrospective study. Inclusion criteria were patients with 100-300 g of uterine weight. The exclusion criteria were patients with stage IV endometriosis. Mean operative time and learning curve were compared among the first-half RAH, second-half RAH, and TLH groups.Results There were 40 eligible cases (first-half RAH: 20 cases, second-half RAH: 20 cases) in the RAH group and 44 cases in the TLH group. The total operative time (TOT) of the second half of RAH was significantly shorter than that of the first half of RAH (p = 0.021) and was comparable to that of the TLH group. The operative time (OT) of the second half of RAH was shorter than that of TLH (p = 0.023). The preparation time of TLH was shorter than that of the RAH group (p < 0.01). The learning curve of the TOT in RAH crossed that of TLH on the 31st case of RAH. In contrast, both curves of the OT crossed on the 11th case of RAH.Conclusion The TOT of the introduced RAH was equivalent to that of skilled TLH in approximately 30 cases since the first RAH. Furthermore, the OT of RAH was comparable to that of TLH in approximately 10 cases of surgery since the first RAH.

C1 [Iida, Yuki; Komatsu, Hiroaki; Kudoh, Akiko; Azuma, Yukihiro; Sato, Shinya; Harada, Tasuku; Taniguchi, Fuminori] Tottori Univ, Fac Med, Dept Obstet & Gynecol, 36-1 Nishicho, Yonago, Tottori 6838504, Japan.

C3 Tottori University

RP Komatsu, H (corresponding author), Tottori Univ, Fac Med, Dept Obstet & Gynecol, 36-1 Nishicho, Yonago, Tottori 6838504, Japan.

EM komatsu.h.med@gmail.com

OI Komatsu, Hiroaki/0000-0002-4507-6848

NR 20

TC 0

Z9 0

U1 0

U2 0

PU WILEY

PI HOBOKEN

PA 111 RIVER ST, HOBOKEN 07030-5774, NJ USA

SN 1341-8076

EI 1447-0756

J9 J OBSTET GYNAECOL RE

JI J. Obstet. Gynaecol. Res.

PD OCT

PY 2023

VL 49

IS 10

BP 2494

EP 2500

DI 10.1111/jog.15741

EA JUL 2023

PG 7

WC Obstetrics & Gynecology

WE Science Citation Index Expanded (SCI-EXPANDED)

SC Obstetrics & Gynecology

GA T9KN3

UT WOS:001036948800001

PM 37493096

DA 2024-01-18

ER

PT J

AU Falus, N

Lazarou, G

Gabriel, I

Sabatino, N

Grigorescu, B

AF Falus, Nicole

Lazarou, George

Gabriel, Iwona

Sabatino, Nicholas

Grigorescu, Bogdan

TI Contained specimen morcellation during robotics-assisted laparoscopic

supracervical hysterectomy for pelvic organ prolapse

SO INTERNATIONAL UROGYNECOLOGY JOURNAL

LA English

DT Article; Early Access

DE Pelvic organ prolapse; Robotically assisted laparoscopic hysterectomy;

POP repair; Manual morcellation; Contained morcellation

ID PREVALENCE; WOMEN; SACROCOLPOPEXY

AB Introduction and hypothesisRobotics-assisted laparoscopic supracervical hysterectomy (RALSH) with concomitant apical robotics-assisted POP repair provides advantages of minimally invasive procedures; however specimen removal without intraperitoneal spillage of potential pathology remains challenging. The primary aim of our study is to determine the factors affecting contained manual morcellation (CMM) of specimens during RALSH for POP surgery. The secondary aim of the study is to report complications associated with CMM and on specimen pathology.MethodsA total of 67 sequential patients underwent RALSH with concomitant robotics-assisted sacrocolpopexy or uterosacral vaginal suspension. Factors analyzed to affect CMM were specimen weight, length of skin and fascia incisions, patient age, body mass index (BMI), and estimated blood loss (EBL).ResultsMedian CMM time was 11 min (1 to 46) and specimen weight 62 g (19 to 711). Median patient age was 56 years (36 to 83), and patient BMI was 28 (18 to 44). Median EBL was 50 ml (10 to 150). Median skin and fascial incision lengths were 3 cm (1.5 to 7), and 3.5 cm (1.5 to 8). CMM time was significantly dependent on specimen weight (p < 0.0001) and length of rectus fascia incision (p < 0.0126). There was no gross tissue spillage or bag ruptures. Uterine pathology revealed normal tissue (26%), leiomyoma (47%), adenomyosis (49%), and endometriosis (14%). 4.5% of specimens had evidence of microscopic neoplasm, and 5 years after surgery patients were cancer free.ConclusionContained manual extraction of the uterus and/or adnexae at the time of RALSH for POP surgery is a viable, safe, and efficient method of specimen removal.

C1 [Falus, Nicole] NYIT Coll Osteopath Med, Old Westbury, NY USA.

[Lazarou, George; Gabriel, Iwona; Grigorescu, Bogdan] NYU Long Isl, Sch Med, Dept Obstet & Gynecol, Div Female Pelv Med & Reconstruct Surg, 259 1st St, Mineola, NY 11501 USA.

[Sabatino, Nicholas] NYU Long Isl, Sch Med, Dept Obstet & Gynecol, Mineola, NY USA.

C3 New York Institute Technology

RP Grigorescu, B (corresponding author), NYU Long Isl, Sch Med, Dept Obstet & Gynecol, Div Female Pelv Med & Reconstruct Surg, 259 1st St, Mineola, NY 11501 USA.

EM bogdan.grigorescu@nyulangone.org

OI Gabriel, Iwona/0000-0002-1910-7030

NR 30

TC 0

Z9 0

U1 0

U2 0

PU SPRINGER LONDON LTD

PI LONDON

PA 236 GRAYS INN RD, 6TH FLOOR, LONDON WC1X 8HL, ENGLAND

SN 0937-3462

EI 1433-3023

J9 INT UROGYNECOL J

JI Int. Urogynecol. J.

PD 2023 JUL 25

PY 2023

DI 10.1007/s00192-023-05586-2

EA JUL 2023

PG 7

WC Obstetrics & Gynecology; Urology & Nephrology

WE Science Citation Index Expanded (SCI-EXPANDED)

SC Obstetrics & Gynecology; Urology & Nephrology

GA N2QH6

UT WOS:001035515500001

PM 37490062

DA 2024-01-18

ER

PT J

AU De Nagy, J

Youssef, Y

Moawad, G

AF De Nagy, Joseph

Youssef, Youssef

Moawad, Gaby

TI Strategies and factors to maximize cost- effectiveness of robotic

surgery in benign gynecological disease

SO BEST PRACTICE & RESEARCH CLINICAL OBSTETRICS & GYNAECOLOGY

LA English

DT Article

DE Robotic gynecologic surgery; Cost; Minimally invasive surgery;

Hysterectomy

ID LAPAROSCOPIC HYSTERECTOMY; CLINICAL-OUTCOMES; VOLUME; SITE

AB Operating room procedures account for half of the gross hospital cost in the United States per annum. Hysterectomy is the eighth most common surgery nationally, with more than 300,000 cases every year. Since the introduction of robotic surgery in benign gynecology, concern has been raised regarding the increased cost without significant improvements in outcomes or practice. Sur-geon volume, complication rates, length of hospital stay, and selected intraoperative instrumentation are all factors that have a direct effect on cost in robotic surgery. Cost is indirectly influenced by the OR team workflow, postoperative processes to expedite discharge, and converting surgery to the ambulatory setting. More research is needed to develop evidence-based practices for cost containment in robotic surgery.& COPY; 2023 Published by Elsevier Ltd.

C1 [De Nagy, Joseph] Loma Linda Univ, Dept Obstet & Gynecol, Adventist Hlth White Mem, Loma Linda, CA USA.

[Youssef, Youssef] Michigan State Univ, Hurley Med Ctr, Dept Obstet & Gynecol, Coll Human Med, Flint, MI USA.

[Moawad, Gaby] George Washington Univ, Dept Obstet & Gynecol, Washington, DC USA.

[Moawad, Gaby] Ctr Endometriosis & Adv Pelv Surg, Washington, DC USA.

[Moawad, Gaby] 2720 S Arlington Mill Dr, Arlington, VA 22206 USA.

C3 Loma Linda University; Michigan State University; Michigan State

University College of Human Medicine; George Washington University

RP Moawad, G (corresponding author), 2720 S Arlington Mill Dr, Arlington, VA 22206 USA.

EM gnmoawad@gmail.com

OI Youssef, Youssef/0000-0002-4339-333X

NR 37

TC 1

Z9 1

U1 2

U2 2

PU ELSEVIER SCI LTD

PI OXFORD

PA THE BOULEVARD, LANGFORD LANE, KIDLINGTON, OXFORD OX5 1GB, OXON, ENGLAND

SN 1521-6934

EI 1532-1932

J9 BEST PRACT RES CL OB

JI Best Pract. Res. Clin. Obstet. Gynaecol.

PD AUG

PY 2023

VL 90

AR 102380

DI 10.1016/j.bpobgyn.2023.102380

EA JUL 2023

PG 7

WC Obstetrics & Gynecology

WE Science Citation Index Expanded (SCI-EXPANDED)

SC Obstetrics & Gynecology

GA P1LG5

UT WOS:001048319200001

PM 37481892

DA 2024-01-18

ER

PT J

AU Daykan, Y

Farinha, R

Schraffordt, SE

Mottrie, A

O'Reilly, BA

AF Daykan, Yair

Farinha, Rui

Schraffordt, Steven E.

Mottrie, Alexander

O'Reilly, Barry A.

TI A cost-effective model for training in Robot-Assisted Sacrocolpopexy

SO INTERNATIONAL UROGYNECOLOGY JOURNAL

LA English

DT Article; Early Access

DE Robotic assisted surgery; Sacrocolpopexy; Hysteropexy; Pelvic floor

repair

ID SURGERY

AB BackgroundThe number of robotically assisted sacrocolpopexy procedures are increasing; therefore, experienced clinicians are needed. Simulation-based cadaver models are challenging in aspects of cost and availability. Therefore, we need to look at alternative and more cost-effective models.ObjectiveThe objective of this video was to design a new surgical model for the training of robotic-assisted sacrocolpopexy, which is affordable and accessible.MethodsWe used a whole chicken model to simulate the female pelvic floor. We used Medtronic's Hugo & TRADE; RAS system as the robotic console in that procedure. A vaginal cuff was prepared from the proventriculus (stomach), and a Y shaped mesh was secured to the ischium to simulate the sacrocolpopexy procedure.ConclusionThis model is easily constructed and in our view is cost-effective. We have demonstrated a new valuable education tool that can serve as a practical simulation model to teach the sacrocolpopexy procedure and to improve trainees' skills. A larger cohort study size is essential to demonstrate the learning curve among young trainees using this simulation model.

C1 [Daykan, Yair; O'Reilly, Barry A.] Cork Univ Matern Hosp, Dept Urogynaecol, Cork, Ireland.

[Daykan, Yair] Meir Med Ctr, Dept Obstet & Gynecol, Kefar Sava, Israel.

[Daykan, Yair] Tel Aviv Univ, Sackler Sch Med, Tel Aviv, Israel.

[Farinha, Rui; Mottrie, Alexander] OLV, Dept Urol, Aalst, Belgium.

[Farinha, Rui; Mottrie, Alexander] ORSI Acad, Melle, Belgium.

[Schraffordt, Steven E.] Meander Med Ctr, Dept Urogynecol, Amersfoort, Netherlands.

C3 Tel Aviv University; Sackler Faculty of Medicine; Tel Aviv University;

Sackler Faculty of Medicine; Meander Medisch Centrum

RP Daykan, Y (corresponding author), Cork Univ Matern Hosp, Dept Urogynaecol, Cork, Ireland.; Daykan, Y (corresponding author), Meir Med Ctr, Dept Obstet & Gynecol, Kefar Sava, Israel.; Daykan, Y (corresponding author), Tel Aviv Univ, Sackler Sch Med, Tel Aviv, Israel.

EM yair.dykan@gmail.com

RI Daykan, Yair/AAF-2867-2021

OI Daykan, Yair/0000-0002-0447-2414

NR 14

TC 0

Z9 0

U1 1

U2 1

PU SPRINGER LONDON LTD

PI LONDON

PA 236 GRAYS INN RD, 6TH FLOOR, LONDON WC1X 8HL, ENGLAND

SN 0937-3462

EI 1433-3023

J9 INT UROGYNECOL J

JI Int. Urogynecol. J.

PD 2023 JUL 15

PY 2023

DI 10.1007/s00192-023-05604-3

EA JUL 2023

PG 4

WC Obstetrics & Gynecology; Urology & Nephrology

WE Science Citation Index Expanded (SCI-EXPANDED)

SC Obstetrics & Gynecology; Urology & Nephrology

GA M3GN0

UT WOS:001029096900001

PM 37453031

DA 2024-01-18

ER

PT J

AU Chen, CCG

Malpani, A

Waldram, MM

Romanczyk, C

Tanner, EJ

Fader, AN

Scheib, SA

Hager, GD

Vedula, SS

AF Chen, Chi Chiung Grace

Malpani, Anand

Waldram, Madeleine M.

Romanczyk, Caitlin

Tanner, Edward J.

Fader, Amanda N.

Scheib, Stacey A.

Hager, Gregory D.

Vedula, S. Swaroop

TI Effect of pre-operative warm-up on trainee intraoperative performance

during robot-assisted hysterectomy: a randomized controlled trial

SO INTERNATIONAL UROGYNECOLOGY JOURNAL

LA English

DT Article; Early Access

DE Randomized controlled trial; Robotic hysterectomy; Simulation; Warm-up

ID LAPAROSCOPIC HYSTERECTOMY; SURGICAL PERFORMANCE; CONSTRUCT-VALIDATION;

MENTAL PRACTICE; SKILLS; SURGERY; SIMULATOR; METAANALYSIS; ACQUISITION;

ENVIRONMENT

AB Introduction and hypothesisThe objective was to study the effect of immediate pre-operative warm-up using virtual reality simulation on intraoperative robot-assisted laparoscopic hysterectomy (RALH) performance by gynecology trainees (residents and fellows).MethodsWe randomized the first, non-emergent RALH of the day that involved trainees warming up or not warming up. For cases assigned to warm-up, trainees performed a set of exercises on the da Vinci Skills Simulator immediately before the procedure. The supervising attending surgeon, who was not informed whether or not the trainee was assigned to warm-up, assessed the trainee's performance using the Objective Structured Assessment for Technical Skill (OSATS) and the Global Evaluative Assessment of Robotic Skills (GEARS) immediately after each surgery.ResultsWe randomized 66 cases and analyzed 58 cases (30 warm-up, 28 no warm-up), which involved 21 trainees. Attending surgeons rated trainees similarly irrespective of warm-up randomization with mean (SD) OSATS composite scores of 22.6 (4.3; warm-up) vs 21.8 (3.4; no warm-up) and mean GEARS composite scores of 19.2 (3.8; warm-up) vs 18.8 (3.1; no warm-up). The difference in composite scores between warm-up and no warm-up was 0.34 (95% CI: -1.44, 2.13), and 0.34 (95% CI: -1.22, 1.90) for OSATS and GEARS respectively. Also, we did not observe any significant differences in each of the component/subscale scores within OSATS and GEARS between cases assigned to warm-up and no warm-up.ConclusionPerforming a brief virtual reality-based warm-up before RALH did not significantly improve the intraoperative performance of the trainees.

C1 [Chen, Chi Chiung Grace; Tanner, Edward J.; Fader, Amanda N.] Johns Hopkins Univ, Dept Gynecol & Obstet, Baltimore, MD 21218 USA.

[Malpani, Anand; Hager, Gregory D.; Vedula, S. Swaroop] Johns Hopkins Univ, Malone Ctr Engn Healthcare, Baltimore, MD USA.

[Waldram, Madeleine M.; Romanczyk, Caitlin] Johns Hopkins Univ, Sch Med, Baltimore, MD USA.

[Scheib, Stacey A.] Louisiana State Univ Hlth Sci Ctr, Dept Gynecol & Obstet, New Orleans, LA USA.

C3 Johns Hopkins University; Johns Hopkins University; Johns Hopkins

University; Louisiana State University System; Louisiana State

University Health Sciences Center New Orleans

RP Chen, CCG (corresponding author), Johns Hopkins Univ, Dept Gynecol & Obstet, Baltimore, MD 21218 USA.

EM cchen127@jhmi.edu

OI chen, chi chiung grace/0000-0003-3402-7714

NR 35

TC 0

Z9 0

U1 0

U2 0

PU SPRINGER LONDON LTD

PI LONDON

PA 236 GRAYS INN RD, 6TH FLOOR, LONDON WC1X 8HL, ENGLAND

SN 0937-3462

EI 1433-3023

J9 INT UROGYNECOL J

JI Int. Urogynecol. J.

PD 2023 JUL 14

PY 2023

DI 10.1007/s00192-023-05595-1

EA JUL 2023

PG 8

WC Obstetrics & Gynecology; Urology & Nephrology

WE Science Citation Index Expanded (SCI-EXPANDED)

SC Obstetrics & Gynecology; Urology & Nephrology

GA M2GT0

UT WOS:001028424300001

PM 37449987

DA 2024-01-18

ER

PT J

AU Bachi, A

Bille, A

Khazali, S

AF Bachi, Averyl

Bille, Andrea

Khazali, Shaheen

TI The Combined Robotic-assisted Laparoscopic and Thoracic Approach in the

Management of Diaphragmatic, Pleural, and Pericardial Endometriosis

SO JOURNAL OF MINIMALLY INVASIVE GYNECOLOGY

LA English

DT Article

DE Endometriosis; Diaphragmatic endometriosis; Pericardial endometriosis;

Robotic-assisted laparoscopy; Robotic-assisted thoracic surgery

AB Study Objective: To demonstrate the advantages of a combined robotic-assisted laparoscopic and thoracic approach in the management of extensive diaphragmatic, pleural, and pericardial endometriosis.Design: A video article demonstrating excision of endometriosis from pericardium, diaphragm, and pleura.Setting: Thoracic endometriosis is the most common site of extrapelvic endometriosis [1]. Surgical treatment aims to excise all visible disease to relief symptoms and prevent recurrence [2-4]. Interventions: A 41-year-old lady with cyclical shoulder tip and chest pain and known extensive diaphragmatic endometri-osis was referred to our center. The procedure was done jointly by a gynecologist and a thoracic surgeon experienced in robotic-assisted endometriosis excision (Supplemental Video 1). Robotic-assisted laparoscopy revealed extensive full-thick-ness diaphragmatic endometriosis and a full-thickness pericardial nodule. Pericardial endometriosis excision was performed and a 1 cm defect was left open in the pericardium. Multiple diaphragmatic endometriotic nodules were excised and pleural cavity was entered (Image 2). On robotic-assisted thoracic surgery, further deep endometriotic lesions were detected and excised from the posterior aspect of the diaphragm. These lesions were not identified abdominally despite complete division of falciform ligament, full mobilization of the liver, and the use of a 30-degree scope. Superficial endometriotic lesions on parietal pleura were also detected (Image 3) and excised. The defects on the diaphragm were closed (Image 4). Chest and abdominal drains were left in situ. The patient was discharged on day 4. Conclusion: The combined robotic-assisted laparoscopic and thoracic approach is indicated in selected cases and allows full exploration of the thoracic cavity and both sides of the diaphragm, thus preventing incomplete excision of the disease. Robotic surgery also allows smooth dual-surgeon teamwork. Journal of Minimally Invasive Gynecology (2023) 30, 533 -534. Crown Copyright & COPY; 2023. Published by Elsevier Inc. on behalf of AAGL. All rights reserved.

C1 [Bachi, Averyl; Bille, Andrea; Khazali, Shaheen] HCA Lister Hosp, Ctr Endometriosis & Minimally Invas Gynecol CEMIG, Chelsea Bridge Rd, London SW1W 8RH, England.

[Bille, Andrea] Guys & St Thomas NHS Fdn Trust, Dept Thorac Surg, London, England.

C3 Guy's & St Thomas' NHS Foundation Trust

RP Khazali, S (corresponding author), HCA Lister Hosp, Ctr Endometriosis & Minimally Invas Gynecol CEMIG, Chelsea Bridge Rd, London SW1W 8RH, England.

EM s.khazali@me.com

NR 4

TC 2

Z9 2

U1 0

U2 0

PU ELSEVIER SCIENCE INC

PI NEW YORK

PA STE 800, 230 PARK AVE, NEW YORK, NY 10169 USA

SN 1553-4650

EI 1553-4669

J9 J MINIM INVAS GYN

JI J. Minim. Invasive Gynecol.

PD JUL

PY 2023

VL 30

IS 7

BP 533

EP 534

DI 10.1016/j.jmig.2023.03.026

EA JUL 2023

PG 2

WC Obstetrics & Gynecology

WE Science Citation Index Expanded (SCI-EXPANDED)

SC Obstetrics & Gynecology

GA N6DC4

UT WOS:001037885300001

PM 37031860

DA 2024-01-18

ER

PT J

AU Polin, M

Boone, R

Lim, F

Advincula, AP

May, B

Hur, C

Hur, HC

AF Polin, Melanie

Boone, Ryan

Lim, Francesca

Advincula, Arnold P.

May, Benjamin

Hur, Chin

Hur, Hye-Chun

TI Hysterectomy Trends and Risk of Vaginal Cuff Dehiscence: An Update by

Mode of Surgery

SO JOURNAL OF MINIMALLY INVASIVE GYNECOLOGY

LA English

DT Article

DE Minimally invasive hysterectomy; Robotic hysterectomy; Laparoscopic

hysterectomy; Hysterectomy; Surgical complica-tions; Vaginal cuff

dehiscence

ID LAPAROSCOPIC HYSTERECTOMY; EVISCERATION

AB Study Objective: To analyze hysterectomy trends and vaginal cuff dehiscence (VCD) rates by mode of surgery at a tertiary care medical center and to describe characteristics of VCD cases. Design: Observational retrospective cohort study. Setting: Large academic hospital and affiliated community hospital. Patients: 4722 patients who underwent hysterectomy at Columbia University Irving Medical Center between January 2010 and August 2021. Interventions: Current Procedural Terminology and International Classification of Diseases codes identified hysterectomies and VCD cases. Hysterectomy trends and VCD rates were calculated by mode of surgery. Relative risks of VCD for each mode were compared with total abdominal hysterectomy (TAH). Clinical characteristics of VCDs were reviewed. Measurements and Main Results: There were 4059 total hysterectomies. Laparoscopic hysterectomies, including total laparoscopic hysterectomies (TLHs), laparoscopic-assisted vaginal hysterectomies, and robot-assisted TLHs (RA-TLHs), increased from 41.9% in 2010 to 65.9% in 2021 (p <.001). RA-TLH increased from 5.7% in 2010 to 40.2% in 2021. Supracervical hysterectomies followed similar trends and were excluded from VCD analysis. There were 15 VCDs (overall rate 0.37%). VCD was highest after RA-TLH (0.66%), followed by TLH (0.32%) and TAH (0.27%), with no VCDs after laparoscopic-assisted vaginal hysterectomy or total vaginal hysterectomy. Compared with TAH, the relative risk for VCD after RA-TLH was 2.44 (95% confidence interval 0.66-9.00) and after TLH was 1.18 (95% confidence interval 0.24-5.83), which were not statistically significant. The mean time to dehiscence was 39 days (range 8-145 days). The most common trigger event was coitus (41%). Conclusion: VCD rates were low (<1%) for all modes of hysterectomy, and rates after robotic and laparoscopic hysterectomy were much lower than previously reported. Although VCD rates trended higher after robotic and laparoscopic hysterectomy compared with abdominal hysterectomy, the difference was not significant. It is difficult to determine whether this finding represents true lack of difference vs a lack of power to detect a significant difference given the rarity of VCD. Journal of Minimally Invasive Gynecology (2023) 30, 562-568. & COPY; 2023 AAGL. All rights reserved.

C1 [Polin, Melanie; Boone, Ryan; Advincula, Arnold P.; Hur, Hye-Chun] Columbia Univ, Irving Med Ctr, Dept Obstet & Gynecol, New York, NY USA.

[Lim, Francesca; Hur, Chin] Columbia Univ, Irving Med Ctr, Dept Med, New York, NY USA.

[May, Benjamin; Hur, Chin] Columbia Univ, Herbert Irving Comprehens Canc Ctr, Irving Med Ctr, New York, NY USA.

[Hur, Hye-Chun] New York Univ NYU Langone Hlth, Dept Obstet & Gynecol, 150 55thSt, Brooklyn, NY 11220 USA.

C3 NewYork-Presbyterian Hospital; Columbia University; NewYork-Presbyterian

Hospital; Columbia University; Columbia University; NewYork-Presbyterian

Hospital

RP Hur, HC (corresponding author), New York Univ NYU Langone Hlth, Dept Obstet & Gynecol, 150 55thSt, Brooklyn, NY 11220 USA.

EM hye-chun.hur@nyulangone.org

OI Polin, Melanie/0000-0003-2988-0526

NR 17

TC 4

Z9 4

U1 1

U2 1

PU ELSEVIER SCIENCE INC

PI NEW YORK

PA STE 800, 230 PARK AVE, NEW YORK, NY 10169 USA

SN 1553-4650

EI 1553-4669

J9 J MINIM INVAS GYN

JI J. Minim. Invasive Gynecol.

PD JUL

PY 2023

VL 30

IS 7

BP 562

EP 568

DI 10.1016/j.jmig.2023.03.005

EA JUL 2023

PG 7

WC Obstetrics & Gynecology

WE Science Citation Index Expanded (SCI-EXPANDED)

SC Obstetrics & Gynecology

GA N5YG9

UT WOS:001037759000001

PM 36921892

DA 2024-01-18

ER

PT J

AU Roth, K

Kaier, K

Stachon, P

von zur Mühlen, C

Jungmann, P

Grimm, J

Klar, M

Juhasz-Böss, I

Taran, FA

AF Roth, Katrin

Kaier, Klaus

Stachon, Peter

von zur Muehlen, Constantin

Jungmann, Peter

Grimm, Juliane

Klar, Maximilian

Juhasz-Boess, Ingolf

Taran, Florin-Andrei

TI Evolving trends in the surgical therapy of patients with endometrial

cancer in Germany: analysis of a nationwide registry with special

emphasis on perioperative outcomes

SO ARCHIVES OF GYNECOLOGY AND OBSTETRICS

LA English

DT Article

DE Endometrial cancer; Robotic-assisted laparoscopic surgery; Laparoscopic

surgery

ID MINIMALLY INVASIVE SURGERY; LAPAROSCOPIC HYSTERECTOMY; ROBOTIC SURGERY;

CARCINOMA; MORBIDITY; SURVIVAL; COST

AB PurposeEndometrial cancer (EC) is the most common gynecological malignancy in women, with increasing incidence in the last decades. Surgical therapy is the mainstay of the initial management. The present study analyzed the evolving trends of surgical therapy in Germany in patients diagnosed with EC recorded in a nationwide registry.MethodsAll patients with the diagnosis of EC undergoing open surgery, laparoscopic surgery, and robotic-assisted laparoscopic surgery between 2007 and 2018 were identified by international classification of diseases (ICD) or specific operational codes (OPS) within the database of the German federal bureau of statistics.ResultsA total of 85,204 patients underwent surgical therapy for EC. Beginning with 2013, minimal-invasive surgical therapy was the leading approach for patients with EC. Open surgery was associated with a higher risk of in-hospital mortality (1.3% vs. 0.2%, p < 0.001), of prolonged mechanical ventilation (1.3% vs. 0.2%, p < 0.001), and of prolonged hospital stay (13.7 & PLUSMN; 10.2 days vs. 7.2 & PLUSMN; 5.3 days, p < 0.001) compared to laparoscopic surgery. A total of 1551 (0.04%) patients undergoing laparoscopic surgery were converted to laparotomy. Procedure costs were highest for laparotomy, followed by robotic-assisted laparoscopy and laparoscopy (8286 & PLUSMN; 7533euro vs. 7083 & PLUSMN; 3893euro vs. 6047 & PLUSMN; 3509euro, p < 0.001).ConclusionThe present study revealed that minimal-invasive surgery has increasingly become the standard surgical procedure for patients with EC in Germany. Furthermore, minimal-invasive surgery had superior in-hospital outcomes compared to laparotomy. Moreover, the use of robotic-assisted laparoscopic surgery is increasing, with a comparable in-hospital safety profile to conventional laparoscopy.

C1 [Roth, Katrin; Jungmann, Peter; Grimm, Juliane; Klar, Maximilian; Juhasz-Boess, Ingolf; Taran, Florin-Andrei] Univ Freiburg, Univ Med Ctr Freiburg, Fac Med, Dept Obstet & Gynecol, Hugstetter Str 55, D-79106 Freiburg, Germany.

[Kaier, Klaus] Univ Freiburg, Inst Med Biometry & Stat, Fac Med, Med Ctr, Freiburg, Germany.

[Kaier, Klaus; Stachon, Peter; von zur Muehlen, Constantin] Univ Freiburg, Univ Heart Ctr Freiburg, Fac Med, Dept Cardiol & Angiology1, Freiburg, Germany.

[Stachon, Peter; von zur Muehlen, Constantin] Univ Freiburg, Heart Ctr Freiburg Univ, Ctr Big Data Anal Cardiol CeBAC, Fac Med,Dept Cardiol & Angiol 1, Freiburg, Germany.

C3 University of Freiburg; University of Freiburg; Universitats Herzzentrum

Freiburg; University of Freiburg; University of Freiburg; Universitats

Herzzentrum Freiburg

RP Taran, FA (corresponding author), Univ Freiburg, Univ Med Ctr Freiburg, Fac Med, Dept Obstet & Gynecol, Hugstetter Str 55, D-79106 Freiburg, Germany.

EM florin-andrei.taran@uniklinik-freiburg.de

RI Kaier, Klaus/B-4227-2009

OI Kaier, Klaus/0000-0003-0837-6945; Taran,

Florin-Andrei/0000-0001-7384-2928

FU Projekt DEAL

FX Open Access funding enabled and organized by Projekt DEAL. The authors

declare that no funds, grants, or other support were received during the

preparation of this manuscript.

NR 28

TC 1

Z9 1

U1 1

U2 1

PU SPRINGER HEIDELBERG

PI HEIDELBERG

PA TIERGARTENSTRASSE 17, D-69121 HEIDELBERG, GERMANY

SN 0932-0067

EI 1432-0711

J9 ARCH GYNECOL OBSTET

JI Arch. Gynecol. Obstet.

PD NOV

PY 2023

VL 308

IS 5

BP 1635

EP 1640

DI 10.1007/s00404-023-07127-0

EA JUL 2023

PG 6

WC Obstetrics & Gynecology

WE Science Citation Index Expanded (SCI-EXPANDED)

SC Obstetrics & Gynecology

GA S8IQ4

UT WOS:001021348100002

PM 37395751

OA Green Published, Green Submitted, hybrid

DA 2024-01-18

ER

PT J

AU Isono-Taniguchi, R

Tsubamoto, H

Inoue, K

Ueda, T

Saeki, S

Takimoto, Y

Wakimoto, Y

Shibahara, H

AF Isono-Taniguchi, Roze

Tsubamoto, Hiroshi

Inoue, Kayo

Ueda, Tomoko

Saeki, Shinichiro

Takimoto, Yumi

Wakimoto, Yu

Shibahara, Hiroaki

TI Weight-loss interventions and levonorgestrel intrauterine system

implantation for early-stage endometrial cancer and atypical endometrial

hyperplasia to reduce perioperative risk of severely obese patients

SO GYNECOLOGY AND MINIMALLY INVASIVE THERAPY-GMIT

LA English

DT Article

DE Atypical endometrial hyperplasia; endometrial cancer; levonorgestrel

intrauterine system; preoperative management; weight-loss interventions

ID COMPLICATIONS; OUTCOMES; IMPACT

AB Endometrial cancer (EC) and atypical endometrial hyperplasia (AEH) are associated with obesity, which increases the perioperative morbidity and surgical difficulties in laparoscopic and robotic surgery. Weight-loss interventions (WLIs) are likely to reduce morbidity; however, delayed surgery may cause cancer progression. To minimize the tumor progression, levonorgestrel intrauterine system (LNG-IUS) with minimal side effects was used until the planned surgery. During 2016 and 2021, we conducted preoperative management of WLI using LNG-IUS for seven highly obese women with a body mass index (BMI) =35 kg/m(2) who had AEH and EC with Grade 1 and no myometrial invasion on magnetic resonance imaging. In three of the seven patients, the BMI decreased by more than 5. Two patients with AEH achieved remission after LNG-IUS placement and requested conservative management. Five patients with EC underwent laparoscopic hysterectomy, without perioperative complications.

C1 [Isono-Taniguchi, Roze; Tsubamoto, Hiroshi; Inoue, Kayo; Ueda, Tomoko; Saeki, Shinichiro; Takimoto, Yumi; Wakimoto, Yu; Shibahara, Hiroaki] Hyogo Med Univ, Dept Obstet & Gynecol, Nishinomiya, Hyogo, Japan.

[Tsubamoto, Hiroshi] Hyogo Med Univ, Dept Obstet & Gynecol, Mukogawa 1-1, Nishinomiya, Hyogo 6638501, Japan.

C3 Hyogo College of Medicine; University of Hyogo; University of Hyogo;

Hyogo College of Medicine

RP Tsubamoto, H (corresponding author), Hyogo Med Univ, Dept Obstet & Gynecol, Mukogawa 1-1, Nishinomiya, Hyogo 6638501, Japan.

EM tsuba@hyo-med.ac.jp

NR 11

TC 0

Z9 0

U1 0

U2 0

PU WOLTERS KLUWER MEDKNOW PUBLICATIONS

PI MUMBAI

PA WOLTERS KLUWER INDIA PVT LTD , A-202, 2ND FLR, QUBE, C T S NO 1498A-2

VILLAGE MAROL, ANDHERI EAST, MUMBAI, Maharashtra, INDIA

SN 2213-3070

EI 2213-3089

J9 GYNECOL MINIM INVASI

JI Gynecol. Minim. Invasive Ther.-GMIT

PD JUL-SEP

PY 2023

VL 12

IS 3

BP 175

EP 178

DI 10.4103/gmit.gmit_98_22

PG 4

WC Obstetrics & Gynecology

WE Emerging Sources Citation Index (ESCI)

SC Obstetrics & Gynecology

GA R1RS0

UT WOS:001062188900010

PM 37807990

OA Green Published, gold

DA 2024-01-18

ER

PT J

AU Kantarci, S

Inan, AH

Töz, E

Bolukbasi, M

Kanmaz, AG

AF Kantarci, Sercan

Inan, Abdurrahman Hamdi

Toz, Emrah

Bolukbasi, Mehmet

Kanmaz, Ahkam Goksel

TI Analysis of hysterectomy trends in the last 5 years at a tertiary center

SO GYNECOLOGY AND MINIMALLY INVASIVE THERAPY-GMIT

LA English

DT Article

DE Laparoscopic hysterectomy; minimally invasive surgery; major

complications

ID VAGINAL CUFF DEHISCENCE

AB Objectives: This study aimed to assess trends by evaluating the types and complications of hysterectomies performed for benign gynecological reasons at our clinic, which is one of the largest hospitals in Turkey. Materials and Methods: Hysterectomies performed for benign reasons at our gynecology and obstetrics clinic between January 1, 2015 and December 31, 2020 were retrospectively reviewed and included in the analysis. Of the 4288 patients who had undergone hysterectomy, 888 patients were excluded some reasons. The data of the remaining 3400 patients were analyzed. Results: For the 3400 patients, the hysterectomy methods performed were as follows: Total Abdominal Hysterectomy (TAH (60%, n = 2055), Total Laparoscopic Hysterectomy (TLH), (27%, n = 948), Vaginal Hysterectomy (VH), (8.9%, n = 302), Conversion from laparoscopy to laparotomy (L / S > LT). (1.4%, n = 49), Robotic hysterectomy (RH), (1%, n = 33), and Subtotal hysterectomy (SH), (0.4%, n = 13). The length of hospital stay was statistically significantly lower in the TLH group than in the TAH group (P < 0.05). A statistically significant and moderate correlation was noted between the length of hospital stay and the duration of operation (r: 0.68 P = 0.00). Conclusion: The ratio of TLH group among hysterectomy modalities has increased over the years. There are many factors that affect the surgeon's decision in determining the hysterectomy method.TLH is the first option in patients who are not suitable for vaginal hysterectomy.

C1 [Kantarci, Sercan; Inan, Abdurrahman Hamdi; Toz, Emrah; Bolukbasi, Mehmet; Kanmaz, Ahkam Goksel] Tepecik Training & Res Hosp, Dept Obstet & Gynecol, Izmir, Turkiye.

[Kantarci, Sercan] Tepecik Training & Res Hosp, Dept Obstet & Gynecol, TR-35170 Izmir, Turkiye.

C3 Izmir Tepecik Training & Research Hospital; Izmir Tepecik Training &

Research Hospital

RP Kantarci, S (corresponding author), Tepecik Training & Res Hosp, Dept Obstet & Gynecol, TR-35170 Izmir, Turkiye.

EM sercan.kntrc@gmail.com

NR 19

TC 0

Z9 0

U1 0

U2 0

PU WOLTERS KLUWER MEDKNOW PUBLICATIONS

PI MUMBAI

PA WOLTERS KLUWER INDIA PVT LTD , A-202, 2ND FLR, QUBE, C T S NO 1498A-2

VILLAGE MAROL, ANDHERI EAST, MUMBAI, Maharashtra, INDIA

SN 2213-3070

EI 2213-3089

J9 GYNECOL MINIM INVASI

JI Gynecol. Minim. Invasive Ther.-GMIT

PD JUL-SEP

PY 2023

VL 12

IS 3

BP 135

EP 140

DI 10.4103/gmit.gmit_30_22

PG 6

WC Obstetrics & Gynecology

WE Emerging Sources Citation Index (ESCI)

SC Obstetrics & Gynecology

GA R1RS0

UT WOS:001062188900003

PM 37807992

OA Green Published, gold

DA 2024-01-18

ER

PT J

AU Rustia, GM

Baracy, MG

Khair, E

Hagglund, KH

Aslam, MF

AF Rustia, Gabriella M.

Baracy Jr, Michael G. G.

Khair, Emilee

Hagglund, Karen H.

Aslam, Muhammad Faisal

TI Pain With Differing Insufflation Pressures During Robotic Sacrocolpopexy

A Randomized Controlled Trial

SO OBSTETRICS AND GYNECOLOGY

LA English

DT Article

ID ENHANCED RECOVERY; SURGERY; GUIDELINES

AB OBJECTIVE:To evaluate whether decreasing insufflation pressure reduces postoperative pain and opioid use in women undergoing robotic-assisted sacrocolpopexy.METHODS:In a single-blinded randomized trial, women with pelvic organ prolapse underwent robotic-assisted sacrocolpopexy at either 12 mm Hg (experimental) or 15 mm Hg (standard) insufflation pressure. The primary outcome was pain rating on a visual analog scale (VAS) on postoperative day 1 within 24 hours of surgery. Secondary outcomes included VAS pain rating at outpatient follow-up, inpatient and outpatient use of opioids, operative time, and estimated blood loss. A margin of 15 mm was considered clinically different on the VAS, and at 80% power, a sample size of at least 64 participants was needed to show significance.RESULTS:From April 27, 2021, to May 17, 2022, 80 women were enrolled, with 41 in the experimental group and 39 in the standard group. All participants underwent surgery as planned and attended a 2-week postoperative follow-up. Participants in the experimental group had less pain on postoperative day 1 with median VAS of 17.0 mm (interquartile range 26.0) compared with 29.0 mm (interquartile range 32.0, P=.007) in the standard group. No differences were noted in the secondary outcomes of operative time, estimated blood loss, or length of stay. Participants in the experimental group were noted to use fewer opioids while an inpatient (P=.04) and outpatient (P=.02). In multivariable analyses, lower insufflation pressure and increasing age were negatively associated with postoperative VAS scores.CONCLUSION:Lowering insufflation pressure (12 mm Hg) during robotic-assisted sacrocolpopexy safely reduced postoperative pain and opioid use compared with standard pressure (15 mm Hg).

C1 [Rustia, Gabriella M.] Ascension St John Hosp, Dept Obstet & Gynecol, Detroit, MI 48236 USA.

Ascension St John Hosp, Dept Biomed Invest & Res, Detroit, MI USA.

Ascension St John Hosp, Dept Obstet & Gynecol, Div FPMRS, Detroit, MI USA.

Michigan State Univ, E Lansing, MI USA.

C3 Michigan State University

RP Rustia, GM (corresponding author), Ascension St John Hosp, Dept Obstet & Gynecol, Detroit, MI 48236 USA.

EM gmrustia@gmail.com

FU Department of Graduate Medical Education at Ascension St. John Hospital

FX The Department of Graduate Medical Education at Ascension St. John

Hospital provided funding for this study

NR 16

TC 0

Z9 0

U1 0

U2 0

PU LIPPINCOTT WILLIAMS & WILKINS

PI PHILADELPHIA

PA TWO COMMERCE SQ, 2001 MARKET ST, PHILADELPHIA, PA 19103 USA

SN 0029-7844

J9 OBSTET GYNECOL

JI Obstet. Gynecol.

PD JUL

PY 2023

VL 142

IS 1

BP 151

EP 159

DI 10.1097/AOG.0000000000005231

PG 9

WC Obstetrics & Gynecology

WE Science Citation Index Expanded (SCI-EXPANDED)

SC Obstetrics & Gynecology

GA L8FT6

UT WOS:001025570900019

PM 37348093

DA 2024-01-18

ER

PT J

AU Arcieri, M

Morlacco, A

Montebelli, F

Mancini, M

Soligo, M

Restaino, S

Driul, L

Campagna, G

Panico, G

Ercoli, A

Scambia, G

Dal Moro, F

Vizzielli, G

AF Arcieri, Martina

Morlacco, Alessandro

Montebelli, Francesco

Mancini, Mariangela

Soligo, Matteo

Restaino, Stefano

Driul, Lorenza

Campagna, Giuseppe

Panico, Giovanni

Ercoli, Alfredo

Scambia, Giovanni

Dal Moro, Fabrizio

Vizzielli, Giuseppe

TI Sacrocolpopexy after sub-total hysterectomy vs. sacral hysteropexy for

advanced urogenital prolapse: A propensity-matched study

SO INTERNATIONAL JOURNAL OF GYNECOLOGY & OBSTETRICS

LA English

DT Article; Early Access

DE Hysteropexy; mini-invasive surgery; pelvic organ prolapse; robotic

surgery; Sacropexy; urogynecology

ID SURGERY; COMPLICATIONS; VALIDATION

AB ObjectiveTo compare objective and subjective outcomes of laparoscopic sacral colpopexy with supracervical hysterectomy (L-SCP) and robotic sacral hysteropexy (R-SHP). MethodsThis is a multicenter retrospective propensity score matched study. In the period between January 2014 and December 2018, we enrolled 161 patients with apical prolapse stage 2 or above, alone or with multicompartment descensus. ResultsAfter propensity-match analysis, there were 44 women for each group. Patients of the two groups had similar preoperative characteristics. No difference was found in terms of estimated blood loss, hospital stay, operative time, and intraoperative or postoperative complications. Subjective success rate, 12 months after surgery, was statistically better in the L-SCP group (P = 0.034): 81.8% and 97.8% women had Patient Global Impression of Improvement scores less than 3, in R-SHP and L-SCP, respectively. The objective cure rate was high in both groups without any significant differences in recurrence rate (P = 0.266). ConclusionBoth procedures are safe and effective in pelvic organ prolapse treatment. Patients who no longer desire uterine preservation could be encouraged to consider L-SCP. R-SHP is an alternative in women who are strongly motivated to preserve their uterus in the absence of abnormal uterine findings.

C1 [Arcieri, Martina] Univ Messina, Dept Biomed Dent Morphol & Funct Imaging Sci, Via Consolare Valeria, I-98125 Messina, Italy.

[Arcieri, Martina; Restaino, Stefano; Driul, Lorenza; Vizzielli, Giuseppe] Univ Hosp Udine, Dept Maternal & Child Hlth, Obstet & Gynecol Clin, Udine, Italy.

[Morlacco, Alessandro; Montebelli, Francesco; Mancini, Mariangela; Soligo, Matteo; Dal Moro, Fabrizio] Padova Univ, Dept Surg Oncol & Gastroenterol Sci, Urol Clin, Padua, Italy.

[Driul, Lorenza; Vizzielli, Giuseppe] Univ Udine, Dept Med, Obstet & Gynecol Clin, Udine, Italy.

[Campagna, Giuseppe; Panico, Giovanni; Scambia, Giovanni] Fdn Policlin Univ A Gemelli, Ist Ricovero & Cura Carattere Sci IRCCS, Dept Woman Child & Publ Hlth, Rome, Italy.

[Ercoli, Alfredo] Univ Messina, Dept Human Pathol Adult & Childhood G Barresi, Unit Gynecol & Obstet, Messina, Italy.

[Scambia, Giovanni] Univ Cattolica Sacro Cuore, Dept Woman Child & Publ Hlth, Rome, Italy.

C3 University of Messina; University of Udine; University Hospital of

Udine; University of Padua; University of Udine; Catholic University of

the Sacred Heart; IRCCS Policlinico Gemelli; University of Messina;

Catholic University of the Sacred Heart; IRCCS Policlinico Gemelli

RP Arcieri, M (corresponding author), Univ Messina, Dept Biomed Dent Morphol & Funct Imaging Sci, Via Consolare Valeria, I-98125 Messina, Italy.

EM martina.arcieri@unime.it

RI Panico, Giovanni/GLV-0862-2022; Arcieri, Martina/AEU-5553-2022;

Vizzielli, Giuseppe/L-1062-2016

OI Panico, Giovanni/0000-0001-5473-9503; Arcieri,

Martina/0000-0002-8257-0618; Vizzielli, Giuseppe/0000-0002-2424-2691

NR 25

TC 1

Z9 1

U1 0

U2 0

PU WILEY

PI HOBOKEN

PA 111 RIVER ST, HOBOKEN 07030-5774, NJ USA

SN 0020-7292

EI 1879-3479

J9 INT J GYNECOL OBSTET

JI Int. J. Gynecol. Obstet.

PD 2023 JUN 29

PY 2023

DI 10.1002/ijgo.14959

EA JUN 2023

PG 7

WC Obstetrics & Gynecology

WE Science Citation Index Expanded (SCI-EXPANDED)

SC Obstetrics & Gynecology

GA K4OR9

UT WOS:001016253600001

PM 37382353

OA hybrid

DA 2024-01-18

ER

PT J

AU Taylor, KN

Kim, KH

AF Taylor, Kristin N.

Kim, Kenneth H.

TI Robotics in Gynecologic Oncology: Past, Present, and Future

SO JOURNAL OF GYNECOLOGIC SURGERY

LA English

DT Article

DE gynecologic surgery; gynecologic cancer; minimally invasive surgery;

robotic surgery; robotic simulation

ID LAPAROSCOPIC HYSTERECTOMY; ASSISTED HYSTERECTOMY; CANCER; LAPAROTOMY;

SURVIVAL; SURGERY; VALIDATION

AB In the 20 years since its inception, robotic surgery has evolved greatly in its design and clinical use. Most recently, the approach to training and gaining proficiency in robotic surgery techniques has also matured. This article reviews the development of robotic surgery systems, the trajectory of their use for patients with benign and malignant gynecologic conditions, and the advances in novel technologies that are driving the applications of robotic surgery forward. (J GYNECOL SURG 20XX:000)

C1 [Taylor, Kristin N.; Kim, Kenneth H.] Cedars Sinai Med Ctr, Samuel Oschin Canc Ctr, Dept Obstet & Gynecol, Div Gynecol Oncol, Los Angeles, CA USA.

[Kim, Kenneth H.] Cedars Sinai Med Ctr, 8700 Beverly Blvd,Suite 290W, Los Angeles, CA 90048 USA.

C3 Cedars Sinai Medical Center; Cedars Sinai Medical Center

RP Kim, KH (corresponding author), Cedars Sinai Med Ctr, 8700 Beverly Blvd,Suite 290W, Los Angeles, CA 90048 USA.

EM kenneth.kim3@cshs.org

NR 37

TC 0

Z9 0

U1 0

U2 0

PU MARY ANN LIEBERT, INC

PI NEW ROCHELLE

PA 140 HUGUENOT STREET, 3RD FL, NEW ROCHELLE, NY 10801 USA

SN 1042-4067

EI 1557-7724

J9 J GYNECOL SURG

JI J. Gynecol. Surg.

PD OCT 1

PY 2023

VL 39

IS 5

BP 204

EP 212

DI 10.1089/gyn.2023.0042

EA JUN 2023

PG 9

WC Obstetrics & Gynecology; Surgery

WE Emerging Sources Citation Index (ESCI)

SC Obstetrics & Gynecology; Surgery

GA T7MD3

UT WOS:001018053400001

DA 2024-01-18

ER

PT J

AU Lönnerfors, C

Persson, J

AF Lonnerfors, Celine

Persson, Jan

TI Can robotic-assisted surgery support enhanced recovery programs?

SO BEST PRACTICE & RESEARCH CLINICAL OBSTETRICS & GYNAECOLOGY

LA English

DT Article

DE ERAS; Enhanced recovery; MIS; Robotic surgery; Sweden

ID SAME-DAY DISCHARGE; TOTAL LAPAROSCOPIC HYSTERECTOMY; POSTOPERATIVE

URINARY RETENTION; LENGTH-OF-STAY; GYNECOLOGIC ONCOLOGY;

RANDOMIZED-TRIAL; PERIOPERATIVE OUTCOMES; VAGINAL HYSTERECTOMY;

COLORECTAL SURGERY; BOWEL PREPARATION

AB Enhanced recovery after surgery (ERAS) protocols comprise a multimodal approach to optimize patient outcome and recovery. ERAS guidelines recommend minimally invasive surgery (MIS) when possible. Key components in MIS include preoperative patient education and optimization; multimodal and narcoticsparing analgesia; prophylactic measures regarding nausea, infection, and venous thrombosis; maintenance of euvolemia; and promotion of the early activity. ERAS protocols in MIS improve outcome mainly in terms of reduced length of stay and subsequently reduced cost. In addition, ERAS protocols in MIS reduce postoperative pain and nausea, increase patient satisfaction, and might reduce the rate of postoperative complications. Robotic surgery supports ERAS through facilitating MIS in complex procedures where laparotomy is an alternative approach.& COPY; 2023 Published by Elsevier Ltd.

C1 [Lonnerfors, Celine; Persson, Jan] Skane Univ Hosp, Dept Obstet & Gynecol, Div Gynecol Oncol, Lund, Sweden.

[Lonnerfors, Celine; Persson, Jan] Lund Univ, Fac Med, Dept Clin Sci Obstet & Gynecol, Lund, Sweden.

[Lonnerfors, Celine] Skane Univ Hosp, Dept Obstet & Gynecol, SE-22185 Lund, Sweden.

C3 Lund University; Skane University Hospital; Lund University; Lund

University; Skane University Hospital

RP Lönnerfors, C (corresponding author), Skane Univ Hosp, Dept Obstet & Gynecol, SE-22185 Lund, Sweden.

EM celine.lonnerfors@skane.se; jan.persson@med.lu.se

NR 93

TC 0

Z9 0

U1 1

U2 1

PU ELSEVIER SCI LTD

PI OXFORD

PA THE BOULEVARD, LANGFORD LANE, KIDLINGTON, OXFORD OX5 1GB, OXON, ENGLAND

SN 1521-6934

EI 1532-1932

J9 BEST PRACT RES CL OB

JI Best Pract. Res. Clin. Obstet. Gynaecol.

PD AUG

PY 2023

VL 90

AR 102366

DI 10.1016/j.bpobgyn.2023.102366

EA JUN 2023

PG 13

WC Obstetrics & Gynecology

WE Science Citation Index Expanded (SCI-EXPANDED)

SC Obstetrics & Gynecology

GA N4UV5

UT WOS:001036990600001

PM 37356336

DA 2024-01-18

ER

PT J

AU Saini, A

Gao, JY

Leung, K

Wilkie, G

Matteson, K

Korets, S

AF Saini, Aashna

Gao, Jenny

Leung, Katherine

Wilkie, Gianna

Matteson, Kristen

Korets, Sharmilee

TI Intra-operative tumor spillage in minimally invasive surgery for

endometrial cancer and its impact on recurrence risk

SO GYNECOLOGIC ONCOLOGY

LA English

DT Article

DE Intra-operative tumor spillage in endometrial cancer; Minimally invasive

surgery endometrial cancer Recurrence with intra-operative tumor

spillage Uterine manipulator and recurrence in endo- metrial cancer;

Uterine perforation and recurrence in endome- trial cancer;

Intra-operative tumor spillage and minimally invasive surgery

ID LAPAROSCOPIC HYSTERECTOMY; CELL SPILLAGE; SURVIVAL; RUPTURE; CAVITY

AB Objective. The prognostic impact of intra-operative tumor spillage (ITS) during minimally invasive surgery (MIS) for endometrial cancer (EC) is not well studied. The objective of this study was to determine if there is an association between ITS and EC recurrence.Methods. We performed a case-control study of patients with a laparoscopic or robot-assisted hysterectomy with EC on final pathology between 2017 and 2022 and compared those with (case) and without (control) a sub-sequent EC recurrence. Electronic medical records were reviewed for demographic, intra-operative and patho-logic details, and recurrence status. ITS was defined as uterine perforation with a manipulator, presence of extra-uterine tumor after colpotomy or specimen delivery, exposure of uncontained specimen into peritoneum, and/or pathology/operative reports noting specimen fragmentation. Conditional logistic regression was used to determine odds ratios for the association of cancer recurrence with ITS. We adjusted for >50% myoinvasion, tumor size, and adjuvant treatment.Results. 1057 patients underwent MIS for EC. Approximately 8% (n = 86) developed recurrent cancer and 172 patients were selected as controls. Twenty percent of recurrent cases (17/86) had ITS compared with 4% of non -recurrent controls (7/172). When adjusted for tumor size, deep myoinvasion, and adjuvant treatment, patients with ITS had a 5.6 times increased odds (aOR 5.63, 95% CI 1.52-20.86) of recurrence compared to patients without ITS.Conclusions. In patients with EC, we found an association between ITS and cancer recurrence. These findings warrant further investigation to determine if adjuvant therapy or surgical technique should be altered to improve outcomes.& COPY; 2023 Published by Elsevier Inc.

C1 [Saini, Aashna; Wilkie, Gianna; Matteson, Kristen; Korets, Sharmilee] Univ Massachusetts, Dept Obstet & Gynecol, Mem Med Ctr, Worcester, MA 01605 USA.

[Saini, Aashna; Gao, Jenny; Leung, Katherine; Wilkie, Gianna; Matteson, Kristen; Korets, Sharmilee] Univ Massachusetts, TH Chan Med Sch, Worcester, MA 01655 USA.

[Saini, Aashna] Univ Massachusetts, Dept Obstet & Gynecol, Med Ctr, Worcester, MA 01605 USA.

C3 University of Massachusetts System; University of Massachusetts

Worcester; University of Massachusetts System; University of

Massachusetts Worcester; University of Massachusetts System; University

of Massachusetts Worcester

RP Saini, A (corresponding author), Univ Massachusetts, Dept Obstet & Gynecol, Med Ctr, Worcester, MA 01605 USA.

EM Aashna.saini@umassmemorial.org

NR 25

TC 1

Z9 1

U1 2

U2 2

PU ACADEMIC PRESS INC ELSEVIER SCIENCE

PI SAN DIEGO

PA 525 B ST, STE 1900, SAN DIEGO, CA 92101-4495 USA

SN 0090-8258

EI 1095-6859

J9 GYNECOL ONCOL

JI Gynecol. Oncol.

PD AUG

PY 2023

VL 175

BP 128

EP 132

DI 10.1016/j.ygyno.2023.06.005

EA JUN 2023

PG 5

WC Oncology; Obstetrics & Gynecology

WE Science Citation Index Expanded (SCI-EXPANDED)

SC Oncology; Obstetrics & Gynecology

GA N2XK0

UT WOS:001035701700001

PM 37356313

DA 2024-01-18

ER

PT J

AU Sallée, C

Lacorre, A

Despoux, F

Mbou, VB

Margueritte, F

Gauthier, T

AF Sallee, C.

Lacorre, A.

Despoux, F.

Mbou, V. B.

Margueritte, F.

Gauthier, T.

TI Use of uterine manipulator and uterine perforation in minimally invasive

endometrial cancer surgery

SO JOURNAL OF GYNECOLOGY OBSTETRICS AND HUMAN REPRODUCTION

LA English

DT Article

DE Endometrial cancer; Uterine manipulator; Uterine perforation

ID TOTAL LAPAROSCOPIC HYSTERECTOMY; LYMPHOVASCULAR SPACE INVASION;

MANAGEMENT; CYTOLOGY; RISK

AB Objective: Safety of the uterine manipulator (UM) within endometrial cancer (EC) surgery is being ques-tioned. Its use might be one of the issues for potential tumor dissemination during the procedure, especially in the case of uterine perforation (UP). No prospective data on this surgical complication, nor on the oncolog-ical consequences exist. The aim of this study was to assess the rate of UP while using UM when performing surgery for EC and the impact of UP on the choice of adjuvant treatment. Methods: We conducted a prospective single-center cohort study from November 2018 to February 2022, considering all EC cases surgically treated by a minimally invasive approach with the help of a UM. Demo-graphic, preoperative, postoperative and adjuvant treatment corresponding to the included patients were collected and comparatively analyzed according to the absence or presence of a UP. Results: Of the 82 patients included in the study, 9 UPs (11%) occurred during surgery. There was no signifi-cant difference in demographics and disease characteristics at diagnosis that may have induced UP. The type of UM used or the approach (laparoscopic vs. robotic) did not influence the occurrence of UP (p = 0.44). No positive peritoneal cytology was found post hysterectomy. There was a statistically significantly higher rate of lymph-vascular space invasion within the perforation group, 67% vs. 25% in the no perforation group, p = 0.02. Two out of nine (22%) adjuvant therapies were changed because of UP. The median follow-up time for patients was 7.6 months (range 0.5-33.1 months). No recurrence was found in the UP group. Conclusion: Our study found a uterine perforation rate of 11%. This information needs to be further integrated to consider the usefulness of MU for EC surgery. & COPY; 2023 Elsevier Masson SAS. All rights reserved.

C1 [Sallee, C.; Lacorre, A.; Despoux, F.; Gauthier, T.] CHU Limoges, Dept Gynecol & Obstet, 8 Ave Domin Larrey, F-87042 Limoges, France.

[Mbou, V. B.] CHU Limoges, Dept Anatomopathol, 8 Ave Dominique Larrey, F-87042 Limoges, France.

[Margueritte, F.] CHI Poissy, Dept Gynecol & Obstet, 10 Rue Champ Gaillard, F-78300 Poissy, France.

C3 CHU Limoges; CHU Limoges; Hospital Chi of Poissy Saint Germain

RP Sallée, C (corresponding author), CHU Limoges, Dept Gynecol & Obstet, 8 Ave Domin Larrey, F-87042 Limoges, France.

EM camille.sallee@gmail.com

NR 33

TC 0

Z9 0

U1 0

U2 0

PU ELSEVIER MASSON, CORP OFF

PI PARIS

PA 65 CAMILLE DESMOULINS CS50083 ISSY-LES-MOULINEAUX, 92442 PARIS, FRANCE

SN 2468-7847

EI 1773-0430

J9 J GYNECOL OBSTET HUM

JI J. Gynecol. Obstet. Hum. Reprod.

PD SEP

PY 2023

VL 52

IS 7

AR 102621

DI 10.1016/j.jogoh.2023.102621

EA JUN 2023

PG 6

WC Obstetrics & Gynecology

WE Science Citation Index Expanded (SCI-EXPANDED)

SC Obstetrics & Gynecology

GA N0PC4

UT WOS:001034128900001

PM 37301478

DA 2024-01-18

ER

PT J

AU Mercorio, A

Zizolfi, B

Barbuto, S

Danzi, R

Sardo, AD

Moawad, G

Bifulco, G

Giampaolino, P

AF Mercorio, Antonio

Zizolfi, Brunella

Barbuto, Simona

Danzi, Roberta

Sardo, Attilio Di Spiezio

Moawad, Gaby

Bifulco, Giuseppe

Giampaolino, Pierluigi

TI Three-dimensional imaging reconstruction and laparoscopic robotic

surgery: a winning combination for a complex case of multiple myomectomy

SO FERTILITY AND STERILITY

LA English

DT Article

DE Myomectomy; infertility; laparoscopic robotic surgery; 3D imaging

AB Objective: To demonstrate the intraoperative use of three-dimensional (3D) imaging reconstruction for a complex case of multiple myomectomy assigned to robot-assisted laparoscopic surgery.Design: Stepwise demonstration of the technique with narrated video footage.Setting: University tertiary care hospital.Patient(s): A 36-year-old nulliparous infertile woman with multiple uterine myomas (>20) presented with menorrhagia and pelvic discomfort for many months. Because of the huge number of fibroids present, the patient was considered eligible for laparoscopic robotic-assisted myomectomy.Intervention(s): A robotic-assisted laparoscopic myomectomy was performed with the use of intraoperative 3D imaging reconstruction. After opening the retroperitoneum through the adnexal triangle and identifying the ureters, to reduce intraoperative bleeding, bulldog clamps were used to temporarily reduce uterine vascularization. A multiple myomectomy was then performed with the use of tenaculum and Maryland bipolar forceps. During the intervention, the surgeon used the 3D uterine reconstruction to adapt its surgical strategy. Multilayer running closure was achieved using a bidirectional barbed suture ensuring introflexion of the serosa. Patients' consent was obtained for publication of the case; institutional review board approval was not required for this case report as per our institution's policy.Main Outcome Measure(s): Description of a robotic-assisted myomectomy with the intraoperative use of 3D imaging reconstruction.Result(s): The total operative time was 105 minutes. A total of 21 fibroids were removed with 150 mL of intraoperative blood loss. The patient was discharged the day after.Conclusion(s): The application of 3D imaging technology could overcome one of the limitations of robot-assisted minimally invasive surgery, the lack of haptic feedback, enabling the surgeon to rapidly locate myomas and guide the intraoperative plan to optimize the results. Additional studies evaluating the clinical impact of this technique and its improvement are required. (Fertil Sterile 2023;120:202-4.& COPY;2023 by American Society for Reproductive Medicine.) El resumen esta disponible en Espanol al final del articulo.

C1 [Mercorio, Antonio; Zizolfi, Brunella; Bifulco, Giuseppe] Univ Naples Federico II, Dept Neurosci Reprod Sci & Dent, Naples, Italy.

[Mercorio, Antonio; Danzi, Roberta; Sardo, Attilio Di Spiezio; Giampaolino, Pierluigi] Univ Naples Federico II, Dept Publ Hlth, Naples, Italy.

[Barbuto, Simona] Medics, Turin, Italy.

[Moawad, Gaby] George Washington Univ, Dept Obstet & Gynecol, Washington, DC USA.

[Moawad, Gaby] Ctr Endometriosis & Adv Pelv Surg, Washington, DC USA.

C3 University of Naples Federico II; University of Naples Federico II;

George Washington University

RP Mercorio, A (corresponding author), Univ Naples Federico II, Dept Publ Hlth, Naples, Italy.; Mercorio, A (corresponding author), Univ Naples Federico II, Dept Publ Hlth, Via Sergio Pansini 5, I-80131 Naples, Italy.

EM antoniomercorio@gmail.com

OI Mercorio, Antonio/0000-0002-2113-7706

NR 6

TC 1

Z9 1

U1 2

U2 2

PU ELSEVIER SCIENCE INC

PI NEW YORK

PA STE 800, 230 PARK AVE, NEW YORK, NY 10169 USA

SN 0015-0282

EI 1556-5653

J9 FERTIL STERIL

JI Fertil. Steril.

PD JUL

PY 2023

VL 120

IS 1

BP 202

EP 204

DI 10.1016/j.fertnstert.2023.04.015

EA JUN 2023

PG 3

WC Obstetrics & Gynecology; Reproductive Biology

WE Science Citation Index Expanded (SCI-EXPANDED)

SC Obstetrics & Gynecology; Reproductive Biology

GA L8ND2

UT WOS:001025767200001

PM 37085096

DA 2024-01-18

ER

PT J

AU Gwacham, NI

Kilowski, KA

Recio, FO

Awada, A

Kuhn, TM

Zhu, JB

Patel, A

Ahmad, S

McKenzie, ND

Kendrick, JE

Holloway, RW

AF Gwacham, Nnamdi I.

Kilowski, Karolina A.

Recio, Fernando O.

Awada, Ahmad

Kuhn, Theresa M.

Zhu, Jianbin

Patel, Ameya

Ahmad, Sarfraz

McKenzie, Nathalie D.

Kendrick, James E.

Holloway, Robert W.

TI Malignant peritoneal cytologic contamination with robotic hysterectomy

for endometrial cancer

SO GYNECOLOGIC ONCOLOGY

LA English

DT Article

DE Endometrial cancer; Minimally invasive surgery; Peritoneal cytology;

Robotic hysterectomy; Pelvic washings

ID LAPAROSCOPIC RADICAL HYSTERECTOMY; PROGNOSTIC-SIGNIFICANCE; CARCINOMA;

SURVIVAL; CERVIX

AB Background. Malignant peritoneal cytology in endometrial cancer (EC) is not considered an independent ad-verse prognostic factor for uterine-confined disease and is not a determinant factor in the International Federa-tion of Gynecology and Obstetrics (FIGO) staging system. NCCN Guidelines still recommend obtaining cytologies. The aim of this study was to determine the prevalence of peritoneal cytologic contamination following robotic hysterectomy for EC. Methods. Peritoneal cytology from the pelvis and diaphragm were obtained at the initiation of surgery, and from the pelvis only at the completion of robotic hysterectomy with sentinel lymph node mapping (SLNM). Cy-tology specimens were evaluated for the presence of malignant cells. Pre-and post-hysterectomy cytology re-sults were compared, and pelvic contamination was defined as conversion from negative to positive cytology following surgery. Results. 244 patients underwent robotic hysterectomy with SLNM for EC. Pelvic contamination was identified in 32 (13.1%) cases. In multivariate analysis, pelvic contamination was associated with >50% myometrial inva-sion, tumor size >2 cm, lymphovascular space invasion (LVSI), and lymph node metastasis. There was no asso-ciation with FIGO stage or histology subtypes. Conclusions. Malignant peritoneal contamination occurred during robotic surgery for EC. Large lesions (>2 cm), deep invasion (>50%), LVSI, and lymph node metastasis were each independently associated with peri-toneal contamination. Whether or not peritoneal contamination increases risk for disease recurrence should be studied in larger series, including an evaluation of patterns of recurrence and the potential impact of adjuvant therapies. Until the clinical impact of peritoneal contamination during hysterectomy for EC is better understood, methods to reduce peritoneal contamination are warranted. & COPY; 2023 Elsevier Inc. All rights reserved.

C1 [Gwacham, Nnamdi I.; Kilowski, Karolina A.; Recio, Fernando O.; Awada, Ahmad; Kuhn, Theresa M.; Zhu, Jianbin; Ahmad, Sarfraz; McKenzie, Nathalie D.; Kendrick, James E.; Holloway, Robert W.] AdventHlth Canc Inst, Gynecol Oncol Program, Orlando, FL 32804 USA.

[Patel, Ameya] Trinity Preparatory Sch, Winter Pk, FL 32792 USA.

[Gwacham, Nnamdi I.; Ahmad, Sarfraz] AdventHlth Canc Inst, Gynecol Oncol Program, 2501 N Orange Ave,Ste 786, Orlando, FL 32804 USA.

C3 Adventist Health Services; AdventHealth; Adventist Health Services;

AdventHealth

RP Gwacham, NI; Ahmad, S (corresponding author), AdventHlth Canc Inst, Gynecol Oncol Program, 2501 N Orange Ave,Ste 786, Orlando, FL 32804 USA.

EM nnamdi.gwacham.do@adventhealth.com; sarfraz.ahmad@adventhealth.com

OI McKenzie, Nathalie/0000-0002-9550-3457

NR 36

TC 0

Z9 0

U1 1

U2 2

PU ACADEMIC PRESS INC ELSEVIER SCIENCE

PI SAN DIEGO

PA 525 B ST, STE 1900, SAN DIEGO, CA 92101-4495 USA

SN 0090-8258

EI 1095-6859

J9 GYNECOL ONCOL

JI Gynecol. Oncol.

PD AUG

PY 2023

VL 175

BP 93

EP 96

DI 10.1016/j.ygyno.2023.06.006

EA JUN 2023

PG 4

WC Oncology; Obstetrics & Gynecology

WE Science Citation Index Expanded (SCI-EXPANDED)

SC Oncology; Obstetrics & Gynecology

GA L9EA5

UT WOS:001026211200001

PM 37329874

DA 2024-01-18

ER

PT J

AU Falcone, F

Lagana, AS

Casarin, J

Chiofalo, B

Barra, F

Garzon, S

Ghezzi, F

Vizza, E

Malzoni, M

AF Falcone, Francesca

Lagana, Antonio Simone

Casarin, Jvan

Chiofalo, Benito

Barra, Fabio

Garzon, Simone

Ghezzi, Fabio

Vizza, Enrico

Malzoni, Mario

TI Evaluation of Peri-Operative Management in Women with Deep Endometriosis

Who are Candidates for Bowel Surgery: A Survey from the Italian Society

of Gynecologic Endoscopy

SO JOURNAL OF MINIMALLY INVASIVE GYNECOLOGY

LA English

DT Article

DE Endometriosis; ERAS; Perioperative management

ID LAPAROSCOPIC COLORECTAL SURGERY; PATIENT-CONTROLLED ANALGESIA;

RANDOMIZED CLINICAL-TRIAL; ENHANCED RECOVERY; CARE; GUIDELINES;

RECOMMENDATIONS

AB Study Objective: There is great consensus that the implementation of the enhanced recovery after surgery (ERAS) approach is beneficial for surgical patients, but there is a paucity of data concerning its application in women with deep endometriosis (DE) who are candidates for bowel surgery. The survey described herein was aimed at gathering detailed information on perioperative management of DE patients who were undergoing sigmoid/rectal (discoid or segmental) resec-tion within the Italian Society of Gynecologic Endoscopy (SEGI) group.Design: Baseline survey.Setting: National survey conducted within the main Italian cooperative group in minimally invasive gynecologic surgery (SEGI).Patients: The study did not involve patients.Interventions: A 63-item questionnaire covering ERAS items for gynecologic/elective colorectal surgery was sent to SEGI centers. Only questionnaires from centers that reported performing & GE;10 sigmoid/rectal resections per year were considered for this analysis.Measurements and Main Results: Thirty-three of 38 (86.8%) of the questionnaires were analyzed. The rates of concor-dance with the ERAS guidelines were 40.4%, 64.4%, and 62.6% for preoperative, intraoperative, and postoperative items, respectively. The proportion of overall agreement was 56.6%. Preoperative diet, fasting and bowel preparation, correction of anemia, avoidance of peritoneal drains, postoperative feeding, and early mobilization were the most controversial items. Comparative analysis revealed that the referred rates of complete disease removal and conversion to open surgery were sig-nificantly different depending on case volume (p = .044 and p = .003, respectively) and gynecologist's/surgeon's experience (p = .042 and p = .022, respectively), with higher chances of obtaining a complete laparoscopic/robotic excision of endome-triosis in centers that reported & GE;30 DE surgeries performed per year and/or & GE;90% of bowel resections performed by a gyne-cologist/general surgeon specifically dedicated to DE management. In contrast, the rates of concordance with the ERAS guidelines were not significantly different according to case volume (p = .081) or gynecologist's/surgeon's experience (p = .294).Conclusion: This is the first study on DE conducted on a national scale. The current survey results revealed suboptimal compliance with the ERAS recommendations and underline the need to improve the quality of perioperative care in DE patients undergoing sigmoid/rectal resection. This study is a first step toward building a consistent, structured reporting platform for the SEGI units and facilitating wide implementation and standardization of the ERAS protocol for DE patients in Italy. Journal of Minimally Invasive Gynecology (2023) 30, 462-472. & COPY; 2023 AAGL. All rights reserved.

C1 [Falcone, Francesca; Malzoni, Mario] Ctr Adv Endoscop Gynecol Surg, Endoscop Malzoni, Via C Errico 2, I-83100 Avellino, Italy.

[Lagana, Antonio Simone] Univ Palermo, Dept Hlth Promot Mother & Child Care, Unit Gynecol Oncol, ARNAS Civico Di Cristina Benfratelli, Palermo, Italy.

[Casarin, Jvan; Ghezzi, Fabio] Univ Insubria, Filippo Ponte Hosp, Dept Obstet & Gynecol, Varese, Italy.

[Chiofalo, Benito; Vizza, Enrico] IRCCS Regina Elena Natl Canc Inst, Dept Expt Clin Oncol, Gynecol Oncol Unit, Rome, Italy.

[Barra, Fabio] PO Osped Tigullio ASL4, Unit Obstet & Gynecol, Metropolitan Area Genoa, Genoa, Italy.

[Barra, Fabio] Univ Genoa, Dept Neurosci Rehabil Ophthalmol Ophthalmol Genet, Genoa, Italy.

[Garzon, Simone] Univ Verona, Dept Obstet & Gynecol, AOUI Verona, Verona, Italy.

C3 University of Palermo; A.R.N.A.S. Ospedali Civico Di Cristina

Benfratelli; University of Insubria; University of Genoa; University of

Verona; Azienda Ospedaliera Universitaria Integrata Verona

RP Falcone, F (corresponding author), Ctr Adv Endoscop Gynecol Surg, Endoscop Malzoni, Via C Errico 2, I-83100 Avellino, Italy.

EM francesca.falcone3@libero.it

RI Chiofalo, Benito/AAD-3286-2020; Laganà, Antonio Simone/E-6466-2015;

Garzon, Simone/A-1991-2019

OI Chiofalo, Benito/0000-0003-2801-2962; Laganà, Antonio

Simone/0000-0003-1543-2802; Falcone, Francesca/0000-0002-3729-2321;

Garzon, Simone/0000-0002-5840-699X

NR 21

TC 1

Z9 1

U1 0

U2 2

PU ELSEVIER SCIENCE INC

PI NEW YORK

PA STE 800, 230 PARK AVE, NEW YORK, NY 10169 USA

SN 1553-4650

EI 1553-4669

J9 J MINIM INVAS GYN

JI J. Minim. Invasive Gynecol.

PD JUN

PY 2023

VL 30

IS 6

BP 462

EP 472

DI 10.1016/j.jmig.2023.01.020

EA JUN 2023

PG 11

WC Obstetrics & Gynecology

WE Science Citation Index Expanded (SCI-EXPANDED)

SC Obstetrics & Gynecology

GA K9FH5

UT WOS:001019417000001

PM 36754274

DA 2024-01-18

ER

PT J

AU Holtzman, S

Stoffels, G

Flint, M

Carr, C

Prasad-Hayes, M

Zeligs, K

Blank, SV

AF Holtzman, Sharonne

Stoffels, Guillaume

Flint, Matt

Carr, Caitlin

Prasad-Hayes, Monica

Zeligs, Kristen

Blank, Stephanie V.

TI Outcomes for patients with high-risk endometrial cancer undergoing

sentinel lymph node assessment versus full lymphadenectomy

SO GYNECOLOGIC ONCOLOGY

LA English

DT Article

DE High-risk endometrial cancer; Sentinel lymph node biopsy

ID SURVIVAL; IMPACT; MULTICENTER; CARCINOMA; BIOPSY

AB Objective. The objective of this study was to determine the progression free survival (PFS) and overall survival (OS) among patients with high-risk endometrial cancer (EC) who underwent sentinel lymph node (SLN) map-ping and dissection compared to patients who underwent pelvic +/- para-aortic lymphadenectomy (LND).Methods. Patients with newly diagnosed high-risk EC were identified. Inclusion criteria included patients who underwent primary surgical management from January 1, 2014 to September 1, 2020 at our institution. Patients were categorized into either the SLN or LND group based on their method of planned lymph node assessment. Patients in the SLN group had dye injected followed by successful bilateral lymph node mapping, retrieval, and processing per our institutional protocol. Clinicopathological and follow-up data were extracted from patient's medical records. The t-test or Mann-Whitney test was used to compare continuous variables and Chi-squared or Fisher's exact test were used for categorical variables. Progression-free survival (PFS) was calculated from the date of initial surgery to the date of progression, death, or last follow-up. Overall survival (OS) was calculated from the date of surgical staging to the date of death or last follow-up. Three-year PFS and OS were calculated using the Kaplan-Meier method, and the log-rank test was used to compare cohorts. Multivariable Cox regression models were used to assess the relationship between nodal assessment cohort and OS/PFS while adjusting for age, adjuvant therapy, and surgical approach. A result was considered statistically significant at the p < 0.05 level of significance and all statistical analysis was done using SAS version 9.4 (SAS Institute, Cary, NC).Results. Out of 674 patients diagnosed with EC during the study period, 189 were diagnosed with high-risk EC based on our criteria. Forty-six (23.7%) patients underwent SLN assessment and 143 (73.7%) underwent LND. No difference was observed between the two groups in regards to age, histology, stage, body mass index, tumors myometrial invasion, lymphovascular space invasion, or peritoneal washing positivity. Patients in the SLN group underwent robotic-assisted procedures more frequently than those in the LND group (p < 0.0001). The three-year PFS rate was 71.1% (95% CI 51.3-84.0%) in the SLN group and 71.3% (95% CI 62.0-78.6%) in the LND group (p = 0.91). The unadjusted hazard ratio (HR) for recurrence in the SLN versus LND group was 1.11 (95% CI 0.56-2.18; p = 0.77), and after adjusting for age, adjuvant therapy, and surgical approach, the HR for recurrence was 1.04 (95% CI 0.47-2.30, p = 0.91). The three-year OS rate was 81.1% (95% CI 51.1-93.7%) in the SLN group and 95.1% (95% CI 89.4-97.8%) in the LND group (p = 0.009). Although the unadjusted HR for death was 3.74 in the SLN vs LND group (95% CI 1.39-10.09; p = 0.009), when adjusted for age, adjuvant therapy, and surgical approach, it was no longer significant with a HR of 2.90 (95% CI 0.94-8.95, p = 0.06).Conclusions. There was no difference in three-year PFS in patients diagnosed with high-risk EC who under-went SLN evaluation compared to those who underwent full LND in our cohort. The SLN group did experience shorter unadjusted OS; however, when adjusting for age, adjuvant therapy and surgical approach, there was no difference OS in patients who underwent SLN compared to LND.& COPY; 2023 Elsevier Inc. All rights reserved.

C1 [Holtzman, Sharonne; Stoffels, Guillaume; Flint, Matt; Carr, Caitlin; Prasad-Hayes, Monica; Zeligs, Kristen; Blank, Stephanie V.] Icahn Sch Med Mt Sinai, Dept Obstet Gynecol & Reprod Sci, New York, NY 10029 USA.

C3 Icahn School of Medicine at Mount Sinai

RP Holtzman, S (corresponding author), Icahn Sch Med Mt Sinai, Dept Obstet Gynecol & Reprod Sci, New York, NY 10029 USA.

EM sharonne.holtzman@mountsinai.org

NR 20

TC 1

Z9 1

U1 1

U2 1

PU ACADEMIC PRESS INC ELSEVIER SCIENCE

PI SAN DIEGO

PA 525 B ST, STE 1900, SAN DIEGO, CA 92101-4495 USA

SN 0090-8258

EI 1095-6859

J9 GYNECOL ONCOL

JI Gynecol. Oncol.

PD JUL

PY 2023

VL 174

BP 273

EP 277

DI 10.1016/j.ygyno.2023.05.002

EA JUN 2023

PG 5

WC Oncology; Obstetrics & Gynecology

WE Science Citation Index Expanded (SCI-EXPANDED)

SC Oncology; Obstetrics & Gynecology

GA K2FG7

UT WOS:001014644500001

PM 37270906

DA 2024-01-18

ER

PT J

AU Eissa, A

Sighinolfi, MC

Elsodany, I

Habib, G

Puliatti, S

Zoeir, A

Elsherbiny, A

Abo-Elenien, M

Mousa, A

Elbendary, M

Radwan, M

Gaia, G

Elbahnasy, AH

Micali, S

Rocco, B

AF Eissa, Ahmed

Sighinolfi, Maria Chiara

Elsodany, Ibrahim

Habib, George

Puliatti, Stefano

Zoeir, Ahmed

Elsherbiny, Ahmed

Abo-Elenien, Mohamed

Mousa, Ayman

Elbendary, Mohamed

Radwan, Mohamed

Gaia, Giorgia

Elbahnasy, Abdel Hamid

Micali, Slavatore

Rocco, Bernardo

TI Robotic Pelvic Lymphadenectomy in Gynecological and Urological

Malignancies

SO CLINICAL AND EXPERIMENTAL OBSTETRICS & GYNECOLOGY

LA English

DT Review

DE pelvic lymph node dissection; robotic surgery; bladder cancer; cervical

cancer; endometrial cancer; prostate cancer

ID LYMPH-NODE DISSECTION; OPEN RADICAL CYSTECTOMY; STAGE CERVICAL-CANCER;

ENDOMETRIAL CANCER; PROSTATE-CANCER; BLADDER-CANCER; SENTINEL NODE;

ONCOLOGICAL OUTCOMES; SURGICAL OUTCOMES; INDOCYANINE GREEN

AB Objectives: Pelvic lymphadenectomy is a crucial step in the management of different pelvic cancers for both prognostic and/or therapeutic goals. Robotic surgeries offered numerous benefits over open and/or laparoscopic surgeries such as better visualization, shorter hospital stay, less pain and better cosmoses. The aim of this narrative review is to evaluate the value and outcomes of robotic pelvic lymph node dissection (PLND).Mechanism: The PubMed database was searched using the following keywords "Robotic" AND "pelvic lymph node dissection" to identify all the relevant articles concerned with the role and outcomes of robotic PLND. We included only English articles published between 2010 and 2022. Data from the retrieved articles were then used to formulate this review that highlight the introduction, the outcomes of robotic pelvic lymph node dissection (PLND), and the mapping of sentinel lymph node (SLN) in cervical, endometrial, prostate, and bladder cancers.Findings in Brief: PLND is an integral part of gynecological and urological oncology for its role in tumor staging and planning of further treatment plan. Furthermore, it may play an important therapeutic role in bladder cancer. Robotic approach to PLND is safe and efficient and can be potentially used for cervical, endometrial, prostate, and bladder cancers.Conclusions: Robotic PLND could be an alternative to open and laparoscopic approaches as it may decrease the associated morbidities without compromising the quality of Lymph node dissection (LND).

C1 [Eissa, Ahmed; Elsodany, Ibrahim; Habib, George; Zoeir, Ahmed; Elsherbiny, Ahmed; Abo-Elenien, Mohamed; Mousa, Ayman; Elbendary, Mohamed; Radwan, Mohamed; Elbahnasy, Abdel Hamid] Tanta Univ, Fac Med, Urol Dept, Tanta 31527, Egypt.

[Sighinolfi, Maria Chiara; Rocco, Bernardo] Univ Milan, Urol Dept, ASST St Paolo & Carlo, I-20142 Milan, Italy.

[Puliatti, Stefano; Micali, Slavatore] Univ Modena & Reggio Emilia, Urol Dept, I-41126 Modena, Italy.

[Puliatti, Stefano] ORSI Acad, B-9090 Melle, Belgium.

[Gaia, Giorgia] ASST St Paolo & Carlo, Obstet & Gynecol Dept, I-20142 Milan, Italy.

C3 Egyptian Knowledge Bank (EKB); Tanta University; University of Milan;

Universita di Modena e Reggio Emilia

RP Eissa, A (corresponding author), Tanta Univ, Fac Med, Urol Dept, Tanta 31527, Egypt.

EM ahmed.essa@med.tanta.edu.eg

RI Eissa, Ahmed/K-2328-2019

OI Eissa, Ahmed/0000-0001-6817-6887

NR 112

TC 0

Z9 0

U1 2

U2 2

PU IMR PRESS

PI ROBINSON

PA 112 ROBINSON RD, ROBINSON, SINGAPORE

SN 0390-6663

EI 2709-0094

J9 CLIN EXP OBSTET GYN

JI Clin. Exp. Obstet. Gynecol.

PD JUN

PY 2023

VL 50

IS 6

AR 5006123

DI 10.31083/j.ceog5006123

PG 11

WC Obstetrics & Gynecology

WE Science Citation Index Expanded (SCI-EXPANDED)

SC Obstetrics & Gynecology

GA M0AP6

UT WOS:001026813500006

OA gold

DA 2024-01-18

ER

PT J

AU Sia, TY

Basaran, D

Dagher, C

Sassine, D

Brandt, B

Rosalik, K

Mueller, JJ

Broach, V

Makker, V

Soslow, RA

Abu-Rustum, NR

Leitao, MM

AF Sia, Tiffany Y.

Basaran, Derman

Dagher, Christian

Sassine, Dib

Brandt, Benny

Rosalik, Kendall

Mueller, Jennifer J.

Broach, Vance

Makker, Vicky

Soslow, Robert A.

Abu-Rustum, Nadeem R.

Leitao, Mario M., Jr.

TI Laparoscopy with or without robotic assistance does not negatively

impact long-term oncologic outcomes in patients with uterine serous

carcinoma

SO GYNECOLOGIC ONCOLOGY

LA English

DT Article

DE Laparotomy; Minimally invasive surgery; Uterine serous carcinoma

ID ENDOMETRIAL CANCER; ABDOMINAL HYSTERECTOMY; RISK; SURVIVAL; SURGERY;

WOMEN

AB Objectives. We sought to compare outcomes between minimally invasive surgery (MIS) and laparotomy in patients with clinical stage I uterine serous carcinoma (USC).

Methods. Patients who underwent surgery for newly diagnosed USC between 11/1/1993 and 12/31/2017 were retrospectively identified and assigned to either the MIS cohort or the laparotomy cohort. Patients with conversion to laparotomy were analyzed with the MIS cohort. Chi-square and Mann-Whitney tests were used to compare categorical and continuous variables, respectively. Kaplan-Meier curves were used to estimate survival and compared using the log-rank test.

Results. In total, 391 patients met inclusion criteria; 242 underwent MIS (35% non-robotic and 65% robotic-assisted laparoscopies) and 149 underwent laparotomy. Age, BMI, stage, and washings status did not differ between cohorts. Patients who underwent MIS were less likely to have lymphovascular space invasion (LVSI; 35.1% vs 48.3%), had fewer nodes removed (median, 9 vs 15), and lower rates of paraaortic nodal dissection (44.6% vs 65.1%). Rates of adjuvant therapy did not differ between cohorts. Median follow-up timeswere 63.0 months (MIS cohort) vs 71.0 months (laparotomy cohort; P=.04). Five-year PFS rates were 58.7% (MIS) vs 59.8% (laparotomy; P =.1). Five-year OS rates were 65.2% (MIS) compared to 63.5% (laparotomy; P =.2). On multivariable analysis, higher stage, deep myometrial invasion, and positive washings were associated with decreased PFS. Age >= 65 years, higher stage, LVSI, and positive washings were associated with shorter OS.

Conclusions. MIS does not compromise outcomes in patients with newly diagnosed USC and should be offered to these patients to minimize surgical morbidity. (c) 2023 Elsevier Inc. All rights reserved.

C1 [Sia, Tiffany Y.; Basaran, Derman; Dagher, Christian; Sassine, Dib; Brandt, Benny; Rosalik, Kendall; Mueller, Jennifer J.; Broach, Vance; Abu-Rustum, Nadeem R.; Leitao, Mario M., Jr.] Mem Sloan Kettering Canc Ctr, Dept Surg, Gynecol Serv, New York, NY USA.

[Mueller, Jennifer J.; Broach, Vance; Abu-Rustum, Nadeem R.; Leitao, Mario M., Jr.] Weill Cornell Med Coll, Dept OB GYN, New York, NY USA.

[Makker, Vicky] Mem Sloan Kettering Canc Ctr, Dept Med, New York, NY USA.

[Makker, Vicky] Weill Cornell Med Coll, Dept Med, New York, NY USA.

[Soslow, Robert A.] Mem Sloan Kettering Canc Ctr, Dept Pathol, New York, NY USA.

[Leitao, Mario M., Jr.] Mem Sloan Kettering Canc Ctr, Dept Surg, Gynecol Serv, 1275 York Ave, New York, NY 10065 USA.

C3 Memorial Sloan Kettering Cancer Center; Cornell University; Weill

Cornell Medicine; Memorial Sloan Kettering Cancer Center; Cornell

University; Weill Cornell Medicine; Memorial Sloan Kettering Cancer

Center; Memorial Sloan Kettering Cancer Center

RP Leitao, MM (corresponding author), Mem Sloan Kettering Canc Ctr, Dept Surg, Gynecol Serv, 1275 York Ave, New York, NY 10065 USA.

EM leitaom@mskcc.org

RI BASARAN, Derman/I-9648-2013; Dagher, Christian/JQW-7746-2023

OI BASARAN, Derman/0000-0002-2689-1417; Rosalik, Kendal/0000-0002-1749-6386

FU National Institutes of Health (NIH) /National Cancer Institute (NCI)

Cancer Center Support Grant [P30 CA008748]

FX Funding This work was supported in part by a National Institutes of

Health (NIH) /National Cancer Institute (NCI) Cancer Center Support

Grant (P30 CA008748) .

NR 26

TC 0

Z9 0

U1 3

U2 3

PU ACADEMIC PRESS INC ELSEVIER SCIENCE

PI SAN DIEGO

PA 525 B ST, STE 1900, SAN DIEGO, CA 92101-4495 USA

SN 0090-8258

EI 1095-6859

J9 GYNECOL ONCOL

JI Gynecol. Oncol.

PD AUG

PY 2023

VL 175

BP 8

EP 14

DI 10.1016/j.ygyno.2023.05.064

EA MAY 2023

PG 7

WC Oncology; Obstetrics & Gynecology

WE Science Citation Index Expanded (SCI-EXPANDED)

SC Oncology; Obstetrics & Gynecology

GA K5AT7

UT WOS:001016572000001

PM 37267674

DA 2024-01-18

ER

PT J

AU Netter, A

Litaudon, C

Tourette, C

Miquel, L

Courbiere, B

Agostini, A

AF Netter, Antoine

Litaudon, Charlotte

Tourette, Claire

Miquel, Laura

Courbiere, Blandine

Agostini, Aubert

TI Robot-assisted tubo-tubal reanastomosis after sterilization in 10 steps

SO JOURNAL OF GYNECOLOGY OBSTETRICS AND HUMAN REPRODUCTION

LA English

DT Article

DE Robot -assisted laparoscopy; Tubo-tubal reanastomosis; Sterilization

ID ANASTOMOSIS; REVERSAL

AB Five to 20% of women regret having a tubal ligation. These women are generally otherwise fertile and have a better chance of pregnancy than other patients experiencing infertility, whether by in vitro fertilization or after tubal surgery. Historically, tubal anastomosis surgery has long been performed by microsurgery through laparotomy, which provided very high precision but was associated with some degree of morbidity. The parallel development of in vitro fertilization and laparoscopy have contributed to reducing the indica-tions for tubal surgery. The laparoscopic approach is challenging because of the number and precision of the sutures needed. The robot-assisted laparoscopic approach may reduce the surgical difficulty and improve the accessibility of this technique. We have described the technique of tubo-tubal reanastomosis after steriliza-tion with robot-assisted laparoscopy in 10 steps. Robot-assisted laparoscopy provides favourable conditions for performing tubo-tubal reanastomosis after sterilization due to the camera stability, precision of move-ment, and amplitude of articulations.& COPY; 2023 Elsevier Masson SAS. All rights reserved.

C1 [Netter, Antoine; Litaudon, Charlotte; Tourette, Claire; Miquel, Laura; Courbiere, Blandine; Agostini, Aubert] Aix Marseille Univ, Concept Hosp, AP HM, Dept Obstet & Gynecol, F-13005 Marseille, France.

[Netter, Antoine; Courbiere, Blandine] Aix Marseille Univ, Avignon Univ, Inst Mediterraneen Biodivers & Ecol Marine & Cont, CNRS,IRD, Marseille, France.

C3 Aix-Marseille Universite; Assistance Publique-Hopitaux de Marseille;

Avignon Universite; Centre National de la Recherche Scientifique (CNRS);

Institut de Recherche pour le Developpement (IRD); Aix-Marseille

Universite

RP Netter, A (corresponding author), AP HM Concept, Dept Gynecol & Obstet, 147 Bd Baille, F-13005 Marseille, France.

EM antoine.netter@gmail.com

RI Netter, Antoine/ABB-8570-2020

OI Netter, Antoine/0000-0002-3532-0800; MIQUEL, Laura/0000-0002-0844-3370

NR 11

TC 0

Z9 0

U1 4

U2 4

PU ELSEVIER MASSON, CORP OFF

PI PARIS

PA 65 CAMILLE DESMOULINS CS50083 ISSY-LES-MOULINEAUX, 92442 PARIS, FRANCE

SN 2468-7847

EI 1773-0430

J9 J GYNECOL OBSTET HUM

JI J. Gynecol. Obstet. Hum. Reprod.

PD JUN

PY 2023

VL 52

IS 6

AR 102605

DI 10.1016/j.jogoh.2023.102605

EA MAY 2023

PG 2

WC Obstetrics & Gynecology

WE Science Citation Index Expanded (SCI-EXPANDED)

SC Obstetrics & Gynecology

GA J9BL2

UT WOS:001012504800001

PM 37210010

OA Green Published

DA 2024-01-18

ER

PT J

AU Lecoanet, P

Madanelo, M

Tricard, T

de Varennes, AM

Haudebert, C

Richard, C

Hascoet, J

Bentellis, I

Tibi, B

Saussine, C

Hubert, J

Peyronnet, B

AF Lecoanet, Pierre

Madanelo, Mariana

Tricard, Thibault

Mauger de Varennes, Anne

Haudebert, Camille

Richard, Claire

Hascoet, Juliette

Bentellis, Imad

Tibi, Branwell

Saussine, Christian

Hubert, Jacques

Peyronnet, Benoit

TI Robot-assisted vesicovaginal fistula repair: comparison of the

extravesical and transvesical techniques

SO INTERNATIONAL UROGYNECOLOGY JOURNAL

LA English

DT Article

DE Robot-assisted surgery; Fistula; Vesicovaginal; Multicenter study

ID SURGICAL REPAIR

AB Introduction and objectivesAlmost two decades after the description of robotic vesicovaginal fistula repair (R-VVF), the literature remains limited. The aims of this study are to report the outcomes of R-VVF and to compare the transvesical versus extravesical techniques.MethodsWe performed an observational, retrospective, multicenter study, including all patients who underwent R-VVF from March 2017 to September 2021 at four academic institutions. All abdominal VVF repair over the study period were performed using a robotic approach. The success of R-VVF was defined as the absence of clinical recurrence. The outcomes of the extravesical versus transvesical techniques were compared.ResultsTwenty-two patients were included. The median age was 43 years old (IQR 38-50). Fistulas were supratrigonal and trigonal in 18 and 4 cases respectively. Five patients had undergone previous attempts of fistula repair (22.7%). The fistulous tract was systematically excised, and an interposition flap was used in all but two cases (90.9%). The transvesical and extravesical techniques were used in 13 and 9 cases respectively. There were four postoperative complications, three minor and one major. None of the patients had vesicovaginal fistula recurrence after a median follow-up of 15 months.ConclusionsThe present series, one of the largest R-VVF reported to date, is consistent with the few series already published with a 100% cure rate. Systematic excision of the fistulous tract and the high rate of flap interposition may explain the high success rate. The transvesical and extravesical approaches yielded similar outcomes.

C1 [Lecoanet, Pierre; Hubert, Jacques] Univ Nancy, Dept Urol, Nancy, France.

[Madanelo, Mariana] Ctr Hosp Univ Santo Antonio, Dept Urol, Porto, Portugal.

[Tricard, Thibault; Saussine, Christian] Univ Strasbourg, Dept Urol, Strasbourg, France.

[Mauger de Varennes, Anne; Haudebert, Camille; Richard, Claire; Hascoet, Juliette; Peyronnet, Benoit] Univ Rennes, Dept Urol, Rennes, France.

[Bentellis, Imad; Tibi, Branwell] Univ Nice, Dept Urol, Nice, France.

C3 Universite de Lorraine; Universites de Strasbourg Etablissements

Associes; Universite de Strasbourg; Universite de Rennes; Universite

Cote d'Azur

RP Madanelo, M (corresponding author), Ctr Hosp Univ Santo Antonio, Dept Urol, Porto, Portugal.

EM marianacmadanelo@gmail.com

OI Madanelo, Mariana/0000-0001-8429-7785

NR 31

TC 0

Z9 0

U1 0

U2 0

PU SPRINGER LONDON LTD

PI LONDON

PA 236 GRAYS INN RD, 6TH FLOOR, LONDON WC1X 8HL, ENGLAND

SN 0937-3462

EI 1433-3023

J9 INT UROGYNECOL J

JI Int. Urogynecol. J.

PD OCT

PY 2023

VL 34

IS 10

BP 2479

EP 2485

DI 10.1007/s00192-023-05565-7

EA MAY 2023

PG 7

WC Obstetrics & Gynecology; Urology & Nephrology

WE Science Citation Index Expanded (SCI-EXPANDED)

SC Obstetrics & Gynecology; Urology & Nephrology

GA U9KR4

UT WOS:000990937400001

PM 37204473

DA 2024-01-18

ER

PT J

AU Baker, MV

Zhao, ZG

Murarka, SM

Adam, RA

Prescott, LS

AF Baker, Mary V.

Zhao, Zhiguo

Murarka, Shivani M.

Adam, Rony A.

Prescott, Lauren S.

TI COVID-19 as a Catalyst for Same Day Discharge After Minimally Invasive

Hysterectomy

SO JOURNAL OF GYNECOLOGIC SURGERY

LA English

DT Article

DE gynecology; gynecologic surgery; outpatient surgery; coronavirus; health

care utilization; enhanced recovery

ID SURGERY

AB Objective: This study quantified the COVID-19 pandemic's impact on same-day discharges for minimally invasive hysterectomy and evaluated the effect on postoperative morbidity and health care use.Materials and Methods: This retrospective cohort study, from March 2018 to October 2021 at a single institution, included women older than age 18 who had laparoscopic, vaginal, or robotic-assisted hysterectomy by any gynecologic surgeon. Primary outcome was rate of same-day hospital discharge. Secondary measures were length of stay and rates of 30-day postoperative morbidity and health care use. Univariate and multivariable logistic regression analyses were conducted to evaluate associations between patients' characteristics and likelihood of same-day discharge.Results: There were 1608 women included, 896 in a prepandemic cohort and 712 in a postpandemic cohort. Surgeon subspecialty rates were similar between groups, but surgical approaches differed, with more laparoscopic procedures in the postpandemic cohort (p = 0.007). Case order and lengths, and concurrent procedures were not different between groups. Postpandemic patients were more likely to be discharged on the same day even after controlling for confounders in a multivariable regression (32% versus 54%, respectively; odds ratio: 2.78; p < 0.001). Rates of 30-day postoperative complications, transfusions, emergency department visits, readmissions, reoperations, and mortality were not significantly different.Conclusions: The COVID-19 pandemic was associated with increased same-day discharges without increases in 30-day postoperative complications. The data confirmed that same-day discharge following minimally invasive hysterectomy was safe for managing hospital constraints caused by the COVID-19 pandemic. (J GYNECOL SURG 20XX:000)

C1 [Baker, Mary V.; Murarka, Shivani M.; Adam, Rony A.; Prescott, Lauren S.] Vanderbilt Univ, Dept Obstet & Gynecol, Med Ctr, Nashville, TN USA.

[Zhao, Zhiguo] Vanderbilt Univ, Dept Biostat, Med Ctr, Nashville, TN USA.

[Baker, Mary V.] 1161 21st Ave South,B1124 MCN, Nashville, TN 37232 USA.

C3 Vanderbilt University; Vanderbilt University

RP Baker, MV (corresponding author), 1161 21st Ave South,B1124 MCN, Nashville, TN 37232 USA.

EM Mary.vm.baker@vumc.org

NR 23

TC 0

Z9 0

U1 0

U2 0

PU MARY ANN LIEBERT, INC

PI NEW ROCHELLE

PA 140 HUGUENOT STREET, 3RD FL, NEW ROCHELLE, NY 10801 USA

SN 1042-4067

EI 1557-7724

J9 J GYNECOL SURG

JI J. Gynecol. Surg.

PD AUG 1

PY 2023

VL 39

IS 4

BP 170

EP 176

DI 10.1089/gyn.2023.0014

EA MAY 2023

PG 7

WC Obstetrics & Gynecology; Surgery

WE Emerging Sources Citation Index (ESCI)

SC Obstetrics & Gynecology; Surgery

GA N6RC6

UT WOS:000986981200001

DA 2024-01-18

ER

PT J

AU Farah, S

Albaini, O

Al Jardali, M

Daccache, A

Jallad, K

AF Farah, Stephanie

Albaini, Obey

Al Jardali, Marwa

Daccache, Aimee

Jallad, Karl

TI The Feasibility and Safety of vNOTES Hysterectomy and Uterosacral

Ligament Suspension: A Case Series

SO JOURNAL OF MINIMALLY INVASIVE GYNECOLOGY

LA English

DT Article

DE Feasibility; Hysterectomy; Safety; Uterosacral; vNOTES

ID TRANSLUMINAL ENDOSCOPIC SURGERY; SALPINGO-OOPHORECTOMY; LIFETIME RISK;

ADNEXECTOMY

AB The transvaginal natural orifice transluminal endoscopic surgery (vNOTES) is a recently introduced surgical approach that is even less invasive than conventional laparoscopy or robotic surgery. We conducted this study to report our experience in vNOTES hysterectomy and uterosacral ligament suspension and determine the feasibility and safety of this approach. Surgeries on 23 women were performed by a single surgeon in 1 tertiary medical center. Patient demographics, perioperative data, and follow-up details of 23 women were collected prospectively. Average age was 56.7 + 8.9 years. Median parity was 3. Nine patients were smokers, and 4 patients had diabetes. Median stage of prolapse was 3. One patient had extensive adhesions, and after vNOTES hysterectomy was completed, decision was made to perform uterosacral suspension by conventional vaginal access. Another patient had intraoperative identification by cystoscopy of unilateral kinking of the ureter that was resolved after the most distal uterosacral stitch was released. Mean uterine weight was 271.9 + 131.9 g. Average estimated blood loss was 85.22 + 55.6 mL. Median length of stay in the hospital was 1 day. Only 1 patient had intermittent voiding postoperatively and required an indwelling catheter for 3 days. Hysterectomy and uterosacral ligament suspension when performed via vNOTES is a safe and feasible procedure. Large prospective trials are on the way to continue shedding light on this new surgical modality. Journal of Minimally Invasive Gynecology (2023) 30, 414-417. & COPY; 2023 AAGL. All rights reserved.

C1 [Farah, Stephanie; Albaini, Obey] Lebanese Amer Univ, Lebanese Amer Univ Med Ctr LAUMCRH, Gilbert & Rose Marie Chagoury Sch Med, Beirut, Lebanon.

[Al Jardali, Marwa; Daccache, Aimee] Lebanese Amer Univ, Lebanese Amer Univ Med Ctr LAUMCRH, Gilbert & Rose Marie Chagoury Sch Med, Beirut, Lebanon.

[Jallad, Karl] Lebanese Amer Univ, Lebanese Amer Univ Med Ctr LAUMCR, Obstet Gynecol & Surg, Gilbert & Rose Marie Chagoury Sch Med, Beirut, Lebanon.

[Jallad, Karl] Lebanese Amer Univ, Lebanese Amer Univ Med Ctr LAUMCRH, Gilbertand Rose Marie Chagoury Sch Med, Gynecol & Surg, Beirut, Lebanon.

C3 Lebanese American University; Lebanese American University; Lebanese

American University; Lebanese American University

RP Jallad, K (corresponding author), Lebanese Amer Univ, Lebanese Amer Univ Med Ctr LAUMCRH, Gilbertand Rose Marie Chagoury Sch Med, Gynecol & Surg, Beirut, Lebanon.

EM karljallad@gmail.com

NR 28

TC 2

Z9 3

U1 2

U2 2

PU ELSEVIER SCIENCE INC

PI NEW YORK

PA STE 800, 230 PARK AVE, NEW YORK, NY 10169 USA

SN 1553-4650

EI 1553-4669

J9 J MINIM INVAS GYN

JI J. Minim. Invasive Gynecol.

PD MAY

PY 2023

VL 30

IS 5

BP 414

EP 417

DI 10.1016/j.jmig.2023.01.005

EA MAY 2023

PG 4

WC Obstetrics & Gynecology

WE Science Citation Index Expanded (SCI-EXPANDED)

SC Obstetrics & Gynecology

GA J9LW6

UT WOS:001012778600001

PM 36646312

DA 2024-01-18

ER

PT J

AU Fu, HL

Zhang, JH

Zhao, SY

He, NN

AF Fu, Hanlin

Zhang, Jiahui

Zhao, Shiyi

He, Nannan

TI Survival outcomes of robotic-assisted laparoscopy versus conventional

laparoscopy and laparotomy for endometrial cancer: A systematic review

and meta-analysis

SO GYNECOLOGIC ONCOLOGY

LA English

DT Review

DE Endometrial cancer; Robotic-assisted laparoscopy; Long-term survival;

Conventional laparoscopy; Laparotomy

ID OPEN SURGERY; HYSTERECTOMY; WOMEN; CARCINOMA; OBESE; MANAGEMENT; COST

AB Objective. Robotic-assisted laparoscopy (RALS) has gained widespread acceptance in the field of gynecologi-cal oncology. However, whether the prognosis of endometrial cancer after RALS is superior to conventional lap-aroscopy (CLS) and laparotomy (LT) remains inconclusive. Therefore, the aim of this meta-analysis was to compare the long-term survival outcomes of RALS with CLS and LT for endometrial cancer.Methods. A systematic literature search was conducted on electronic databases (PubMed, Cochrane, EMBASE and Web of Science) until May 24, 2022, followed by a manual search. Based on inclusion and exclusion criteria, publications investigating long-term survival outcomes after RALS vs CLS or LT in endometrial cancer patients were collected. The primary outcomes included overall survival (OS), disease-specific survival (DSS), recurrence-free survival (RFS) and disease-free survival (DFS). Fixed effects models or random effects models were employed to calculate the pooled hazard ratios (HRs) and 95% confidence intervals (CIs) as appropriate. Heterogeneity and publication bias were also assessed.Results. RALS and CLS had no difference in OS (HR = 0.962, 95% CI: 0.922-1.004), RFS (HR = 1.096, 95% CI: 0.947-1.296), and DSS (HR = 1.489, 95% CI: 0.713-3.107) for endometrial cancer; however, RALS was signifi-cantly associated with favorable OS (HR = 0.682, 95% CI: 0.576-0.807), RFS (HR = 0.793, 95% CI: 0.653-0.964), and DSS (HR = 0.441, 95% CI: 0.298-0.652) when compared with LT. In the subgroup analysis of effect measures and follow-up length, RALS showed comparable or superior RFS/OS to CLS and LT. In early-stage endometrial cancer patients, RALS had similar OS but worse RFS than CLS.Conclusions. RALS is safe in the management of endometrial cancer, with long-term oncological outcomes equivalent to CLS and superior to LT.(c) 2023 The Authors. Published by Elsevier Inc. This is an open access article under the CC BY-NC-ND license (http:// creativecommons.org/licenses/by-nc-nd/4.0/).

C1 [Fu, Hanlin; Zhang, Jiahui; Zhao, Shiyi; He, Nannan] Zhengzhou Univ, Dept Gynecol, Affiliated Hosp 1, Zhengzhou, Peoples R China.

[He, Nannan] Zhengzhou Univ, Dept Gynecol, Affiliated Hosp 1, 1 Jianshe East Rd, Zhengzhou 450052, Henan Province, Peoples R China.

C3 Zhengzhou University; Zhengzhou University

RP He, NN (corresponding author), Zhengzhou Univ, Dept Gynecol, Affiliated Hosp 1, Zhengzhou, Peoples R China.; He, NN (corresponding author), Zhengzhou Univ, Dept Gynecol, Affiliated Hosp 1, 1 Jianshe East Rd, Zhengzhou 450052, Henan Province, Peoples R China.

EM nhe@zzu.edu.cn

FU Medical Science and Technology Re-search Plan Joint Construction Project

of Henan Province [LHGJ20190125, LHGJ20210352]; Startup Research Fund of

Zhengzhou University [32213014]

FX Acknowledgements This work was supported by the Medical Science and

Technology Re-search Plan Joint Construction Project of Henan Province

(grant num-bers LHGJ20190125 and LHGJ20210352) ; and the Startup

Research Fund of Zhengzhou University (grant number 32213014) .

NR 50

TC 2

Z9 2

U1 2

U2 3

PU ACADEMIC PRESS INC ELSEVIER SCIENCE

PI SAN DIEGO

PA 525 B ST, STE 1900, SAN DIEGO, CA 92101-4495 USA

SN 0090-8258

EI 1095-6859

J9 GYNECOL ONCOL

JI Gynecol. Oncol.

PD JUL

PY 2023

VL 174

BP 55

EP 67

DI 10.1016/j.ygyno.2023.04.026

EA MAY 2023

PG 13

WC Oncology; Obstetrics & Gynecology

WE Science Citation Index Expanded (SCI-EXPANDED)

SC Oncology; Obstetrics & Gynecology

GA I1PE2

UT WOS:001000564700001

PM 37149906

OA hybrid

DA 2024-01-18

ER

PT J

AU Piedimonte, S

Helpman, L

Pond, G

Nelson, G

Kwon, J

Altman, A

Feigenberg, T

Elit, L

Lau, S

Sabourin, J

Samouelian, V

Willows, K

Aubrey, C

Jang, JH

-Fortin, LAT

Cockburn, N

Saunders, NB

Shamiya, S

Vicus, D

Plante, M

AF Piedimonte, Sabrina

Helpman, Limor

Pond, Gregory

Nelson, Gregg

Kwon, Janice

Altman, Alon

Feigenberg, Tomer

Elit, Laurie

Lau, Susie

Sabourin, Jeanelle

Samouelian, Vanessa

Willows, Karla

Aubrey, Christa

Jang, Ji-Hyun

-Fortin, Ly-Ann Teo

Cockburn, Norah

Saunders, Nora -Beth

Shamiya, Sarah

Vicus, Danielle

Plante, Marie

TI Surgical margin status in relation to surgical approach in the

management of early-stage cervical Cancer: A Canadian cervical Cancer

collaborative (4C) study

SO GYNECOLOGIC ONCOLOGY

LA English

DT Article

DE Early cervical cancer; MIS; Surgical margin; Survival

ID RADICAL SURGERY; HYSTERECTOMY

AB Objective. Surgical margin status in women undergoing surgery for early-stage cervical cancer is an important prognostic factor. We sought to determine whether close (<3 mm) and positive surgical margins are associated with surgical approach and survival. Methods. This is a national retrospective cohort study of cervical cancer patients treated with radical hysterectomy. Patients with stage IA1/LVSI-Ib2(FIGO 2018) with lesions up to 4 cm at 11 Canadian institutions from 2007 to 2019 were included. Surgical approach included robotic/laparoscopic (LRH), abdominal (ARH) or combined laparoscopic-assisted vaginal/vaginal (LVRH) radical hysterectomy. Recurrence free survival(RFS) and overall survival (OS) were estimated using Kaplan-Meier analysis. Chi-square and log-rank tests were used to compare groups.Results. 956 patients met inclusion criteria. Surgical margins were as follows: negative (87.0%), positive (0.4%) or close <3 mm (6.8%), missing (5.8%). Most patients had squamous histology (46.9%); 34.6% had adenocarcinomas and 11.3% adenosquamous. Most were stage IB (75.1%) and 24.9% were IA. Mode of surgery included: LRH Predictive factors for close/positive margins included stage, tumour diameter, vaginal involvement and parametrial extension. Surgical approach was not associated with margin status (p = 0.27). Close/positivemargins were associated with a higher risk of death on univariate analysis (HR = non calculable for positive and HR = 1.83 for close margins, p = 0.017), but not significant for OS when adjusted for stage, histology, surgical approach and adjuvant treatment. There were 7 recurrences in patients with close margins (10.3%, p = 0.25). 71.5% with positive/close margins received adjuvant treatment. In addition, MIS was associated with a higher risk of death (OR = 2.39, p = 0.029).Conclusion. Surgical approach was not associated to close or positive margins. Close surgical margins were associated with a higher risk of death. MIS was associated with worse survival, suggesting that margin status may not be the driver of worse survival in these cases. (c) 2023 Elsevier Inc. All rights reserved.

C1 [Helpman, Limor; Pond, Gregory; Elit, Laurie; Cockburn, Norah] Univ Toronto, Div Gynecol Oncol, Toronto, ON, Canada.

[Nelson, Gregg] McMaster Univ, Juravinski Canc Ctr, Dept Biostat, Hamilton Hlth Sci, Hamilton, ON, Canada.

[Kwon, Janice; Jang, Ji-Hyun] Univ Calgary, Div Gynecol Oncol, Calgary, AB, Canada.

[Altman, Alon; Saunders, Nora -Beth] Univ British Columbia, Div Gynecol Oncol, Vancouver, BC, Canada.

[Feigenberg, Tomer] Univ Manitoba, Div Gynecol Oncol, Winnipeg, MB, Canada.

[Lau, Susie] Univ Toronto, Div Gynecol Oncol, Trillium Hlth partners, Toronto, ON, Canada.

[Sabourin, Jeanelle; Aubrey, Christa; Shamiya, Sarah] McGill Univ, Div Gynecol Oncol, Montreal, PQ, Canada.

[Samouelian, Vanessa] Univ Alberta, Div Gynecol Oncol, Edmonton, AB, Canada.

[Willows, Karla] Univ Montreal, Div Gynecol Oncol, Montreal, PQ, Canada.

[Vicus, Danielle] Univ Halifax, Div Gynecol Oncol, Halifax, NS, Canada.

[-Fortin, Ly-Ann Teo; Vicus, Danielle; Plante, Marie] Univ Toronto, Sunnybrook Hlth Sci Ctr, Div Gynecol Oncol, Toronto, ON, Canada.

[Plante, Marie] Laval Univ, Div Gynecol Oncol, Quebec City, PQ, Canada.

[Plante, Marie] Ctr Hosp Univ Quebec, 11 Cote Palais, Quebec City, PQ G1R 2J6, Canada.

C3 University of Toronto; McMaster University; University of Calgary;

University of British Columbia; University of Manitoba; University of

Toronto; Trillium Health Partners; McGill University; University of

Alberta; Universite de Montreal; University of Toronto; Sunnybrook

Health Science Center; Sunnybrook Research Institute; Laval University;

Laval University

RP Plante, M (corresponding author), Ctr Hosp Univ Quebec, 11 Cote Palais, Quebec City, PQ G1R 2J6, Canada.

EM marie.plante.med@ssss.gouv.qc.ca

RI Nelson, Gregg/HCH-9224-2022

OI Nelson, Gregg/0000-0002-8177-1098; Pond, Gregory/0000-0003-1033-0882;

Altman, Alon/0000-0002-6345-4910

NR 15

TC 0

Z9 0

U1 1

U2 1

PU ACADEMIC PRESS INC ELSEVIER SCIENCE

PI SAN DIEGO

PA 525 B ST, STE 1900, SAN DIEGO, CA 92101-4495 USA

SN 0090-8258

EI 1095-6859

J9 GYNECOL ONCOL

JI Gynecol. Oncol.

PD JUL

PY 2023

VL 174

BP 21

EP 27

DI 10.1016/j.ygyno.2023.03.005

EA MAY 2023

PG 7

WC Oncology; Obstetrics & Gynecology

WE Science Citation Index Expanded (SCI-EXPANDED)

SC Oncology; Obstetrics & Gynecology

GA I3QJ1

UT WOS:001001954800001

PM 37146436

DA 2024-01-18

ER

PT J

AU Reitz, L

Castel, M

Boukerrou, M

Tran, PL

AF Reitz, Laurianne

Castel, Mathilde

Boukerrou, Malik

Tran, Phuong Lien

TI Robotic surgery implementation in an isolated overseas territory-The

case of Reunion Island

SO JOURNAL OF GYNECOLOGY OBSTETRICS AND HUMAN REPRODUCTION

LA English

DT Article

DE Robotic surgery; Reunion Island; Learning curve; Gynecology;

Hysterectomy

ID LEARNING-CURVE

AB Introduction: In February 2020, robotic surgery was introduced in University Hospital of St Pierre in Reunion Island. The aim of this study was to evaluate the implementation of robotic assisted surgery in the hospital and its impact on operating times and patient outcomes.Methods: Data was prospectively collected on patients undergoing laparoscopic robotic assisted surgery between February 2020 and February 2022. Information included patient demographics, type of surgery, operating times and length of stay.Results: Over the two-year study period, 137 patients underwent laparoscopic robotic assisted surgery per-formed by 6 different surgeons. 89 of the surgeries were in gynecology, including 58 hysterectomies, 37 were in digestive surgery, and 11 in urology. The installation and docking times decreased across all special-ties and were found to be significantly reduced when comparing the first and last 15 hysterectomies: mean installation time decreased from 18.7 to 14.5 minutes (p=0.048), mean docking time decreased from 11.3 to 7.1 minutes (p = 0.009).Conclusions: The implementation of robotic assisted surgery in an isolated territory such as Reunion Island was slow due to a lack of trained surgeons, supply difficulties and Covid crisis. Despite these challenges, the use of robotic surgery allowed for technically more challenging surgeries and demonstrated similar learning curves to other centers.(c) 2023 Elsevier Masson SAS. All rights reserved.

C1 [Reitz, Laurianne; Castel, Mathilde; Boukerrou, Malik; Tran, Phuong Lien] Ctr Hosp Univ CHU La Reunion, Serv Gynecol & Obstet, Ave Francois Mitterrand BP 350 97448 St Pierre, F-78280 La Reunion, France.

[Boukerrou, Malik; Tran, Phuong Lien] Fac Med, Unite Format & Rech Sante Reunion, F-97490 St Denis, France.

[Boukerrou, Malik; Tran, Phuong Lien] Ctr Hosp Univ Sud Reunion, Ctr Etud Perinatales Ocean Indien, BP 350, F-97448 St Pierre, France.

C3 CHU Reunion; CHU Reunion

RP Reitz, L (corresponding author), Ctr Hosp Univ CHU La Reunion, Serv Gynecol & Obstet, Ave Francois Mitterrand BP 350 97448 St Pierre, F-78280 La Reunion, France.

EM laurianne.rtz@gmail.com

OI Reitz, Laurianne/0000-0001-8417-7003

NR 13

TC 0

Z9 0

U1 0

U2 0

PU ELSEVIER MASSON, CORP OFF

PI PARIS

PA 65 CAMILLE DESMOULINS CS50083 ISSY-LES-MOULINEAUX, 92442 PARIS, FRANCE

SN 2468-7847

EI 1773-0430

J9 J GYNECOL OBSTET HUM

JI J. Gynecol. Obstet. Hum. Reprod.

PD MAY

PY 2023

VL 52

IS 5

AR 102586

DI 10.1016/j.jogoh.2023.102586

EA APR 2023

PG 5

WC Obstetrics & Gynecology

WE Science Citation Index Expanded (SCI-EXPANDED)

SC Obstetrics & Gynecology

GA F8IJ5

UT WOS:000984727600001

PM 37030505

DA 2024-01-18

ER

PT J

AU Guan, XM

Guan, ZK

Sunkara, S

Thigpen, B

AF Guan, Xiaoming

Guan, Zhenkun

Sunkara, Sowmya

Thigpen, Brooke

TI Indocyanine Green-Assisted Retrograde Ureterolysis in Robotic

Transvaginal NOTES for the Management of Stage IV Endometriosis with

Obliterated Cul-de-sac

SO JOURNAL OF MINIMALLY INVASIVE GYNECOLOGY

LA English

DT Article

DE Indocyanine green; Robotic; Stage IV endometriosis; Reverted

ureterolysis; Obliterated cul-de-sac

AB Study Objective: To explore the use of indocyanine green (ICG) in highlighting ureteral anatomical landmarks for the suc-cessful and safe execution of robotic-assisted transvaginal NOTES hysterectomy with resection of deeply infiltrated endometriosis. Design: Stepwise demonstration with narrated video footage. Setting: An academic tertiary care hospital. Our patient is a 38-year-old G4P1031 with a symptomatic enlarged uterus sec-ondary to adenomyosis and uterine myomas, dense adhesions between the posterior uterus, and left uterosacral ligament. Interventions: Stage IV endometriosis with obliterated cul-de-sac is a challenging procedure in the surgical management of endometriosis. Ureterolysis is the key step to performing this surgery successfully and safely; however, the routine dissec-tion of ureters from the sacral promontory level to the uterine artery is challenging in obliterated cul-de-sacs with pelvic side wall adhesions with the proximal ureter at greatest risk [1-4]. Using the ICG firefly technique allowed us to rapidly identify and safely dissect the ureter through robotic transabdominal endometriosis surgery [5,6]. The angle of approach in transvaginal NOTES surgery for hysterectomy with obliterated cul-de-sac endometriosis leads to far more difficulty in iden-tifying the ureter at the beginning of surgery [3]. Therefore, an obliterated cul-de-sac was associated with a potentially increased risk of ureteral injury and bowel injury. We used ICG to help identify the ureter at the beginning of the case lead-ing to reducing the risk of surgical complication, in which the concept of ureterolysis from the level of the uterine artery to the bifurcation of common iliac vessels in vNOTES surgery will be referred to as "vNOTES retrograde ureterolysis." With the cystoscope in place, a ureteral catheter was inserted into the right ureter and 5 cc of ICG was injected, and the same procedure was done on the left [1,5]. Bovie electrosurgical device was used to incise circumferentially around the cervix. The bladder was dissected off the pubovesical cervical fascia anteriorly and posteriorly with a combination of the Bovie as well as blunt and sharp dissection. Bilateral uterosacral and cardinal ligaments, as well as uterine arteries, were then clamped, transected with Mayo scissors, and secured. Entry into the anterior cul-de-sac was completed, and a stitch using 0 vicryl was used to tag the anterior peritoneum to the anterior vaginal cuff. Posterior entry was attempted unsuccessfully. The Gelpoint mini device was then placed, and the Da Vinci XI robot was docked. Bilateral ureters were identified and dis-sected out of bilateral pelvic sidewalls using the firefly mode at the level of the uterine artery. The ureters were easily dis-sected away from the uterus. The left broad ligament was then cauterized and transected using the vessel sealer. The plane between the uterus and the rectum was identified laterally, and the rectum was taken down from the uterus from the right to the left side. Bilateral broad ligaments were then cauterized and transected using the vessel sealer, followed by cauterization and transection of the round ligaments, utero-ovarian ligaments, and mesosalpinx bilaterally. The vaginal cuff angles were secured with a figure-of-eight stitch of 0 vicryl, and the vaginal cuff was then closed in a running fashion with 0 V-Loc. The patient was discharged in one day with reports of minimal pain (Videos 1-3).

Conclusion: Robotic-assisted NOTES hysterectomy with deeply infiltrated endometriosis resection is feasible and safe with ICG-assisted ureteral labeling in a case of obliterated cul-de-sac. The unique green color labeling of ureters offers a prominent landmark in assisting the ureteral dissection while avoiding ureteral and bowel injury, resulting in the possibility of using vNOTES surgery in challenging cases. Journal of Minimally Invasive Gynecology (2023) 30, 266-267. (c) 2023 AAGL. All rights reserved.

C1 [Guan, Xiaoming; Sunkara, Sowmya; Thigpen, Brooke] Baylor Coll Med, Div Minimally Invas Gynecol Surg, Houston, TX USA.

[Guan, Zhenkun] Guangzhou Med Univ, Guangzhou, Guangdong, Peoples R China.

[Guan, Xiaoming] Baylor Coll Med, Dept Minimally Invas Gynecol Surg, 6651 Main St,10th Floor, Houston, TX 77030 USA.

C3 Baylor College of Medicine; Guangzhou Medical University; Baylor College

of Medicine

RP Guan, XM (corresponding author), Baylor Coll Med, Dept Minimally Invas Gynecol Surg, 6651 Main St,10th Floor, Houston, TX 77030 USA.

EM xiaoming@bcm.edu

NR 6

TC 2

Z9 3

U1 1

U2 3

PU ELSEVIER SCIENCE INC

PI NEW YORK

PA STE 800, 230 PARK AVE, NEW YORK, NY 10169 USA

SN 1553-4650

EI 1553-4669

J9 J MINIM INVAS GYN

JI J. Minim. Invasive Gynecol.

PD APR

PY 2023

VL 30

IS 4

BP 266

EP 267

DI 10.1016/j.jmig.2023.02.005

EA APR 2023

PG 2

WC Obstetrics & Gynecology

WE Science Citation Index Expanded (SCI-EXPANDED)

SC Obstetrics & Gynecology

GA E7CI1

UT WOS:000977076200001

PM 36764648

DA 2024-01-18

ER

PT J

AU Tannus, S

Giannini, A

Magrina, JF

Crosson, J

Kosiorek, H

Yi, JHY

Butler, KA

AF Tannus, Samer

Giannini, Andrea

Magrina, Javier F.

Crosson, Jacque

Kosiorek, Heidi

Yi, Johnny

Butler, Kristina A.

TI Same-day Discharge after Robotic Hysterectomy for Benign Conditions:

Feasibility and Safety

SO JOURNAL OF MINIMALLY INVASIVE GYNECOLOGY

LA English

DT Article

DE Hysterectomy; Outcome; Predictors; Robotic; Same-day discharge

ID LAPAROSCOPIC HYSTERECTOMY

AB Study Objective: To investigate the feasibility and predictive factors for same-day discharge (SDD) after robotic hysterec-tomy (RH) for benign indications to optimize patient selection by incorporating preoperative, intraoperative, and postopera-tive variables. Design: A single-center retrospective cohort study. Setting: Tertiary academic hospital. Patients: Patients undergoing RH for benign indications. Interventions: Patients were designated for SDD by implementing enhanced recovery after surgery protocol. Measurements and Main Results: The study included 890 patients who underwent RH for benign indications between the years 2016 and 2021. Of these, 618 (69.4%) were discharged the same day and 272 (30.5%) were admitted for overnight stay. Both groups had similar age (46.4 vs 46.2 years), body mass index (28.3 vs 28.9), and indications for surgery. In multi -variable logistic regression, factors that were significant for overnight stay were American Society of Anesthesiologists score 3, Charlson comorbidity index, previous laparotomy, and operative time. Other factors such as surgery start time and preoperative hemoglobin levels were not statistically significant. Postoperative outcomes were comparable for both groups with similar readmission and reoperation rates. Conclusion: The likelihood of SDD after RH in this cohort after implementing enhanced recovery after surgery protocol was almost 70%, and most of the predictive factors for overnight stay were nonmodifiable. Importantly, both groups had similar outcomes after surgery. Journal of Minimally Invasive Gynecology (2023) 30, 277-283. (c) 2022 AAGL. All rights reserved.

C1 [Tannus, Samer; Giannini, Andrea; Magrina, Javier F.; Crosson, Jacque; Yi, Johnny; Butler, Kristina A.] Mayo Clin, Dept Med & Surg Gynecol, Phoenix, AZ 85054 USA.

[Kosiorek, Heidi] Mayo Clin, Dept Quantitat Hlth Sci, Scottsdale, AZ USA.

[Tannus, Samer] Mayo Clin, Dept Med & Surg Gynecol, 5777 East Mayo Blvd, Phoenix, AZ 85054 USA.

C3 Mayo Clinic; Mayo Clinic Phoenix; Mayo Clinic; Mayo Clinic Phoenix; Mayo

Clinic; Mayo Clinic Phoenix

RP Tannus, S (corresponding author), Mayo Clin, Dept Med & Surg Gynecol, 5777 East Mayo Blvd, Phoenix, AZ 85054 USA.

EM tannus.samer@mayo.edu

OI Giannini, Andrea/0000-0003-0512-4435

NR 19

TC 2

Z9 2

U1 0

U2 0

PU ELSEVIER SCIENCE INC

PI NEW YORK

PA STE 800, 230 PARK AVE, NEW YORK, NY 10169 USA

SN 1553-4650

EI 1553-4669

J9 J MINIM INVAS GYN

JI J. Minim. Invasive Gynecol.

PD APR

PY 2023

VL 30

IS 4

BP 277

EP 283

DI 10.1016/j.jmig.2022.12.007

EA APR 2023

PG 7

WC Obstetrics & Gynecology

WE Science Citation Index Expanded (SCI-EXPANDED)

SC Obstetrics & Gynecology

GA F5BW4

UT WOS:000982509100001

PM 36528258

DA 2024-01-18

ER

PT J

AU Mothes, AR

Kather, A

Cepraga, I

Esber, A

Kwetkat, A

Runnebaum, IB

AF Mothes, Anke R.

Kather, Angela

Cepraga, Irina

Esber, Anke

Kwetkat, Anja

Runnebaum, Ingo B.

TI Robotic-assisted Gynecological Surgery in Older Patients - a Comparative

Cohort Study of Perioperative Outcomes

SO GEBURTSHILFE UND FRAUENHEILKUNDE

LA German

DT Article

DE benign and oncological indications; comorbidity; Clavien-Dindo

classification; old age; robotic-assisted gynecological surgery

ID ENDOMETRIAL CANCER; UTERINE-CANCER; CO-MORBIDITY; HYSTERECTOMY; AGE;

CLASSIFICATION; COMORBIDITY; PREVALENCE; LAPAROTOMY; PROLAPSE

AB Study design Because of current demographic developments, a hypothesis was proposed whereby older female patients aged > 65 years can be safely operated using minimally invasive, robotic-assisted surgery, despite having more preoperative comorbidities. A comparative cohort study was designed to compare the age group = 65 years (older age group, OAG) with the age group < 65 years (younger age group, YAG) after robotic-assisted gynecological surgery (RAS) in two German centers. Patients and methods Consecutive RAS procedures performed between 2016 and 2021 at the Women's University Hospital of Jena and the Robotic Center Eisenach to treat benign or oncological indications were included in the study. The age groups were compared according to their preoperative comorbidities (ASA, Charlson comorbidity index [CCI], cumulative illness rating scale - geriatric version [CIRS-G]) and perioperative parameters such as Clavien-Dindo (CD) classification of surgical complications. Analysis was performed using Welch's t-test, chi2 test, and Fisher's exact test. Results A total of 242 datasets were identified, of which 63 (73 +/- 5 years) were OAG and 179 were YAG (48 +/- 10 years). Patient characteristics and the percentage of benign or oncological indications did not differ between the two age groups. Comorbidity scores and the percentage of obese patients were higher in the OAG group: CCI (2.7 +/- 2.0 vs. 1.5 +/- 1.3; p < 0.001), CIRS-G (9.7 +/- 3.9 vs. 5.4 +/- 2.9; p < 0.001), ASA class II/ III (91.8% vs. 74.1 %; p = 0.004), obesity (54.1% vs. 38.2 %; p = 0.030). There was no difference between age groups, even grouped for benign or oncological indications, with regard to perioperative parameters such as duration of surgery (p = 0.088; p = 0.368), length of hospital stay (p = 0.786; p = 0.814), decrease in Hb levels (p = 0.811; p = 0.058), conversion rate (p = 1.000; p = 1.000) and CD complications (p = 0.433; p = 0.745). Conclusion Although preoperative comorbidity was higher in the group of older female patients, no differences were found between age groups with regard to perioperative outcomes following robotic-assisted gynecological surgery. Patient age is not a contraindication for robotic gynecological surgery.

C1 [Mothes, Anke R.; Cepraga, Irina; Esber, Anke] Univ Klinikum Jena, St Georg Klinikum Eisenach, Klin Frauenheilkunde & Robot Zentrum, Akad Lehrkrankenhaus, Eisenach, Germany.

[Kather, Angela; Cepraga, Irina; Esber, Anke; Runnebaum, Ingo B.] Univ Klinikum Jena, Klin & Poliklin Frauenheilkunde & Fortpflanzung M, Jena, Germany.

[Kwetkat, Anja] Klinikum Osnabruck GmbH, Klin Geriatr & Palliat Med, Osnabruck, Germany.

C3 Friedrich Schiller University of Jena

RP Runnebaum, IB (corresponding author), Univ Klinikum Jena, Klin & Poliklin Frauenheilkunde & Fortpflanzung M, Klinikum 1, D-07747 Jena, Germany.

EM ingo.runnebaum@med.uni-jena.de

NR 45

TC 0

Z9 0

U1 0

U2 0

PU GEORG THIEME VERLAG KG

PI STUTTGART

PA RUDIGERSTR 14, D-70469 STUTTGART, GERMANY

SN 0016-5751

EI 1438-8804

J9 GEBURTSH FRAUENHEILK

JI Geburtshilfe Frauenheilkd.

PD APR

PY 2023

VL 83

IS 4

BP 437

EP 445

PG 9

WC Obstetrics & Gynecology

WE Science Citation Index Expanded (SCI-EXPANDED)

SC Obstetrics & Gynecology

GA M4BK0

UT WOS:001029660900011

PM 37153652

OA Green Published, hybrid

DA 2024-01-18

ER

PT J

AU Lechartier, C

Bernard, J

Renaud, MC

Plante, M

AF Lechartier, Celine

Bernard, Juliette

Renaud, Marie-Claude

Plante, Marie

TI Robotic-assisted surgery for endometrial cancer is safe in morbidly and

extremely morbidly obese patients

SO GYNECOLOGIC ONCOLOGY

LA English

DT Article

DE Endometrial cancer; Morbid obesity; Robotic-assisted surgery

ID BODY-MASS INDEX; LAPAROSCOPIC HYSTERECTOMY; SURGICAL-TREATMENT;

LAPAROTOMY; OUTCOMES; IMPACT; COMPLICATIONS; HYPERPLASIA; EXPERIENCE;

CARCINOMA

AB Objective. Obesity has risen to affect >25% of the Canadian population. Perioperative challenges with in-creased morbidity are encountered. We evaluated the outcome of robotic-assisted surgery for endometrial can-cer (EC) in obese patients.Methods. We retrospectively reviewed all robotic surgeries performed for EC in women with BMI >= 40 kg/m2, from 2012 to 2020 in our center. Patients were divided into 2 groups (class III: 40-49 kg/m2, class IV: >= 50 kg/m2). Complications and outcome were compared.Results. 185 patients were included: 139 class III and 46 class IV. The main histology was endometrioid ade-nocarcinoma (70,5% of class III and 58,1% of class IV (p = 0,138)). The mean blood loss, overall sentinel node de-tection and median length of stay were similar in both groups. Six class III (4,3%) and 3 class IV (6,5%) patients required conversion to laparotomy due to poor surgical field exposure (p = 0,692). The rate of intraoperative complications was similar between the 2 groups (1.4% in class III vs none in class IV, p = 1). There were 10 class III (7,2%) and 10 class IV (21,7%) post-operative complications (p = 0.011), but most were grade 2 (3,6% in class III vs 13% in class IV, p = 0.029)). Grade 3 and 4 postoperative complications were low (2.7%) and not statistically different between the 2 groups. Readmission rate was low in both groups (4 in each group, p = 1.07). Recurrence occurred in 5,8% of class III and 4,3% of class IV patients (p = 1).Conclusion. Robotic-assisted surgery for EC in class III and class IV obese patients is a safe and feasible proce-dure, with low complication rate, similar oncologic outcome, conversion rate, blood loss, readmission rate and length of hospital stay.(c) 2023 Elsevier Inc. All rights reserved.

C1 [Lechartier, Celine; Bernard, Juliette; Renaud, Marie-Claude; Plante, Marie] Laval Univ, Ctr Hosp Univ Quebec, Gynecol Oncol Div, Lhotel Dieu Quebec, Quebec City, PQ, Canada.

C3 Laval University

RP Lechartier, C; Plante, M (corresponding author), Laval Univ, Ctr Hosp Univ Quebec, Gynecol Oncol Div, Lhotel Dieu Quebec, Quebec City, PQ, Canada.

EM celine.lechartier.1@ulaval.ca; marie.plante@crhdq.ulaval.ca

NR 33

TC 1

Z9 1

U1 0

U2 1

PU ACADEMIC PRESS INC ELSEVIER SCIENCE

PI SAN DIEGO

PA 525 B ST, STE 1900, SAN DIEGO, CA 92101-4495 USA

SN 0090-8258

EI 1095-6859

J9 GYNECOL ONCOL

JI Gynecol. Oncol.

PD MAY

PY 2023

VL 172

BP 15

EP 20

DI 10.1016/j.ygyno.2023.02.014

EA MAR 2023

PG 6

WC Oncology; Obstetrics & Gynecology

WE Science Citation Index Expanded (SCI-EXPANDED)

SC Oncology; Obstetrics & Gynecology

GA A6XR3

UT WOS:000956536200001

PM 36905768

DA 2024-01-18

ER

PT J

AU Fallon, M

Nolan, W

Jeyalingam, P

AF Fallon, Matthew

Nolan, William

Jeyalingam, Praba

TI Report Robotic-assisted Laparoscopic Supracervical Repair of a Chronic

Puerperal Uterine Inversion

SO JOURNAL OF MINIMALLY INVASIVE GYNECOLOGY

LA English

DT Article

DE DaVinci; Hysterotomy; Pregnancy

AB Surgical correction of a chronic puerperal uterine inversion traditionally requires an anterior or posterior cervical incision to relieve the constricting band. This case is only the second reported case of robotic-assisted correction of a chronic puerperal uterine inversion and the first to avoid a cervical incision. The patient was 5 months postpartum and desired future preg-nancy. After a laparoscopic Huntington technique was unsuccessful, a vertical hysterotomy was created in the anterior lower uterine segment and extended toward the fundus until the inversion could be relieved. The incision was repaired in 3 layers and a round ligament plication was performed to provide additional support within the pelvis. The patient's symptoms grad-ually improved during her postoperative course, and ultrasound 2 weeks after the procedure revealed the uterus in anatomic position in the pelvis. With a paucity of reported cases of laparoscopic correction of chronic puerperal uterine inversion, the present case offers a novel surgical approach that maintains cervical integrity and thereby minimizes long-term effects of the procedure on future pregnancies. Journal of Minimally Invasive Gynecology (2023) 30, 245-248. (c) 2022 AAGL. All rights reserved.

C1 [Fallon, Matthew; Nolan, William; Jeyalingam, Praba] Univ Buffalo, Sisters Char Hosp, Dept Obstet & Gynecol, Buffalo, NY USA.

[Fallon, Matthew] Sisters Charity Hosp, Dept Obstet & Gynecol, 2157 Main St, Buffalo, NY 14214 USA.

C3 State University of New York (SUNY) System; State University of New York

(SUNY) Buffalo

RP Fallon, M (corresponding author), Sisters Charity Hosp, Dept Obstet & Gynecol, 2157 Main St, Buffalo, NY 14214 USA.

EM matthewdavidfallon@gmail.com

NR 10

TC 0

Z9 0

U1 0

U2 0

PU ELSEVIER SCIENCE INC

PI NEW YORK

PA STE 800, 230 PARK AVE, NEW YORK, NY 10169 USA

SN 1553-4650

EI 1553-4669

J9 J MINIM INVAS GYN

JI J. Minim. Invasive Gynecol.

PD MAR

PY 2023

VL 30

IS 3

BP 245

EP 248

DI 10.1016/j.jmig.2022.11.016

EA MAR 2023

PG 4

WC Obstetrics & Gynecology

WE Science Citation Index Expanded (SCI-EXPANDED)

SC Obstetrics & Gynecology

GA A4IT5

UT WOS:000954787600001

PM 36481556

DA 2024-01-18

ER

PT J

AU Hiratsuka, D

Tsuchiya, A

Isono, W

Honda, M

Tsuchiya, H

Matsuyama, R

Fujimoto, A

Nishii, O

AF Hiratsuka, Daiki

Tsuchiya, Akira

Isono, Wataru

Honda, Michiko

Tsuchiya, Hiroko

Matsuyama, Reiko

Fujimoto, Akihisa

Nishii, Osamu

TI Robotic-Assisted Laparoscopic Hysterectomy versus Conventional

Laparoscopic Hysterectomy for Endometrial Cancer at a Regional

Institution: A Retrospective Study

SO CLINICAL AND EXPERIMENTAL OBSTETRICS & GYNECOLOGY

LA English

DT Article

DE laparoscopic hysterectomy; local facility; minimally invasive surgery;

pelvic lymphadenectomy; robotic surgery; rural hospital; uterine corpus

cancer

ID SURGERY; SURVIVAL; RECURRENCE

AB Background: Minimally invasive surgeries, such as laparoscopic and robotic surgeries, have been the main treatment methods for stage I endometrial cancer instead of laparotomy. However, minimally invasive surgeries for malignant tumors have not yet been established in many rural hospitals or hospitals with few gynecologists. This study aimed to investigate whether laparoscopic or robotic surgery for stage I endometrial cancer is more sustainable and useful at a rural hospital where a single non-laparoscopic-specialized surgeon performs oncologic surgery and provides outpatient care. Methods: This retrospective case-control study was conducted at our hospital. The study enrolled 65 patients with endometrial cancer who underwent robotic-assisted laparoscopic hysterectomy (RALH) or total laparoscopic hysterectomy (TLH). We compared surgical outcomes such as patient background, operation time, blood loss, and other indices. Results: Exactly 34 patients underwent robotic surgery, and 31 underwent laparoscopic surgery. No severe adverse events required reoperation, conversion to laparotomy, or ureteral injury during either operation. The operation time decreased in patients who underwent robotic surgery compared with those who underwent laparoscopic surgery (193 (140-227) vs. 253 (219-287) min, p < 0.001). In addition, the blood loss volume decreased by half in patients who underwent robotic surgery compared to those who underwent laparoscopic surgery. Significantly more operations were completed by two operators rather than three operators at robotic surgery compared to laparoscopic surgery (59% vs. 26%, p = 0.007). The hospitalization days were 1.5 days shorter in the robotic surgery group than in the laparoscopic surgery group (p < 0.001). Exactly 18 patients underwent robotic surgery with pelvic lymphadenectomy, and 26 underwent laparoscopic surgery with pelvic lymphadenectomy. Patients who underwent robotic surgery required less operation time than those who underwent laparoscopic surgery (226 (199-246) vs. 261 (236-287) min, p = 0.001). Conclusions: In the surgical treatment of stage I endometrial cancer, robotic surgery was associated with a significantly shorter operation time, shorter hospital stay, and no obvious complications. This study proposes that robotic surgery is a promising solution for the sustainable introduction of minimally invasive surgery for stage I endometrial cancer in rural hospitals or hospitals with few gynecologists.

C1 [Hiratsuka, Daiki; Tsuchiya, Akira; Isono, Wataru; Honda, Michiko; Tsuchiya, Hiroko; Matsuyama, Reiko; Fujimoto, Akihisa; Nishii, Osamu] Teikyo Univ, Univ Hosp Mizonokuchi, Dept Obstet & Gynaecol, Sch Med, Kawasaki, Kanagawa 2138507, Japan.

C3 Teikyo University

RP Tsuchiya, A (corresponding author), Teikyo Univ, Univ Hosp Mizonokuchi, Dept Obstet & Gynaecol, Sch Med, Kawasaki, Kanagawa 2138507, Japan.

EM tsuchiya@med.teikyo-u.ac.jp

OI Hiratsuka, Daiki/0000-0001-7128-2859

NR 24

TC 0

Z9 0

U1 0

U2 0

PU IMR PRESS

PI ROBINSON

PA 112 ROBINSON RD, ROBINSON, SINGAPORE

SN 0390-6663

EI 2709-0094

J9 CLIN EXP OBSTET GYN

JI Clin. Exp. Obstet. Gynecol.

PD MAR

PY 2023

VL 50

IS 3

AR 61

DI 10.31083/j.ceog5003061

PG 7

WC Obstetrics & Gynecology

WE Science Citation Index Expanded (SCI-EXPANDED)

SC Obstetrics & Gynecology

GA E4SJ5

UT WOS:000975453700004

OA gold

DA 2024-01-18

ER

PT J

AU Panico, G

Campagna, G

Caramazza, D

Vacca, L

Mastrovito, S

Ercoli, A

Scambia, G

AF Panico, G.

Campagna, G.

Caramazza, D.

Vacca, L.

Mastrovito, S.

Ercoli, A.

Scambia, G.

TI HUGOTM RAS System in urogynaecology: the first nerve sparing Sacral

Colpopexy for Pelvic Organ Prolapse

SO FACTS VIEWS AND VISION IN OBGYN

LA English

DT Article

DE Laparoscopy; pelvic organ prolapse; colposacropexy; anatomy; dissection

ID PERCUTANEOUS SURGICAL SYSTEM; SALPINGO-OOPHORECTOMY; HYSTERECTOMY;

METAANALYSIS

AB Background: Minimally invasive sacral colpopexy is considered the gold standard for surgical treatment of Pelvic Organ Prolapse (POP), combining high success rates with low recurrence risk in comparison to other techniques. This is the first case of robotic sacral colpopexy (RSCP) performed with the innovative HugoTM RAS robotic system. Objectives: The aim of this article is to show the surgical steps of a nerve sparing RSCP performed with the new HugoTM RAS robotic system (Medtronic), by also evaluating the feasibility of this technique using this novel Robotic System.Materials and methods: A 50-year-old Caucasian woman with symptomatic pelvic organ prolapse (POP-Q): Aa: +2, Ba: +3, C: +4, D: +4, Bp:-2, Ap:-2 , TVL:10 GH: 3,5 BP:3 underwent RSCP as well as a subtotal hysterectomy with bilateral salpingo-oophorectomy, using the new surgical robot HugoTM RAS in the Division of Urogynaecology and Pelvic Reconstructive Surgery, Fondazione Policlinico Universitario A. Gemelli IRCCS, Rome, Italy. Main outcome measures: Intraoperative data, docking specifics, objective and subjective outcomes at three months follow up.Results: Surgical procedure was carried out without intra-operative complications, operative time (OT) was 150 minutes, docking time was 9 minutes. No system errors or faults in the robotic arms were registered. Urogynaecological examination at three months follow up showed a complete resolution of the prolapse.Conclusion: RSCP using the HugoTM RAS system seems to be a feasible and effective approach according to results in terms of operative time, cosmetic results, postoperative pain and length of hospitalisation. Large number of case reports as well as longer follow up are mandatory to better define its benefits, advantages, and costs.

C1 [Panico, G.; Campagna, G.; Caramazza, D.; Vacca, L.; Mastrovito, S.; Scambia, G.] Univ Cattolica Sacro Cuore, Fdn Policlin Univ A Gemelli IRCCS, Dipartimento Sci Salute Donna & Bambino & Sanita P, UOC Chirurg Ginecol, I-00168 Rome, Italy.

[Ercoli, A.] Univ Messina, PID Ginecol Oncolog & Chirurg Ginecolog Miniinvasi, Policlin GMartino, Messina, Italy.

C3 Catholic University of the Sacred Heart; IRCCS Policlinico Gemelli;

University of Messina

RP Campagna, G (corresponding author), Univ Cattolica Sacro Cuore, Fdn Policlin Univ A Gemelli IRCCS, Dipartimento Sci Salute Donna & Bambino & Sanita P, UOC Chirurg Ginecol, I-00168 Rome, Italy.

EM giuseppecampagna80@yahoo.com

RI Mastrovito, Sara/HTT-4728-2023; Panico, Giovanni/GLV-0862-2022

OI Panico, Giovanni/0000-0001-5473-9503

NR 17

TC 8

Z9 8

U1 0

U2 1

PU UNIVERSA PRESS

PI WETTEREN

PA RUE HOENDER 24, WETTEREN, 9230, BELGIUM

SN 2032-0418

J9 FACTS VIEWS VIS OBGY

JI Facts Views Vis. ObGyn

PD MAR

PY 2023

VL 15

IS 1

BP 83

EP 87

DI 10.52054/FVVO.15.1.054

PG 5

WC Obstetrics & Gynecology

WE Emerging Sources Citation Index (ESCI)

SC Obstetrics & Gynecology

GA E4YM4

UT WOS:000975613100011

PM 37010339

OA Bronze, Green Published

DA 2024-01-18

ER

PT J

AU Swartz, AZ

Novoa, VNYA

Hassoun, JS

Crispens, MA

Prescott, LS

AF Swartz, Alison Z.

Novoa, Victoria Novoa Y. Arruga

Hassoun, Jenine S.

Crispens, Marta A.

Prescott, Lauren S.

TI Robotic-assisted gynecologic surgery associated tympanic membrane

perforation: A report of two cases and review of the literature

SO GYNECOLOGIC ONCOLOGY REPORTS

LA English

DT Review

DE Tympanic membrane perforation; Otorrhagia; Gynecologic surgery;

Trendelenburg; Robotic surgery; Endometrial cancer

ID BILATERAL OTORRHAGIA

AB Robotic gynecologic surgery is associated with the use of steep Trendelenburg positioning. Steep Trendelenburg is necessary to provide optimal exposure to the pelvis but is associated with an increased risk of non-surgical complications such as suboptimal ventilation, facial and laryngeal edema, increased intraocular and intracranial pressure as well as neurologic injury. Several case reports have described otorrhagia after robotic assisted surgery; however, there are limited reports on the risk of tympanic membrane perforation. To our knowledge, there are no published reports on tympanic membrane perforation in gynecologic nor gynecologic oncology surgery. We report two cases of perioperative tympanic membrane rupture and bloody otorrhagia associated with robot-assisted gynecologic surgery. In both cases otolaryngology/Ear Nose and Throat (ENT) was consulted, and the perforations resolved with conservative management.

C1 [Swartz, Alison Z.] Vanderbilt Univ, Sch Med, Nashville, TN 37235 USA.

[Novoa, Victoria Novoa Y. Arruga; Hassoun, Jenine S.; Crispens, Marta A.; Prescott, Lauren S.] Vanderbilt Univ, Med Ctr, Dept Gynecol Oncol, Nashville, TN USA.

C3 Vanderbilt University; Vanderbilt University

RP Swartz, AZ (corresponding author), Vanderbilt Univ, Sch Med, Nashville, TN 37235 USA.

EM alison.z.williams@vanderbilt.edu

OI Williams, Alison/0000-0003-4203-6389

NR 15

TC 0

Z9 0

U1 0

U2 0

PU ELSEVIER SCIENCE INC

PI NEW YORK

PA STE 800, 230 PARK AVE, NEW YORK, NY 10169 USA

SN 2352-5789

J9 GYNECOL ONCOL REP

JI Gynecol. Oncol. Rep.

PD APR

PY 2023

VL 46

AR 101151

DI 10.1016/j.gore.2023.101151

EA FEB 2023

PG 3

WC Obstetrics & Gynecology

WE Emerging Sources Citation Index (ESCI)

SC Obstetrics & Gynecology

GA D2ND8

UT WOS:000967132700001

PM 36873858

OA Green Published, gold

DA 2024-01-18

ER

PT J

AU Lange, S

Chatziioannidou, K

Daellenbach, P

AF Lange, Soeren

Chatziioannidou, Kyriaki

Daellenbach, Patrick

TI Robotically assisted laparoscopic lateral suspension: a step-by-step

approach aiming to standardize a novel procedure

SO INTERNATIONAL UROGYNECOLOGY JOURNAL

LA English

DT Article

DE Pelvic organ prolapse; Robotic surgery; Laparoscopic lateral suspension;

Uterine prolapse

ID PROLAPSE

AB Introduction and hypothesis The aim of this video is to show a step-by-step approach to robotically assisted laparoscopic lateral suspension for pelvic organ prolapse aiming to standardize this procedure.

Methods This video shows a robotically assisted laparoscopic approach to a POP-Q stage 3 prolapse with a combined anterior and apical defect. First, the trocars are positioned, with one 8-mm trocar, two lateral trocars 5 cm above the anterior-superior iliac spine, and a 10-mm assistant trocar either paraumbilically or suprapubically. Second, the uterovesical pouch is dissected up to 2 cm above the level of the bladder neck. The mesh is then fixed to the vesicovaginal fascia and to the isthmus uteri. Next, a laparoscopic forceps is inserted retroperitoneally through the lateral trocars and the lateral arms of the mesh are pulled retroperitoneally. The peritoneum of the uterovesical fold is sutured, including round ligament plication. Finally, the lateral arms of the peritoneum are fixed to the peritoneum of the abdominal wall.

Conclusions Robotically assisted laparoscopic lateral suspension is a safe alternative to laparoscopic and robotically assisted laparoscopic sacropexy and very well suited for uterine-preserving POP surgery. This video contributes to the standardization of this procedure, and we believe our video to be useful in helping urogynecologists to perform this innovative procedure.

C1 [Lange, Soeren] Univ Hosp Vienna, Dept Gynecol & Obstet, Div Gynecol, Urogynecol Unit, Vienna, Austria.

[Lange, Soeren] Med Univ Vienna, Waehringer Guertel 18, A-1090 Vienna, Austria.

[Chatziioannidou, Kyriaki] Nyon Hosp, GHOL Grp, Dept Gynecol & Obstet, Nyon, Switzerland.

[Daellenbach, Patrick] Geneva Univ Hosp, Dept Pediat Gynecol & Obstet, Div Gynecol, Urogynecol Unit, Geneva, Switzerland.

C3 University Hospital Vienna; Medical University of Vienna; University of

Geneva

RP Lange, S (corresponding author), Univ Hosp Vienna, Dept Gynecol & Obstet, Div Gynecol, Urogynecol Unit, Vienna, Austria.; Lange, S (corresponding author), Med Univ Vienna, Waehringer Guertel 18, A-1090 Vienna, Austria.

EM Soeren.lange@meduniwien.ac.at

RI Dällenbach, Patrick/W-7241-2019

OI Dällenbach, Patrick/0000-0002-1469-3552; Lange,

Soren/0000-0002-9066-7380

FU Medical University of Vienna

FX Open access funding provided by Medical University of Vienna.

NR 11

TC 0

Z9 0

U1 0

U2 1

PU SPRINGER LONDON LTD

PI LONDON

PA 236 GRAYS INN RD, 6TH FLOOR, LONDON WC1X 8HL, ENGLAND

SN 0937-3462

EI 1433-3023

J9 INT UROGYNECOL J

JI Int. Urogynecol. J.

PD MAY

PY 2023

VL 34

IS 5

BP 1131

EP 1134

DI 10.1007/s00192-023-05477-6

EA FEB 2023

PG 4

WC Obstetrics & Gynecology; Urology & Nephrology

WE Science Citation Index Expanded (SCI-EXPANDED)

SC Obstetrics & Gynecology; Urology & Nephrology

GA AJ6P9

UT WOS:000939360000002

PM 36826519

OA hybrid

DA 2024-01-18

ER

PT J

AU Montera, R

Ficarola, F

Plotti, F

Terranova, C

Nardone, CD

Guzzo, F

Angioli, R

Luvero, D

AF Montera, Roberto

Ficarola, Fernando

Plotti, Francesco

Terranova, Corrado

Nardone, Carlo De Cicco

Guzzo, Federica

Angioli, Roberto

Luvero, Daniela

TI The use of sealing hemostat patch (HEMOPATCH<SUP>®</SUP>) in laparotomic

myomectomy: a prospective case-control study

SO ARCHIVES OF GYNECOLOGY AND OBSTETRICS

LA English

DT Article

DE HEMOPATCH((R)); Sealing hemostat patch; Hemostatic agents; Laparotomic

myomectomy; Hemostasis time

ID LAPAROSCOPIC MYOMECTOMY; SEALANT; MATRIX; WOMEN; HYSTERECTOMY;

ADHESIONS; FLOSEAL; AGENT

AB Purpose Uterine myomas are the most common gynecological disease. In these cases, a myomectomy is performed traditionally laparotomically. However, alternatives have been widely used, including laparoscopic, endoscopic, and robotic surgery. During these techniques, diffuse parenchymatous bleeding remains one of the main intraoperative and postoperative complications and sometimes requires unplanned hysterectomies. Recently, hemostatic agents and sealants have been used to prevent excessive blood loss during surgical repair.

Methods We propose a prospective case-control study on the use of a sealing hemostat patch (HEMOPATCH((R))) on uterine sutures in laparotomic myomectomy. In the period between July 2016 and April 2017, 46 patients with symptomatic uterine fibromatosis underwent surgery. They were divided into two groups of 23 patients, with different treatments in the hemostatic phase of oozing bleeding. HEMOPATCH((R)) is applied in group A, and spray electrocoagulation is applied in group B.

Results In group A, we achieve faster hemostasis (p < 0.05), than in group B. We report a significantly lower C-reactive protein value on the second and third days after surgery for group A compared to group B.

Conclusions HEMOPATCH((R)), during laparotomic myomectomy, is a valid alternative solution for obtaining rapid hemostasis and consequently intraoperative and postoperative bleeding. Furthermore, we suggest that a lower inflammatory peritoneal state is probably correlated with the barrier effect of the patch on the suture.

C1 [Montera, Roberto; Ficarola, Fernando; Plotti, Francesco; Terranova, Corrado; Nardone, Carlo De Cicco; Guzzo, Federica; Angioli, Roberto; Luvero, Daniela] Univ Campus Biomed Rome, Campus Biomed Univ Hosp Fdn, Dept Obstet & Gynecol, Via Alvaro del Portillo 200, I-00128 Rome, Italy.

C3 University Campus Bio-Medico - Rome Italy; Fondazione Policlinico

Universitario Campus Bio-Medico

RP Ficarola, F (corresponding author), Univ Campus Biomed Rome, Campus Biomed Univ Hosp Fdn, Dept Obstet & Gynecol, Via Alvaro del Portillo 200, I-00128 Rome, Italy.

EM f.ficarola@unicampus.it

OI Ficarola, Fernando/0000-0002-2474-2442

NR 32

TC 2

Z9 2

U1 0

U2 0

PU SPRINGER HEIDELBERG

PI HEIDELBERG

PA TIERGARTENSTRASSE 17, D-69121 HEIDELBERG, GERMANY

SN 0932-0067

EI 1432-0711

J9 ARCH GYNECOL OBSTET

JI Arch. Gynecol. Obstet.

PD MAY

PY 2023

VL 307

IS 5

BP 1521

EP 1528

DI 10.1007/s00404-023-06957-2

EA FEB 2023

PG 8

WC Obstetrics & Gynecology

WE Science Citation Index Expanded (SCI-EXPANDED)

SC Obstetrics & Gynecology

GA G5KF9

UT WOS:000933669300002

PM 36790464

OA Green Submitted

DA 2024-01-18

ER

PT J

AU Bolovis, DI

Schreibmayer, M

Hitzl, W

Brucker, CVM

AF Bolovis, Dimitrios Ilias

Schreibmayer, Michael

Hitzl, Wolfgang

Brucker, Cosima Veronika Maria

TI Retrospective analysis of apical prolapse correction by unilateral

pectineal suspension: perioperative and short-term results

SO INTERNATIONAL UROGYNECOLOGY JOURNAL

LA English

DT Article

DE Pelvic organ prolapse; Unilateral pectineal suspension; Robotic surgery

ID PELVIC ORGAN PROLAPSE; LAPAROSCOPIC LATERAL SUSPENSION; CONTINUOUS

SERIES; OUTCOME REPORT; SACROCOLPOPEXY; EPIDEMIOLOGY; SYMPTOMS; INDEX;

WOMEN; MESH

AB Introduction and hypothesisWe have previously published the novel method of unilateral pectineal suspension (UPS) for apical prolapse correction. UPS provides mesh-free midline uterus suspension using a single non-absorbable suture to attach the anterior cervix to the lateral part of the iliopectineal ligament. The purpose of this retrospective cohort study was to analyze the short-term efficacy, perioperative complication rate, and overall patient acceptance of the new UPS surgical concept.MethodsForty-seven patients with POP-Q stage 2-4 who underwent robotic UPS between January 1, 2020 and December 31, 2021 were included in the study. Patient data were taken retrospectively from the patient files. Treatment success was the primary endpoint, measured both objectively using a defined composite endpoint and subjectively according to patients' acceptance 3-6 months after surgery during a follow-up examination. Secondary outcome measures included complications and conversions, and effect of additional procedures on operative time.ResultsTreatment success as measured by the defined composite endpoint was 93.6% for the entire cohort. No complications or conversions occurred. Mean operation time for isolated UPS was 46.5 min (n = 33 patients). UPS can be easily combined with additional surgical procedures for repair of remaining pelvic floor defects, incontinence surgery or other indications. Additional procedures performed had a significant influence on operation time (p < 0.0005, n = 14).ConclusionsUPS shows highly favorable results when looking at an unselected cohort of patients in need of primary POP surgery with respect to established quality parameters of POP repair.

C1 [Bolovis, Dimitrios Ilias; Schreibmayer, Michael; Brucker, Cosima Veronika Maria] Paracelsus Med Univ, Univ Womens Hosp, Nurnberg, Germany.

[Bolovis, Dimitrios Ilias] Georg Simon Ohm Tech Univ, Nurnberg, Germany.

[Bolovis, Dimitrios Ilias; Schreibmayer, Michael; Hitzl, Wolfgang; Brucker, Cosima Veronika Maria] Paracelsus Med Univ, Res Program Expt Ophthalmol & Glaucoma Res, Salzburg, Austria.

[Bolovis, Dimitrios Ilias; Schreibmayer, Michael; Hitzl, Wolfgang; Brucker, Cosima Veronika Maria] Paracelsus Med Univ, Dept Res & Innovat, Salzburg, Austria.

[Bolovis, Dimitrios Ilias] Klinikum Nuremberg, Dept Obstet & Gynecol, Prof Ernst Nathan Str 1, D-90419 Nurnberg, Germany.

[Schreibmayer, Michael] Barmherzige Bruder Krankenhaus, St Veit Glan, Austria.

[Hitzl, Wolfgang] Paracelsus Med Univ, Dept Ophthalmol & Optometry, Salzburg, Austria.

C3 Paracelsus Private Medical University; Paracelsus Private Medical

University; Klinikum Nurnberg Nord; Konventhospital Der Barmherzigen

Bruder; Paracelsus Private Medical University

RP Bolovis, DI (corresponding author), Paracelsus Med Univ, Univ Womens Hosp, Nurnberg, Germany.; Bolovis, DI (corresponding author), Georg Simon Ohm Tech Univ, Nurnberg, Germany.; Bolovis, DI (corresponding author), Paracelsus Med Univ, Res Program Expt Ophthalmol & Glaucoma Res, Salzburg, Austria.; Bolovis, DI (corresponding author), Paracelsus Med Univ, Dept Res & Innovat, Salzburg, Austria.; Bolovis, DI (corresponding author), Klinikum Nuremberg, Dept Obstet & Gynecol, Prof Ernst Nathan Str 1, D-90419 Nurnberg, Germany.

EM dimitrios.bolovis@klinikum-nuernberg.de

OI Bolovis, Dimitrios/0000-0002-4935-2328

NR 28

TC 1

Z9 1

U1 0

U2 0

PU SPRINGER LONDON LTD

PI LONDON

PA 236 GRAYS INN RD, 6TH FLOOR, LONDON WC1X 8HL, ENGLAND

SN 0937-3462

EI 1433-3023

J9 INT UROGYNECOL J

JI Int. Urogynecol. J.

PD AUG

PY 2023

VL 34

IS 8

BP 1877

EP 1884

DI 10.1007/s00192-023-05479-4

EA FEB 2023

PG 8

WC Obstetrics & Gynecology; Urology & Nephrology

WE Science Citation Index Expanded (SCI-EXPANDED)

SC Obstetrics & Gynecology; Urology & Nephrology

GA O9HB8

UT WOS:000931777000001

PM 36786854

OA hybrid, Green Published

DA 2024-01-18

ER

PT J

AU Eckhardt, S

Laus, K

DeAndrade, S

Lee, JE

Nguyen, J

AF Eckhardt, Sarah

Laus, Katharina

DeAndrade, Samantha

Lee, Janet

Nguyen, John

TI The impact of diabetes mellitus on pelvic organ prolapse recurrence

after robotic sacrocolpopexy

SO INTERNATIONAL UROGYNECOLOGY JOURNAL

LA English

DT Article

DE Sacrocolpopexy; Diabetes; Prolapse recurrence

ID RISK-FACTORS

AB Introduction and hypothesisData examining the effect of diabetes mellitus (DM) on prolapse recurrence after sacrocolpopexy (SCP) is limited. The primary objective of this study was to determine if DM affects prolapse recurrence after robotic SCP.MethodsThis was a retrospective cohort study of women who underwent robotic SCP between 2012 and 2019 at Kaiser Permanente Southern California. The cohort was divided into women with and without DM at the time of SCP. The primary outcome was composite failure. Secondary outcomes included recurrent compartment-specific prolapse, reoperation rates, and surgical complications.ResultsOf 547 patients included, 100 had DM. Women with DM were older, had higher BMI, higher parity, and were more likely to be nonwhite. Women with DM had more advanced prolapse at baseline but were not more likely to undergo concomitant procedures at the time of SCP. Over a median follow-up of 2.1 years (IQR 1.3, 3.4), women with DM had significantly increased risk of anterior vaginal prolapse (AVP) recurrence (13% vs 3%, p<0.01), but not composite failure (21% vs 14%, p=0.14). On multivariate regression, women with DM were almost 4 times as likely to experience AVP recurrence over time (AVP hazard ratio (HR) 3.93, 95% CI 1.29-12.03, p=0.02).ConclusionIn our cohort, DM was a risk factor for AVP recurrence but not composite failure after robotic SCP.

C1 [Eckhardt, Sarah; Laus, Katharina; DeAndrade, Samantha; Nguyen, John] Kaiser Permanente Downey Med Ctr, Dept Obstet & Gynecol, Div Female Pelv Med & Reconstruct Surg, 9449 E Imperial Hwy,C327, Downey, CA 90242 USA.

[Laus, Katharina; DeAndrade, Samantha] Harbor UCLA Med Ctr, Dept Female Pelv Med & Reconstruct Surg, Torrance, CA USA.

[Lee, Janet] Kaiser Permanente Southern Calif, Dept Res & Evaluat, Pasadena, CA USA.

C3 Kaiser Permanente; University of California System; University of

California Los Angeles; University of California Los Angeles Medical

Center; Kaiser Permanente

RP Eckhardt, S (corresponding author), Kaiser Permanente Downey Med Ctr, Dept Obstet & Gynecol, Div Female Pelv Med & Reconstruct Surg, 9449 E Imperial Hwy,C327, Downey, CA 90242 USA.

EM sarah.e.eckhardt@gmail.com

NR 22

TC 0

Z9 0

U1 0

U2 0

PU SPRINGER LONDON LTD

PI LONDON

PA 236 GRAYS INN RD, 6TH FLOOR, LONDON WC1X 8HL, ENGLAND

SN 0937-3462

EI 1433-3023

J9 INT UROGYNECOL J

JI Int. Urogynecol. J.

PD AUG

PY 2023

VL 34

IS 8

BP 1859

EP 1866

DI 10.1007/s00192-023-05455-y

EA FEB 2023

PG 8

WC Obstetrics & Gynecology; Urology & Nephrology

WE Science Citation Index Expanded (SCI-EXPANDED)

SC Obstetrics & Gynecology; Urology & Nephrology

GA O9HB8

UT WOS:000930154200001

PM 36780019

DA 2024-01-18

ER

PT J

AU Özcan, C

Sanci, A

AF Ozcan, Cihat

Sanci, Adem

TI Evaluation of robotic-assisted sacrocolpopexy videos on YouTube

SO INTERNATIONAL UROGYNECOLOGY JOURNAL

LA English

DT Article

DE Robotic; Sacrocolpopexy; YouTube

ID HEALTH INFORMATION; PATIENT; CARE

AB ObjectiveTo evaluate the quality and reliability of videos on YouTube about robotic-assisted sacrocolpopexy.MethodsYouTube's search function was used to find videos associated with robotic-assisted sacrocolpopexy. On June 1, 2022, systematic research was performed using the term "robotic-assisted sacrocolpopexy". The first 50 videos were included in the study. The quality of the videos was evaluated using the Global Quality Scale (GQS) [a 5-point scale: high quality (4 or 5), acceptable quality (3), low quality (1 or 2)]. The modified DISCERN scale was used to evaluate the reliability of the videos [a 5-point scale: high reliability (5), moderate reliability (3 and 4), low reliability (1 or 2)].ResultsA total of 50 videos associated with robotic-assisted sacrocolpopexy were analyzed. The most frequently discussed topics in the videos were the duration of the surgery (72%), the anatomical success rates (70%), the advantages and disadvantages of the procedure compared to the classical methods (68%), and the postoperative complications (60%). The mean GQS of the videos was 3.04 +/- 0.75. The mean DISCERN score of the videos was 2.21 +/- 1. No appropriate references were cited in any of the videos. There was no video that received a full score (5 points) from DISCERN.ConclusionOur study shows that the level of quality in YouTube videos associated with robotic-assisted sacrocolpopexy is at an acceptable and high level, but its reliability is low. Therefore, short and concise videos that contain accurate information and refer to scientific facts should be prepared by urogynecology associations.

C1 [Ozcan, Cihat] Univ Hlth Sci, Gulhane Training & Res Hosp, Dept Urol, Ankara, Turkiye.

[Sanci, Adem] Kizilcahamam State Hosp, Dept Urol, Kizilcahamam, Turkiye.

C3 University of Health Sciences Turkey; Gulhane Training & Research

Hospital; Kizilcahamam State Hospital

RP Özcan, C (corresponding author), Univ Hlth Sci, Gulhane Training & Res Hosp, Dept Urol, Ankara, Turkiye.

EM dr.cihatozcan@gmail.com; dr.adem88@hotmail.com

RI SANCI, ADEM/AAS-6285-2021; ozcan, cihat/AAC-6161-2020

OI ozcan, cihat/0000-0003-1168-5503

NR 19

TC 0

Z9 0

U1 5

U2 7

PU SPRINGER LONDON LTD

PI LONDON

PA 236 GRAYS INN RD, 6TH FLOOR, LONDON WC1X 8HL, ENGLAND

SN 0937-3462

EI 1433-3023

J9 INT UROGYNECOL J

JI Int. Urogynecol. J.

PD AUG

PY 2023

VL 34

IS 8

BP 1843

EP 1847

DI 10.1007/s00192-023-05480-x

EA FEB 2023

PG 5

WC Obstetrics & Gynecology; Urology & Nephrology

WE Science Citation Index Expanded (SCI-EXPANDED)

SC Obstetrics & Gynecology; Urology & Nephrology

GA O9HB8

UT WOS:000931836800002

PM 36763146

DA 2024-01-18

ER

PT J

AU Roman, H

Dennis, T

Forestier, D

François, MO

Assenat, V

Chanavaz-Lacheray, I

Denost, Q

Merlot, B

AF Roman, Horace

Dennis, Thomas

Forestier, Damien

Francois, Marc Olivier

Assenat, Vincent

Chanavaz-Lacheray, Isabella

Denost, Quentin

Merlot, Benjamin

TI Excision of Deep Rectovaginal Endometriosis Nodules with Large

Infiltration of Both Rectum and Vagina: What Is a Reasonable Rate of

Preventive Stoma? A Comparative Study

SO JOURNAL OF MINIMALLY INVASIVE GYNECOLOGY

LA English

DT Article

DE Deep endometriosis; Rectum; Disc excision; Rectovaginal fistula; Stoma

ID LOW ANTERIOR RESECTION; SYMPTOMATIC ANASTOMOTIC LEAKAGE; COLORECTAL

RESECTION; DEFUNCTIONING STOMA; DIVERTING STOMA; CANCER; VALIDATION

AB Study Objective: To compare postoperative complications and rectovaginal fistula rate in women undergoing excision of large rectovaginal endometriosis requiring concomitant excision of rectum and vagina during 2 time periods with differing policies for preventive stoma confection.Design: Retrospective before-and-after comparative cohort study on data prospectively recorded in a database. Patients managed from September 2018 to March 2020 (first period) were compared with those managed from April 2020 to June 2022 (second period). Patients: One hundred sixty-eight patients presenting with deep endometriosis infiltrating the rectum and vagina, with lesions more than 3 cm in diameter during 2 consecutive time periods with differing policies regarding use of preventive stoma.Interventions: Rectal disc excision or colorectal resection, concomitantly with large vaginal excision.Measurements and Main Results: A total of 87 and 81 women received surgery during the first and the second period, respectively, during which the rate of preventive stoma was, respectively, 32.2% and 8.6%. Deep rectovaginal nodule characteristics were comparable. The mean height (SD) of rectal sutures after disc excision and colorectal resection were, respectively, 6.5 cm (2.3 cm) and 7.2 cm (3.8 cm). Rectovaginal fistula was recorded in 17 patients, corresponding to an overall rate of 10.1%. The rates of rectovaginal fistula in the group of patients with and without preventive stoma, regardless of the period in which surgery was performed, were 11.4% and 9.8%, respectively (p = .76). The rates of fistula recorded during the first and the second period were, respectively, 9.2% and 11.1% (p = .80), and that of overall early main complications were 31% and 29.6% (p = .84). Regression logistic model identified an independent relationship between smoking and rectovaginal fistula (adjusted odds ratio [OR] 3.9, 95% confidence interval [CI] 1.1-14) after adjustment for the period (adjusted OR 1.4, 95% CI 0.4-4.9 related to the second period), stoma confection (adjusted OR 1.8, 95% CI 0.5-7.1 related to stoma confection), robotic surgery (adjusted OR 1.7, 95% CI 0.3-10.1 related to robotic assistance), and type of rectal surgery (adjusted OR 0.4, 95% CI 0.1-1.4 related to disc excision when compared with colorectal resection).Conclusion: No statistically significant differences were found concerning risk of rectovaginal fistula in women with rectovaginal endometriosis requiring large rectal and vaginal excision after a decision to no longer routinely perform preventive stoma. Journal of Minimally Invasive Gynecology (2023) 30, 147-155. (c) 2022 AAGL. All rights reserved.

C1 [Roman, Horace; Dennis, Thomas; Forestier, Damien; Francois, Marc Olivier; Chanavaz-Lacheray, Isabella; Merlot, Benjamin] Franco European Multidisciplinary Endometriosis I, Bordeaux, France.

[Francois, Marc Olivier; Assenat, Vincent; Denost, Quentin] Bordeaux Colorectal Inst, Bordeaux, France.

[Francois, Marc Olivier; Assenat, Vincent; Denost, Quentin] Clin Tivoli Ducos, Bordeaux, France.

[Roman, Horace] Aarhus Univ Hosp, Dept Gynecol & Obstet, Aarhus, Denmark.

[Roman, Horace] Clin Tivoli Ducos, IFEM Endo, 91 Rue Riviere, F-33000 Bordeaux, France.

C3 Aarhus University

RP Roman, H (corresponding author), Clin Tivoli Ducos, IFEM Endo, 91 Rue Riviere, F-33000 Bordeaux, France.

EM horace.roman@gmail.com

OI Roman, Horace/0000-0002-9237-0628

NR 19

TC 1

Z9 1

U1 1

U2 2

PU ELSEVIER SCIENCE INC

PI NEW YORK

PA STE 800, 230 PARK AVE, NEW YORK, NY 10169 USA

SN 1553-4650

EI 1553-4669

J9 J MINIM INVAS GYN

JI J. Minim. Invasive Gynecol.

PD FEB

PY 2023

VL 30

IS 2

DI 10.1016/j.jmig.2022.11.006

EA FEB 2023

PG 9

WC Obstetrics & Gynecology

WE Science Citation Index Expanded (SCI-EXPANDED)

SC Obstetrics & Gynecology

GA 8Y3UM

UT WOS:000932626300001

PM 36402380

DA 2024-01-18

ER

PT J

AU Xu, ZY

Li, LX

Wang, XG

Wang, MX

Cao, GJ

Chen, BL

Liu, SJ

AF Xu, Zhi-yang

Li, Ling-xia

Wang, Xing-guo

Wang, Meng-xin

Cao, Gai-jing

Chen, Bi-liang

Liu, Shu-juan

TI The long-term outcomes of vaginoplasty using acellular porcine small

intestinal submucosa grafts in patients with

Mayer-Rokitansky-Kuster-Hauser syndrome: A case series

SO BJOG-AN INTERNATIONAL JOURNAL OF OBSTETRICS AND GYNAECOLOGY

LA English

DT Article

DE Mayer-Rokitansky-Kuster-Hauser syndrome; psychological outcomes; sexual

function restoration; small intestinal submucosa grafts; vaginoplasty

ID FEMALE SEXUAL FUNCTION; INDEX

AB Objective: To investigate the long -term outcomes for Mayer- Rokitansky- Kuster- Hauser syndrome (MRKH) patients undergoing vaginoplasty using acellular porcine small intestinal submucosa grafts (SIS).Design: A case series.Population: Seventy -eight MRKH syndrome patients and a post- SIS patient who delivered a baby following the world's first robot-assisted uterus transplantation.Methods: Mayer- Rokitansky- Kuster- Hauser syndrome patients were grouped based on the postoperative time and the diagnosis- surgery interval. Outcomes of sexual function and psychological status were assessed using the female sexual function index (FSFI), self-rating scale of body image (SSBI) and self-acceptance questionnaire (SAQ). Anatomical outcomes were measured by clinicians.Main Outcome Measures: The primary outcome was restoration of sexual function, defined by an FSFI score in the "good' range. Anatomical and psychological outcomes were also analysed.Results: Sexual function was restored in 42.3% (33/78) of patients and the total FSFI score was 23.44 +/- 4.43. Three factors (body defect, recognition of physical appearance and willingness to change physical appearance scores) in the SSBI and two in the SAQ decreased as the postoperative time increased. Based on the interval between diagnosis and surgery, the total SSBI score was lower in the short-interval group than in the long-interval group (7.25 +/- 5.55 versus 12.04 +/- 10.21, p = 0.038).Conclusions: Nearly half of MRKH patients in our study had good long -term sexual function after SIS vaginoplasty. Sexual function and psychological status improved as postoperative time increased. In addition, reducing the diagnosis to surgery interval was associated with improved psychological function.

C1 [Xu, Zhi-yang; Li, Ling-xia; Wang, Xing-guo; Wang, Meng-xin; Cao, Gai-jing; Chen, Bi-liang; Liu, Shu-juan] Fourth Mil Med Univ, Xijing Hosp, Dept Obstet & Gynecol, Xian, Peoples R China.

[Cao, Gai-jing] Med Sch Yanan Univ, Yanan, Peoples R China.

C3 Air Force Military Medical University

RP Xu, ZY; Liu, SJ (corresponding author), Fourth Mil Med Univ, Xijing Hosp, Dept Gynecol, Dept Obstet & Gynecol, 129 Changle West Rd, Xian, Peoples R China.

EM hanliu@fmmu.edu.cn

FU Academic Boosting Program of Xijing Hospital, Fourth Military Medical

University [XJZT18Z01]

FX Academic Boosting Program of Xijing Hospital, Fourth Military Medical

University, Grant/Award Number: XJZT18Z01

NR 21

TC 0

Z9 0

U1 4

U2 7

PU WILEY

PI HOBOKEN

PA 111 RIVER ST, HOBOKEN 07030-5774, NJ USA

SN 1470-0328

EI 1471-0528

J9 BJOG-INT J OBSTET GY

JI BJOG

PD MAY

PY 2023

VL 130

IS 6

BP 645

EP 652

DI 10.1111/1471-0528.17388

EA FEB 2023

PG 8

WC Obstetrics & Gynecology

WE Science Citation Index Expanded (SCI-EXPANDED)

SC Obstetrics & Gynecology

GA D7EJ6

UT WOS:000934219100001

PM 36660801

OA hybrid

DA 2024-01-18

ER

PT J

AU Tappy, E

Pan, E

Corton, M

AF Tappy, Erryn

Pan, Evelyn

Corton, Marlene

TI Robotic Burch colposuspension: anatomical and technical considerations

SO INTERNATIONAL UROGYNECOLOGY JOURNAL

LA English

DT Article

DE Robot-assisted colposuspension; Surgical education; Stress urinary

incontinence

ID STRESS URINARY-INCONTINENCE; COOPERS LIGAMENT

AB Introduction and hypothesisUp to 13.6% of women will undergo surgical treatment for stress urinary incontinence during their lifetime. Midurethral slings are the mainstay of stress incontinence treatment; however, diversity of surgical options is needed to serve the large number of patients desiring treatment. The Burch colposuspension remains a viable treatment option for appropriately selected patients. Currently, information on procedural standardization and tools for surgical training on robot-assisted colposuspension is limited.MethodsWe describe a stepwise robotic approach aimed at enhancing procedural reproducibility, while decreasing risks of intraoperative injury and postoperative complications. We analyze perioperative outcomes of our technique in a retrospective cohort of patients who underwent robot-assisted colposuspension at our institution.ResultsSeven key procedural steps are defined to optimize safe dissection in the retropubic space and to reduce the potential for surgical complications. These include methods of avoiding bladder, urethral, and neurovascular injury, as well as enhancing adequate suture fixation that prevents urethral obstruction and adverse postoperative urinary and pain-related symptoms. Surgical outcomes for 20 patients are reported and reveal low rates of perioperative complications.ConclusionRobot-assisted colposuspension requires thorough knowledge of the retropubic space and the application of standardized techniques may reduce the risk of injury and optimize procedure efficiency and reproducibility.

C1 [Tappy, Erryn; Pan, Evelyn; Corton, Marlene] UT Southwestern Med Ctr, Dallas, TX 75390 USA.

C3 University of Texas System; University of Texas Southwestern Medical

Center Dallas

RP Tappy, E (corresponding author), UT Southwestern Med Ctr, Dallas, TX 75390 USA.

EM ErrynTappy@gmail.com

OI Pan, Evelyn/0000-0001-5806-1269

NR 14

TC 0

Z9 0

U1 0

U2 0

PU SPRINGER LONDON LTD

PI LONDON

PA 236 GRAYS INN RD, 6TH FLOOR, LONDON WC1X 8HL, ENGLAND

SN 0937-3462

EI 1433-3023

J9 INT UROGYNECOL J

JI Int. Urogynecol. J.

PD JUL

PY 2023

VL 34

IS 7

BP 1653

EP 1657

DI 10.1007/s00192-023-05452-1

EA FEB 2023

PG 5

WC Obstetrics & Gynecology; Urology & Nephrology

WE Science Citation Index Expanded (SCI-EXPANDED)

SC Obstetrics & Gynecology; Urology & Nephrology

GA J9VY8

UT WOS:000926340900002

PM 36745132

DA 2024-01-18

ER

PT J

AU El Haraki, AS

Shepherd, JP

Parker-Autry, C

Matthews, CA

AF El Haraki, Amr S.

Shepherd, Jonathan P.

Parker-Autry, Candace

Matthews, Catherine A.

TI Financial analysis of minimally invasive sacrocolpopexy compared with

native tissue vaginal repair with concomitant hysterectomy

SO INTERNATIONAL UROGYNECOLOGY JOURNAL

LA English

DT Article

DE Cost; ERAS; Sacrocolpopexy; Minimally invasive; Uterosacral;

Sacrospinous; Native tissue repair; Robotic

ID PELVIC ORGAN PROLAPSE; COST-ANALYSIS; LAPAROSCOPIC SACROCOLPOPEXY;

LIFETIME RISK

AB IntroductionMinimally invasive sacrocolpopexy (MISCP) is increasingly used for uterovaginal prolapse, but comparative cost data of MISCP versus native tissue vaginal repair (NTR) are lacking. The objective was to determine the cost difference, from a hospital perspective, between MISCP and NTR performed with hysterectomy for uterovaginal prolapse.MethodsThis was a retrospective cohort study at a tertiary care center of women who underwent NTR or MISCP with concomitant hysterectomy in 2021. Hospital charges, direct and indirect costs, and operating margin (revenue minus costs) were obtained from Strata Jazz and compared using SPSS.ResultsA total of 82 women were included, 33 MISCP (25 robotic, 8 laparoscopic) versus 49 NTR. Demographic and surgical data were similar, except that MISCP had younger age (50.5 vs 61.1 years, p<0.01). Same-day discharge and estimated blood loss were similar, but operative time was longer for MISCP (204 vs 161 min, p<0.01). MISCP total costs were higher (US$17,422 vs US$13,001, p<0.01). MISCP had higher direct costs (US$12,354 vs US$9,305, p<0.01) and indirect costs (US$5,068 vs US$3,696, p<0.01). Consumable supply costs were higher with MISCP (US$4,429 vs US$2,089, p<0.01), but the cost of operating room time and staff was similar (US$7,926 vs US$7,216, p=0.07). Controlling for same-day discharge, anti-incontinence procedures and smoking, total costs were higher for MISCP (adjusted beta = US$4,262, p<0.01). Mean charges (US$102,060 vs US$97,185, p=0.379), revenue (US$22,214 vs US$22,491, p=0.929), and operating margin (US$8,719 vs US$3,966, p=0.134) were not statistically different.ConclusionMinimally invasive sacrocolpopexy had higher costs than NTR; however, charges, reimbursement, and operating margins were not statistically significantly different between the groups.

C1 [El Haraki, Amr S.; Parker-Autry, Candace; Matthews, Catherine A.] Wake Forest Baptist Med Ctr, Dept Urol, Winston Salem, NC 27103 USA.

[El Haraki, Amr S.; Parker-Autry, Candace; Matthews, Catherine A.] Wake Forest Baptist Med Ctr, Dept Obstet & Gynecol, Winston Salem, NC USA.

[Shepherd, Jonathan P.] Univ Connecticut, Dept Obstet & Gynecol, Hlth Ctr, Farmington, CT USA.

C3 Wake Forest University; Wake Forest Baptist Medical Center; Wake Forest

University; Wake Forest Baptist Medical Center; University of

Connecticut

RP Matthews, CA (corresponding author), Wake Forest Baptist Med Ctr, Dept Urol, Winston Salem, NC 27103 USA.

EM camatthe@wakehealth.edu

OI El Haraki, Amr/0000-0001-8412-5967

NR 18

TC 1

Z9 1

U1 0

U2 1

PU SPRINGER LONDON LTD

PI LONDON

PA 236 GRAYS INN RD, 6TH FLOOR, LONDON WC1X 8HL, ENGLAND

SN 0937-3462

EI 1433-3023

J9 INT UROGYNECOL J

JI Int. Urogynecol. J.

PD MAY

PY 2023

VL 34

IS 5

BP 1121

EP 1126

DI 10.1007/s00192-022-05445-6

EA FEB 2023

PG 6

WC Obstetrics & Gynecology; Urology & Nephrology

WE Science Citation Index Expanded (SCI-EXPANDED)

SC Obstetrics & Gynecology; Urology & Nephrology

GA AJ6M9

UT WOS:000924617300001

PM 36729164

OA Bronze, Green Published

DA 2024-01-18

ER

PT J

AU Jansen, SM

Ghatalia, D

Mayo, A

Lokke, A

Petersen, TR

Serna-Gallegos, T

Meriwether, KV

AF Jansen, Sierra M.

Ghatalia, Desna

Mayo, Alyssa

Lokke, Amanda

Petersen, Timothy R.

Serna-Gallegos, Tasha

Meriwether, Kate V.

TI Factors Associated With Increased Estimated Blood Loss and Factors

Associated With Utilization of Type and Screen in Benign Gynecology: A

Retrospective Chart Review

SO UROGYNECOLOGY

LA English

DT Review

ID ENHANCED RECOVERY

AB Importance There is minimal literature discussing factors associated with increased estimated blood loss (EBL) or transfusion in gynecologic surgery in tertiary academic centers.Objective The aim of the study was to determine factors associated with transfusion and increased blood loss during gynecologic surgery.Study Design This retrospective cohort investigated patients undergoing benign gynecologic procedures at a tertiary medical center. We excluded women undergoing surgery for known or suspected malignancy, emergent surgery, obstetrical procedures, or cases with another surgical specialty. Patient age, body mass index, American Society of Anesthesiologists class, medical history, EBL, arterial line placement, preoperative laboratory studies, and transfusion receipt for up to 6 weeks postoperatively were extracted. The primary outcome was transfusion within 6 weeks of surgery; risk factors for high blood loss (EBL > 500 mL) and transfusion were explored.Results Nine hundred seventy-five surgical procedures were included (59% vaginal, 36% laparoscopic, 4% robotic). Median EBL was 50 mL (interquartile range, 10-100 mL). Estimated blood loss increased with duration of surgery (P < 0.01). Transfusions were more likely to occur during open procedures (13%) compared with vaginal (2%), laparoscopic (2%), or robotic (3%). Arterial line placement (relative risk [RR], 11.8; 95% confidence interval [CI], 5.3-26.1) and additional intravenous placement (RR, 6.0; 95% CI, 2.6 to 13.7) were associated with transfusion. Vaginal surgery (RR, 0.13; 95% CI, 0.05 to 0.32) and urogynecologic procedures (RR, 0.1; CI, 0.01-0.7) were associated with reduced risk of needing transfusion.Conclusions Most benign gynecologic surgical procedures have minimal blood loss. Patients undergoing surgery through minimally invasive routes or urogynecologic procedures are at further decreased risk of transfusion.

C1 [Jansen, Sierra M.] HealthPartners, Dept Obstet & Gynecol, Bloomington, MN 55420 USA.

[Ghatalia, Desna; Lokke, Amanda] Univ New Mexico, Sch Med, Albuquerque, NM USA.

[Mayo, Alyssa; Petersen, Timothy R.] Univ New Mexico, Dept Obstet & Gynecol, Albuquerque, NM USA.

[Petersen, Timothy R.] Univ New Mexico, Dept Anesthesiol, Albuquerque, NM USA.

[Serna-Gallegos, Tasha; Meriwether, Kate V.] Univ New Mexico, Div Female Pelv Med & Reconstruct Surg, Albuquerque, NM USA.

C3 HealthPartners Institute for Education & Research; University of New

Mexico; University of New Mexico; University of New Mexico; University

of New Mexico

RP Jansen, SM (corresponding author), HealthPartners, Dept Obstet & Gynecol, Bloomington, MN 55420 USA.

EM sierramariette@gmail.com; dvghatalia@salud.unm.edu;

almayo@salud.unm.edu; alokke36@siumed.edu; TimPetersen@salud.unm.edu;

Tsernagallegos@gmail.com; meriwet2@salud.unm.edu

RI Petersen, Timothy/IUP-9067-2023

OI Petersen, Timothy/0000-0002-2483-5614

NR 14

TC 0

Z9 0

U1 0

U2 0

PU LIPPINCOTT WILLIAMS & WILKINS

PI PHILADELPHIA

PA TWO COMMERCE SQ, 2001 MARKET ST, PHILADELPHIA, PA 19103 USA

EI 2771-1897

J9 UROGYNECOLOGY

JI Urogynecol.

PD FEB

PY 2023

VL 29

IS 2

BP 195

EP 201

DI 10.1097/SPV.0000000000001275

PG 7

WC Obstetrics & Gynecology

WE Science Citation Index Expanded (SCI-EXPANDED)

SC Obstetrics & Gynecology

GA 8J9UD

UT WOS:000922756300015

PM 36735434

DA 2024-01-18

ER

PT J

AU Sighinolfi, MC

Gaia, G

Afonina, M

Assumma, S

Calcagnile, T

Garelli, G

Sangalli, M

Guarnerio, PP

Felline, M

Eissa, A

Sarchi, L

Terzoni, S

Micali, S

Marconi, A

Rocco, B

AF Sighinolfi, Maria Chiara

Gaia, Giorgia

Afonina, Margarita

Assumma, Simone

Calcagnile, Tommaso

Garelli, Giulia

Sangalli, Mattia

Guarnerio, Paolo Pasquale

Felline, Mauro

Eissa, Ahmed

Sarchi, Luca

Terzoni, Stefano

Micali, Salvatore

Marconi, Annamaria

Rocco, Bernardo

TI Level 1 Evidence for Robotic Surgery for Urological and Gynecological

Pelvic Cancers: Where do We Currently Stand?

SO CLINICAL AND EXPERIMENTAL OBSTETRICS & GYNECOLOGY

LA English

DT Article

DE robotic surgery; randomized controlled trial; pelvic cancer; prostate

cancer; bladder cancer; endometrial cancer; cervical cancer

ID ASSISTED RADICAL CYSTECTOMY; ENDOMETRIAL

AB Robotic surgery is used for the surgical removal of female pelvic malignancies and encompasses procedures as radical cystectomy and radical hysterectomy. The aim of this paper is to provide an update of level 1 literature evidence about the outcomes of robotic surgery compared to other surgical approaches for the treatment of bladder, endometrial and cervical cancer. A non-systematic search of the PubMed and Scopus databases was conducted to identify peer-reviewed randomized controlled trials (RCTs) comparing surgical approaches for radical cystectomy and hysterectomy. To the purpose of capturing the lastest updates, 2020-2022 literature was reviewed. In the field of radical cystectomy, two RCTs supported the implementation of robotics as a more beneficial approach than open surgery -in terms of faster recovery, less thromboembolic events, less infectious events. In gynecology, despite robotics is accepted for the treatment of early endometrial tumors, the role of minimally invasive surgery (MIS) for the treatment of cervical cancer is still debated, with two recent systematic reviews and meta-analyses reporting conflicting results. Two-decades after the introduction of robotic surgery, there is still a number of current studies evaluating its role for the treatment of urological pelvic malignancies, especially for bladder cancer. The role of robotic surgery alone for the treatment of gynecological malignancies has been scarcely addressed with robotics being mostly evaluated as a part of MIS; updates about MIS for the treatment of cervical cancer continue to be ongoing.

C1 [Sighinolfi, Maria Chiara; Assumma, Simone; Calcagnile, Tommaso; Garelli, Giulia; Sangalli, Mattia; Rocco, Bernardo] ASST Santi Paolo & Carlo, San Paolo Hosp Med Sch, Unit Urol, I-20142 Milan, Italy.

[Gaia, Giorgia; Afonina, Margarita; Marconi, Annamaria] ASST Santi Paolo & Carlo, San Paolo Hosp Med Sch, Unit Obstet & Gynecol, I-20142 Milan, Italy.

[Guarnerio, Paolo Pasquale; Felline, Mauro] ASST Santi Paolo & Carlo, San Carlo Hosp, Unit Obstet & Gynecol, I-20153 Milan, Italy.

[Eissa, Ahmed] Tanta Univ, Dept Urol, Tanta 31527, Egypt.

[Sarchi, Luca] Orsi Acad, Dept Urol, B-82822 Melle, Belgium.

[Terzoni, Stefano] ASST Santi Paolo & Carlo, San Paolo Hosp Med Sch, San Paolo Bachelor Sch Nursing, I-20142 Milan, Italy.

[Micali, Salvatore] Univ Modena & Reggio Emilia, Unit Urol, I-41100 Modena, Italy.

[Marconi, Annamaria; Rocco, Bernardo] Univ Milan, Dept Hlth Sci, I-20142 Milan, Italy.

C3 San Carlo Borromeo Hospital; Egyptian Knowledge Bank (EKB); Tanta

University; Universita di Modena e Reggio Emilia; University of Milan

RP Sighinolfi, MC (corresponding author), ASST Santi Paolo & Carlo, San Paolo Hosp Med Sch, Unit Urol, I-20142 Milan, Italy.

EM sighinolfic@gmail.com

RI Terzoni, Stefano/AAH-7582-2021

OI Terzoni, Stefano/0000-0002-0716-5663; Afonina,

Margarita/0000-0002-7126-3866

NR 21

TC 0

Z9 0

U1 0

U2 0

PU IMR PRESS

PI ROBINSON

PA 112 ROBINSON RD, ROBINSON, SINGAPORE

SN 0390-6663

EI 2709-0094

J9 CLIN EXP OBSTET GYN

JI Clin. Exp. Obstet. Gynecol.

PD FEB

PY 2023

VL 50

IS 2

AR 44

DI 10.31083/j.ceog5002044

PG 4

WC Obstetrics & Gynecology

WE Science Citation Index Expanded (SCI-EXPANDED)

SC Obstetrics & Gynecology

GA H4XB7

UT WOS:000995998900008

OA gold

DA 2024-01-18

ER

PT J

AU Zuo, SW

Carter-Brooks, CM

Zyczynski, HM

Ackenbom, MF

AF Zuo, Stephanie W. W.

Carter-Brooks, Charelle M.

Zyczynski, Halina M.

Ackenbom, Mary F.

TI Frailty and Acute Postoperative Urinary Retention in Older Women

Undergoing Pelvic Organ Prolapse Surgery

SO UROGYNECOLOGY

LA English

DT Article

ID MALNUTRITION; DISABILITY

AB Importance Acute postoperative urinary retention (POUR) is common after pelvic reconstructive surgery, occurring in 15-45% of women. There is a paucity of data on the relationship between frailty and POUR after prolapse surgery.Objective This study aimed to examine the association between frailty and POUR in older women who underwent pelvic organ prolapse surgery.Study Design This secondary analysis of a prospective study of postoperative delirium enrolled women 60 years and older undergoing prolapse surgery. The Fried Frailty Index was used to assess frailty before surgery. Acute POUR was defined as failure to pass a retrograde voiding trial at hospital discharge with postvoid residual volume of greater than 100 mL.Results Analyses included 165 women, with a mean & PLUSMN; SD age of 72.5 +/- 6.1 years and a body mass index of 28.0 +/- 4.4 kg/m(2). There were 49 laparoscopic/robotic apical suspension procedures (29.7%), 60 vaginal obliterative procedures (36.4%), 47 vaginal apical suspension procedures (28.5%), and 9 isolated anterior and/or posterior colporrhaphies (5.5%), of which 9 had a concomitant incontinence procedure. Seventy-eight women (47.3%) experienced acute POUR. Thirty-one (18.8%) met the criteria for "not frail, " 115 (88.5%) were "prefrail, " and 19 (11.5%) were "frail. " Neither frailty status nor score was associated with POUR. In an analysis of individual Fried Frailty Index components, self-reported unintentional weight loss was significantly associated with POUR (odds ratio, 4.6; 95% confidence interval, 1.23-17.15). This remained significant on multivariable logistic regression (adjusted odds ratio, 4.06; 95% confidence interval, 1.01-16.39).Conclusions Frailty was not associated with POUR in older women undergoing prolapse surgery. The observed association between POUR and unintended weight loss before surgery warrants further investigation.

C1 [Zuo, Stephanie W. W.; Zyczynski, Halina M.; Ackenbom, Mary F.] Univ Pittsburgh, Med Ctr, Dept Obstet Gynecol & Reprod Sci, Div Urogynecol, Pittsburgh, PA 15260 USA.

[Carter-Brooks, Charelle M.] George Washington Sch Med & Hlth Sci, Dept Obstet & Gynecol, Washington, DC USA.

C3 Pennsylvania Commonwealth System of Higher Education (PCSHE); University

of Pittsburgh; George Washington University

RP Zuo, SW (corresponding author), Univ Pittsburgh, Med Ctr, Dept Obstet Gynecol & Reprod Sci, Div Urogynecol, Pittsburgh, PA 15260 USA.

EM zuos@upmc.edu; chcarterbrooks@mfa.gwu.edu; zyczhm@mwri.magee.edu;

ackenbommf@upmc.edu

RI Zuo, Stephanie/IAP-8564-2023

OI Zuo, Stephanie/0000-0003-0067-9159

FU National Institute on Aging [R03AG064378-01, K23AG073517-01];

Pennsylvania Department of Health [4100088553]

FX M.F.A. is supported by the National Institute on Aging (R03AG064378-01,

K23AG073517-01) and the Pennsylvania Department of Health (4100088553).

M.F.A.'s spouse is employed by Johnson & Johnson Corporate. The rest of

the authors have declared no conflict of interest.

NR 33

TC 0

Z9 0

U1 2

U2 2

PU LIPPINCOTT WILLIAMS & WILKINS

PI PHILADELPHIA

PA TWO COMMERCE SQ, 2001 MARKET ST, PHILADELPHIA, PA 19103 USA

EI 2771-1897

J9 UROGYNECOLOGY

JI Urogynecol.

PD FEB

PY 2023

VL 29

IS 2

BP 168

EP 174

DI 10.1097/SPV.0000000000001289

PG 7

WC Obstetrics & Gynecology

WE Science Citation Index Expanded (SCI-EXPANDED)

SC Obstetrics & Gynecology

GA 8J9UD

UT WOS:000922756300011

PM 36735430

DA 2024-01-18

ER

PT J

AU Won, S

Choi, SH

Kim, SJ

Lee, N

Shim, SH

Kim, M

Kim, MK

Jung, YW

Yun, BS

Seong, SJ

Kim, ML

AF Won, Seyeon

Choi, Su Hyeon

Kim, Su Jin

Lee, Nara

Shim, So Hyun

Kim, Miseon

Kim, Mi Kyoung

Jung, Yong Wook

Yun, Bo Seong

Seong, Seok Ju

Kim, Mi-La

TI Comparison of cesarean delivery outcome after robotic and laparoscopic

myomectomy

SO TAIWANESE JOURNAL OF OBSTETRICS & GYNECOLOGY

LA English

DT Article

DE Laparoscopy; Robotic surgical procedures; Uterine fibroids; Uterine

myomectomy

ID PREGNANCY OUTCOMES; ADHESION FORMATION; FERTILITY; MYOMAS

AB Objective: The aim of this study was to determine whether robotic myomectomy (RM) resulted in any measurable clinical improvement over laparoscopic myomectomy (LM) in subsequent cesarean delivery.Materials and methods: The medical records of 273 patients who had undergone LM or RM followed by subsequent cesarean delivery for the period of September 2015 to December 2020 were retrospectively reviewed. The patients were divided into LM (n = 222) and RM (n = 51) groups. The cesarean delivery outcomes between the two groups were compared. Results: RM had significantly more myomas removed (6.0 +/- 4.8 vs. 3.6 +/- 3.5, p < 0.001) and a larger size of largest myoma (7.7 +/- 2.4 vs. 6.1 +/- 2.4, p = 0.002) at myomectomy compared with LM. However, there were no significant differences in the groups' surgical characteristics at cesarean section, in their pregnancy complications, or in adhesion formation.Conclusions: Although more and larger myomas were removed in the RM group, RM showed similar cesarean delivery outcomes and adhesion formation to LM.(c) 2023 Taiwan Association of Obstetrics & Gynecology. Publishing services by Elsevier B.V. This is an open access article under the CC BY-NC-ND license (http://creativecommons.org/licenses/by-nc-nd/4.0/).

C1 [Won, Seyeon; Choi, Su Hyeon; Kim, Su Jin; Lee, Nara; Shim, So Hyun; Kim, Miseon; Kim, Mi Kyoung; Jung, Yong Wook; Seong, Seok Ju; Kim, Mi-La] CHA Univ, CHA Gangnam Med Ctr, Dept Obstet & Gynecol, Sch Med, Seoul, South Korea.

[Yun, Bo Seong] CHA Univ, CHA Ilsan Med Ctr, Dept Obstet & Gynecol, Sch Med, Goyang, South Korea.

[Kim, Mi-La] CHA Univ, CHA Gangnam Med Ctr, Dept Obstet & Gynecol, 566 Nonhyeon Ro, Seoul 06135, South Korea.

C3 Pochon Cha University; Pochon Cha University; Pochon Cha University

RP Kim, ML (corresponding author), CHA Univ, CHA Gangnam Med Ctr, Dept Obstet & Gynecol, 566 Nonhyeon Ro, Seoul 06135, South Korea.

EM mila76@naver.com

NR 22

TC 2

Z9 2

U1 0

U2 0

PU ELSEVIER TAIWAN

PI TAIPEI

PA RM N-412, 4F, CHIA HSIN BUILDING 11, NO 96, ZHONG SHAN N ROAD SEC 2,

TAIPEI, 10449, TAIWAN

SN 1028-4559

J9 TAIWAN J OBSTET GYNE

JI Taiwan. J. Obstet. Gynecol.

PD JAN

PY 2023

VL 62

IS 1

BP 12

EP 15

DI 10.1016/j.tjog.2022.06.016

EA JAN 2023

PG 4

WC Obstetrics & Gynecology

WE Science Citation Index Expanded (SCI-EXPANDED)

SC Obstetrics & Gynecology

GA D4TW0

UT WOS:000968684000001

PM 36720523

OA gold

DA 2024-01-18

ER

PT J

AU Buderath, P

Kimmig, R

Dominowski, L

Mach, P

AF Buderath, Paul

Kimmig, Rainer

Dominowski, Lisa

Mach, Pawel

TI Hysterectomy over the course of time. Retrospective analysis of surgical

techniques at the University Gynecology Hospital Essen over the last 20

years

SO GYNAKOLOGIE

LA German

DT Article; Early Access

DE Laparoscopy; Laparotomy; Intraoperative complications; Postoperative

complications; Robotic surgical procedures

ID LAPAROSCOPIC HYSTERECTOMY; ASSISTED HYSTERECTOMY; SURGERY

AB Introduction: Minimally invasive surgery has for a long time been the standard for performing hysterectomy for benign indications. In this context robotic-assisted operations are generally considered comparable to conventional laparoscopy. Furthermore, robot-assisted surgery has the potential to also enable complex minimally invasive interventions and to make laparotomy unnecessary in the majority of patients.Material and methods: A total of 1939 patients who underwent hysterectomy for benign indications at the University Gynecology Hospital Essen between 2002 and 2020 were identified. Perioperative and postoperative data and patient characteristics were retrospectively collated.Results: Robotic surgery has been carried out in this hospital since 2010 and was the most frequent surgical approach (n = 771; 39.8%) and 60.2% (1168/1938) of hysterectomies were carried out using minimally invasive surgery. Over the years there was a clear shift with respect to the access route. In 2002, 51.4% of all hysterectomies were carried out using an open procedure but in 2020 this was reduced to 1.4%. Correspondingly, the proportion of minimally invasive procedures rose from 18.9% in 2002 to 98.6% in 2020. The introduction of robotic surgery in 2010 led to a significant shift to minimally invasive procedures, which led to shorter times in bed and less postoperative complications compared to laparotomy. Furthermore, this cohort includes the largest uterine myoma ever reported in the scientific literature with a weight of 54.8 kg.Conclusion: These data support the hypothesis that the introduction of robotic surgery expands the possibilities for minimally invasive surgery, whereby laparotomy can be avoided in nearly all cases. The known advantages of minimally invasive surgery could be confirmed.

C1 [Buderath, Paul; Kimmig, Rainer; Dominowski, Lisa; Mach, Pawel] Univ Duisburg Essen, Klin Frauenheilkunde & Geburtshilfe, Westdeutsches Tumorzentrum, Univ Klinikum Essen, Essen, Germany.

[Buderath, Paul] Univ Duisburg Essen, Klin Frauenheilkunde & Geburtshilfe, Westdeutsches Tumorzentrum, Univ Klinikum Essen, Hufelandstr 55, D-45147 Essen, Germany.

C3 University of Duisburg Essen; University of Duisburg Essen

RP Buderath, P (corresponding author), Univ Duisburg Essen, Klin Frauenheilkunde & Geburtshilfe, Westdeutsches Tumorzentrum, Univ Klinikum Essen, Hufelandstr 55, D-45147 Essen, Germany.

EM paul.buderath@uk-essen.de

NR 17

TC 0

Z9 0

U1 0

U2 0

PU SPRINGER HEIDELBERG

PI HEIDELBERG

PA TIERGARTENSTRASSE 17, D-69121 HEIDELBERG, GERMANY

SN 2731-7102

EI 2731-7110

J9 GYNAKOLOGIE

JI Gynakologie

PD 2023 JAN 26

PY 2023

DI 10.1007/s00129-022-05050-0

EA JAN 2023

PG 5

WC Obstetrics & Gynecology

WE Emerging Sources Citation Index (ESCI)

SC Obstetrics & Gynecology

GA 8X4MK

UT WOS:000931988200002

DA 2024-01-18

ER

PT J

AU Lindfors, A

Järvholm, S

Dahm-Kähler, P

AF Lindfors, Anna

Jarvholm, Stina

Dahm-Kahler, Pernilla

TI Health-related quality of life after robotic surgery for endometrial

cancer: a prospective longitudinal one-year follow-up study

SO ARCHIVES OF GYNECOLOGY AND OBSTETRICS

LA English

DT Article

DE Quality of life; Mental health; Depression; Anxiety; Endometrial cancer;

Robotic surgical procedures

ID ASSISTED LAPAROSCOPIC HYSTERECTOMY; EUROPEAN-ORGANIZATION;

CLINICAL-TRIALS; GUIDELINES; QLQ-C30

AB PurposeThis study aimed to explore how patients treated for endometrial cancer (EC) with robotic surgery are affected in symptoms of anxiety and depression and HRQoL in the long term.MethodsWomen scheduled for primary robotic surgery for EC were included (n = 64), in this single-center study. Socioeconomic variables were obtained at baseline. The European Organization for Research and Treatment of Cancers Quality of Life Questionnaire Core 30 (QLQ-C30), its module for EC (EN24), the Generalized Anxiety Disorder Scale (GAD-7), and the Patient Health Questionnaire Depression Scale (PHQ-9) were followed prospectively from baseline to 2 weeks, 3 months and 1 year postoperatively.ResultsThe number of patients scoring above the clinical threshold for anxiety decreased from 17 (27.0%) at baseline to 4 (7.0%) at 2 weeks (p = 0.012). Depressive symptoms were reported in 20% of patients at baseline and did not change significantly during the one-year follow-up (p = 0.58). A significant decrease in Global health status was seen at 2 weeks (from 69.8 to 62.7; p = 0.048), with return to baseline levels after 3 months (68.5; p = 0.32) and stable at 1 year. Unemployment, low income, and adjuvant therapy correlated with lower Global health status at 3 months.ConclusionThe significant proportion of patients with anxiety symptoms preoperatively reduced prompt after surgery, while the proportion with depression remained constant, indicating that the primary treatment has no long-term negative effect on patients' mental health. At 3 months, there is no obvious remaining negative impact on patients' HRQoL, and these results are consistent after 1 year.

C1 [Lindfors, Anna; Jarvholm, Stina; Dahm-Kahler, Pernilla] Univ Gothenburg, Inst Clin Sci, Sahlgrenska Acad, Dept Obstet & Gynecol, S-41345 Gothenburg, Sweden.

[Lindfors, Anna; Jarvholm, Stina; Dahm-Kahler, Pernilla] Sahlgrens Univ Hosp, Dept Gynecol, Gothenburg, Sweden.

C3 University of Gothenburg; Sahlgrenska University Hospital

RP Lindfors, A (corresponding author), Univ Gothenburg, Inst Clin Sci, Sahlgrenska Acad, Dept Obstet & Gynecol, S-41345 Gothenburg, Sweden.; Lindfors, A (corresponding author), Sahlgrens Univ Hosp, Dept Gynecol, Gothenburg, Sweden.

EM anna.m.lindfors@vgregion.se

OI Lindfors, Anna/0000-0002-9491-4406

FU University of Gothenburg; Swedish Cancer Society [CAN2017/594, 201346

PjF]; Swedish state under the ALF [ALFGBG-813171/965702]; Hjalmar

Svensson Foundation [HJSV2019058]; Assar Gabrielsson Foundation

[FB17-65]; Goeteborg Medical Society [689021/17]

FX Open access funding provided by University of Gothenburg. This work was

supported by the Swedish Cancer Society (CAN2017/594; 201346 PjF),

Grants from the Swedish state under the ALF agreement between the

Swedish government and the county councils (ALFGBG-813171/965702), the

Hjalmar Svensson Foundation (HJSV2019058), the Assar Gabrielsson

Foundation (FB17-65), and the Goeteborg Medical Society (689021/17). The

funders had no role in the conduct of this research.

NR 28

TC 0

Z9 0

U1 1

U2 2

PU SPRINGER HEIDELBERG

PI HEIDELBERG

PA TIERGARTENSTRASSE 17, D-69121 HEIDELBERG, GERMANY

SN 0932-0067

EI 1432-0711

J9 ARCH GYNECOL OBSTET

JI Arch. Gynecol. Obstet.

PD AUG

PY 2023

VL 308

IS 2

BP 515

EP 525

DI 10.1007/s00404-023-06917-w

EA JAN 2023

PG 11

WC Obstetrics & Gynecology

WE Science Citation Index Expanded (SCI-EXPANDED)

SC Obstetrics & Gynecology

GA L1AB6

UT WOS:000919929700001

PM 36694036

OA Green Published, hybrid

DA 2024-01-18

ER

PT J

AU Chakraborty, N

Rhodes, S

Luchristt, D

Bretschneider, CE

Sheyn, D

AF Chakraborty, Natalie

Rhodes, Stephen

Luchristt, Douglas

Bretschneider, C. Emi

Sheyn, David

TI Is total laparoscopic hysterectomy with longer operative time associated

with a decreased benefit compared with total abdominal hysterectomy?

SO AMERICAN JOURNAL OF OBSTETRICS AND GYNECOLOGY

LA English

DT Article

DE hysterectomy; operative time; morbidity

ID AMERICAN-COLLEGE; SURGICAL VOLUME; MORBIDITY; OUTCOMES; MORTALITY;

COMPLICATIONS; METAANALYSIS; SURGERY; INDEX

AB BACKGROUND: It is well known that, in general, total laparoscopic hysterectomy is associated with less perioperative morbidity compared with total abdominal hysterectomy. However, total laparoscopic hysterectomy is also associated with longer operating times, which itself is an independent predictor of morbidity. Currently, it is unknown whether there is an operative time threshold beyond which total laparoscopic hysterectomy provides a diminishing return and higher risk of morbidity than a shorter abdominal hysterectomy.OBJECTIVE: This study aimed to determine whether there is an operative time limit beyond which the benefits of total laparoscopic hysterectomy diminished compared with shorter total abdominal hysterectomy.STUDY DESIGN: Targeted hysterectomy-specific data from the National Surgical Quality Improvement Project was used to identify patients undergoing total laparoscopic hysterectomy and total abdominal hysterectomy for benign indications between the years 2014 and 2018. The primary outcomes of interest were any major morbidity, and the length of stay after surgery was analyzed using generalized linear models. The models controlled for demographic data, comorbidities, and hysterectomy-specific information, such as uterine weight, presence of endometriosis, and pelvic inflammatory disease at the time of surgery. Missing data were addressed using multiple imputation analysis. Sensitivity analyses using propensity score matching and generalized additive models were per-formed to assess the effect of selection bias and nonlinear interactions between covariates and the outcomes, respectively. Common Procedural Terminology codes were used to identify women who underwent total abdominal hysterectomy (n=58,152) or total laparoscopic hysterectomy (n=58,570-58,573). Conventional laparoscopy could not be differenti-ated from robotic surgery as there is no mechanism for doing so within the National Surgical Quality Improvement Project. Therefore, total laparo-scopic hysterectomy also includes robotic-assisted surgery. Additional exclusion criteria included any surgery lasting > 360 minutes, as these represent significant outliers in the data and clinical practice; pelvic reconstructive procedure; anti-incontinence surgery; lymphadenectomy; radical hysterectomy; cytoreductive surgery; a pre-or postoperative diagnostic code for gynecologic malignancy; preoperative sepsis or renal failure; emergency surgery; or any concurrent nongynecologic surgery. Patients who underwent ureteral stenting during the procedure with no additional urologic procedures were included, as this may be performed at the time of hysterectomy or to address ureteral injury.RESULTS: The mean operating time was similar for both routes, 129 +/- 60 minutes for total laparoscopic hysterectomy and 129 +/- 64 minutes for total abdominal hysterectomy (P=.45). The complication rate was higher for total abdominal hysterectomy than total laparoscopic hysterectomy (16.6% vs 7.7%; P <.001); and the median length of stay was longer for total abdominal hysterectomy (2 [interquartile range, 2-3] days vs 1 [interquartile range, 0-1] days; P <.001).

After adjusting for confounders, an increase of 1 hour in operative time for hysterectomy was associated with a 45% (95% confidence interval, 41%-49%) increase in the risk of major morbidity; furthermore, total abdominal hysterectomy was associated with an additional time detriment, such that there was an additional 61% (95% confidence interval, 53%-68%) increase in the risk of a major morbidity for each additional hour of a total abdominal hysterectomy. There was no time point at which total abdominal hysterectomy was associated with less morbidity or a shorter length of stay than total laparoscopic hysterectomy, even if total laparoscopic hysterectomy was significantly longer than total abdominal hysterectomy. The same conclusions remained true with the propensity-matched analysis and generalized additive model analyses.CONCLUSION: Our findings showed that there is no reasonable operative time at which total laparoscopic hysterectomy is associated with a higher rate of complications or longer length of stay than total abdominal hysterectomy.

C1 [Chakraborty, Natalie; Rhodes, Stephen; Sheyn, David] Univ Hosp, Urol Inst, Div Female Pelv Med & Reconstruct Surg, Cleveland, OH 44106 USA.

[Chakraborty, Natalie] Univ Hosp Cleveland Med Ctr, Dept Surg, Cleveland, OH USA.

[Luchristt, Douglas] Duke Univ, Dept Obstet & Gynecol, Div Female Pelv Med & Reconstruct Surg, Durham, NC USA.

[Bretschneider, C. Emi] Northwestern Univ, Dept Obstet & Gynecol, Div Female Pelv Med & Reconstruct Surg, Chicago, IL USA.

C3 University Hospitals of Cleveland; University Hospitals of Cleveland;

Duke University; Northwestern University

RP Sheyn, D (corresponding author), Univ Hosp, Urol Inst, Div Female Pelv Med & Reconstruct Surg, Cleveland, OH 44106 USA.

EM David.d.sheyn@gmail.com

RI Luchristt, Douglas/AAV-8819-2021

OI Luchristt, Douglas/0000-0002-3534-7800

NR 40

TC 1

Z9 1

U1 1

U2 1

PU MOSBY-ELSEVIER

PI NEW YORK

PA 360 PARK AVENUE SOUTH, NEW YORK, NY 10010-1710 USA

SN 0002-9378

EI 1097-6868

J9 AM J OBSTET GYNECOL

JI Am. J. Obstet. Gynecol.

PD FEB

PY 2023

VL 228

IS 2

DI 10.1016/j.ajog.2022.09.042

EA JAN 2023

PG 12

WC Obstetrics & Gynecology

WE Science Citation Index Expanded (SCI-EXPANDED)

SC Obstetrics & Gynecology

GA 8X9TX

UT WOS:000932350000001

PM 36202231

DA 2024-01-18

ER

PT J

AU Linder, BJ

Occhino, JA

AF Linder, Brian J. J.

Occhino, John A. A.

TI Robotic-Assisted Surgery for Pelvic Organ Prolapse: Sacrocolpopexy and

Beyond

SO JOURNAL OF GYNECOLOGIC SURGERY

LA English

DT Article

DE Da Vinci device; vagina; vaginal; pelvic organ prolapse; surgery;

sacrocolpopexy

ID SURGICAL-MANAGEMENT; HYSTERECTOMY

AB Pelvic organ prolapse is a highly prevalent condition that can have a large impact on a patient's quality of life. Multiple approaches to surgical repair exist, each with a unique set of risks and benefits. For patients with apical prolapse, repair of the apex is the cornerstone of any surgical procedure. Since adaptation of robotic techniques to sacrocolpopexy were introduced in the early 2000s, the minimally invasive approach to sacrocolpopexy has surpassed open case volumes. Compared to native-tissue transvaginal procedures, minimally invasive sacrocolpopexy offers potential advantages in durability. This article reviews surgical techniques, troubleshooting, outcomes, and ongoing areas of development regarding the use of a robotic approach to prolapse surgery. (J GYNECOLOGIC SURG 2023:000)

C1 [Linder, Brian J. J.] Mayo Clin, Dept Urol, Rochester, MN USA.

[Linder, Brian J. J.; Occhino, John A. A.] Mayo Clin, Dept Obstet & Gynecol, Rochester, MN USA.

[Linder, Brian J. J.] Mayo Clin, Dept Urol, 200 First St,SW, Rochester, MN 55905 USA.

C3 Mayo Clinic; Mayo Clinic; Mayo Clinic

RP Linder, BJ (corresponding author), Mayo Clin, Dept Urol, 200 First St,SW, Rochester, MN 55905 USA.

EM Linder.Brian@mayo.edu

NR 17

TC 0

Z9 0

U1 0

U2 0

PU MARY ANN LIEBERT, INC

PI NEW ROCHELLE

PA 140 HUGUENOT STREET, 3RD FL, NEW ROCHELLE, NY 10801 USA

SN 1042-4067

EI 1557-7724

J9 J GYNECOL SURG

JI J. Gynecol. Surg.

PD FEB 1

PY 2023

VL 39

IS 1

BP 25

EP 29

DI 10.1089/gyn.2022.0119

EA JAN 2023

PG 5

WC Obstetrics & Gynecology; Surgery

WE Emerging Sources Citation Index (ESCI)

SC Obstetrics & Gynecology; Surgery

GA 8W5BE

UT WOS:000911085800001

DA 2024-01-18

ER

PT J

AU Baker, MV

Trad, ATA

Tamhane, P

Weaver, AL

Visscher, SL

Borah, BJ

Klingele, CJ

Gebhart, JB

Trabuco, EC

AF Baker, Mary V.

Teles Abrao Trad, Ayssa

Tamhane, Prajakta

Weaver, Amy L.

Visscher, Sue L.

Borah, Bijan J.

Klingele, Christopher J.

Gebhart, John B.

Trabuco, Emanuel C.

TI Abdominal and robotic sacrocolpopexy costs following implementation of

enhanced recovery after surgery

SO INTERNATIONAL JOURNAL OF GYNECOLOGY & OBSTETRICS

LA English

DT Article

DE cost; enhanced recovery after surgery; liposomal bupivacaine; robotic;

sacrocolpopexy

ID LAPAROSCOPIC SACROCOLPOPEXY; OUTCOMES

AB ObjectiveTo compare perioperative costs and morbidity between open and robotic sacrocolpopexy after implementation of enhanced recovery after surgery (ERAS) pathway. MethodsThe present retrospective cohort study of patients undergoing open or robotic sacrocolpopexy (January 1, 2014, through November 30, 2017) used an ERAS protocol with liposomal bupivacaine infiltration of laparotomy incisions. Primary outcomes were costs associated with index surgery and hospitalization, determined with Medicare cost-to-charge ratios and reimbursement rates and adjusted for variables expected to impact costs. Secondary outcomes included narcotic use, length of stay (LOS), and complications from index hospitalization to postoperative day 30. ResultsFor the total of 231 patients (open cohort, 90; robotic cohort, 141), the adjusted mean cost of robotic surgery was $3239 higher compared with open sacrocolpopexy (95% confidence interval [CI] $1331-$5147; P < 0.001). Rates were not significantly different for intraoperative complications (robotic, 4.3% [6/141]; open, 5.6% [5/90]; P = 0.754), 30-day postoperative complications (robotic, 11.4% [16/141]; open, 16.7% [15/90]; P = 0.322), or readmissions (robotic, 5.7% [8/141]; open, 3.3% [3/90]; P = 0.535). The percentage of patients dismissed on postoperative day 1 was greater in the robotic group (89.4% [126/141] vs. 48.9% [44/90], P < 0.001). ConclusionsDecreased LOS associated with ERAS provided significant cost savings with open sacrocolpopexy versus robotic sacrocolpopexy without adverse impacts on perioperative complications or readmissions.

C1 [Baker, Mary V.; Teles Abrao Trad, Ayssa] Mayo Clin, Sch Grad Med Educ, Dept Obstet & Gynecol, Coll Med & Sci, Rochester, MN USA.

[Tamhane, Prajakta] Reid Hlth, Dept Family Med, Richmond, IN USA.

[Weaver, Amy L.; Borah, Bijan J.] Mayo Clin, Dept Quantitat Hlth Sci, Rochester, MN USA.

[Visscher, Sue L.; Borah, Bijan J.] Mayo Clin, Robert D & Patricia E Kern Ctr Sci Hlth Care Deliv, Rochester, MN USA.

[Borah, Bijan J.; Gebhart, John B.; Trabuco, Emanuel C.] Mayo Clin, Dept Obstet & Gynecol, Rochester, MN USA.

[Klingele, Christopher J.] Olmsted Med Ctr, Obstet & Gynecol, Rochester, MN USA.

[Trabuco, Emanuel C.] Dept Obstet & Gynecol, Mayo Clin, 200 First St SW, Rochester, MN 55905 USA.

C3 Mayo Clinic; Mayo Clinic; Mayo Clinic; Mayo Clinic; Olmsted Medical

Center; Mayo Clinic

RP Trabuco, EC (corresponding author), Dept Obstet & Gynecol, Mayo Clin, 200 First St SW, Rochester, MN 55905 USA.

EM trabuco.emanuel@mayo.edu

NR 23

TC 2

Z9 2

U1 0

U2 0

PU WILEY

PI HOBOKEN

PA 111 RIVER ST, HOBOKEN 07030-5774, NJ USA

SN 0020-7292

EI 1879-3479

J9 INT J GYNECOL OBSTET

JI Int. J. Gynecol. Obstet.

PD MAY

PY 2023

VL 161

IS 2

BP 655

EP 660

DI 10.1002/ijgo.14623

EA JAN 2023

PG 6

WC Obstetrics & Gynecology

WE Science Citation Index Expanded (SCI-EXPANDED)

SC Obstetrics & Gynecology

GA E3RY6

UT WOS:000909460800001

PM 36504261

DA 2024-01-18

ER

PT J

AU Reddy, H

Dellacerra, G

Malcher, F

Plewniak, K

Arabkhazaeli, M

Sankin, A

Lerner, V

AF Reddy, Himabindu

Dellacerra, Gary

Malcher, Flavio

Plewniak, Kari

Arabkhazaeli, Moona

Sankin, Alexander

Lerner, Veronica

TI Excision of triple compartment deep infiltrating endometriosis with

visceral involvement

SO JOURNAL OF ENDOMETRIOSIS AND PELVIC PAIN DISORDERS

LA English

DT Article

DE Deep infiltrating endometriosis; triple compartment; visceral

involvement; laparoscopy; rectal surgery; bladder surgery; hysterectomy

ID ULTRASOUND; DIAGNOSIS; ACCURACY

AB Background: Excision of multi-compartment deep infiltrating endometriosis with visceral involvement is challenging. We illustrate an interdisciplinary approach to complete minimally invasive excision in a single surgery. Case: We present a case of deep infiltrating endometriosis with visceral involvement in the anterior, middle, and posterior compartments. A collaborative surgical approach was taken with gynecologic, colorectal, and urologic surgeons to perform a robot-assisted total laparoscopic hysterectomy, bilateral salpingectomy, ovarian cystectomy, and unilateral oophorectomy with concurrent segmental resection of rectosigmoid and excision of transmural bladder and vaginal nodules. Conclusion: Thorough preoperative evaluation and an interdisciplinary approach to surgical planning involving radiology, gynecology, colorectal surgery, and urology allowed for complete simultaneous resection of bladder, rectosigmoid, and pelvic deep infiltrating endometriosis without complications via a minimally invasive route.

C1 [Reddy, Himabindu] Montefiore Med Ctr, Dept Obstet & Gynecol, Bronx, NY USA.

[Reddy, Himabindu; Dellacerra, Gary; Plewniak, Kari; Sankin, Alexander] Einstein Coll Med, Bronx, NY USA.

[Dellacerra, Gary] Montefiore Med Ctr, Dept Radiol, Bronx, NY USA.

[Malcher, Flavio] NYU, Grossman Sch Med, Dept Surg, New York, NY USA.

[Plewniak, Kari] Montefiore Med Ctr, Dept Obstet & Gynecol, Bronx, NY USA.

[Arabkhazaeli, Moona] Minnesota Womens Care, 2603 White Bear Ave North, Maplewood, MN USA.

[Sankin, Alexander] Montefiore Med Ctr, Dept Urol, Bronx, NY USA.

[Lerner, Veronica] Lenox Hill Hosp, Zucker Sch Med Hofstra Northwell Hlth, Dept Obstet & Gynecol, New York, NY USA.

[Lerner, Veronica] Zucker Sch Med Hofstra Northwell Hlth, Lenox Hill Hosp, Dept Obstet & Gynecol, New York, NY 10075 USA.

C3 Yeshiva University; Albert Einstein College of Medicine; Montefiore

Medical Center; Yeshiva University; Albert Einstein College of Medicine;

Yeshiva University; Albert Einstein College of Medicine; Montefiore

Medical Center; New York University; Montefiore Medical Center; Yeshiva

University; Albert Einstein College of Medicine; Montefiore Medical

Center; Yeshiva University; Albert Einstein College of Medicine;

Northwell Health; Northwell Health

RP Lerner, V (corresponding author), Zucker Sch Med Hofstra Northwell Hlth, Lenox Hill Hosp, Dept Obstet & Gynecol, New York, NY 10075 USA.

EM Lernervt02@gmail.com

NR 16

TC 0

Z9 0

U1 0

U2 0

PU SAGE PUBLICATIONS LTD

PI LONDON

PA 1 OLIVERS YARD, 55 CITY ROAD, LONDON EC1Y 1SP, ENGLAND

SN 2284-0265

EI 2284-0273

J9 J ENDOMETR PELVIC PA

JI J. Endometr. Pelvic Pain Disord.

PD DEC

PY 2022

VL 14

IS 4

BP 217

EP 222

DI 10.1177/22840265221146479

EA JAN 2023

PG 6

WC Obstetrics & Gynecology

WE Emerging Sources Citation Index (ESCI)

SC Obstetrics & Gynecology

GA 8U5BC

UT WOS:000909851600001

DA 2024-01-18

ER

PT J

AU Galhotra, S

Zeng, KT

Hu, CC

Norton, T

Mahnert, N

Smith, R

Mourad, J

AF Galhotra, Sheena

Zeng, Katie

Hu, Chengcheng

Norton, Taylor

Mahnert, Nichole

Smith, Rachael

Mourad, Jamal

TI The Effect of Patient Positioning on Ureteral Efflux During

Intraoperative Cystoscopy: A Randomized Controlled Trial

SO JOURNAL OF MINIMALLY INVASIVE GYNECOLOGY

LA English

DT Article

DE Intraoperative cystoscopy; Laparoscopy; Patient positioning; Ureteral

injury; Ureteral patency

ID BENIGN GYNECOLOGIC SURGERY; URINARY-TRACT INJURY; HYSTERECTOMY; PATENCY

AB Study Objective: To identify the relationship between patient position during surgery and time to confirmation of ureteral patency on cystoscopy.Design: Randomized controlled trial.Setting: Academic tertiary care medical center.Patients or Participants: A total of 91 adult women undergoing laparoscopic (either conventional or robotic) hysterectomy between February 2021 and February 2022 were randomized to intervention (n = 45) or control (n = 46). Exclusion criteria included known kidney disease or urinary tract anomaly, current ureteral stent, pregnancy, malignancy, and recognized intraoperative urinary tract injury. Interventions: Subjects in the control group were placed in a 0 degrees supine position during cystoscopy. Subjects in the interven-tion group were placed in a 20 degrees angle in reverse Trendelenburg (RT) position during cystoscopy.Measurements and Main Results: The primary outcome, time to confirmation of bilateral ureteral patency, was measured at the time the second ureteral jet was viewed during intraoperative cystoscopy. There was no significant difference in mean time to confirmation (66.5 seconds in supine vs 67 seconds in RT, p = .2) nor in total cystoscopy time (111 seconds in supine vs 104.5 seconds in RT, p = .39). There were no significant differences in need for alternative modalities to aid in ureteral efflux visualization, delayed diagnosis of ureteric injury, and operative time. RT position seemed to have reduced the time to confirmation for the small group of patients with longer confirmation time (>120 seconds).Conclusion: RT position does not change time to confirmation of bilateral ureteral patency compared with supine position. However, there may be a benefit in position change if time to confirmation is >120 seconds. Journal of Minimally Invasive Gynecology (2023) 30, 13-18.(c) 2022 AAGL. All rights reserved.

C1 [Galhotra, Sheena; Zeng, Katie; Hu, Chengcheng; Norton, Taylor; Mahnert, Nichole; Smith, Rachael; Mourad, Jamal] Banner Univ Med Ctr, Dept Minimally Invas Gynecol Surg Obstet & Gynecol, Phoenix, AZ USA.

[Galhotra, Sheena] Banner Univ Med Ctr, Dept Obstet & Gynecol, 1441 N 12th St, 3rd Floor 3032, Phoenix, AZ 85006 USA.

RP Galhotra, S (corresponding author), Banner Univ Med Ctr, Dept Obstet & Gynecol, 1441 N 12th St, 3rd Floor 3032, Phoenix, AZ 85006 USA.

EM sheenagalhotra@gmail.com

OI Galhotra, Sheena/0000-0002-3893-1041

NR 20

TC 0

Z9 0

U1 1

U2 5

PU ELSEVIER SCIENCE INC

PI NEW YORK

PA STE 800, 230 PARK AVE, NEW YORK, NY 10169 USA

SN 1553-4650

EI 1553-4669

J9 J MINIM INVAS GYN

JI J. Minim. Invasive Gynecol.

PD JAN

PY 2023

VL 30

IS 1

BP 13

EP 18

DI 10.1016/j.jmig.2022.09.003

EA JAN 2023

PG 6

WC Obstetrics & Gynecology

WE Science Citation Index Expanded (SCI-EXPANDED)

SC Obstetrics & Gynecology

GA 8H5VC

UT WOS:000921100000001

PM 36103970

DA 2024-01-18

ER

PT J

AU Jiang, SL

Li, Z

Ma, X

Ma, XH

Meng, YG

Ye, MX

Zhang, L

Zhang, P

Zhang, NN

Zhang, Y

Wang, N

Li, L

AF Jiang, Shengli

Li, Zhen

Ma, Xin

Ma, Xiaohui

Meng, Yuanguang

Ye, Mingxia

Zhang, Lin

Zhang, Peng

Zhang, Nina

Zhang, Yue

Wang, Nan

Li, Lian

TI Robotic-assisted laparoscopic and thoracoscopic approach: a challenging

multidisciplinary minimally invasive surgery of intravascular

leiomyomatosis with intracardiac extension

SO FERTILITY AND STERILITY

LA English

DT Article

DE Intravascular leiomyomatosis; robotic-assisted surgery; thoracoscopic

surgery

ID INTRAVENOUS LEIOMYOMATOSIS

AB Objective: To show a case of severe intravascular leiomyomatosis with intracardiac extension treated by a multidisciplinary minimally invasive surgery.Design: Stepwise demonstration of the technique with a video. Setting: General Hospital.Patient(s): A 40-year-old woman with palpitation and dyspnea. Intervention(s): The patient was diagnosed with intravascular leiomyomatosis by computed tomography scan. She underwent a suc-cessful single-stage minimally invasive surgery with complete excision.Main Outcome Measure(s): The feasibility and safety of using this technique for intravascular leiomyomatosis with intracardiac extension.Result(s): A combined thoracoabdominal surgery was successfully performed. During the procedure, cardiopulmonary bypass was maintained for 72 minutes. The patient soon recovered and was discharged.Conclusion(s): Minimally invasive surgery is a possible choice for intravascular leiomyomatosis with intracardiac extension. (Fertil Steriln 2023;119:155-57.(c) 2022 by American Society for Reproductive Medicine.)

C1 [Jiang, Shengli; Zhang, Lin] Chinese Peoples Liberat Army Gen Hosp, Dept Cardiovasc Surg, Beijing, Peoples R China.

[Li, Zhen; Meng, Yuanguang; Ye, Mingxia; Zhang, Nina; Wang, Nan; Li, Lian] Chinese Peoples Liberat Army Gen Hosp, Dept Obstet & Gynecol, Beijing, Peoples R China.

[Ma, Xin; Zhang, Peng] Chinese Peoples Liberat Army Gen Hosp, Dept Urol, Beijing, Peoples R China.

[Ma, Xiaohui] Chinese Peoples Liberat Army Gen Hosp, Dept Vasc & Endovascular Surg, Beijing, Peoples R China.

[Zhang, Yue] Chinese PLA 988 Hosp, Dept Obstet & Gynecol, Beijing, Henan, Peoples R China.

[Li, Lian] Chinese Peoples Liberat Army Gen Hosp, Dept Obstet & Gynecol, 28 Fuxing Rd, Beijing 100089, Peoples R China.

C3 Chinese People's Liberation Army General Hospital; Chinese People's

Liberation Army General Hospital; Chinese People's Liberation Army

General Hospital; Chinese People's Liberation Army General Hospital;

Chinese People's Liberation Army General Hospital

RP Li, L (corresponding author), Chinese Peoples Liberat Army Gen Hosp, Dept Obstet & Gynecol, 28 Fuxing Rd, Beijing 100089, Peoples R China.

EM llapla@163.com

RI meng, yuan/HZJ-3227-2023; Meng, yuan/JRY-8417-2023

NR 16

TC 0

Z9 1

U1 0

U2 4

PU ELSEVIER SCIENCE INC

PI NEW YORK

PA STE 800, 230 PARK AVE, NEW YORK, NY 10169 USA

SN 0015-0282

EI 1556-5653

J9 FERTIL STERIL

JI Fertil. Steril.

PD JAN

PY 2023

VL 119

IS 1

BP 155

EP 157

DI 10.1016/j.fertnstert.2022.09.022

EA JAN 2023

PG 3

WC Obstetrics & Gynecology; Reproductive Biology

WE Science Citation Index Expanded (SCI-EXPANDED)

SC Obstetrics & Gynecology; Reproductive Biology

GA 8E3EW

UT WOS:000918862000001

PM 36400596

OA hybrid

DA 2024-01-18

ER

PT J

AU Correa-Paris, A

Ochoa, VG

Gutiérrez, AH

Estellés, JG

Díaz-Feijoo, B

Gil-Moreno, A

AF Correa-Paris, Alejandro

Gorraiz Ochoa, Veronica

Hernandez Gutierrez, Alicia

Gilabert Estelles, Juan

Diaz-Feijoo, Berta

Gil-Moreno, Antonio

TI Simple radiologic assessment of visceral obesity and prediction of

surgical morbidity in endometrial cancer patients undergoing

laparoscopic aortic lymphadenectomy: A reliability and accuracy study

SO JOURNAL OF OBSTETRICS AND GYNAECOLOGY RESEARCH

LA English

DT Article

DE complications; endometrial carcinoma; intra-abdominal fat;

lymphadenectomy; minimally invasive surgical procedures

ID SAGITTAL ABDOMINAL DIAMETER; TOTAL MESORECTAL EXCISION; BODY-MASS INDEX;

COMPLICATIONS; SURGERY; RISK; OUTCOMES; IMPACT; EXTRAPERITONEAL;

TRANSPERITONEAL

AB AimTo evaluate the reliability of sagittal abdominal diameter (SAD)-a surrogate of visceral obesity-in magnetic resonance imaging, and its accuracy to predict the surgical morbidity of aortic lymphadenectomy.MethodsWe conducted a multicenter reliability (phase 1) and accuracy (phase 2) cohort study in three Spanish referral hospitals. We retrospectively analyzed data from the STELLA-2 randomized controlled trial that included high-risk endometrial cancer patients undergoing minimally invasive surgical staging. Patients were classified into subgroups: conventional versus robotic-assisted laparoscopy, and transperitoneal versus extraperitoneal technique. In the first phase, we measured the agreement of three SAD measurements (at the umbilicus, renal vein, and inferior mesenteric artery) and selected the most reliable one. In phase 2, we evaluated the diagnostic accuracy of SAD to predict surgical morbidity. Surgical morbidity was the main outcome measure, it was defined by a core outcome set including variables related to blood loss, operative time, surgical complications, and para-aortic lymphadenectomy difficulty.ResultsIn phase 1, all measurements showed good inter-rater and intra-rater agreement. Umbilical SAD (u-SAD) was the most reliable one. In phase 2, we included 136 patients. u-SAD had a good diagnostic accuracy to predict surgical morbidity in patients undergoing transperitoneal laparoscopic lymphadenectomy (0.73 in ROC curve). It performed better than body mass index and other anthropometric measurements. We calculated a cut-off point of 246 mm (sensitivity: 0.56, specificity: 0.80).Conclusionsu-SAD is a simple, reliable, and potentially useful measurement to predict surgical morbidity in endometrial cancer patients undergoing minimally invasive surgical staging, especially when facing transperitoneal aortic lymphadenectomy.

C1 [Correa-Paris, Alejandro; Gorraiz Ochoa, Veronica; Diaz-Feijoo, Berta; Gil-Moreno, Antonio] Univ Autonoma Barcelona, Obstet & Gynecol Dept, Vall dHebron Barcelona Hosp Campus, Barcelona, Spain.

[Hernandez Gutierrez, Alicia] Hosp Univ La Paz, Obstet & Gynecol Dept, Madrid, Spain.

[Gilabert Estelles, Juan] Hosp Gen Valencia, Obstet & Gynecol Dept, Valencia, Spain.

[Gilabert Estelles, Juan] Univ Valencia, Valencia, Spain.

[Gil-Moreno, Antonio] Vall dHebron Inst Recerca, Biomed Res Grp Gynecol, Barcelona, Spain.

[Correa-Paris, Alejandro; Gorraiz Ochoa, Veronica] HIRU Hlth Independent Res United, C Virgen Codes 8, Pamplona 31011, Navarra, Spain.

[Diaz-Feijoo, Berta] Hosp Clin Barcelona, Inst Clin Gynecol Obstet & Neonatol, C Villarroel 170, Barcelona, Spain.

C3 Autonomous University of Barcelona; Hospital Universitario La Paz;

University of Valencia; Autonomous University of Barcelona; Hospital

Universitari Vall d'Hebron; Vall d'Hebron Institut de Recerca (VHIR);

University of Barcelona; Hospital Clinic de Barcelona

RP Correa-Paris, A (corresponding author), HIRU Hlth Independent Res United, C Virgen Codes 8, Pamplona 31011, Navarra, Spain.

EM acorreap@hiru-research.org

RI Gil-Moreno, Antonio/C-1122-2016; Gilabert-Estelles, Juan/ABH-9381-2020

OI Gil-Moreno, Antonio/0000-0003-1106-5590; Gilabert-Estelles,

Juan/0000-0002-3992-1929; Diaz-Feijoo, Berta/0000-0002-6451-1817

NR 43

TC 0

Z9 0

U1 0

U2 1

PU WILEY

PI HOBOKEN

PA 111 RIVER ST, HOBOKEN 07030-5774, NJ USA

SN 1341-8076

EI 1447-0756

J9 J OBSTET GYNAECOL RE

JI J. Obstet. Gynaecol. Res.

PD MAR

PY 2023

VL 49

IS 3

BP 988

EP 997

DI 10.1111/jog.15528

EA JAN 2023

PG 10

WC Obstetrics & Gynecology

WE Science Citation Index Expanded (SCI-EXPANDED)

SC Obstetrics & Gynecology

GA D9BM9

UT WOS:000906800200001

PM 36593218

DA 2024-01-18

ER

PT J

AU Alboni, C

Mattos, LC

La Marca, A

Raimondo, D

Casadio, P

Seracchioli, R

Gaia, G

AF Alboni, Carlo

Mattos, Ludovica Camacho

La Marca, Antonio

Raimondo, Diego

Casadio, Paolo

Seracchioli, Renato

Gaia, Giorgia

TI Robotic Surgery and Deep Infiltrating Endometriosis Treatment: The State

of Art

SO CLINICAL AND EXPERIMENTAL OBSTETRICS & GYNECOLOGY

LA English

DT Review

DE robotic surgery; deep endometriosis; laparoscopy

ID PELVIC PAIN; LAPAROSCOPIC EXCISION; ASSISTED LAPAROSCOPY; LIFE

INSTRUMENT; CLASSIFICATION; HYSTERECTOMY; MANAGEMENT; HEALTH;

COMPLICATIONS; CYSTECTOMY

AB Objective: Surgical treatment of endometriosis, when indicated, has demonstrated to be effective in reducing painful symptoms and improve quality of life of patients affected with endometriosis. The minimally invasive approach via laparoscopy is the preferred method when compared with laparotomy but in the last two decades another minimally invasive approach has become available, the robotically assisted laparoscopic surgery. Robotic technology is widely used in different surgical branches, such as general surgery and urology. Moreover, the use of robotic surgery is already accepted for different gynecological procedures either for benign and for oncological diseases. The advantages of robotic surgery such as improve dexterity of movements, avoided tremor, increased magnification of 3 -dimensional vision seem strategic in the context of a complex surgery as is deep endometriosis eradication. However, to date there is no unanimous consensus on whether robotically assisted procedures are a valid and safe alternative to laparoscopy in the treatment of endometriosis. Mechanism: In this narrative review we analyze the available literature assessesing the robotic treatment of all types of endometriosis and specifically deep infiltrating endometriosis, compared to the outcomes of conventional laparoscopy. Findings in Brief: Indeed, the evidence of safety and effectiveness of robotically assisted laparoscopy in endometriosis treatment is strong and almost unanimous. There is no clear superiority of one approach to the other but robotic-related advantages and future prospective are promising to be able to improve operative outcomes, reduce surgeon's fatigue and provide a technology easy to implement with a fast learning curve. Conclusions: Robotic technology applied to laparoscopy in the treatment of endometriosis could be seen as an effective and safe alternative to the conventional laparoscopic treatment.

C1 [Alboni, Carlo] Univ Hosp Modena, Minimally Invas Gynecol Surg Unit, I-41124 Modena, Italy.

[Mattos, Ludovica Camacho] Osped Civile Sassuolo, Ostetricia & Ginecol, I-41049 Modena, Italy.

[La Marca, Antonio] Univ Modena & Reggio Emilia, Dept Med & Surg Sci Children & Adults, I-41124 Modena, Italy.

[Raimondo, Diego; Casadio, Paolo; Seracchioli, Renato] Univ Bologna, Gynecol & Human Reprod Physiopatol, IRCCS Azienda Osped, I-40138 Bologna, Italy.

[Raimondo, Diego; Seracchioli, Renato] Univ Bologna, Dept Med & Surg Sci, I-40138 Bologna, Italy.

[Gaia, Giorgia] San Paolo Hosp, Dept Obstet & Gynecol, ASST Santi Paolo & Carlo, Med Sch, I-20142 Milan, Italy.

C3 Universita di Modena e Reggio Emilia; Universita di Modena e Reggio

Emilia Hospital; Universita di Modena e Reggio Emilia; University of

Bologna; University of Bologna; San Paolo-Polo Universitaria Hospital

RP Mattos, LC (corresponding author), Osped Civile Sassuolo, Ostetricia & Ginecol, I-41049 Modena, Italy.

EM ludovica.camacho@gmail.com

RI Raimondo, Diego/AAC-4332-2021; Casadio, Paolo/AAM-4092-2021

OI Casadio, Paolo/0000-0001-7706-5580

NR 67

TC 0

Z9 0

U1 2

U2 2

PU IMR PRESS

PI ROBINSON

PA 112 ROBINSON RD, ROBINSON, SINGAPORE

SN 0390-6663

EI 2709-0094

J9 CLIN EXP OBSTET GYN

JI Clin. Exp. Obstet. Gynecol.

PD JAN

PY 2023

VL 50

IS 1

AR 13

DI 10.31083/j.ceog5001013

PG 10

WC Obstetrics & Gynecology

WE Science Citation Index Expanded (SCI-EXPANDED)

SC Obstetrics & Gynecology

GA 8R0CV

UT WOS:000927565100004

OA gold

DA 2024-01-18

ER

PT J

AU Dinoi, G

Ghoniem, K

Murad, MH

Segarra-Vidal, B

Zanfagnin, V

Coronado, PJ

Kyrgiou, M

Perrone, AM

Zola, P

Weaver, A

McGree, M

Fanfani, F

Scambia, G

Ramirez, PT

Mariani, A

AF Dinoi, Giorgia

Ghoniem, Khaled

Murad, M. Hassan

Segarra-Vidal, Blanca

Zanfagnin, Valentina

Coronado, Pluvio J.

Kyrgiou, Maria

Perrone, Anna M.

Zola, Paolo

Weaver, Amy

McGree, Michaela

Fanfani, Francesco

Scambia, Giovanni

Ramirez, Pedro T.

Mariani, Andrea

TI Minimally Invasive Compared With Open Surgery in High-Risk Endometrial

Cancer <i>A Systematic Review and Meta-analysis</i>

SO OBSTETRICS AND GYNECOLOGY

LA English

DT Review

ID CLEAR-CELL CARCINOMA; ROBOTIC SURGERY; PERIOPERATIVE OUTCOMES;

UTERINE-CANCER; EARLY-STAGE; LAPAROSCOPY; LAPAROTOMY; WOMEN; PAPILLARY;

SURVIVAL

AB OBJECTIVE: To compare outcomes between minimally invasive surgery and open surgery in patients with high-risk endometrial cancer. DATA SOURCES: A cohort study of all patients who underwent surgery for high-risk endometrial cancer between 1999 and 2016 at Mayo Clinic (Rochester, Minnesota) and a literature search of MEDLINE, EMBASE, , Cochrane Central Register of Controlled Trials, and Scopus of all published studies until December 2020. METHODS OF STUDY SELECTION: The systematic review identified 2,332 patients (14 studies, all retrospective except a subanalysis of a randomized comparison) and the cohort study identified 542 additional patients. Articles were included if reporting original data on overall survival and disease-free survival among patients with high-risk endometrial cancer, defined as International Federation of Gynecology and Obstetrics grade 3 endometrioid, serous, clear cell, mixed histology, or uterine carcinosarcoma. Studies that did not report at least one of the main outcomes, those in which one surgical technique (robotic or laparoscopic surgery) was missing in the comparison analysis with open surgery, and case reports were excluded. Additional data were extracted from a retrospective cohort of patients from Mayo. A random-effect model was used for meta-analysis. TABULATION, INTEGRATION, AND RESULTS: This systematic review and meta-analysis was registered in PROSPERO. Literature search and data extraction were performed independently by two reviewers, as well as quality assessment using GRADE (Grading of Recommendations Assessment, Development and Evaluation) methodology, and the Newcastle-Ottawa Scale. PRISMA (Preferred Reporting Items for Systematic Reviews and Meta-Analyses) guidelines were followed. Meta-analysis showed that disease-free survival and overall survival in patients with high-risk endometrial cancer who underwent minimally invasive surgery were not statistically different from those of patients who underwent open abdominal surgery (relative risk [RR] 0.93, 95% CI 0.82-1.05, I-2 20%, P=.23; and RR 0.92, 95% CI 0.77-1.11, I-2 31%, P=.12, respectively). Subgroup analysis by stage (early vs advanced) did not identify a difference between surgical approaches. CONCLUSION: Minimally invasive surgery and open surgery had similar disease-free survival and overall survival in patients with high-risk endometrial cancer.

C1 [Dinoi, Giorgia; Ghoniem, Khaled; Murad, M. Hassan; Segarra-Vidal, Blanca; Zanfagnin, Valentina; Coronado, Pluvio J.; Kyrgiou, Maria; Perrone, Anna M.; Zola, Paolo; Weaver, Amy; McGree, Michaela; Fanfani, Francesco; Scambia, Giovanni; Ramirez, Pedro T.; Mariani, Andrea] Fdn Policlin Univ A Gemelli IRCCS, Dept Woman Child & Publ Hlth, Gynecol Oncol Unit, Rome, Italy.

[Dinoi, Giorgia; Ghoniem, Khaled; Murad, M. Hassan; Segarra-Vidal, Blanca; Zanfagnin, Valentina; Coronado, Pluvio J.; Kyrgiou, Maria; Perrone, Anna M.; Zola, Paolo; Weaver, Amy; McGree, Michaela; Fanfani, Francesco; Scambia, Giovanni; Ramirez, Pedro T.; Mariani, Andrea] Univ Bologna, IRCCS Azienda Ospedaliera, Bologna, Italy.

[Dinoi, Giorgia; Ghoniem, Khaled; Murad, M. Hassan; Segarra-Vidal, Blanca; Zanfagnin, Valentina; Coronado, Pluvio J.; Kyrgiou, Maria; Perrone, Anna M.; Zola, Paolo; Weaver, Amy; McGree, Michaela; Fanfani, Francesco; Scambia, Giovanni; Ramirez, Pedro T.; Mariani, Andrea] Univ Turin, Dept Surg Sci, Turin, Italy.

[Dinoi, Giorgia; Ghoniem, Khaled; Murad, M. Hassan; Segarra-Vidal, Blanca; Zanfagnin, Valentina; Coronado, Pluvio J.; Kyrgiou, Maria; Perrone, Anna M.; Zola, Paolo; Weaver, Amy; McGree, Michaela; Fanfani, Francesco; Scambia, Giovanni; Ramirez, Pedro T.; Mariani, Andrea] Mayo Clin, Kern Ctr Sci Healthcare Deliver, Dept Obstet & Gynecol, Div Gynecol Surg, Rochester, MN USA.

[Dinoi, Giorgia; Ghoniem, Khaled; Murad, M. Hassan; Segarra-Vidal, Blanca; Zanfagnin, Valentina; Coronado, Pluvio J.; Kyrgiou, Maria; Perrone, Anna M.; Zola, Paolo; Weaver, Amy; McGree, Michaela; Fanfani, Francesco; Scambia, Giovanni; Ramirez, Pedro T.; Mariani, Andrea] Mayo Clin, Dept Hlth Sci Res, Div Biomed Stat & Informat, Rochester, MN USA.

[Dinoi, Giorgia; Ghoniem, Khaled; Murad, M. Hassan; Segarra-Vidal, Blanca; Zanfagnin, Valentina; Coronado, Pluvio J.; Kyrgiou, Maria; Perrone, Anna M.; Zola, Paolo; Weaver, Amy; McGree, Michaela; Fanfani, Francesco; Scambia, Giovanni; Ramirez, Pedro T.; Mariani, Andrea] La Fe Univ, Polytech Hosp, Dept Gynecol Oncol, Valencia, Spain.

[Dinoi, Giorgia; Ghoniem, Khaled; Murad, M. Hassan; Segarra-Vidal, Blanca; Zanfagnin, Valentina; Coronado, Pluvio J.; Kyrgiou, Maria; Perrone, Anna M.; Zola, Paolo; Weaver, Amy; McGree, Michaela; Fanfani, Francesco; Scambia, Giovanni; Ramirez, Pedro T.; Mariani, Andrea] Hosp Clin San Carlos, Dept Obstet & Ginecol, Madrid, Spain.

[Dinoi, Giorgia; Ghoniem, Khaled; Murad, M. Hassan; Segarra-Vidal, Blanca; Zanfagnin, Valentina; Coronado, Pluvio J.; Kyrgiou, Maria; Perrone, Anna M.; Zola, Paolo; Weaver, Amy; McGree, Michaela; Fanfani, Francesco; Scambia, Giovanni; Ramirez, Pedro T.; Mariani, Andrea] Imperial Coll Healthcare NHS Trust, Imperial Coll, Fac Med, Dept Metab Digest & Reprod,Surg & Canc, London, England.

[Dinoi, Giorgia; Ghoniem, Khaled; Murad, M. Hassan; Segarra-Vidal, Blanca; Zanfagnin, Valentina; Coronado, Pluvio J.; Kyrgiou, Maria; Perrone, Anna M.; Zola, Paolo; Weaver, Amy; McGree, Michaela; Fanfani, Francesco; Scambia, Giovanni; Ramirez, Pedro T.; Mariani, Andrea] Imperial Coll Healthcare NHS Trust, West London Gynaecol Canc Ctr, London, England.

[Dinoi, Giorgia; Ghoniem, Khaled; Murad, M. Hassan; Segarra-Vidal, Blanca; Zanfagnin, Valentina; Coronado, Pluvio J.; Kyrgiou, Maria; Perrone, Anna M.; Zola, Paolo; Weaver, Amy; McGree, Michaela; Fanfani, Francesco; Scambia, Giovanni; Ramirez, Pedro T.; Mariani, Andrea] Univ Texas MD Anderson Canc Ctr, Dept Gynecol Oncol & Reprod Med, Houston, TX USA.

[Mariani, Andrea] Mayo Clin, Dept Obstet & Gynecol, Div Gynecol Surg, Rochester, MN 55902 USA.

C3 Catholic University of the Sacred Heart; IRCCS Policlinico Gemelli;

University of Bologna; University of Turin; Mayo Clinic; Mayo Clinic;

Hospital Clinico San Carlos; Imperial College London; Imperial College

London; University of Texas System; UTMD Anderson Cancer Center; Mayo

Clinic

RP Mariani, A (corresponding author), Mayo Clin, Dept Obstet & Gynecol, Div Gynecol Surg, Rochester, MN 55902 USA.

EM mariani.andrea@mayo.edu

RI Perrone, Anna Myriam/AAR-9590-2020; Scambia, Giovanni/K-7539-2016;

Fanfani, Francesco/Q-1154-2015; Perrone, Anna Myriam/JED-6362-2023

OI Perrone, Anna Myriam/0000-0003-3140-4772; Scambia,

Giovanni/0000-0002-9503-9041; Fanfani, Francesco/0000-0003-1991-7284;

Perrone, Anna Myriam/0000-0003-3140-4772

NR 44

TC 3

Z9 3

U1 2

U2 3

PU LIPPINCOTT WILLIAMS & WILKINS

PI PHILADELPHIA

PA TWO COMMERCE SQ, 2001 MARKET ST, PHILADELPHIA, PA 19103 USA

SN 0029-7844

J9 OBSTET GYNECOL

JI Obstet. Gynecol.

PD JAN

PY 2023

VL 141

IS 1

BP 59

EP 68

DI 10.1097/AOG.0000000000004995

PG 10

WC Obstetrics & Gynecology

WE Science Citation Index Expanded (SCI-EXPANDED)

SC Obstetrics & Gynecology

GA 8O9UW

UT WOS:000926177900008

PM 36701610

DA 2024-01-18

ER

PT J

AU Mereu, L

Gaia, G

Afonina, M

Terzoni, S

Tateo, S

Spinillo, A

AF Mereu, Liliana

Gaia, Giorgia

Afonina, Margarita

Terzoni, Stefano

Tateo, Saverio

Spinillo, Arsenio

TI ?Less is More, is R-LESS More??-The Use of Robotic Laparoendoscopic

Single-Site Surgery in Gynaecology: A Scoping Review

SO CLINICAL AND EXPERIMENTAL OBSTETRICS & GYNECOLOGY

LA English

DT Review

DE single-site surgery; robotic surgery; review

ID TOTAL LAPAROSCOPIC HYSTERECTOMY; ENDOMETRIAL CANCER; ASSISTED

SACROCOLPOPEXY; PORT; FEASIBILITY; MYOMECTOMY; 1ST; METAANALYSIS;

GUIDANCE

AB Background: Since the Da Vinci system was introduced in the gynaecological profession, for benign and most malignant procedures, it appeared that using 5 incisions for trocar insertion could jeopardize the system's mini-invasiveness. To protect this important characteristic, robotic laparoendoscopic single-site surgery was developed and authorized for gynaecological use in 2013. Using a single small incision for the entire treatment appears to be a promising attempt to improve cosmetic results while lowering wound infections, postoperative pain, and recovery time. After nearly ten years of use, several limitations of this technique became apparent, such as a limited set of non-articulating instruments and electrical possibilities compared to multiport surgery, smoke evacuation and visual impairment. By examining the most relevant research, the goal of this review was to emphasize the indications, risks, and benefits of R-LESS in gynaecological surgery. Methods: A scoping review was conducted on Pubmed, Scopus, Web of Science, and Embase. Publications in English or Italian in the previous 10 years on the use of single-site robotic surgery in gynaecology for benign disorders were included. Results: This review includes 37 of the 297 papers that were retrieved. Myomectomy, hysterectomy, pelvic floor surgery, and endometriosis were the most common indications for single-site surgery. Several studies have reported R-LESS usage in cancer patients. According to the data analysis, the R-LESS approach is comparable to robotic multi-port surgery as regards feasibility and safety, with faster operative and postoperative durations, reduced pain, and a superior cosmetic outcome. Conclusions: The single-port robotic technique is gaining popularity. Our findings provide preliminary evidence of the global experience of surgical teams. Standardizing operative durations and conducting comparative research on the R-LESS learning curve represent one of the most significant future difficulties, as do surgical outcomes, costs, and patient satisfaction in the long run.

C1 [Mereu, Liliana] ASST Santi Paolo & Carlo, San Paolo Bachelor Sch Nursing, I-20142 Milan, Italy.

[Gaia, Giorgia; Afonina, Margarita; Terzoni, Stefano] ASST Santi Paolo & Carlo, San Paolo Hosp, Med Sch, Dept Obstet & Gynecol, I-20142 Milan, Italy.

[Tateo, Saverio] Univ Parma, Dept Chem Life Sci & Environm Sustainabil, Lab Probiogen, I-43124 Parma, Italy.

[Spinillo, Arsenio] Univ Pavia, IRCCS Fdn Policlin San Matteo, Dept Obstet & Gynecol, I-27100 Pavia, Italy.

C3 San Paolo-Polo Universitaria Hospital; University of Parma; IRCCS

Fondazione San Matteo; University of Pavia

RP Terzoni, S (corresponding author), ASST Santi Paolo & Carlo, San Paolo Hosp, Med Sch, Dept Obstet & Gynecol, I-20142 Milan, Italy.

EM stefano.terzoni@unimi.it

RI Mereu, Liliana/AAW-3039-2021; Terzoni, Stefano/AAH-7582-2021; Afonina,

Margarita/IAM-3678-2023

OI Mereu, Liliana/0000-0002-0610-5132; Terzoni,

Stefano/0000-0002-0716-5663; Afonina, Margarita/0000-0002-7126-3866

NR 64

TC 0

Z9 0

U1 0

U2 0

PU IMR PRESS

PI ROBINSON

PA 112 ROBINSON RD, ROBINSON, SINGAPORE

SN 0390-6663

EI 2709-0094

J9 CLIN EXP OBSTET GYN

JI Clin. Exp. Obstet. Gynecol.

PD JAN

PY 2023

VL 50

IS 1

AR 19

DI 10.31083/j.ceog5001019

PG 9

WC Obstetrics & Gynecology

WE Science Citation Index Expanded (SCI-EXPANDED)

SC Obstetrics & Gynecology

GA 8R0CV

UT WOS:000927565100008

OA gold

DA 2024-01-18

ER

PT J

AU Michal, M

Valha, P

Veleminsky, M

AF Michal, M.

Valha, P.

Veleminsky, M.

TI Sentinel lymph node mapping in endometrial cancer-robotic vs.

laparoscopic detection system

SO CESKA GYNEKOLOGIE-CZECH GYNAECOLOGY

LA English

DT Article

DE endometrial cancer; sentinel lymph node; indocyanine green; Da Vinci

Firefly; Novadaq Pinpoint

ID CARCINOMA; INJECTION
[truncated: 4,550,460 more chars]
